# Effects of alcohol consumption on employment and social outcomes: a Mendelian randomisation study

**DOI:** 10.1093/alcalc/agaf038

**Published:** 2025-07-18

**Authors:** Desmond D Campbell, Michael Green, Neil M Davies, Sean Harrison, Evangelia Demou, Laura D Howe, Marcus R Munafò, Srinivasa Vittal Katikireddi

**Affiliations:** MRC/CSO Social & Public Health Sciences Unit, University of Glasgow, Clarice Pears Building, 90 Byres Road, Glasgow, G12 8TB, United Kingdom; MRC/CSO Social & Public Health Sciences Unit, University of Glasgow, Clarice Pears Building, 90 Byres Road, Glasgow, G12 8TB, United Kingdom; Department of Obstetrics & Gynecology, Duke University School of Medicine, DUMC 3084, 203 Baker House, Durham, NC 27710, United States; MRC Integrative Epidemiology Unit (IEU), Population Health Sciences, Bristol Medical School, University of Bristol, Oakfield House, Oakfield Grove, Bristol, BS8 2BN, United Kingdom; Division of Psychiatry, University College London, Maple House, 149 Tottenham Court Rd, London, W1T 7NF, United Kingdom; Department of Statistical Sciences, University College London, Gower Street, London, WC1E 6BT, United Kingdom; K.G. Jebsen Center for Genetic Epidemiology, Department of Public Health and Nursing, NTNU, Norwegian University of Science and Technology, Postboks 8900, Torgarden, NO-7491 Trondheim, Norway; MRC Integrative Epidemiology Unit (IEU), Population Health Sciences, Bristol Medical School, University of Bristol, Oakfield House, Oakfield Grove, Bristol, BS8 2BN, United Kingdom; MRC/CSO Social & Public Health Sciences Unit, University of Glasgow, Clarice Pears Building, 90 Byres Road, Glasgow, G12 8TB, United Kingdom; MRC Integrative Epidemiology Unit (IEU), Population Health Sciences, Bristol Medical School, University of Bristol, Oakfield House, Oakfield Grove, Bristol, BS8 2BN, United Kingdom; Population Health Sciences, Bristol Medical School, University of Bristol, Canynge Hall, 39 Whatley Road, Bristol, United Kingdom; MRC Integrative Epidemiology Unit (IEU), Population Health Sciences, Bristol Medical School, University of Bristol, Oakfield House, Oakfield Grove, Bristol, BS8 2BN, United Kingdom; School of Psychological Science, University of Bristol, 12a, Priory Road, Bristol, BS8 1TU, United Kingdom; MRC/CSO Social & Public Health Sciences Unit, University of Glasgow, Clarice Pears Building, 90 Byres Road, Glasgow, G12 8TB, United Kingdom

**Keywords:** alcohol, employment, socioeconomic position, Mendelian randomisation, deprivation

## Abstract

**Aims:**

Alcohol consumption is associated with socioeconomic disadvantage, but causality is unclear. We used Mendelian randomisation (MR) to estimate the effect of alcohol consumption on socioeconomic and employment-related outcomes.

**Methods:**

We conducted observational analyses and two-sample MR analyses using 230 775 working-age participants (aged 40+) of White British ethnicity/ancestry (54.5% male) from the UK Biobank.

**Outcomes:**

employment status (employed vs not; employed vs sickness/disability, unemployment, retirement or caring for home/family); weekly hours worked (among employed); deprivation (Townsend Deprivation Index); highest educational attainment; and household income.

**Exposures:**

alcohol consumption (units per week), hazardous alcohol consumption assessed by Alcohol Use Disorder Identification Test-C score and Alcohol use disorder (AUD).

**Results:**

MR analyses suggested that alcohol exposure increased the risk of living in a more deprived area. A unit per week increase in alcohol consumption increased deprivation by 0.31 deciles (95% CI: 0.10, 0.52). A unit increase in liability for AUD increased deprivation by 0.09 deciles (95% CI: 0.02, 0.16). Findings, and often effect directions, differed by sex. In men, increased alcohol consumption decreased household income and increased the risk of not being in paid employment and unemployment. In women, increased alcohol consumption decreased the risk of not being in paid employment, retirement, and caring for home/family. Effects were generally more detrimental for men than women. This is especially evident for not being in paid employment, household income, and deprivation.

**Conclusions:**

Alcohol consumption may increase the risk of living in deprived neighbourhoods. It may have deleterious effects on employment (including unemployment) and income, but these differ strongly by sex, largely affecting men.

## Teaser

**Short Summary:** Mendelian Randomisation analyses using a working age white British UK Biobank cohort indicated alcohol consumption may increase the risk of living in deprived neighbourhoods. These analyses also indicated deleterious effects of alcohol on employment (including unemployment) and on income, but these effects differ strongly by sex, largely affecting men.

## Introduction

Heavy alcohol consumption has negative impacts on the individual, family, and society at large. It is associated with anti-social behaviour, violence, accidents (Cherpitel, Bond et al. 2003) and a range of noncommunicable disorders (Parry, Patra et al. 2011). Alcohol use has been ranked ninth globally in risk factors for premature death and disability (Murray, Aravkin et al. 2020). Alcohol consumption and its effects are strongly patterned by sex, age, and income, with men more adversely affected. In 2019, the Disability Adjusted Life Years (DALY) rate was 1964 per 100 000 for males and 324 for females. Alcohol was the leading risk factor for DALYs for 15–49 year old males (Murray, Aravkin et al. 2020). The economic costs of alcohol are considerable. In six high and middle-income countries, total attributable costs (predominantly indirect costs of productivity losses) ranged between 1.3% and 3.3% of Gross Domestic Product (GDP) (Rehm, Mathers et al. 2009).

While associations between alcohol and health have been extensively studied (Rehm, Baliunas et al. 2010), less is known about the impact of alcohol on employment. Evidence is mixed, and the effect direction is unclear (Thompson and Pirmohamed 2021, Mangot-Sala, Smidt et al. 2022). High consumption is associated with higher absenteeism, higher presenteeism, lower productivity, and higher workplace stress (Buvik, Moan et al. 2018, Thorrisen, Bonsaksen et al. 2019). A high proportion of workplace accidents involve alcohol consumption. A US study found that 22% of occupational fatalities tested positive for alcohol or other substances (Ramirez, Bedford et al. 2013). Heavy alcohol use appears to reduce employability. In a fixed-effects panel study, binge drinking, dependence, and abuse were associated with experiencing trouble with co-workers, being laid off and remaining unemployed for more than one month (French, Maclean et al. 2011). Detrimental effects may extend to relatives through familial stress (Orford, Templeton et al. 2005).

As well as alcohol consumption potentially having causal effects on employment (i.e. health selection), the opposite has been proposed, namely that employment, unemployment, deprivation, and workplace stress influence alcohol consumption (social causation) (Hoffmann, Kröger et al. 2019). In addition, associations between consumption and employment may be driven by upstream factors (confounding). The likely presence of social causation and confounding makes the study of health selection challenging using standard methods.

Mendelian randomisation (MR) is an instrumental variable approach where the instruments are a set of genetic variants associated with an exposure (Hemani, Zheng et al. 2018). MR allows an estimation of the effect of the exposure on an outcome. In contrast to traditional regression, where statistical control of all confounders is necessary, MR relies on alternative assumptions (genetic variants being randomly inherited from parents to offspring and instruments only affecting the outcome through exposure). MR is a natural experiment where the instruments (randomised by Mendel’s law of segregation) index greater exposure over the life-course (Labrecque and Swanson 2019).

Evidence consistently indicates association between drinking and employment, but whether this reflects a causal relationship remains disputed (Mangot-Sala, Smidt et al. 2022). We used two-sample MR to investigate the health selection hypothesis that alcohol consumption affects employment and socioeconomic position (SEP). We estimated the causal effect of three alcohol measures (one reflecting consumption level, and two indications of hazardous alcohol use) on employment and related SEP measures. We tested whether effects differed by sex. We compared the MR estimates to multivariable-adjusted regression estimated associations of the exposures and outcomes.

## Methods

### Study population

The UK Biobank study collected data on half a million individuals aged between 40 and 69 from across mainland Britain (fieldwork conducted 2006–10) (Bycroft, Freeman et al. 2018). Participants were excluded from the current study if: (i) not of working age (i.e. above retirement age: 60 years for women, 65 years for men); (ii) self-reporting an ethnicity other than White UK; (iii) their genetically determined ancestry did not match their self-report; (iv) withdrawn from the study; (v) they were overly genetically related to another participant (one member of the pair retained); (vi) there were issues with their genetic data; or (vii) they were missing all of the investigated outcomes. Exclusions are detailed in Appendix 2.1 and in a STROBE flowchart (Fig. S1). The UK Biobank fields used in this study are detailed in Table S2.

### Exposures

#### Alcohol consumption

Alcohol consumption (units of alcohol per week) was calculated for each subject as the sum of the self-reported weekly intake of each alcoholic drink type multiplied by units of alcohol per drink measure. Weekly consumption was set to zero for participants who never drank or only drank on special occasions. The variable was winsorised to reduce values exceeding 140 to 140. Variable construction is detailed in Appendix 2.5.

#### Alcohol use disorder

Participant alcohol use disorder (AUD) status was based on self-report and hospital inpatient diagnosis. The subject was positive for AUD if they: (i) self-reported alcohol dependency or alcoholic liver disease/alcoholic cirrhosis (UKB field 20002), (ii) had a main or secondary ICD10 diagnosis of F10.1 or F10.2, or (iii) had a main or secondary ICD9 diagnosis of 303.9, 305.0, 305.1 or 305.9 [following Kranzler et al. (Kranzler, Zhou et al. 2019)].

#### Audit-C

Alcohol Use Disorder Identification Test (AUDIT) is a 10-item alcohol harm screening questionnaire. The first three questions form a hazardous alcohol use rating (AUDIT-C). AUDIT-C score was calculated per participant (0 = low risk, 12 = possible dependence).(Day, Copello et al. 2015) Approximately 80% of men scored four or less (for women three or less), indicating non-hazardous use.

### Genetic variants associated with alcohol exposures

Single nucleotide polymorphism (SNP) associations were obtained from Genome-wide Association Studies (GWASs) for: (i) alcohol consumption (drinks per week) (Liu, Jiang et al. 2019), (ii) AUD (Kranzler, Zhou et al. 2019) and (iii) AUDIT-C (Kranzler, Zhou et al. 2019). For AUD and AUDIT-C, SNP association estimates were obtained by meta-analysing GWAS results (obtained from the authors) from five ethnically homogenous cohorts from the Million Veteran Program (~274 000 subjects) (see Appendix 2.3). For each alcohol exposure, we identified SNPs robustly associated with the trait (*P*-value $\le$5x10^−8^). SNPs were excluded based on Hardy Weinberg Equilibrium [Family Wise Error Rate (FWER) < 1], Information Content (info score < 0.9), Minor Allele Frequency (MAF < 0.01) and being palindromic with a high MAF (MAF > 0.4). Clumping of the remaining SNPs (LD = 100 kb, R^2^ = 0.01), yielded a subset of independently associated SNPs, this constituted our instrument SNP set. The instrument sets (Table S3) contained: (i) 77 SNPs for alcohol consumption, (ii) 9 SNPs for AUD, and (iii) 14 SNPs for AUDIT-C. Three SNPs were shared across exposures, the rest were unique to an exposure. For details, see Appendix 2.3 and flowcharts (Fig. S2, S3 and S4).

### Outcomes

All outcomes were obtained from the baseline survey. Employment status was self-reported, the five most common categories being: (i) ‘in paid employment or self-employed’ (hereafter ‘in paid employment’), (ii) retired, (iii) sickness/disability (i.e. not working due to health), (iv) caring for home/family, and (v) unemployed. As the sample only included working-age people, anyone retired was in early retirement. Employment status was re-coded into: (i) a binary variable contrasting all other categories (hereafter referred to as non-employment) against employment and (ii) into four binary variables comparing each other category against being in paid employment. Where respondents endorsed multiple categories (<8% participants), ‘employed’ took priority for recoding. Other outcomes investigated were self-reported weekly hours in paid employment, household income, highest educational attainment and Townsend Deprivation Index (TDI).(Townsend, Phillimore et al. 1988) TDI measures area-based deprivation; higher TDI scores imply greater deprivation. To aid comparison to other studies, TDI was re-coded into deciles and treated as a continuous variable. Highest educational attainment was ordinally coded for UK academic qualifications from lowest to highest (i) None of the below, (ii) CSEs or equivalent, (iii) O levels/GCSEs or equivalent, (iv) A levels/AS levels or equivalent, (v) NVQ or HND or HNC or equivalent, (vi) Other professional qualifications, e.g. nursing, teaching, and (vii) College or university degree (see Appendix section 2.5 for definition of these acronyms and UK context of these categories). Annual gross household income was ordinally coded as: (i) Less than £18 000, (ii) £18 000 to £30 999, (iii) £31 000 to £51 999, (iv) £52 000 to £100 000, (v) Greater than £100 000.

### Statistical analyses

For each participant, for each alcohol exposure, we generated a polygenic score (PGS) calculated as the weighted sum of the number of risk alleles carried across instrument SNPs. The weights used were the SNP regression coefficients taken from the relevant exposure GWAS. To assess MR’s relevance assumption (that the instrument and exposure are associated), we regressed exposure on PGS. Exposure was regressed on PGS, adjusting for age, sex, assessment centre, and 40 genetic principal components (GPCs). The inclusion of GPCs (and assessment centres) as regression covariates is a standard way of correcting for population stratification, i.e. confounding between the regressor of interest (in this case PGS) and the outcome. Although some of the 40 GPCs available from UK Biobank may be redundant, their inclusion is unlikely to materially reduce power given the sample size (see Appendix 2.4).

We investigated the multivariable-adjusted association between each alcohol exposure and each outcome. Regression models were fitted adjusting for age, sex, assessment centre, and 40 GPCs. An additional covariate, number in household (values winsorised to 12), was included for household income, as this covariate strongly associated with household income. This was carried forward into subsequent MR analyses of household income.

Causal relationships were estimated via two-sample MR. The inputs for this were SNP-exposure associations (from prior studies) and SNP-outcome associations (from our individual-level analyses of UK Biobank). SNP-outcome associations were estimated by linear, logistic and ordinal regression of continuous, binary and ordinal outcomes on each SNP, adjusting for age, sex, assessment centre, and 40 GPCs.

The causal effect of exposures on outcomes was estimated using MR estimators provided in R package TwoSampleMR (Hemani, Zheng et al. 2018). The Rücker model selection framework (Bowden, Spiller et al. 2018) was used to identify the best fitting model from fixed and random effect versions of the Inverse Variance Weighted (IVW) and Egger methods. We reported multiple MR estimators, each making distinct assumptions about pleiotropy. IVW assumes no unbalanced pleiotropy. MR-Egger assumes SNP pleiotropic effect on outcome is independent of the SNP-exposure association. Median and modal estimators assume that the median and modal SNPs have no pleiotropic effects. Under directional pleiotropy, even if a valid instrument, the median/mode estimators will be biased (though not as severely as IVW). A penalised median method reduces such bias further (Hartwig, Davey Smith et al. 2017). The Robust Adjusted Profile Score method uses profile likelihood to model weak instruments under balanced pleiotropy and is robust to idiosyncratic pleiotropy in a small proportion of outliers (Zhao, Wang et al. 2020). For the AUD exposure, an underlying standard logistic distributed liability is assumed. The MR analyses estimate the effect of this liability.

*P*-values are reported without multiple testing correction. As per previous recommendations, we leave it to the reader to infer from our results (Althouse 2016, Amrhein, Greenland et al. 2019). We revisit this in the Discussion.

We assessed potential MR assumption violations. We tested for causal effect estimate heterogeneity and unbalanced pleiotropy. The No Measurement Error assumption for SNP-exposure associations was assessed using ${I}_{GX}^2$ (Bowden, Del Greco M. et al. 2016). To assess whether any single SNP was driving effect estimates, we conducted a single SNP MR analyses and leave one SNP out MR analyses. We repeated analyses with overly influential SNPs removed. These were SNPs whose Egger model fit Cook’s distance exceeded the F distribution (df1 = #SNPs, df2 = #SNPs – 2) median. One SNP rs1229984 (from the ADH1B gene), which strongly associates with alcohol consumption, was exempt from this. For details, see Appendix 2.10.

To investigate sex differences in alcohol exposure impact, analyses were repeated, stratified by sex. Wald tests were used to test for sex differences in effect size. We used the same instrument SNP set for the sex-stratified MR analyses as for the main MR analyses (see Appendix 2.11).

### Ethics

UK Biobank has approval from the North West Multi-Centre Research Ethics Committee (MREC) (UK Biobank Research Ethics Committee approval number 11/NW/0382) to obtain and disseminate data and samples from the participants (www.ukbiobank.ac.uk/ethics), and these ethical regulations cover the work in this study. All participants provided written informed consent.

## Results

### Demographics

Following exclusions (see the flow diagram Fig. S1), the analytical sample comprised 230 775 genetically unrelated working-age participants of White UK ethnic origin (Table 1). In our sample, men tended to be older, worked more hours, reported early retirement more frequently and were in paid work and caring for home and family less frequently.

**Table 1:** Study sample characteristics.

	Female	Male	Overall
Sample size, *n* (%)	104 991 (45.5)	125 784 (54.5)	230 775 (100)
Age [mean (SD)]	50.95 (5.55)	54.47 (7.04)	52.87 (6.64)
Alcohol units weekly (median [IQR])	7.0 [0.0, 15.5]	15.5 [5.5, 28.3]	11.0 [0.0, 22.5]
AUDIT-C score [mean (SD)]	4.29 (2.48)	5.59 (2.78)	4.96 (2.72)
Alcohol use disorder, *n* (%)	687 (.7)	2676 (2.1)	3363 (1.5)
Number of risk alleles [mean (SD)]			
For alcohol units weekly [mean (SD)]	87.57 (5.31)	87.54 (5.33)	87.55 (5.32)
For AUDIT-C score [mean (SD)]	19.80 (2.23)	19.77 (2.25)	19.79 (2.24)
For AUD [mean (SD)]	12.94 (1.79)	12.93 (1.80)	12.93 (1.79)
Employment category, *n* (%)			
In paid employment	83 339 (79.4)	90 252 (71.8)	173 591 (75.2)
Not in paid employment	19 691 (18.8)	33 961 (27.0)	53 652 (23.2)
Early retirement	6 789 (6.5)	24 079 (19.1)	30 868 (13.4)
Sick/disabled	4 949 (4.7)	6 800 (5.4)	11 749 (5.1)
Caring for home/family	7 521 (7.2)	1 069 (.8)	8 590 (3.7)
Unemployed	1 653 (1.6)	3 556 (2.8)	5 209 (2.3)
NA	19 999 (19.0)	31 976 (25.4)	51 975 (22.5)
TDI [mean (SD)]	−1.49 (2.93)	−1.48 (3.02)	−1.49 (2.98)
Work hours weekly [mean (SD)]	32.26 (11.47)	40.33 (1.97)	36.46 (11.92)
Household income (UK £, *n* (%))			
Less than 18 000	13 741 (13.1)	18 032 (14.3)	31 773 (13.8)
18 000 to 30 999	19 970 (19.0)	25 267 (20.1)	45 237 (19.6)
31 000 to 51 999	28 013 (26.7)	33 343 (26.5)	61 356 (26.6)
52 000 to 100 000	24 517 (23.4)	29 703 (23.6)	54 220 (23.5)
Greater than 100 000	6 298 (6.0)	7 996 (6.4)	14 294 (6.2)
NA	12 452 (11.9)	11 443 (9.1)	23 895 (10.4)
Highest educational attainment (%)			
None of the below	551 (.5)	867 (.7)	1418 (.6)
CSEs or equivalent	10 319 (9.8)	14 019 (11.1)	24 338 (1.5)
O levels/GCSEs or equivalent	2 982 (2.8)	3 296 (2.6)	6 278 (2.7)
A levels/AS levels or equivalent	20 667 (19.7)	21 901 (17.4)	42 568 (18.4)
NVQ or HND or HNC or equivalent	11 857 (11.3)	9 214 (7.3)	21 071 (9.1)
Other professional qualifications eg: nursing, teaching	15 591 (14.8)	23 572 (18.7)	39 163 (17.0)
College or university degree	33 575 (32.0)	35 780 (28.4)	69 355 (30.1)
NA	9 449 (9.0)	17 135 (13.6)	26 584 (11.5)

### Association of alcohol exposures with employment-related outcomes

**Figure 1:**
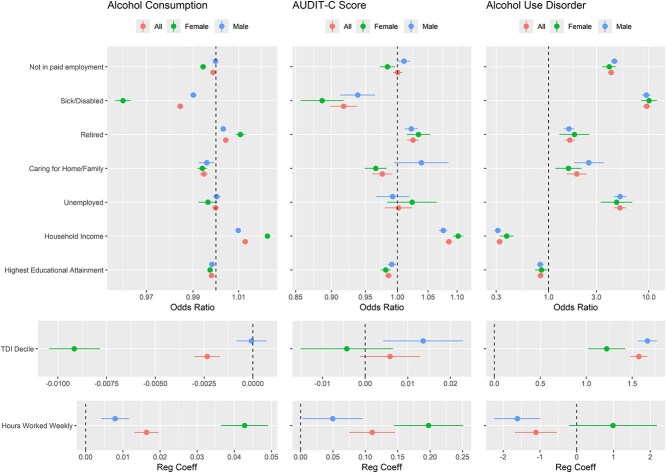
Forest plots of the association of alcohol exposures with outcomes.

Exposure associations (estimated through traditional regression) are presented in Fig. 1 (and in Table S5). AUD was strongly associated with increased risk of every measure of not being in paid employment, especially for being sick/disabled (OR = 9.8, 95% CI: 9.0, 1.7). It was also strongly associated with higher deprivation, lower hours worked, lower educational attainment and lower household income. Association patterns for the other two exposures were weaker, with some effects in the reverse direction.

### Polygenic score regressions

Results from the regression of the alcohol exposures on their PGSs are presented in Table S1. Traditionally weak instrument bias has been considered ignorable if F > 10. The F statistics for alcohol consumption, AUDIT-C and AUD were 1787, 25 and 31, respectively. This indicates the MR assumption of instrument-exposure association was met for each alcohol exposure. Correlations between the PGSs of the exposures were low, the highest being 0.34 (Table S4).

### Mendelian randomisation analyses

**Figure 2:**
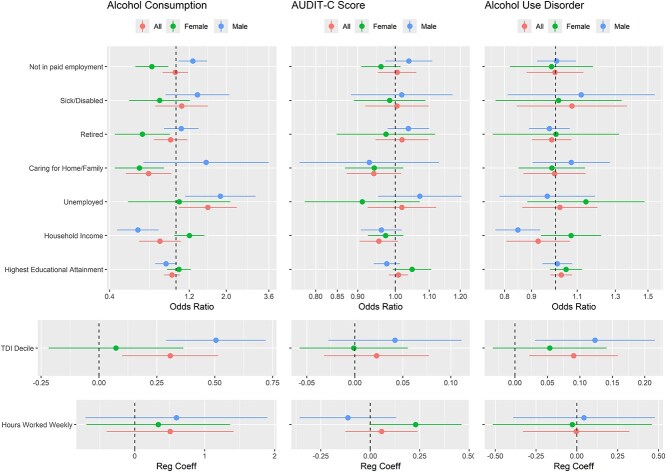
Forest plots of the MR estimates of the effect of alcohol exposures on outcomes.

Estimates from the best fitting model selected via the Rücker model selection framework are presented for the all-sexes effect in Fig. 2 and Table 2. The all-sexes effect estimates were that a unit increase in (i) alcohol consumption and (ii) AUD liability increased TDI decile by (i) 0.31, 95% CI: 0.10, 0.52 and (ii) 0.09, 95% CI: 0.02, 0.16, respectively. The estimate for AUDIT-C effect on TDI was in the same direction. MR analyses suggested alcohol consumption increased unemployment and decreased caring for home/family. MR analyses suggested household income decreased for all three alcohol exposures.

**Table 2:** MR estimates of the effect of alcohol exposures on outcomes.

Outcome	Method	Effect	Effect	Effect	Effect	
		Scale		95% CI	*P*-value	
Alcohol consumption						
Not in paid employment	IVW random effects	Odds ratio	0.99	(0.83, 1.18)	9.1E-01	
Retired	IVW random effects	Odds ratio	0.93	(0.74, 1.17)	5.3E-01	
Sick/disabled	IVW random effects	Odds ratio	1.08	(0.75, 1.56)	6.7E-01	
Caring for home/family	IVW fixed effects	Odds ratio	0.68	(0.50, 0.94)	1.8E-02	*
Unemployed	IVW fixed effects	Odds ratio	1.55	(1.04, 2.31)	3.1E-02	*
Highest educational attainment	IVW fixed effects	Odds ratio	0.95	(0.85, 1.06)	3.3E-01	
Household income	Egger random effects	Odds ratio	0.80	(0.60, 1.07)	1.4E-01	
TDI decile	IVW random effects	Linear	0.31	(0.10, 0.52)	3.7E-03	*
Hours worked weekly	IVW random effects	Linear	0.51	(−0.40, 1.42)	2.7E-01	
AUDIT-C Score						
Not in paid employment	IVW random effects	Odds ratio	1.01	(0.95, 1.06)	8.5E-01	
Retired	IVW random effects	Odds ratio	1.02	(0.95, 1.10)	6.2E-01	
Sick/disabled	IVW random effects	Odds ratio	1.00	(0.92, 1.10)	9.2E-01	
Caring for home/family	IVW fixed effects	Odds ratio	0.94	(0.87, 1.02)	1.2E-01	
Unemployed	IVW fixed effects	Odds ratio	1.02	(0.93, 1.12)	7.0E-01	
Highest educational attainment	IVW fixed effects	Odds ratio	1.01	(0.98, 1.04)	5.2E-01	
Household income	Egger random effects	Odds ratio	0.95	(0.90, 1.01)	1.2E-01	
TDI decile	IVW random effects	Linear	0.02	(−0.032, 0.077)	4.2E-01	
Hours worked weekly	IVW fixed effects	Linear	0.06	(−0.126, 0.239)	5.4E-01	
Alcohol use disorder						
Not in paid employment	IVW random effects	Odds ratio	1.00	(0.88, 1.13)	9.7E-01	
Retired	IVW fixed effects	Odds ratio	0.98	(0.90, 1.07)	7.1E-01	
Sick/disabled	IVW random effects	Odds ratio	1.07	(0.84, 1.37)	5.6E-01	
Caring for home/family	IVW fixed effects	Odds ratio	1.00	(0.87, 1.14)	9.5E-01	
Unemployed	IVW fixed effects	Odds ratio	1.02	(0.86, 1.20)	8.2E-01	
Highest educational attainment	IVW fixed effects	Odds ratio	1.03	(0.978, 1.08)	3.0E-01	
Household income	Egger random effects	Odds ratio	0.93	(0.81, 1.07)	3.2E-01	
TDI decile	IVW fixed effects	Linear	0.09	(0.02, 0.159)	9.4E-03	*
Hours worked weekly	IVW fixed effects	Linear	0.00	(−0.33, 0.32)	9.8E-01	

All effects were adjusted for age, sex, assessment centre, and genetic principal components. Effects on household income level were additionally adjusted for number in household winsorised to 12. We report the estimate of the MR method selected via the Rücker Model selection framework. Being in paid employment is the reference category for outcomes - Not in paid employment, Retired, Sick/Disabled, Caring for Home/Family and Unemployed.

Estimates from the best fitting model selected via the Rücker model selection framework are presented for the sex stratified MR analyses in Fig. 2 and Table 3. Sex differences in MR causal effects were evident. Effects were bigger for men than women for 12 of the 15 exposure non-employment outcome pairings. Effects more detrimental for men than women were also suggested for TDI decile, household income and highest educational attainment. In some cases, effect directions also differed across the sexes. For instance, alcohol consumption increased the risk of not being in paid employment in men OR = 1.26 (1.03, 1.54), while the opposite was seen for women OR = 0.72 (0.57, 0.90).

**Table 3:** Tests for sex differences in MR estimates of the effect of alcohol exposures on outcomes.

**Outcome**	**Rucker selected method**	**Male**	**Female**	**Effect Type**	**Effect Male**	**Effect CI 95% Male**	***P*-value Male**	**Effect Female**	**Effect CI 95% Female**	***P*-value Female**	**Effect delta**	**Sex diff**	
											**F-M**	**P-value**	
Alcohol consumption													
Not in paid employment	IVW fixed effects	Y	Y	Odds ratio	1.26	(1.03, 1.54)	2.2E-02	0.716	(0.57, 0.90)	4.1E-03	−0.544	0.00024	**
Retired	IVW fixed effects	Y	Y	Odds ratio	1.07	(0.87, 1.37)	5.5E-01	0.63	(0.43, 0.92)	1.7E-02	−0.446	0.02	*
Sick/disabled	IVW fixed effects		Y	Odds ratio	1.34	(0.93, 1.94)	1.1E-01	0.80	(0.53, 1.21)	2.9E-01	−0.546	0.066	
Sick/disabled	IVW random effects	Y		Odds ratio	1.34	(0.87, 2.09)	1.9E-01	0.80	(0.50, 1.28)	3.5E-01	−0.546	0.11	
Caring for home/family	IVW fixed effects	Y	Y	Odds ratio	1.51	(0.64, 3.6)	3.5E-01	0.60	(0.43, 0.85)	3.6E-03	−0.910	0.053	
Unemployed	IVW fixed effects	Y	Y	Odds ratio	1.85	(1.14, 3)	1.3E-02	1.05	(0.52, 2.12)	9.0E-01	−0.800	0.19	
Highest educational attainment	IVW fixed effects	Y	Y	Odds ratio	0.87	(0.75, 1.01)	7.1E-02	1.04	(0.89, 1.22)	6.3E-01	0.170	0.11	
Household income	Egger fixed effects	Y		Odds ratio	0.59	(0.44, 0.786)	5.4E-04	1.16	(0.84, 1.6)	3.8E-01	0.568	0.0022	*
Household income	IVW random effects		Y	Odds ratio	0.89	(0.74, 1.06)	1.9E-01	1.2	(0.98, 1.48)	8.2E-02	0.315	0.03	*
TDI decile	IVW fixed effects	Y		Linear	0.51	(0.29, 0.72)	4.0E-06	0.074	(−0.161, 0.308)	5.4E-01	−0.432	0.0078	*
TDI decile	IVW random effects		Y	Linear	0.51	(0.27, 0.74)	2.4E-05	0.074	(−0.22, 0.37)	6.2E-01	−0.432	0.024	*
Hours worked weekly	IVW fixed effects		Y	Linear	0.60	(−0.41, 1.61)	2.5E-01	0.34	(−0.69, 1.37)	5.2E-01	−0.259	0.72	
Hours worked weekly	IVW random effects	Y		Linear	0.60	(−0.71, 1.9)	3.7E-01	0.34	(−0.69, 1.37)	5.2E-01	−0.259	0.76	
AUDIT-C score													
Not in paid employment	IVW fixed effects		Y	Odds ratio	1.04	(0.99, 1.09)	1.2E-01	0.91	(0.79, 1.04)	1.7E-01	−0.130	0.068	
Not in paid employment	IVW random effects	Y		Odds ratio	1.04	(0.97, 1.11)	2.6E-01	0.91	(0.77, 1.08)	2.8E-01	−0.130	0.16	
Retired	IVW fixed effects	Y		Odds ratio	0.99	(0.86, 1.14)	8.9E-01	0.97	(0.89, 1.07)	5.8E-01	−0.016	0.85	
Retired	IVW random effects		Y	Odds ratio	0.99	(0.84, 1.17)	9.0E-01	0.97	(0.85, 1.12)	7.1E-01	−0.016	0.89	
Sick/disabled	IVW fixed effects		Y	Odds ratio	1.06	(0.86, 1.33)	5.7E-01	0.88	(0.69, 1.13)	3.3E-01	−0.181	0.27	
Sick/disabled	IVW random effects	Y		Odds ratio	1.06	(0.74, 1.54)	7.4E-01	0.88	(0.67, 1.17)	3.9E-01	−0.181	0.43	
Caring for home/family	IVW fixed effects	Y	Y	Odds ratio	1.12	(0.69, 1.82)	6.6E-01	0.95	(0.78, 1.16)	5.9E-01	−0.170	0.54	
Unemployed	IVW fixed effects	Y	Y	Odds ratio	1.03	(0.77, 1.37)	8.6E-01	1.06	(0.71, 1.6)	7.7E-01	0.036	0.89	
Highest educational attainment	IVW fixed effects	Y		Odds ratio	0.98	(0.94, 1.01)	2.0E-01	1.11	(1.01, 1.22)	3.6E-02	0.133	0.016	*
Highest educational attainment	IVW random effects		Y	Odds ratio	0.98	(0.94, 1.01)	2.1E-01	1.11	(0.97, 1.27)	1.3E-01	0.133	0.071	
Household income	Egger random effects	Y		Odds ratio	0.84	(0.68, 1.05)	1.6E-01	1.09	(0.88, 1.35)	4.6E-01	0.245	0.11	
Household income	IVW fixed effects		Y	Odds ratio	0.96	(0.88, 1.05)	3.4E-01	1.15	(1.04, 1.27)	5.3E-03	0.195	0.0065	*
TDI decile	IVW fixed effects	Y	Y	Linear	0.14	(0.008, 0.261)	3.7E-02	0.12	(−0.01, 0.25)	7.7E-02	−0.015	0.88	
Hours worked weekly	IVW fixed effects	Y	Y	Linear	−0.22	(−.81, 0.38)	4.7E-01	−0.24	(−0.76, 0.28)	3.7E-01	−0.022	0.96	
Alcohol use disorder													
Not in paid employment	IVW fixed effects	Y		Odds ratio	1	(0.92, 1.09)	9.2E-01	0.98	(0.89, 1.08)	7.3E-01	−0.022	0.74	
Not in paid employment	IVW random effects		Y	Odds ratio	1	(0.89, 1.13)	9.4E-01	0.98	(0.82, 1.18)	8.5E-01	−0.022	0.84	
Retired	IVW fixed effects	Y		Odds ratio	0.97	(0.89, 1.06)	5.6E-01	1.00	(0.85, 1.18)	9.8E-01	0.028	0.76	
Retired	IVW random effects		Y	Odds ratio	0.97	(0.89, 1.06)	5.6E-01	1.00	(0.76, 1.32)	9.9E-01	0.028	0.85	
Sick/disabled	IVW random effects	Y	Y	Odds ratio	1.12	(0.81, 1.55)	4.9E-01	1.01	(0.77, 1.34)	9.2E-01	−0.106	0.65	
Caring for home/family	IVW fixed effects	Y	Y	Odds ratio	1.07	(0.90, 1.27)	4.2E-01	0.98	(0.85, 1.14)	8.3E-01	−0.087	0.46	
Unemployed	IVW fixed effects	Y	Y	Odds ratio	0.97	(0.78, 1.19)	7.4E-01	1.14	(0.88, 1.48)	3.1E-01	0.178	0.32	
Highest educational attainment	IVW fixed effects	Y	Y	Odds ratio	1.01	(0.95, 1.08)	8.1E-01	1.05	(0.98, 1.12)	2.0E-01	0.039	0.44	
Household income	Egger fixed effects	Y		Odds ratio	0.85	(0.77, 0.94)	1.3E-02	1.04	(0.93, 1.16)	5.2E-01	0.191	0.0074	*
Household income	IVW random effects		Y	Odds ratio	0.98	(0.87, 1.11)	7.6E-01	1.07	(0.94, 1.22)	3.1E-01	0.090	0.34	
TDI decile	IVW fixed effects	Y	Y	Linear	0.12	(0.031, 0.22)	8.8E-03	0.054	(−0.03, 0.14)	2.3E-01	−0.070	0.28	
Hours worked weekly	IVW fixed effects	Y	Y	Linear	0.044	(−0.39, 0.48)	8.4E-01	−0.028	(−0.52, 0.46)	9.1E-01	−0.072	0.83	

All effects were adjusted for age, assessment centre, and genetic principal components. Effects on household income level were additionally adjusted for number in household winsorised to 12. We report the effect estimates for male and female only datasets for the MR method selected via the Rücker Model selection framework. Being in paid employment is the reference category for outcomes - Not in paid employment, Retired, Sick/Disabled, Caring for Home/Family and Unemployed.

Phenotypic associations are plotted on the MR effect forest plots for alcohol consumption in Figs. S5, S6 and S7, and for AUDIT-C in Figs. S8, S9 and S10 (for all sexes, males and females respectively, method = ‘assoc. with exposure’). The alcohol consumption phenotypic associations have regression coefficients close to 0 or odds ratios close to 1. These are generally consistent with the MR estimates due to the wide confidence intervals of the latter. However, there are exceptions; notably MR effect estimates were much higher for deprivation. For AUDIT-C, phenotypic association and MR effect estimates were not matched for sick/disabled and household income.

### Sensitivity analyses

The robustness of the causal effect estimates was investigated. In most cases, the estimates of the various MR methods employed agreed, and the Rücker model selection framework MR estimates were representative. Forest plots showing the complete set of MR estimates for the effect of alcohol consumption on the examined outcomes are presented in Fig. S5 for the all-sexes dataset, in Fig. S6 for the male dataset and in Fig. S7 for the female dataset. Similar plots are presented for AUDIT-C in Figs. S8, S9 and S10, and for AUD in Figs. S11, S12 and S13.

Tests suggested effect size heterogeneity across SNPs for all three exposures for the not in paid employment, sick/disabled and household income outcomes and for several other exposure-outcome pairs. The Rücker model selection framework always selected a random-effect model for these cases. Tests produced evidence of unbalanced pleiotropy in only a few cases. The Rücker model selection framework selected an Egger model for all but one of these. For each exposure and outcome, ${I}_{GX}^2$ was above 0.98, above the 0.9 threshold indicative of NOME assumption violation (Bowden, Del Greco M. et al. 2016) (Appendix 3.3).

There was a 33% overlap between our analytic sample and the dataset used for the Liu et al. drinks per week GWAS from which we constructed our alcohol consumption instrument set. Bias arising due to participant overlap in two sample MR analysis has been investigated theoretically and using simulation studies for continuous and discrete outcomes (Burgess, Davies et al. 2016). Burgess et al. provide an R Shiny app for estimating the expected bias due to sample overlap. We used that app to estimate the expected bias in our alcohol consumption MR analysis and found it was negligible. For further details see Appendix 3.4.

## Discussion

We investigated the causal effects of alcohol consumption (weekly units), two measures of hazardous alcohol use (AUDIT-C and AUD) on employment-related outcomes in the UK Biobank study using two sample MR. We also conducted multivariable-adjusted regression analyses for comparison. MR analyses indicated that alcohol increased the likelihood of living in a deprived area. Sex differences were evident. For many exposure-outcome pairings, effect directions differed between men and women, and were generally more detrimental for men than women. Association estimates (obtained through traditional regression) were largely consistent with MR effect estimates for alcohol consumption and AUDIT-C. However, they were mismatched for the sick/disabled, TDI decile and household income outcomes. The mismatch may result from confounding of these phenotypic association, reverse causation or MR assumption violation.

The correlation between alcohol exposure PGSs was low. So similarity of MR estimates across alcohol exposures is unlikely due to instrument similarity, although the instruments could index the same or similar underlying traits.

How alcohol impacts employment is complex (Devaux 2015), and effects may be non-linearly dose dependent. Moderate consumption has been associated with beneficial employment outcomes in a range of countries (Hamilton and Hamilton 1997, Zarkin, French et al. 1998, MacDonald and Shields 2001, Barrett 2002, Lee 2003, Dilmaghani 2022), consistent with our findings for women, while detrimental effects have been seen for heavy drinkers (MacDonald and Shields 2004, Böckerman, Hyytinen et al. 2017), consistent with our findings for men. For instance, in the Danish National Health Survey, high alcohol consumption and problem drinking increased the probability of leaving employment and decreased the chances of gaining employment in those out of work at baseline (Jørgensen, Pedersen et al. 2019). A twins study examined the effect of alcohol consumption on future employment outcomes over a 20-year window while accounting for shared genetics and environment and for standard covariates. It found that former and heavy drinkers earned 20% less than moderate drinkers and were employed 8% less of the time, but results did not differ by sex as ours did (Böckerman, Hyytinen et al. 2017). Detrimental effects have also been seen for very low/zero alcohol consumption, e.g. (Dilmaghani 2022), with evidence that these can be attributed to a ‘sick quitter’ effect (Kaila-Kangas, Kivekäs et al. 2015).

There is evidence for (Erol and Karpyak 2015, Dilmaghani 2022) and against (Böckerman, Hyytinen et al. 2017) sex differences in the effect of alcohol on employment. The sex differences in MR effect we found were striking. In particular, MR estimates indicated that lower alcohol consumption increased the risk of not being in paid employment for women, whereas the opposite was the case for men. This could be due to sex differences in drinking levels, with women having lower drinking levels than men (see Fig. S18). It may be that the above mentioned role for alcohol is not directly due to sex but reflects a non-linear relationship between drinking level and employment. This would align with literature suggesting moderate drinking associates with increased employment. It could also reflect sex differences in drinking behaviour, e.g. rates of binge drinking.

Alcohol consumption is strongly socially patterned (Collins 2016, Keyes, Platt et al. 2021). White, single, young, male, and childless people in the UK drink more alcohol. The interplay between consumption, occupational group and gender may partly explain the sex differences we found. A US study reported differences across job types in binge drinking rates (Shockey and Esser 2020). A similar UK Biobank study found heavy drinker prevalence varied greatly with job type and drinking patterns across occupations differed with sex (Thompson and Pirmohamed 2021).

Our findings related to deprivation suggest that higher alcohol consumption caused downward social selection. This may amplify the harmful effects of alcohol, as lower SEP strata have been observed to suffer disproportionately from the harmful effects of alcohol despite consuming less than higher SEP strata, a phenomenon known as the alcohol harm paradox.(Boyd, Hayes et al. 2023).

Our findings should be considered in the light of potential limitations. We restricted our sample to those of working age of White British ethnicity because MR lacked the power to investigate effects in other ethnicities. Our results may not generalise well to other ethnicities, younger (<40) people, or those beyond working age. The UK Biobank cohort participants tend to be healthier, wealthier, and better educated than the general UK population, consistent with a ‘healthy volunteer’ effect (Fry, Littlejohns et al. 2017). Thus, this sample may be insulated from strong effects of alcohol on employment. However, UK Biobank risk factor-trait associations are similar to those in the UK population (Batty, Gale et al. 2020). Self-selection may have induced collider bias in our findings; however, this is unlikely to be strong (Smith, Lawlor et al. 2007). MR, although robust against many unobserved confounders, may be susceptible to confounding by population structure. Adjusting for assessment centre plus GPCs minimised this. A similar set of covariates adequately accounted for confounding by birth location for some but not all traits (Haworth, Mitchell et al. 2019). Another possible confounding source is dynastic effects, i.e. violation of the MR exclusion assumption via the existence of a path from the participants’ parents’ genes to the participants’ outcome (Brumpton, Sanderson et al. 2020). Alcohol consumption probably has historically played a role in both finding partners and conception. Sibling MR analysis could investigate this, but would be underpowered (only 20 000 sibling pairs in UK Biobank). Thus, residual confounding [independence assumption (Davies, Smith et al. 2013) violation] remains possible. Alcohol-employment relationships may have changed during participants’ lifetimes. Our estimates average these relationships and may reflect causal processes occurring across the life course before participants responded to the survey. Two of our alcohol exposures were self-reported; there is a tendency for people to under-report (De Leeuw, Hox et al. 2012). However, a strength of instrumental variable analysis is that it can overcome non-differential measurement error on the exposure. We have reported *P*-values without multiple testing correction since our interpretation throughout is primarily shaped by effect estimates and 95% confidence intervals, rather than the *P*-values. However, we acknowledge that opinions differ regarding the utility of *P*-values and we therefore report them to assist readers in making their own interpretation based on *P*-values if they so choose.

## Conclusions

Alcohol consumption is an important socialising behaviour in many societies. In the UK, alcohol consumption though declining remains high, 57% reported drinking alcohol in the previous week, and 18% of men reported drinking more than 8 units on their heaviest drinking day that week (13% of women exceeded 6 units). (Office_for_National_Statistics_(UK) 2018).

Our MR analyses support alcohol consumption having considerable adverse economic impact by increasing deprivation. It also indicated deleterious effects of alcohol on employment. As drinking behaviour is modifiable there is potential for substantial reduction in alcohol’s health, social, and economic costs through public health initiatives.

Our effect sizes differed strongly by sex and were more detrimental for men. Caution should be exercised when generalising these results across societies and time. Future research should address the social causation hypothesis and the sex difference in effects, including the intriguing finding that alcohol might increase female paid employment. It should also attempt to quantify the societal costs of alcohol consumption. Dissecting the relationships between alcohol and its social consequences requires application of multiple study designs.

## Supplementary Material

Alcohol_Employment_MR_appendix_2jun25_agaf038

Campbell_Green_Davies_et_al_2025_agaf038

## Data Availability

The UK Biobank dataset, which provides individual level data, is available upon application (www.ukbiobank.ac.uk). GWAS results for AUD and AUDIT-C are available on request from the Database of Genotypes and Phenotypes (dbGaP) (www.ncbi.nlm.nih.gov/gap, dbGaP Study Accession: phs001672.v12.p1). GWAS results for alcohol consumption are available online (https://genome.psych.umn.edu/index.php/GSCAN). Scripts used to conduct our analyses are available upon reasonable request from the authors.
