# Supplementary material for: Effects of alcohol consumption on employment and social outcomes: a Mendelian randomisation study
Source: Alcohol Alcohol. 2025 Jul 18;60(5):agaf038. doi: 10.1093/alcalc/agaf038 (PMC12271571; doi:10.1093/alcalc/agaf038)
Supplement: Alcohol_Employment_MR_appendix_2jun25_agaf038 [file alcohol_employment_mr_appendix_2jun25_agaf038.docx]

Appendix: Effects of alcohol consumption on employment and social outcomes: A Mendelian randomisation study

Contents

[1 Purpose 2](#_Toc195648592)

[2 Methods 2](#_Toc195648593)

[2.1 UK Biobank fields 2](#_Toc195648594)

[2.2 Study population 3](#_Toc195648595)

[2.3 Instrument SNPs 3](#_Toc195648596)

[2.4 Polygenic score 4](#_Toc195648597)

[2.5 Outcomes 4](#_Toc195648598)

[2.6 Exposures 4](#_Toc195648599)

[2.7 Regression of Outcomes on Exposure 5](#_Toc195648600)

[2.8 Regression of Exposure on Polygenic score 5](#_Toc195648601)

[2.9 SNP Associations with Outcomes 5](#_Toc195648602)

[2.10 MR Analyses 6](#_Toc195648603)

[2.11 Investigation of interaction with sex 6](#_Toc195648604)

[2.12 Outlier SNP removal 7](#_Toc195648605)

[3 Results 7](#_Toc195648606)

[3.1 Regression of Exposure on Polygenic score 7](#_Toc195648607)

[3.2 MR Analyses 7](#_Toc195648608)

[3.3 MR Robustness analyses 8](#_Toc195648609)

[3.3.1 Specimen Results 8](#_Toc195648610)

[3.4 Sample Overlap 9](#_Toc195648611)

[4 Discussion 9](#_Toc195648612)

[5 Tables 10](#_Toc195648613)

[5.1 Table S1 10](#_Toc195648614)

[5.2 Table S2 11](#_Toc195648615)

[5.3 Table S3 12](#_Toc195648616)

[5.4 Table S4 14](#_Toc195648617)

[5.5 Table S5 15](#_Toc195648618)

[6 Figures 16](#_Toc195648619)

[6.1 Figure S1 16](#_Toc195648620)

[6.2 Flowcharts of instrument SNP screening 16](#_Toc195648621)

[6.2.1 Figure S2 16](#_Toc195648622)

[6.2.2 Figure S3 18](#_Toc195648623)

[6.2.3 Figure S4 18](#_Toc195648624)

[6.3 MR effect estimates for Alcohol Consumption 19](#_Toc195648625)

[6.3.1 Figure S5 19](#_Toc195648626)

[6.3.2 Figure S6 20](#_Toc195648627)

[6.3.3 Figure S7 21](#_Toc195648628)

[6.4 MR effect estimates for AUDIT-C 22](#_Toc195648629)

[6.4.1 Figure S8 22](#_Toc195648630)

[6.4.2 Figure S9 23](#_Toc195648631)

[6.4.3 Figure S10 24](#_Toc195648632)

[6.5 MR effect estimates for AUD 25](#_Toc195648633)

[6.5.1 Figure S11 25](#_Toc195648634)

[6.5.2 Figure S12 26](#_Toc195648635)

[6.5.3 Figure S13 27](#_Toc195648636)

[6.6 Plots for specimen exposure-outcome pairing 28](#_Toc195648637)

[6.6.1 Figure S15 29](#_Toc195648638)

[6.6.2 Figure S16 30](#_Toc195648639)

[6.6.3 Figure S17 30](#_Toc195648640)

[6.6.4 Figure S18 31](#_Toc195648641)

[6.7 Sample overlap screenshot 31](#_Toc195648642)

[7 References 32](#_Toc195648643)

# Purpose

This is the appendix for the paper

- Effects of alcohol consumption on employment and social outcomes: A Mendelian randomisation study

# Methods

## UK Biobank fields

A list of the important UK Biobank fields used in this study is given in Table S2.

## Study population

UK Biobank participants were excluded for the following reasons

- Not self-reported White UK ethnicity with matching genetics (UK Biobank field 22006)
- Over retirement age at time of assessment (60 years female, 65 years male)
- Participant did not have a value for any of the outcomes of interest.
- mismatch between self-declared sex and genetically predicted sex (UK Biobank field 22001)
- abnormal number of X and Y chromosomes (UK biobank field 22019)
- Low genotyping rate (<98.5%), which is indicative of low-quality DNA (UK Biobank field 22005)
- Participant DNA an outlier for heterozygosity or missing rate, which implies poor quality of genotyping (UK Biobank field 22027)
- Participant withdrawn from the study (before 22nd February 2022)
- Over-relatedness: if a pair surviving the above exclusions were too genetically related (kinship > 0.042, e.g. closer than 2^nd^ cousins), then one of the pair was dropped. The participant with the most relations was preferentially dropped. If both participants had the same number of relations, then the subject dropped was randomly chosen.

A STROBE flowchart (Figure S1) reports the numbers lost through these exclusion criteria. The retained sample (230,775 participants) was the basis of all further analysis.

## Instrument SNPs

Single Nucleotide Polymorphism (SNP) sets were derived from Genome-wide Association Study (GWAS) results for; (i) alcohol consumption, (ii) Alcohol Use Disorder (AUD) and (iii) AUDIT-C.

For AUD and AUDIT-C, the GWAS results as published by authors Kranzler et al. did not allowed us to derive our instrument SNP sets. For these traits, SNP association estimates were obtained by meta-analysing (using METAL (Willer et al., 2010)) GWAS results (obtained from the authors) from five ethnically homogenous cohorts (the largest being Caucasian) from the Million Veteran Program (~274,000 subjects). The SNP alcohol exposure associations were obtained by meta-analysing association results for up to 12,545,884 SNPs from five population groups consisting of

- EA – European American (n=191,840)
- AA – African American (n=53,798)
- LA - Hispanic and Latino American (n=13,456)
- EAA – East Asian American (n=1,304)
- SAA - South Asian American (n=181)

For each alcohol exposure, we identified SNPs robustly associated with the trait (p-value $\leq$ 5x10^-8^). The following SNP exclusion criteria were then applied

- Hardy Weinberg Equilibrium (HWE): We used Family Wide Error Rate (FWER) = 1 Bonferroni correction to screen for SNPs not in HWE. In other words, we would expect one perfectly good SNP to be rejected according to this criterion.
- Low information content: Information score ranges from 0 to 1 and reflects the quality of imputation. SNP dropped if < 0.9
- Low Minor Allele Frequency (MAF): SNP dropped if MAF < 0.01
- Palindromic and high MAF: Palindromic SNP dropped if MAF > 0.4

The last criterion arises from the difficulty of reconciling the strands palindromic SNPs were called on, in the SNP-exposure and SNP-outcome datasets. Linkage Disequilibrium (LD) clumping of remaining SNPs was used to identify sets of mutually independent SNPs. This was done using the ieugwasr R package ld_clump function with default clumping window size of 10 MBases and a cut-off of R2 < 0.01. The retained SNPs (listed in Table S3) constituted our instrument SNP sets used for subsequent Mendelian Randomisation analyses. Flow charts for the generation of instrument SNP sets are shown in Figure S2, Figure S3 and Figure S4. Associations were harmonised (using the TwoSampleMR R package) to ensure consistent directions of association across all SNPs of a SNP set.

## Polygenic score

To measure the strength of our genetic instruments and to validate the instrument-exposure association assumption of the Mendelian randomisation method, we generated a polygenic score for each exposure for each participant. This was constructed as the weighted sum of the number of risk alleles carried across instrument SNPs. The weights used were the SNP regression coefficients taken from the relevant exposure GWAS. Scores were generated using the score function of PLINK 1.9.(Chang et al., 2015) We used the default setting for missing genotypes, namely imputation of the missing genotype with its expectation, (i.e. twice the SNP’s risk allele frequency).

## Outcomes

**Highest educational attainment**

Highest educational attainment was an ordinal coding for UK academic qualifications from lowest to highest (i) None of the below, (ii) CSEs or equivalent, (iii) O levels/GCSEs or equivalent, (iv) A levels/AS levels or equivalent, (v) NVQ or HND or HNC or equivalent, (vi) Other professional qualifications e.g. nursing, teaching, and (vii) College or University degree.

Further information on these categories follows

1. CSEs or equivalent, are school leaving qualifications
   1. https://en.wikipedia.org/wiki/Certificate_of_Secondary_Education
2. O levels/GCSEs or equivalent, are school leaving qualifications
   1. <https://en.wikipedia.org/wiki/GCE_Ordinary_Level_(United_Kingdom)>
   2. https://en.wikipedia.org/wiki/General_Certificate_of_Secondary_Education
3. A levels/AS levels or equivalent, are school leaving qualifications
   1. https://en.wikipedia.org/wiki/A-Level
4. NVQ or HND or HNC or equivalent, are higher education/further education qualifications
   1. <https://en.wikipedia.org/wiki/National_Vocational_Qualification>
   2. <https://en.wikipedia.org/wiki/Higher_National_Diploma>
   3. https://en.wikipedia.org/wiki/Higher_National_Certificate
5. Other professional qualifications e.g. nursing, teaching, are vocational qualifications
6. College or University degree

## Exposures

The main paper Methods, Exposure section covers this adequately.

## Regression of Outcomes on Exposure

We regressed the outcomes on the exposure using the following regression equation.

$$Outcome=Exposure+sex+age+assessmentCentre+GPC1+GPC2+\ldots+GPC40$$

Where

- Exposure = one of the three alcohol exposures
- Sex – coded as male =1, female =2
- AssessmentCentre – the UK Biobank assessment centre. These were represented in the regression by a set of dichotomous dummy variables.
- GPC1 … GPC40 – genetic principal components

For ordinal, binary and continuous outcomes we used ordinal, logistic and linear regression respectively. The logistic and linear regressions were implemented using PLINK.(Chang et al., 2015) The ordinal regressions were implemented using the *polr* function of the MASS R package. For some categories of non-continuous outcomes, the count was low or even zero for some assessment centres. This would cause the regression to fail or return inaccurate estimates for these assessment centres. To overcome this, assessment centres were repeatedly merged until the minimum such count exceeded 50 (in some cases 20) prior to performing regressions. As a rule of thumb it is recommended there is a count of 10 or more of any particular outcome for any given regressor in regressions (e.g. logistic) with categorical outcomes. (Peduzzi et al., 1996, Ranganathan et al.)

## Regression of Exposure on Polygenic score

We regressed each exposure on the polygenic score for the exposure, using the regression equation

$$Exposure=PolygenicScore+sex+age+assessmentCentre+GPC1+GPC2+\ldots+GPC40$$

We conducted ANOVA and calculated the F statistic and adjusted partial R squared for each regressor.

## SNP Associations with Outcomes

We regressed each outcome on each SNP of each instrument set using the UK Biobank dataset. The regression equation was

$$Outcome=riskAlleleCount+sex+age+assessmentCentre+GPC1+GPC2+\ldots+GPC40$$

Where

- $riskAlleleCount$ – the risk allele count (0, 1 or 2) for the SNP in question

We used ordinal, logistic and linear regression for ordinal, dichotomous and continuous outcomes respectively. The logistic and linear regressions were implemented using PLINK.(Chang et al., 2015) The ordinal regressions were implemented using the *polr* function of the *MASS* R package. For some categories of non-continuous outcomes, the count was low or even zero for some assessment centres. This would cause the regression to fail or return inaccurate estimates for these assessment centres. To overcome this, assessment centres were repeatedly merged until the minimum such count exceeded 50 (in some cases 20) prior to performing regressions. As a rule of thumb it is recommended there is a count of 10 or more of any particular outcome for any given regressor in regressions (e.g. logistic) with categorical outcomes. (Peduzzi et al., 1996, Ranganathan et al.)

## MR Analyses

We conducted a two-sample MR analysis using the SNP-exposure associations obtained from the respective GWAS and the SNP-outcome associations from the study sample. We estimated causal effects using the wide range of MR causal effect estimation methods available in the TwoSampleMR R package.(Hemani et al., 2018) We also tried using methods from the RadialMR R package. The Radial MR-Egger method is a regression directly on a Galbraith radial plot. However, for some outcomes the Radial MR-Egger fit as displayed on the (RadialMR generated) plot was clearly wrong. Also, for several outcomes the Radial MR-Egger estimate was very different from all the other estimates. In addition, we could not update the package from its GitHub repository. For these reasons we excluded RadialMR package estimators from our reporting. We used the Rücker model selection framework to identify the best fitting model from fixed and random effect versions of the IVW and Egger methods. The less parsimonious model was rejected at a p-value of > 0.05. We reported this as the representative estimate for each exposure outcome pairing.

Sensitivity analyses were conducted on the MR analyses results. Heterogeneity amongst the causal effect estimates from the instrument SNPs was assessed with Cochran’s Q (assuming balanced pleiotropy) and Rücker’s Q (assuming unbalanced pleiotropy). Using Cochran’s Q and Rücker’s Q as inputs, we applied the Rücker model selection framework to identify the best fitting model between fixed effect and random effect versions of the IVW and Egger methods.(Rucker et al., 2011) We followed Bowden et al. in using 0.05 as a significance threshold for detecting pleiotropy for model selection purposes (see Box 3 of Bowden et al. 2018).(Bowden et al., 2018) We conducted unbalanced pleiotropy tests (implemented via TwoSampleMR::mr_pleiotropy_test). We calculated $I_{GX}^{2}$, a measure of the degree of violation of the No Measurement Error (NOME) assumption for SNP-exposure associations (implemented via TwoSampleMR::Isq). We also investigated whether individual SNPs differed in their effect. We did this by conducting Single SNP MR analyses and Leave One SNP Out MR analyses.

## Investigation of interaction with sex

MR analyses were repeated in male only and female only subsets of the study sample for all outcomes. We tested whether there was evidence for difference in causal effects between the sexes for each exposure outcome pairing. We did this using Fisher’s z-score method (a Wald test), comparing the test statistic *z* to a standard normal distribution, by applying:

$$z=\frac{b_{Male}-b_{Female}}{\sqrt{{se}_{Male}^{2}+ {se}_{Female}^{2}}}$$

Where

- $b$ = causal effect estimate
  - for dichotomous outcomes = log odds ratio
  - for continuous outcomes = beta (regression coefficient)
- $se$ = standard error of $b$
- subscript denotes stratum

The Rücker model selection framework was used to select an estimate to use for each exposure outcome sex combination. We used the same instrument SNP set for the sex stratified MR analyses as for the main MR analyses. There was negligible difference between the sexes in the distribution of the polygenic score (PGS) (see Table 1). Exposure SNP association estimates were from GWASs performed on discovery datasets, so these associations were likely regressed towards the mean in the UK Biobank dataset. This could bias evidence for sex difference in causal effect if the degree of regression towards the mean differed between sexes. However, there is no reason to expect this given similar male to female ratios in the GWAS and UK Biobank datasets.

## Outlier SNP removal

As a further sensitivity analysis, we attempted to identify and exclude from the instrument SNP set, SNPs that were overly influential on MR analyses results. SNP influence was measured using the SNP’s Cook’s distance from the Egger regression fit.  We used the median point of the F distribution F( p=0.5, 2, #snps-2) as a threshold for outlier detection. One SNP in the ADH1B gene which very strongly associates with alcohol measures was retained regardless of Cook’s distance. For most exposure-outcome pairs, no outliers were detected; for the remainder, only one outlier was detected.

# Results

Complete result files are available upon request from the authors as a zip file.

## Regression of Exposure on Polygenic score

The regressions of each alcohol exposure on polygenic score are presented in Table S1. The F statistics indicate the instrument-exposure association MR assumption was met for each alcohol exposure. Table S1 N column reflects the number of non-missing values in our analytical sample for each alcohol exposure. The value for AUDIT-C is only a proportion our analytical sample because the AUDIT-C exposure was constructed from items in an online questionnaire that was completed by 157,366 participants. (Davis et al., 2020)

## MR Analyses

Two sample MR causal effect estimates for all the outcomes are reported in files

- representativeMrEstimate_inc.xlsx – the Rucker model selected framework estimates
- mrEstimate_inc.xlsx – for all MR methods

The estimates for all MR methods are also presented in forest plots in

- Figure S5, Figure S6 and Figure S7 for alcohol consumption in the all-sexes, male and female datasets respectively
- Figure S8, Figure S9 and Figure S10 for AUDIT-C in the all-sexes, male and female datasets respectively
- Figure S11, Figure S12 and Figure S13 for AUD in the all-sexes, male and female datasets respectively

Association estimates (from traditional regression) are presented on the above figures and in ‘mrEstimate_inc.xlsx’ under methods prefixed ‘assoc. with exposure’.

Results for the ‘sign concordance test’ method are not presented as this method did not return any precision estimate. Results for the ‘Unweighted regression’ method are not presented as this method reported huge (probably wrong) confidence intervals.

## MR Robustness analyses

Robustness of the MR analyses results was investigated in several ways.

The Rucker model selection framework was applied to find representative estimates, NOME assumption statistic $I_{GX}^{2}$ was calculated and tests for heterogeneity and unbalanced pleiotropy were conducted. Results after exclusion of outlier SNPs are reported in

- mr_HeterogeneityTest_exc.xlsx - Heterogeneity test results
- mr_BalancedPleiotropyTest_exc.xlsx – balanced pleiotropy test results
- mr_RuckerModelSelection_exc.xlsx – Rucker model selection framework results
- mr_I2gx_exc.xlsx – reports $I_{GX}^{2}$ statistics

### Specimen Results

In addition, for each exposure outcome pairing, the following diagnostic plots were generated

- scatterplot of SNP-outcome versus SNP-exposure association
- forest plot of causal effect estimates
- QQ plot of Single SNP causal effect estimates
- QQ plot of Leave One SNP Out causal effect estimates
- Rücker Model Selection Framework plot
- QQ plot of SNP Cochran Q

These plots are available in files

- do2SampleMrAnalyses_<<Exposure>>_<<Outcome>>_<<CovariateSet>>.pdf

where

- <<Exposure>> = alcoholUnitsWeekly_wins140 | auditc_score | bAlcoholUseDisorder
- <<Outcome>> = iOtherNotEmp | iRetiredNotEmp | iSickNotEmp | iFamilyNotEmp | iUnempNotEmp | workHoursWeekly_wins84 | tdiDecile | highestEducAttainment | householdIncome
- << Covariates >> (covariates id) = ageSexCentreGpc | ageSexNinHouseCentreGpc

We present plots for a specimen exposure-outcome pairing. The plots for alcohol consumption exposure, TDI decile outcome are

- Figure S14 - Scatterplot of SNP - TDI associations, versus SNP - alcohol consumption associations. The strongest SNP - alcohol consumption association is probably for the ADH1B gene SNP
- Figure S15 - Quantile-Quantile plots comparing alcohol consumption on TDI causal effect estimates against Gaussian distributions. The Leave One SNP out analysis has one outlier.
- Figure S16 - Quantile-Quantile plot comparing SNP contribution to Cochran’s Q to a Chi2 df=1 distribution. The SNPs’ contribution of Cochran’s Q appears to follow an inflated Chi^2^ distribution, generally in such cases testing indicates heterogeneity in effect size and a random effects model is normally selected by Rücker model selection framework.
- Figure S17 - Rücker model Selection Framework plot

## Sample Overlap

There was overlap between the Liu et al GWAS and our analytic sample. Liu at al.’s drinks per week GWAS was the source of the SNP exposure associations we used as input to our alcohol consumption MR. Liu et al.’s Table S6 gives a breakdown of the number of participants per contributing study. The Liu et al. drinks per week GWAS was based on a dataset of 941,280 participants of which 311,126 (33.1%) were from UK Biobank.

Biases arising due to participant overlap in two sample MR analysis has been investigated theoretically and using simulation studies for continuous and discrete outcomes (Burgess et al., 2016). For one-sample MR, bias (weak instrument bias) is expected to be approximately equal to the observed exposure-outcome association bias divided by the expectation of F. For two-sample MR (with no overlap) that bias will be towards the null (preferable as it is conservative). When there is overlap, then two-sample MR bias will be a linear mixture of these two biases, and will be proportional to the instrument weakness. The relevant overlap is that in the exposure GWAS (in our case 33%) rather than in the outcome GWAS.

Burgess et al provide an R Shiny app (<https://sb452.shinyapps.io/overlap>) for estimating the expected bias due to sample overlap. We put our study parameters into that app along with their default value of the regression of outcome on exposure bias. The bias reported for 30% overlap was negligible (see Figure S19)

# Discussion

Here we elaborate on limitations of our study.

Two Sample MR assumes the exposure-SNP and outcome-SNP associations are obtained from independent datasets. This assumption was met.

Ideally in MR analyses, the outcome-SNP and exposure-SNP regressions control for the same covariates. Our outcome SNP regressions controlled for age, sex, genetic principal components and assessment centre. The exposure-SNP regressions adjusted for similar covariates.

The exposure-SNP association inputs are likely to be inflated by winner’s curse as we took our exposure-SNP associations from discovery association studies. This inflation would tend to deflate causal effect estimates and their significance. The estimates of $I_{GX}^{2}$ at around 0.98 implies the degree of regression dilution would have negligible effect on the magnitude of our casual effect estimates.

For some exposure-outcome pairings, individual SNP contributions to Cochran’s Q appear distributed as an inflated ChiSq df=1 distribution. This heterogeneity in casual effect estimates across SNPs indicates horizontal pleiotropy and/or unmodelled confounders of the SNP-outcome regression. Some of the estimation methods employed are robust to such heterogeneity, however these estimation methods make the Instrument Strength Independent of Direct Effect (InSIDE) assumption.

Selection bias is a concern for MR study designs. Such selection can induce paths between the casual ancestors of any variable influencing likelihood of study inclusion. If both exposure and outcome were such casual ancestors, then paths would be induced between instrument SNPs and outcome. This would bias the regression of outcome on SNP (via collider bias). Furthermore, the strength of such induced paths, and thence the regression bias, would depend on the SNP exposure association strength, violating the InSIDE assumption.

# Tables

## Table S1

Table S1 Regression of exposure on polygenic risk score

| **Exposure** | **N** | **K** | **Regressor** | **Df** | **Adj Partial Rsq** | **F value** | **Prob(>F)** |
| --- | --- | --- | --- | --- | --- | --- | --- |
| Alcohol Consumption | 230570 | 65 | sex | 1 | 6.8E-02 | 16724.9 | <1E-300 |
| Alcohol Consumption | 230570 | 65 | PGS.weighted | 1 | 7.7E-03 | 1787.2 | 0.0E+00 |
| Alcohol Consumption | 230570 | 65 | assessmentCentre | 21 | 3.2E-03 | 36.1 | 9.7E-147 |
| Alcohol Consumption | 230570 | 65 | GPC5 | 1 | 1.1E-03 | 264.6 | 1.8E-59 |
| Alcohol Consumption | 230570 | 65 | GPC11 | 1 | 3.9E-04 | 91.6 | 1.1E-21 |
| Alcohol Consumption | 230570 | 65 | GPC14 | 1 | 1.6E-04 | 37.9 | 7.4E-10 |
| Alcohol Consumption | 230570 | 65 | GPC9 | 1 | 1.6E-04 | 37.2 | 1.1E-09 |
| Alcohol Consumption | 230570 | 65 | GPC16 | 1 | 1.4E-04 | 32.7 | 1.1E-08 |
| Alcohol Consumption | 230570 | 65 | age | 1 | 1.3E-04 | 30.5 | 3.3E-08 |
| Alcohol Consumption | 230570 | 65 | GPC20 | 1 | 5.1E-05 | 12.8 | 3.5E-04 |
| AUDIT-C Score | 75066 | 65 | sex | 1 | 0.06828 | 5497.2 | <1E-300 |
| AUDIT-C Score | 75066 | 65 | age | 1 | 0.01064 | 807.3 | 1.2E-176 |
| AUDIT-C Score | 75066 | 65 | assessmentCentre | 21 | 0.00419 | 16.0 | 1.5E-58 |
| AUDIT-C Score | 75066 | 65 | GPC5 | 1 | 0.00172 | 130.1 | 4.2E-30 |
| AUDIT-C Score | 75066 | 65 | GPC11 | 1 | 0.00057 | 43.5 | 4.2E-11 |
| AUDIT-C Score | 75066 | 65 | PGS.weighted | 1 | 0.00032 | 24.8 | 6.3E-07 |
| AUDIT-C Score | 75066 | 65 | GPC9 | 1 | 0.00028 | 21.8 | 3.0E-06 |
| AUDIT-C Score | 75066 | 65 | GPC16 | 1 | 0.00022 | 17.4 | 3.0E-05 |
| AUDIT-C Score | 75066 | 65 | GPC14 | 1 | 0.00019 | 15.5 | 8.3E-05 |
| Alcohol Use Disorder | 230775 | 65 | sex | 1 | 4.2E-03 | 864.8 | 9.74E-190 |
| Alcohol Use Disorder | 230775 | 65 | assessmentCentre | 21 | 1.5E-03 | 11.6 | 1.0E-39 |
| Alcohol Use Disorder | 230775 | 65 | GPC5 | 1 | 3.0E-04 | 58.4 | 2.1E-14 |
| Alcohol Use Disorder | 230775 | 65 | PGS.weighted | 1 | 1.4E-04 | 30.9 | 2.8E-08 |
| Alcohol Use Disorder | 230775 | 65 | GPC9 | 1 | 5.1E-05 | 14.1 | 1.8E-04 |

Footnote: The table is truncated to the set of regressors with Prob(>F) < 0.001. GPC = Genetic Principal Component. PGS = polygenic score. Columns: N = regression dataset size i.e. the number of participants with complete data, K = number of parameters estimated in the regression, Df = number of regression parameters used by regressor.

## Table S2

Table S2 UK Biobank Fields used in the study

| **UK Biobank field** | **Type** | **Description** | **Comment** |
| --- | --- | --- | --- |
|  |  |  |  |
| **QC Related** | |  |  |
| 21000 | categorical | Ethnic background | An amalgam of sequential branching questions asked during the initial Assessment Centre visit |
| 22006 | dichotomous | Genetic ethnic grouping | [Indicates samples who self-identified as 'White British' according to Field 21000 and have very similar genetic ancestry based on a principal components analysis of the genotypes.](http://biobank.ctsu.ox.ac.uk/showcase/field.cgi?id=21000) |
| 22019 | dichotomous | Sex chromosome aneuploidy | Sex chromosome aneuploidy marker. This indicates samples which were identified as putatively carrying sex chromosome configurations that are not either XX or XY. |
| 22027 | dichotomous | Outliers for heterozygosity or missing rate | Indicates samples identified as outliers in heterozygosity and missing rates, which implies that the genotypes for these samples are of poor quality. |
| 22005 | continuous | Missingness | Missing rate of each sample based on a set of high-quality markers. |
|  |  |  |  |
| 22001 | dichotomous | Genetic sex | Sex as determined from genotyping analysis. |
| 31 | dichotomous | Sex | Sex of participant. Acquired from central registry at recruitment, but in some cases updated by the participant. |
|  |  |  |  |
| 22009 | continuous | Genetic principal components | Score for each principal component 1-40 |
| 54 | categorical | UK Biobank Assessment Centre | UK Biobank assessment centre at which participant consented, 1 of 22 centres |
| 22021 | continuous | Genetic kinship to other participants | A threshold lowest kinship coefficient of 0.042 (equivalent to second cousins) was used to identify overly related pairs of participants |
|  |  |  |  |
| **Outcome Related** | |  |  |
| 6142 | categorical | Current employment status was self-reported |  |
| 767 | continuous | Length of working week for main job | Collected from all the participants who indicated they were in paid employment or self-employed (see Field 6142) |
| 189 | continuous | Townsend Deprivation Index at recruitment | Townsend Deprivation Index calculated immediately prior to participant joining UK Biobank. |
| 738 | ordinal | Average total household income before tax | Collected from participants except those who indicated they were living in a sheltered accommodation or in a care home |
| 6138 | ordinal | Education Qualifications |  |
|  |  |  |  |
| **Exposure Related** | |  |  |
| 20002 | categorical | Non-cancer illness code, self-reported |  |
| 41202 | categorical | Diagnoses - main ICD10 |  |
| 41204 | categorical | Diagnoses - secondary ICD10 | not used |
| 41203 | categorical | Diagnoses - main ICD9 |  |
| 41205 | categorical | Diagnoses - secondary ICD9 | not used |
|  |  |  |  |
| 20414 | Ordinal | Frequency of drinking alcohol | Used in construction of AUDIT-C score |
| 20403 | Ordinal | Amount of alcohol drunk on a typical drinking day | Used in construction of AUDIT-C score |
| 20416 | Ordinal | Frequency of consuming six or more units of alcohol | Used in construction of AUDIT-C score |

## Table S3

Table S3 Instrument SNP sets used in the study

| **SNP** | **Gene** | **chr** | **Effect**  **allele** | **Other**  **allele** | **Effect**  **Allele**  **Freq** | **Link**  **Function** | **beta** | **se** | **P**  **Value** |
| --- | --- | --- | --- | --- | --- | --- | --- | --- | --- |
|  |  |  |  |  |  |  |  |  |  |
| **Alcohol Consumption** | | |  |  |  |  |  |  |  |
| rs1229984 | ADH1B | 4 | C | T | 0.963 | linear | 0.15053 | 0.00510 | 1.0E-200 |
| rs11940694 | KLB | 4 | G | A | 0.597 | linear | 0.02595 | 0.00153 | 3.0E-68 |
| rs1260326 | GCKR | 2 | C | T | 0.601 | linear | 0.02089 | 0.00153 | 8.0E-45 |
| rs79139602 | C4orf17 | 4 | T | A | 0.021 | linear | 0.06027 | 0.00510 | 2.0E-32 |
| rs13107325 | SLC39A8 | 4 | T | C | 0.072 | linear | -0.02750 | 0.00281 | 2.0E-22 |
| rs2165670 |  | 4 | A | G | 0.106 | linear | 0.02308 | 0.00255 | 2.0E-22 |
| rs13383034 | LINC01833 | 2 | T | C | 0.329 | linear | 0.01493 | 0.00153 | 6.0E-22 |
| rs62250685 | CADM2 | 3 | G | A | 0.614 | linear | -0.01436 | 0.00153 | 1.0E-21 |
| rs281379 |  | 19 | A | G | 0.508 | linear | 0.01372 | 0.00153 | 5.0E-21 |
| rs6951574 | DPP6 | 7 | C | T | 0.458 | linear | 0.01322 | 0.00153 | 2.0E-19 |
| rs378421 | NPIPB9 | 16 | A | G | 0.404 | linear | -0.01121 | 0.00148 | 5.0E-14 |
| rs4699791 |  | 4 | A | G | 0.096 | linear | 0.01857 | 0.00230 | 7.0E-14 |
| rs17177078 | TNRC6A | 16 | T | C | 0.063 | linear | -0.02232 | 0.00306 | 1.0E-13 |
| rs56030824 | SPI1 | 11 | A | G | 0.322 | linear | -0.01160 | 0.00158 | 1.0E-13 |
| rs13024996 | ARHGAP15 | 2 | A | C | 0.364 | linear | -0.01091 | 0.00153 | 6.0E-13 |
| rs28929474 | SERPINA1 | 14 | T | C | 0.018 | linear | -0.03680 | 0.00536 | 1.0E-11 |
| rs4690727 | INPP4B | 4 | G | C | 0.718 | linear | 0.01082 | 0.00163 | 2.0E-11 |
| rs4916723 | LINC00461 | 5 | C | A | 0.416 | linear | -0.00995 | 0.00148 | 2.0E-11 |
| rs11692435 | ACTR1B | 2 | A | G | 0.085 | linear | 0.01745 | 0.00281 | 3.0E-11 |
| rs10750025 |  | 11 | T | C | 0.686 | linear | 0.01032 | 0.00158 | 5.0E-11 |
| rs77165542 |  | 2 | T | C | 0.035 | linear | -0.02601 | 0.00408 | 6.0E-11 |
| rs10978550 |  | 9 | C | T | 0.206 | linear | -0.01175 | 0.00181 | 7.0E-11 |
| rs1217091 |  | 8 | C | T | 0.812 | linear | 0.01216 | 0.00186 | 7.0E-11 |
| rs13094887 |  | 3 | T | A | 0.301 | linear | -0.01031 | 0.00158 | 9.0E-11 |
| rs113443718 | SEZ6L2 | 16 | A | G | 0.305 | linear | -0.01021 | 0.00158 | 1.0E-10 |
| rs4938230 |  | 11 | A | C | 0.842 | linear | 0.01281 | 0.00199 | 1.0E-10 |
| rs62044525 |  | 16 | G | C | 0.184 | linear | -0.01217 | 0.00189 | 1.0E-10 |
| rs6460047 |  | 7 | C | T | 0.208 | linear | 0.01162 | 0.00179 | 1.0E-10 |
| rs7950166 | TRIM66 | 11 | T | C | 0.637 | linear | -0.00980 | 0.00153 | 1.0E-10 |
| rs3803800 | TNFSF13/TNFSF12-TNFSF13 | 17 | G | A | 0.786 | linear | 0.01138 | 0.00179 | 2.0E-10 |
| rs56337305 | LOC105373909 | 2 | C | T | 0.383 | linear | -0.00959 | 0.00151 | 2.0E-10 |
| rs13032049 | WDPCP | 2 | G | A | 0.283 | linear | 0.01019 | 0.00163 | 3.0E-10 |
| rs2472297 |  | 15 | T | C | 0.249 | linear | 0.01061 | 0.00168 | 3.0E-10 |
| rs2764771 |  | 16 | A | G | 0.307 | linear | 0.00989 | 0.00158 | 4.0E-10 |
| rs28680958 | ZBTB37 | 1 | A | G | 0.217 | linear | -0.01100 | 0.00179 | 5.0E-10 |
| rs4501255 | BEND4 | 4 | G | C | 0.235 | linear | 0.01069 | 0.00173 | 5.0E-10 |
| rs10506274 | ACSS3 | 12 | T | G | 0.484 | linear | -0.00904 | 0.00145 | 6.0E-10 |
| rs2854334 |  | 17 | G | A | 0.615 | linear | 0.00922 | 0.00151 | 8.0E-10 |
| rs58107686 | PHC2 | 1 | A | C | 0.328 | linear | -0.00975 | 0.00161 | 8.0E-10 |
| rs705687 |  | 1 | G | A | 0.785 | linear | -0.01090 | 0.00179 | 8.0E-10 |
| rs9950000 | TCF4 | 18 | T | C | 0.395 | linear | -0.00912 | 0.00148 | 9.0E-10 |
| rs10236149 | ARPC1B | 7 | G | A | 0.123 | linear | -0.01350 | 0.00222 | 1.0E-09 |
| rs36052336 | ADH1C | 4 | G | A | 0.061 | linear | -0.01843 | 0.00306 | 1.0E-09 |
| rs3809162 | HNRNPA1/CBX5 | 12 | G | A | 0.397 | linear | 0.00906 | 0.00151 | 1.0E-09 |
| rs823114 | NUCKS1 | 1 | A | G | 0.553 | linear | 0.00877 | 0.00145 | 2.0E-09 |
| rs828867 | TET3 | 2 | A | G | 0.545 | linear | 0.00876 | 0.00145 | 2.0E-09 |
| rs4842786 |  | 12 | A | G | 0.584 | linear | -0.00880 | 0.00148 | 3.0E-09 |
| rs5024204 | PTGER3 | 1 | T | A | 0.278 | linear | 0.00970 | 0.00163 | 3.0E-09 |
| rs13066454 |  | 3 | T | C | 0.398 | linear | -0.00878 | 0.00148 | 4.0E-09 |
| rs72859280 |  | 2 | T | G | 0.036 | linear | 0.02289 | 0.00408 | 4.0E-09 |
| rs500321 | WASF3 | 13 | T | A | 0.736 | linear | -0.00967 | 0.00166 | 5.0E-09 |
| rs55872084 | SGCD | 5 | T | G | 0.235 | linear | 0.00998 | 0.00171 | 6.0E-09 |
| rs2011092 | ZBTB38 | 3 | C | T | 0.339 | linear | -0.00890 | 0.00153 | 7.0E-09 |
| rs1123285 | OTX2 | 14 | G | C | 0.335 | linear | -0.00890 | 0.00153 | 8.0E-09 |
| rs12655091 |  | 5 | A | G | 0.530 | linear | -0.00831 | 0.00148 | 1.0E-08 |
| rs12907323 | AGBL1 | 15 | G | A | 0.411 | linear | 0.00850 | 0.00148 | 1.0E-08 |
| rs2180870 | ARID4A | 14 | C | T | 0.135 | linear | -0.01218 | 0.00214 | 1.0E-08 |
| rs4815364 | ACSS1 | 20 | A | G | 0.616 | linear | 0.00858 | 0.00151 | 1.0E-08 |
| rs55932213 |  | 9 | G | A | 0.736 | linear | 0.00949 | 0.00166 | 1.0E-08 |
| rs10438820 | RPTOR | 17 | T | C | 0.702 | linear | 0.00897 | 0.00161 | 2.0E-08 |
| rs11030084 | BDNF-AS/LINC00678 | 11 | T | C | 0.184 | linear | -0.01061 | 0.00189 | 2.0E-08 |
| rs12088813 | PDE4B | 1 | C | A | 0.267 | linear | -0.00933 | 0.00166 | 2.0E-08 |
| rs17665139 |  | 10 | T | C | 0.149 | linear | -0.01156 | 0.00207 | 2.0E-08 |
| rs3748034 | HGFAC | 4 | T | G | 0.143 | linear | -0.01174 | 0.00207 | 2.0E-08 |
| rs682011 |  | 11 | C | T | 0.559 | linear | 0.00821 | 0.00148 | 2.0E-08 |
| rs7074871 | LINC02661 | 10 | A | G | 0.255 | linear | -0.00940 | 0.00168 | 2.0E-08 |
| rs11625650 | KIF26A | 14 | A | G | 0.233 | linear | -0.00957 | 0.00171 | 3.0E-08 |
| rs12795042 | LINC02743 | 11 | C | A | 0.623 | linear | -0.00832 | 0.00151 | 3.0E-08 |
| rs35034355 | ORC5 | 7 | A | G | 0.521 | linear | -0.00810 | 0.00148 | 3.0E-08 |
| rs4548913 | SRR | 17 | A | G | 0.632 | linear | -0.00836 | 0.00151 | 3.0E-08 |
| rs9838144 | CPNE4/LOC105374113 | 3 | C | G | 0.209 | linear | -0.00996 | 0.00179 | 3.0E-08 |
| rs10753661 |  | 1 | A | G | 0.684 | linear | -0.00864 | 0.00156 | 4.0E-08 |
| rs1713676 |  | 11 | G | A | 0.522 | linear | -0.00799 | 0.00148 | 4.0E-08 |
| rs4092465 |  | 18 | G | A | 0.635 | linear | -0.00829 | 0.00153 | 4.0E-08 |
| rs6787172 | RSRC1 | 3 | G | T | 0.554 | linear | -0.00803 | 0.00145 | 4.0E-08 |
| rs7185555 |  | 16 | C | G | 0.153 | linear | -0.01110 | 0.00204 | 4.0E-08 |
| rs9607814 |  | 22 | A | C | 0.200 | linear | -0.01018 | 0.00186 | 4.0E-08 |
|  |  |  |  |  |  |  |  |  |  |
| **Alcohol Use Disorder** | | | |  |  |  |  |  |  |
| rs1229984 | ADH1B | NA | T | C | 0.022 | logit | -0.51190 | 0.02540 | 2.2E-90 |
| rs1789882 | ADH1B | NA | A | G | 0.177 | logit | -0.13790 | 0.01600 | 6.8E-18 |
| rs13107325 | SLC39A8 | NA | T | C | 0.074 | logit | -0.12040 | 0.01540 | 4.6E-15 |
| rs1260326 | GCKR | NA | T | C | 0.394 | logit | -0.05760 | 0.00770 | 6.5E-14 |
| rs4936277 |  | NA | A | G | 0.547 | logit | 0.05390 | 0.00720 | 1.1E-13 |
| rs1421085 | FTO | NA | T | C | 0.598 | logit | 0.05310 | 0.00790 | 1.6E-11 |
| rs540606 |  | NA | A | G | 0.448 | logit | -0.05470 | 0.00850 | 1.0E-10 |
| rs7906104 | LINC02661 | NA | T | C | 0.260 | logit | -0.04590 | 0.00790 | 5.2E-09 |
| rs284790 |  | NA | T | G | 0.305 | logit | -0.04100 | 0.00750 | 4.5E-08 |
|  |  |  |  |  |  |  |  |  |  |
| **AUDIT-C Score** | | |  |  |  |  |  |  |  |
| rs1229984 | ADH1B | NA | T | C | 0.022 | linear | -0.34140 | 0.01380 | 3.0E-134 |
| rs13107325 | SLC39A8 | NA | T | C | 0.074 | linear | -0.10810 | 0.00940 | 2.4E-30 |
| rs17817964 | FTO | NA | T | C | 0.393 | linear | -0.04540 | 0.00500 | 2.3E-19 |
| rs1260326 | GCKR | NA | T | C | 0.394 | linear | -0.04130 | 0.00490 | 3.6E-17 |
| rs1693457 | ADH1B | NA | T | C | 0.821 | linear | 0.08750 | 0.01110 | 3.6E-15 |
| rs2953441 |  | NA | T | C | 0.628 | linear | 0.03370 | 0.00540 | 5.1E-10 |
| rs4975013 | KLB | NA | A | G | 0.369 | linear | -0.03070 | 0.00510 | 1.6E-09 |
| rs9902512 | IGF2BP1 | NA | C | G | 0.645 | linear | -0.03230 | 0.00560 | 6.2E-09 |
| rs75723348 |  | NA | T | G | 0.714 | linear | 0.03010 | 0.00520 | 9.4E-09 |
| rs2961816 |  | NA | A | C | 0.708 | linear | 0.02840 | 0.00500 | 1.1E-08 |
| rs4423856 |  | NA | T | C | 0.778 | linear | 0.03660 | 0.00650 | 1.5E-08 |
| rs10849916 | MYL2 | NA | T | G | 0.345 | linear | -0.39540 | 0.06990 | 1.6E-08 |
| rs10022174 | LOC100507053 | NA | A | G | 0.918 | linear | 0.04040 | 0.00720 | 1.6E-08 |
| rs2031496 | GIPC2 | NA | T | C | 0.568 | linear | 0.02760 | 0.00510 | 4.7E-08 |

## Table S4

Table S4 Correlation between alcohol exposure PRSs in all-sexes dataset

|  | **Alcohol Consumption** | **AUDIT-C** | **Alcohol Use Disorder** |
| --- | --- | --- | --- |
| **Alcohol Consumption** | 1 | 0.1 | 0.14 |
| **AUDIT-C** | 0.1 | 1 | 0.34 |
| **Alcohol Use Disorder** | 0.14 | 0.34 | 1 |

Footnote: Correlations in male only and female only datasets were similar.

## Table S5

Table S5 Association of alcohol exposures with outcomes estimated by standard multivariable regression

| **Outcome** | **N** | **Effect**  **Scale** | **Effect** | **Effect**  **95 CI** | **Effect**  **P Value** |  |
| --- | --- | --- | --- | --- | --- | --- |
|  |  |  |  |  |  |  |
| **Alcohol Consumption** |  |  |  |  |  |  |
| Not in paid employment | 227057 | Odds Ratio | 0.9987 | (0.998, 0.999) | 4.0E-05 | *** |
| Retired | 204342 | Odds Ratio | 1.0043 | (1.003, 1.005) | 3.3E-25 | *** |
| Sick/Disabled | 185185 | Odds Ratio | 0.9844 | (0.983, 0.986) | 2.1E-112 | *** |
| Caring for Home/Family | 182074 | Odds Ratio | 0.9947 | (0.993, 0.996) | 3.5E-09 | *** |
| Unemployed | 178684 | Odds Ratio | 0.9998 | (0.998, 1.001) | 7.7E-01 |  |
| Highest Educational Attainment | 203973 | Odds Ratio | 0.9980 | (0.998, 0.999) | 1.7E-16 | *** |
| Household Income | 206520 | Odds Ratio | 1.0129 | (1.012, 1.013) | <1E-324 | *** |
| Hours Worked Weekly | 171523 | Response per unit | 0.0164 | (0.013, 0.02) | 9.1E-23 | *** |
| Townsend Deprivation Index Decile | 230261 | Response per unit | -0.0023 | (-0.003, -0.002) | 3.9E-12 | *** |
|  |  |  |  |  |  |  |
| **AUDIT-C Score** |  |  |  |  |  |  |
| Not in paid employment | 74013 | Odds Ratio | 1.0003 | (0.993, 1.008) | 9.4E-01 |  |
| Retired | 69476 | Odds Ratio | 1.0252 | (1.016, 1.035) | 1.5E-07 | *** |
| Sick/Disabled | 60036 | Odds Ratio | 0.9176 | (0.898, 0.938) | 5.3E-15 | *** |
| Caring for Home/Family | 61258 | Odds Ratio | 0.9760 | (0.96, 0.992) | 3.5E-03 | * |
| Unemployed | 59782 | Odds Ratio | 1.0017 | (0.98, 1.024) | 8.8E-01 |  |
| Highest Educational Attainment | 71413 | Odds Ratio | 0.9858 | (0.981, 0.991) | 2.0E-08 | *** |
| Household Income | 69601 | Odds Ratio | 1.0855 | (1.08, 1.091) | 2.4E-209 | *** |
| Hours Worked Weekly | 58079 | Response per unit | 0.1106 | (0.075, 0.146) | 8.6E-10 | *** |
| Townsend Deprivation Index Decile | 74975 | Response per unit | 0.0058 | (-0.001, 0.013) | 1.1E-01 |  |
|  |  |  |  |  |  |  |
| **Alcohol Use Disorder** |  |  |  |  |  |  |
| Not in paid employment | 227243 | Odds Ratio | 4.1995 | (3.888, 4.536) | 8.6E-292 | *** |
| Retired | 204459 | Odds Ratio | 1.6265 | (1.443, 1.833) | 1.7E-15 | *** |
| Sick/Disabled | 185340 | Odds Ratio | 9.5233 | (8.719, 10.402) | 0.0E+00 | *** |
| Caring for Home/Family | 182181 | Odds Ratio | 1.9096 | (1.52, 2.399) | 2.7E-08 | *** |
| Unemployed | 178800 | Odds Ratio | 5.1433 | (4.486, 5.896) | 4.9E-122 | *** |
| Highest Educational Attainment | 204124 | Odds Ratio | 0.8292 | (0.774, 0.888) | 8.7E-08 | *** |
| Household Income | 206633 | Odds Ratio | 0.3243 | (0.302, 0.348) | 1.6E-212 | *** |
| Hours Worked Weekly | 171617 | Response per unit | -1.1118 | (-1.676, -0.547) | 1.1E-04 | ** |
| Townsend Deprivation Index Decile | 230466 | Response per unit | 1.5731 | (1.481, 1.665) | 4.7E-245 | *** |

Associations are for men and women combined. All associations are adjusted for age, sex, assessment centre, and genetic principal components. The household income association is additionally adjusted for number in household (winsorised to 12). Being in paid employment is the reference category for outcomes – Not in paid employment, Retired, Sick/Disabled, Caring for Home/Family and Unemployed. For ordinal outcomes (Highest Educational Attainment and Household Income), the reported odds ratio gives the relative odds of a greater outcome level if the exposure is increased by one unit.

# Figures

## Figure S1

Figure S1 STROBE flowchart of UK Biobank participant exclusions


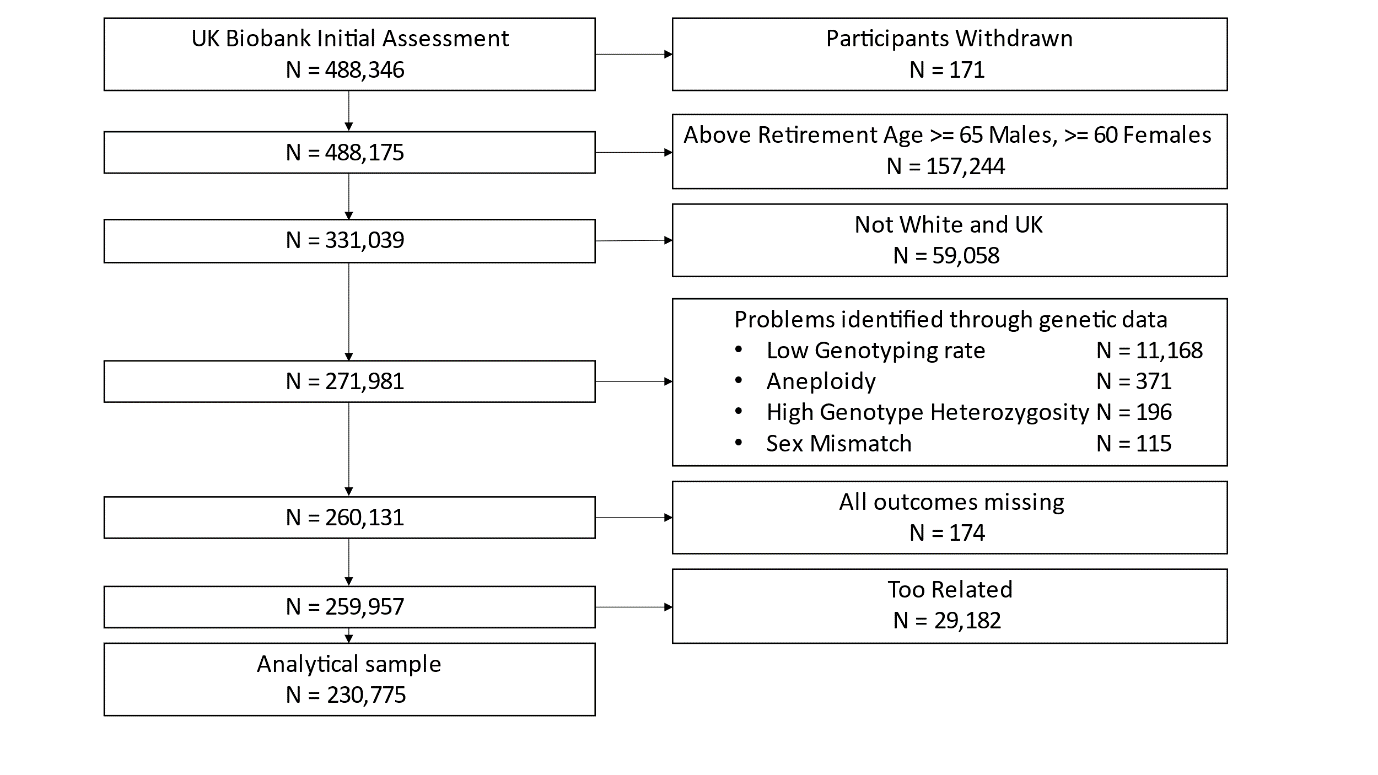


## Flowcharts of instrument SNP screening

### Figure S2

Figure S2 Flowchart of SNP screening for the MR instrument for AUDIT-C


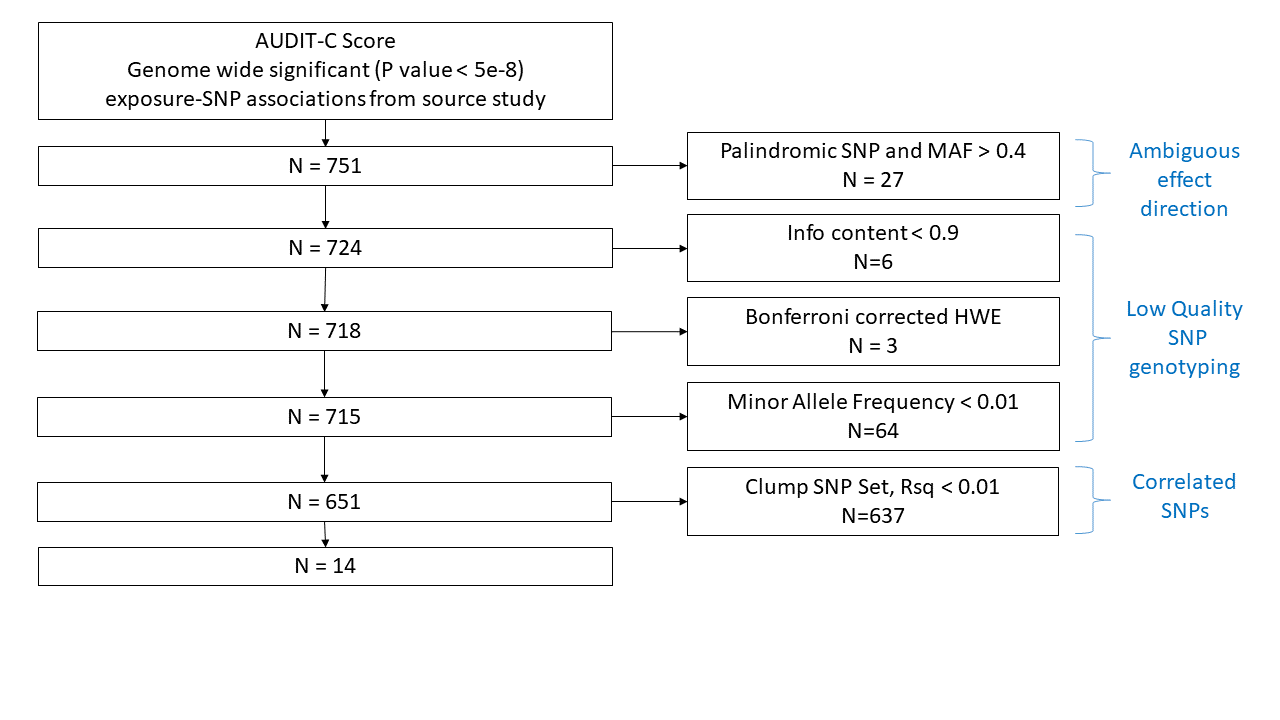


### Figure S3

Figure S3 Flowchart of SNP screening for the MR instrument for AUD


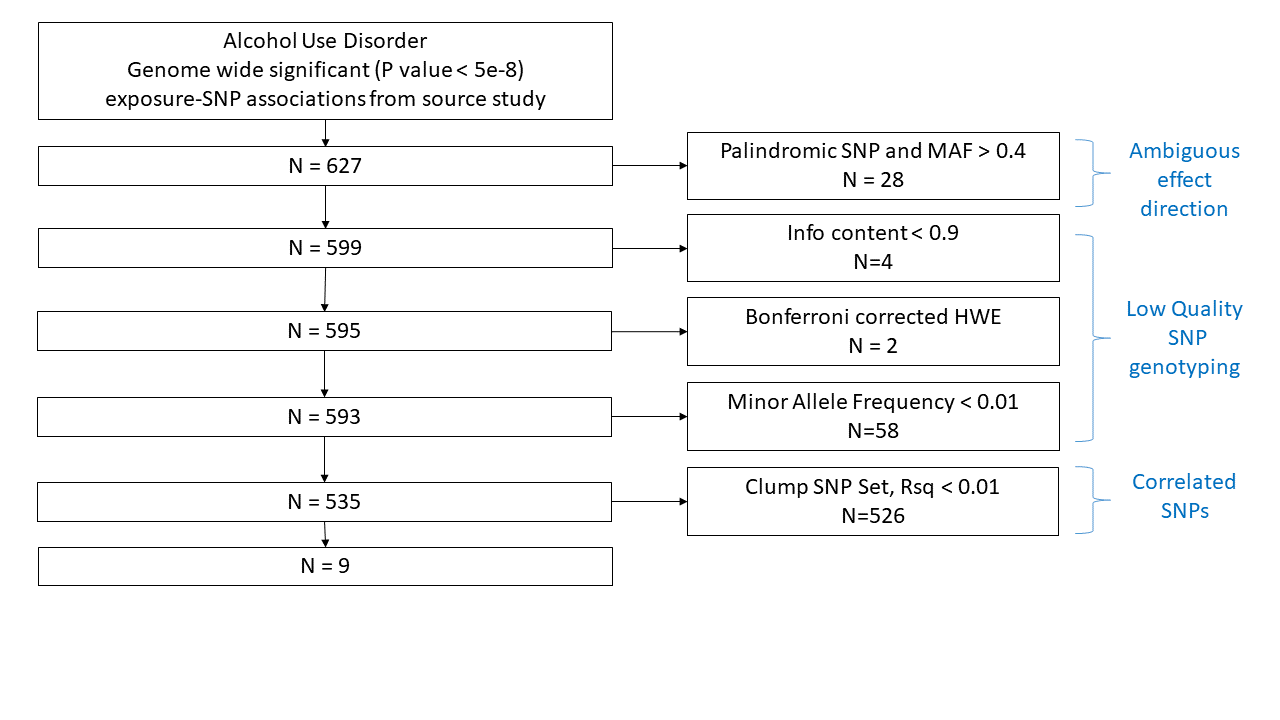


### Figure S4

Figure S4 Flowchart of SNP screening for the MR instrument for Alcohol Consumption


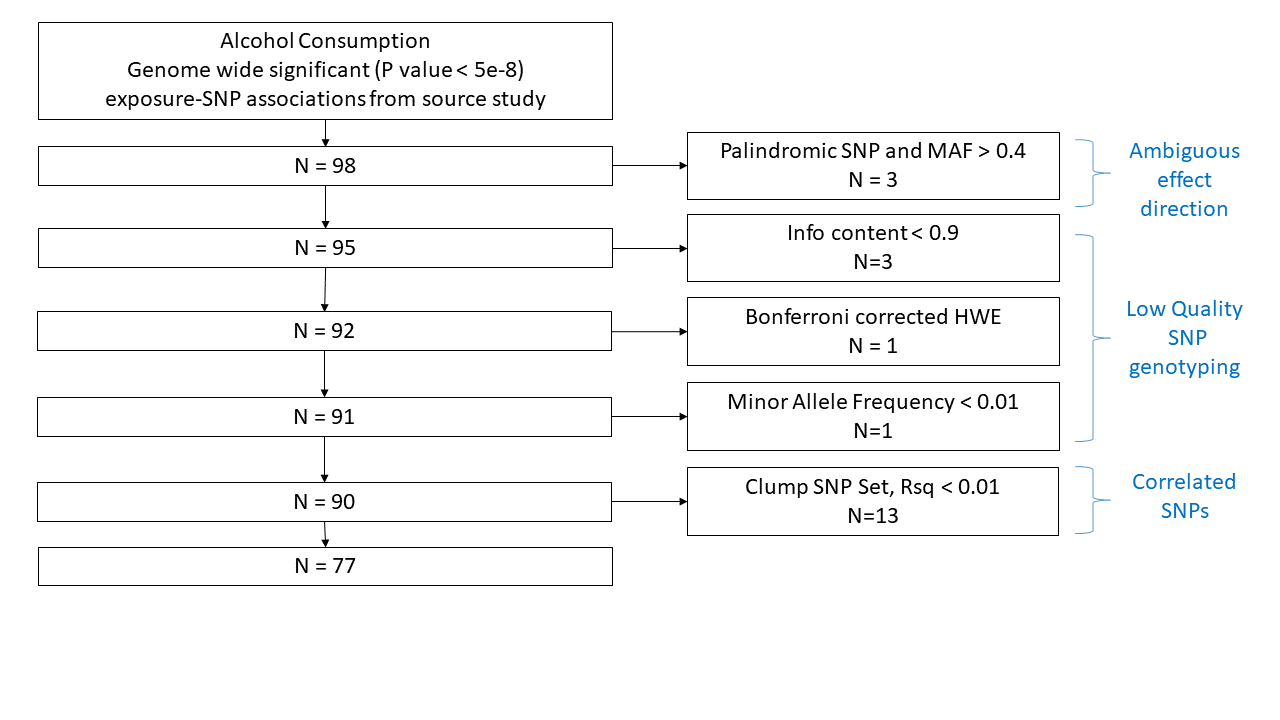


## MR effect estimates for Alcohol Consumption

### Figure S5

Figure S5 Forest plots of causal effect estimates of alcohol consumption on outcomes, for all-sexes dataset.


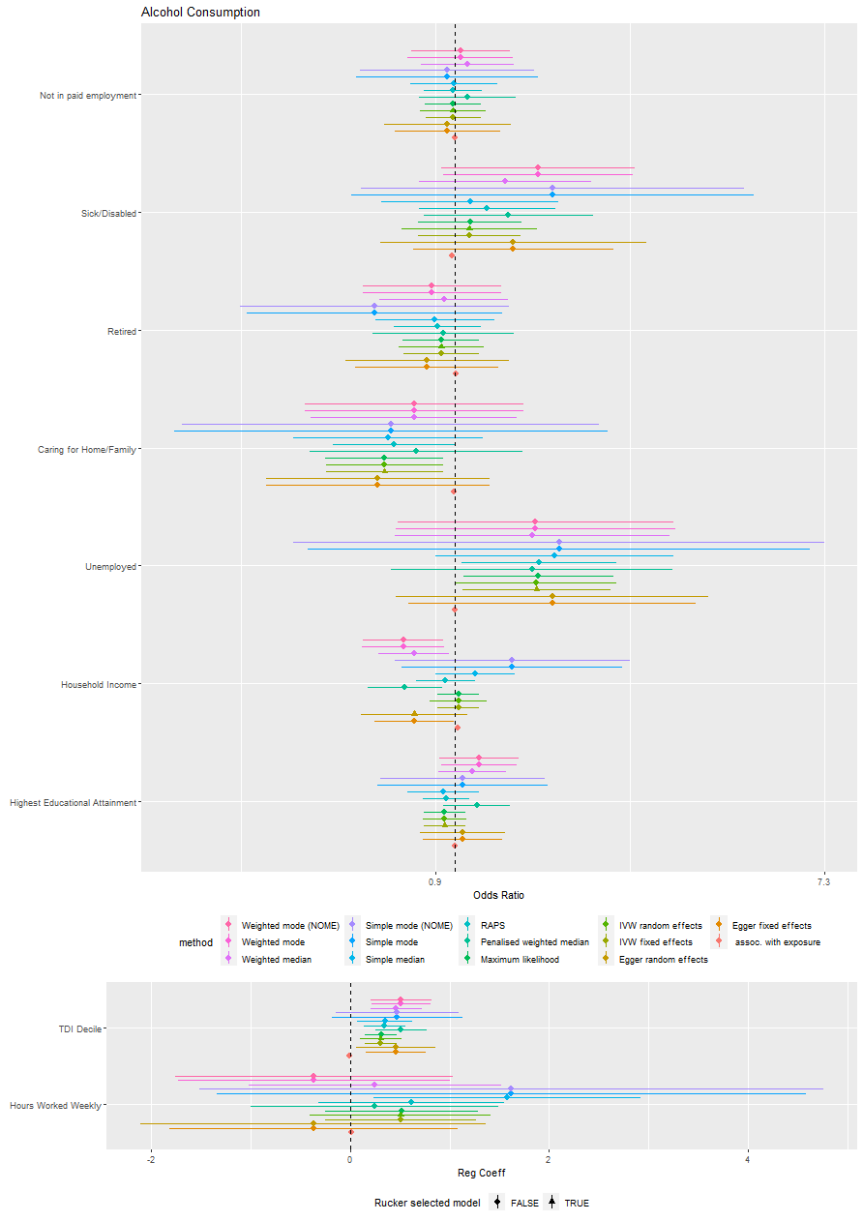


Footnote: Causal effect estimate plus 95% Confidence Interval coloured by method. Association estimates are presented in method titled ‘assoc. with exposure’.

### Figure S6

Figure S6 Forest plots of causal effect estimates of alcohol consumption on outcomes, for men.


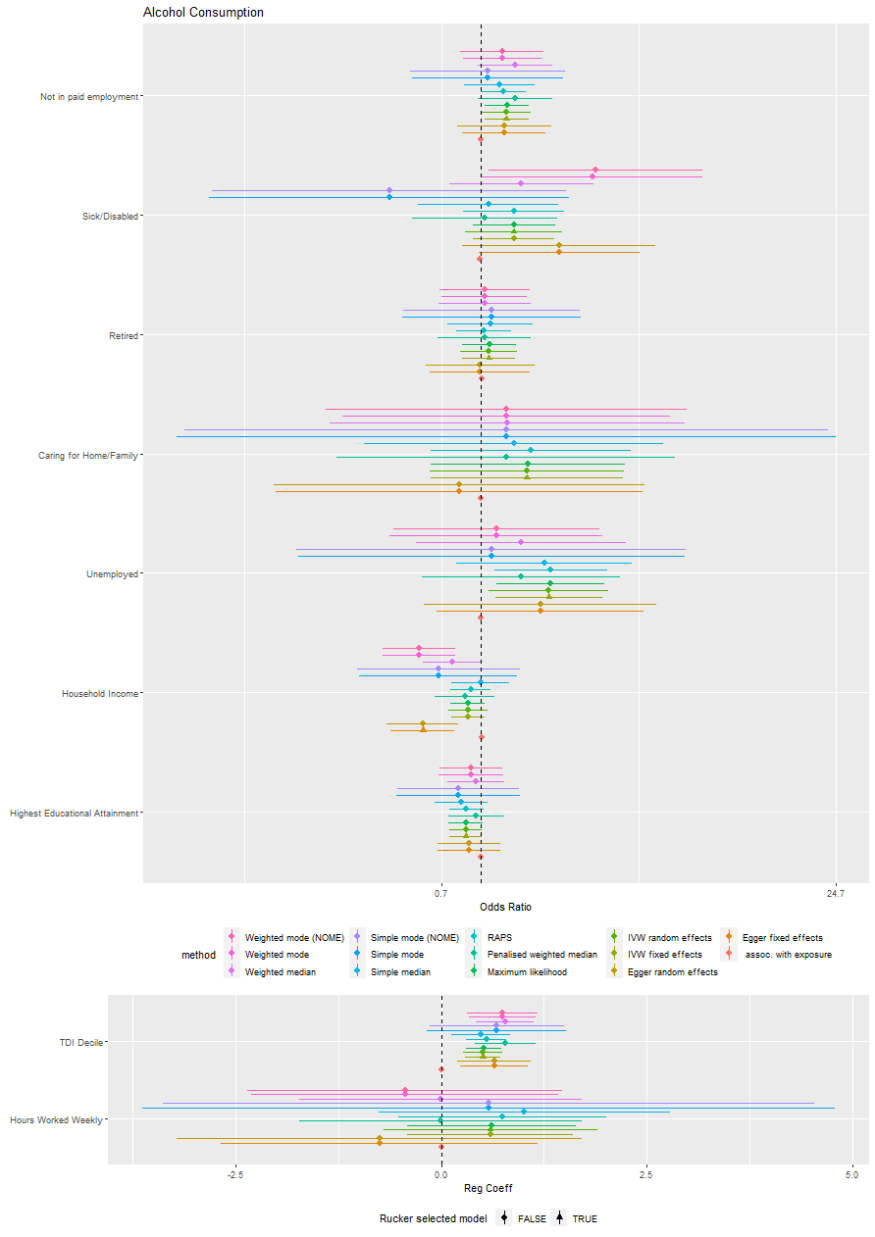


Footnote: Causal effect estimate plus 95% Confidence Interval coloured by method. Association estimates are presented in method titled ‘assoc. with exposure’.

### Figure S7

Figure S7 Forest plots of causal effect estimates of alcohol consumption on outcomes, for women.


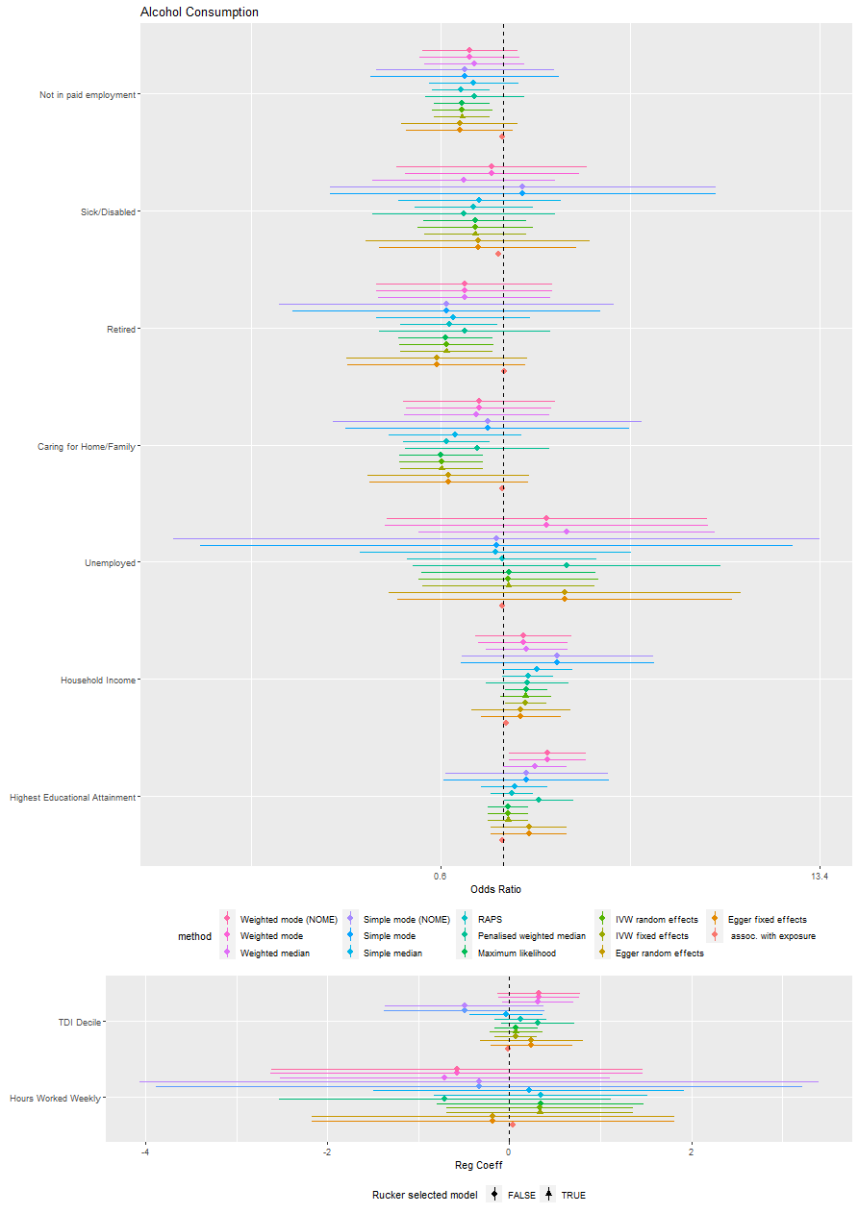


Footnote: Causal effect estimate plus 95% Confidence Interval coloured by method. Association estimates are presented in method titled ‘assoc. with exposure’.

## MR effect estimates for AUDIT-C

### Figure S8

Figure S8 Forest plots of causal effect estimates of AUDIT-C on outcomes, for all-sexes dataset.


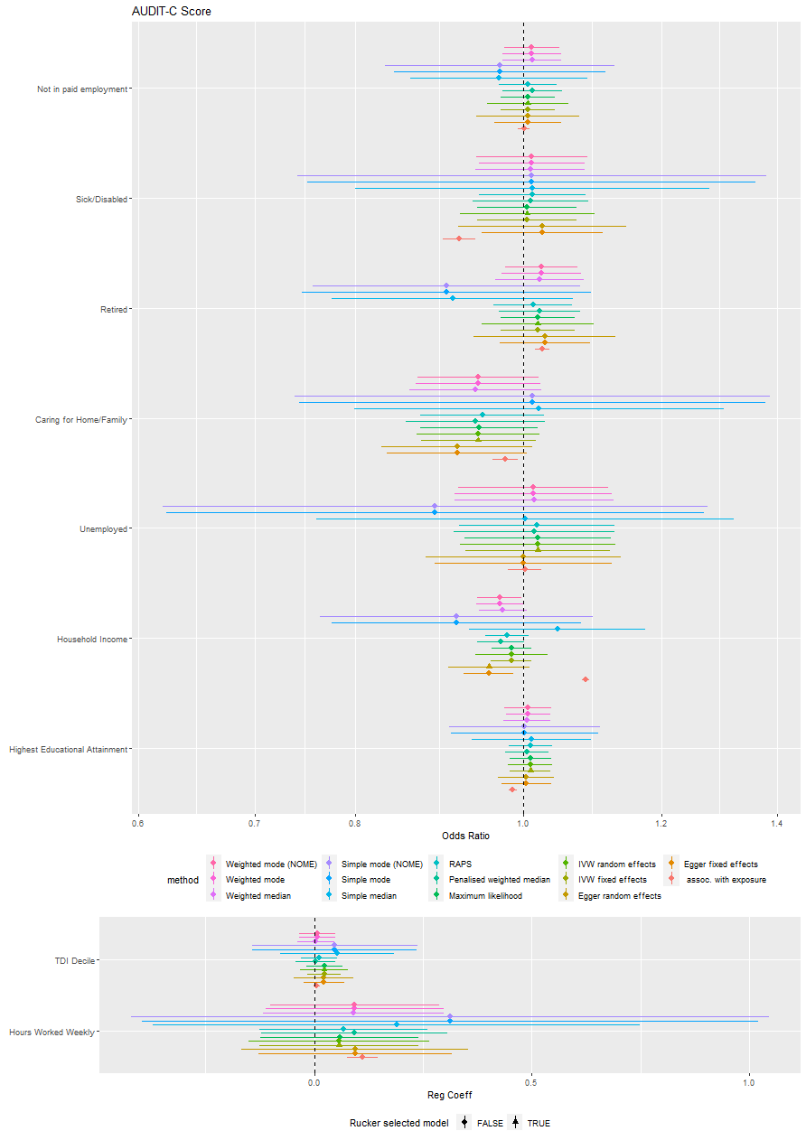


Footnote: Causal effect estimate plus 95% Confidence Interval coloured by method. Association estimates are presented in method titled ‘assoc. with exposure’.

### Figure S9

Figure S9 Forest plots of causal effect estimates of AUDIT-C on outcomes, for men.


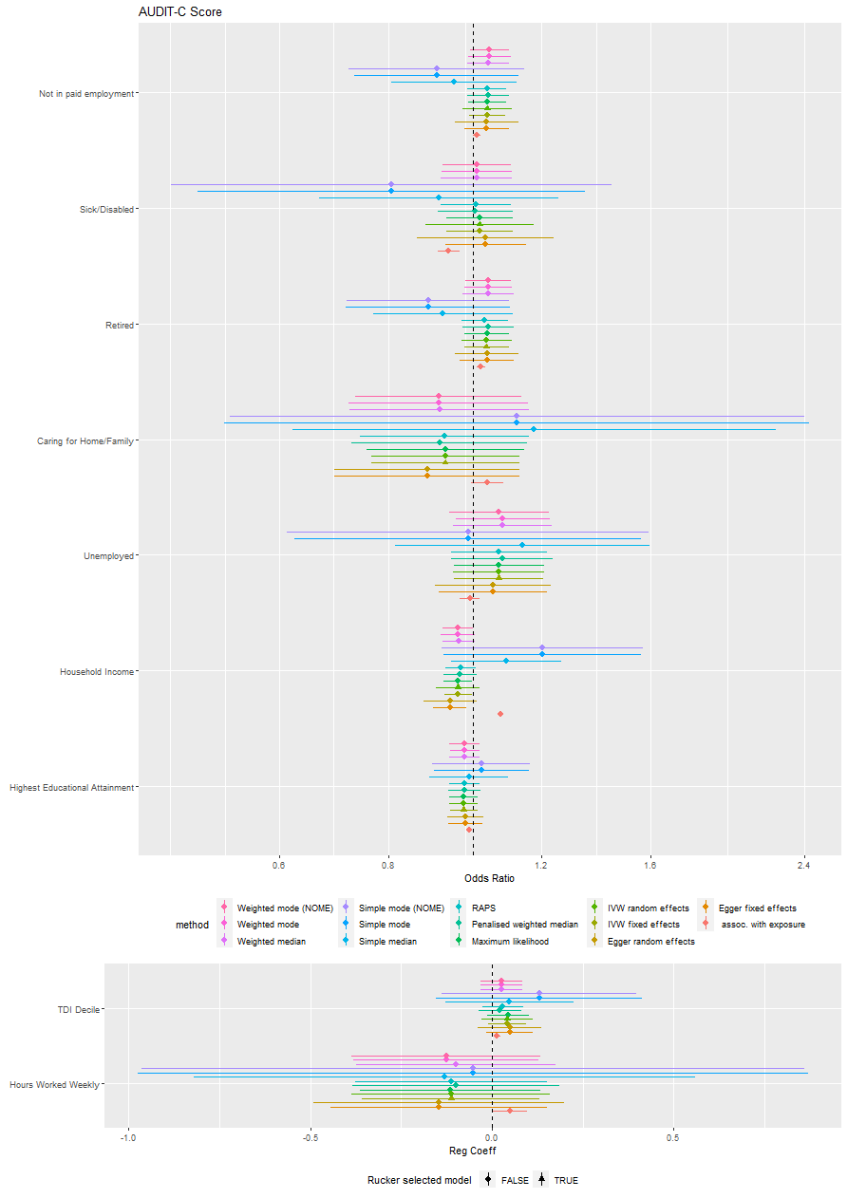


Footnote: Causal effect estimate plus 95% Confidence Interval coloured by method. Association estimates are presented in method titled ‘assoc. with exposure’.

### Figure S10

Figure S10 Forest plots of causal effect estimates of AUDIT-C on outcomes, for women.


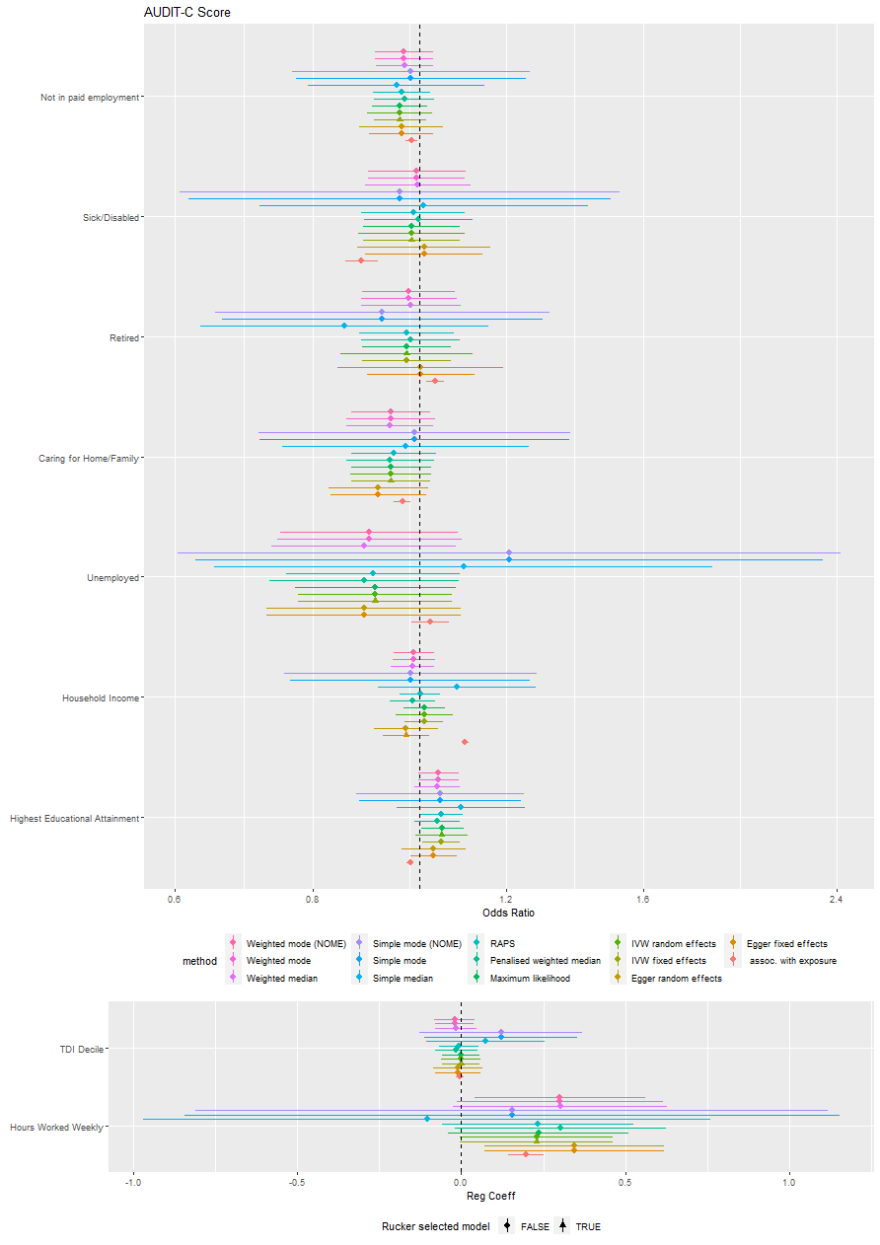


Footnote: Causal effect estimate plus 95% Confidence Interval coloured by method. Association estimates are presented in method titled ‘assoc. with exposure’.

## MR effect estimates for AUD

### Figure S11

Figure S11 Forest plots of causal effect estimates of AUD on outcomes, for all-sexes dataset.


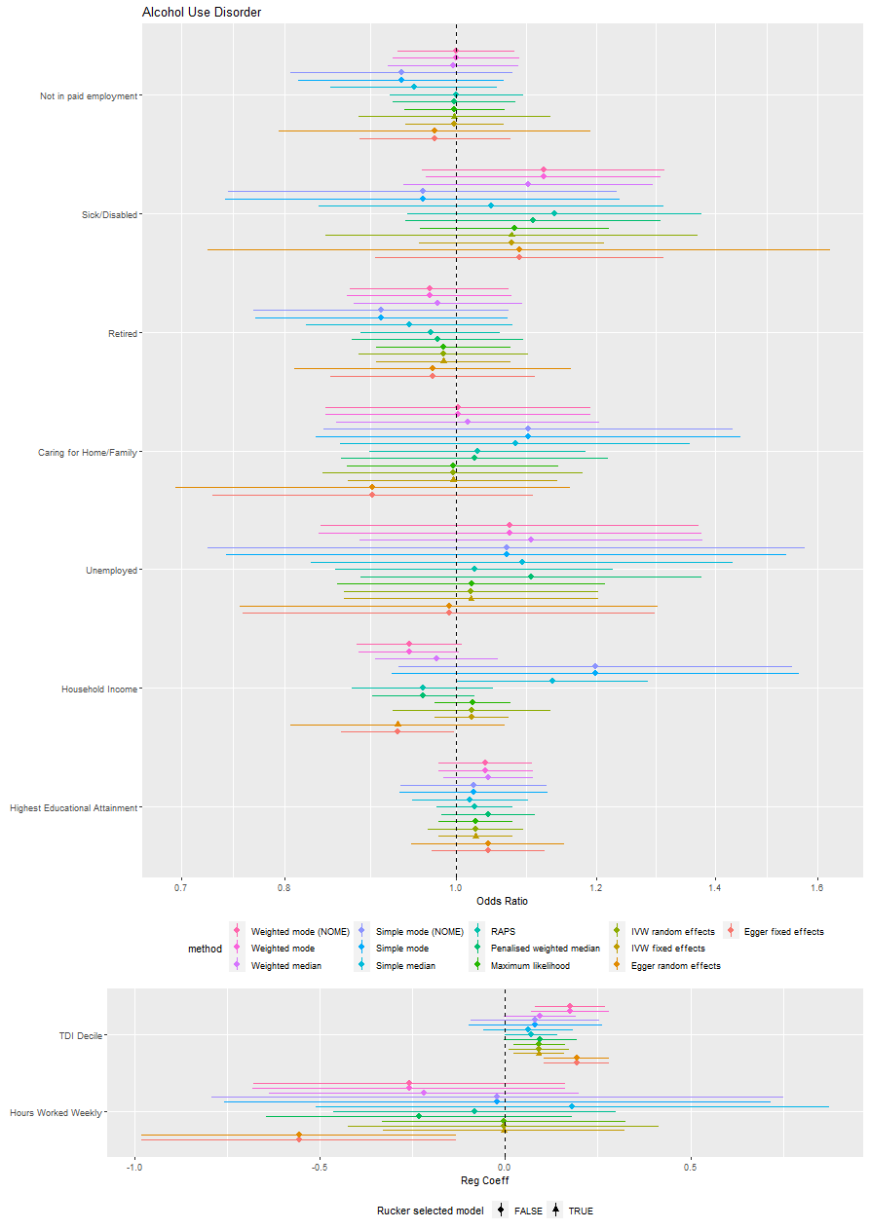


Footnote: Causal effect estimate plus 95% Confidence Interval.

### Figure S12

Figure S12 Forest plots of causal effect estimates of AUD on outcomes, for men.


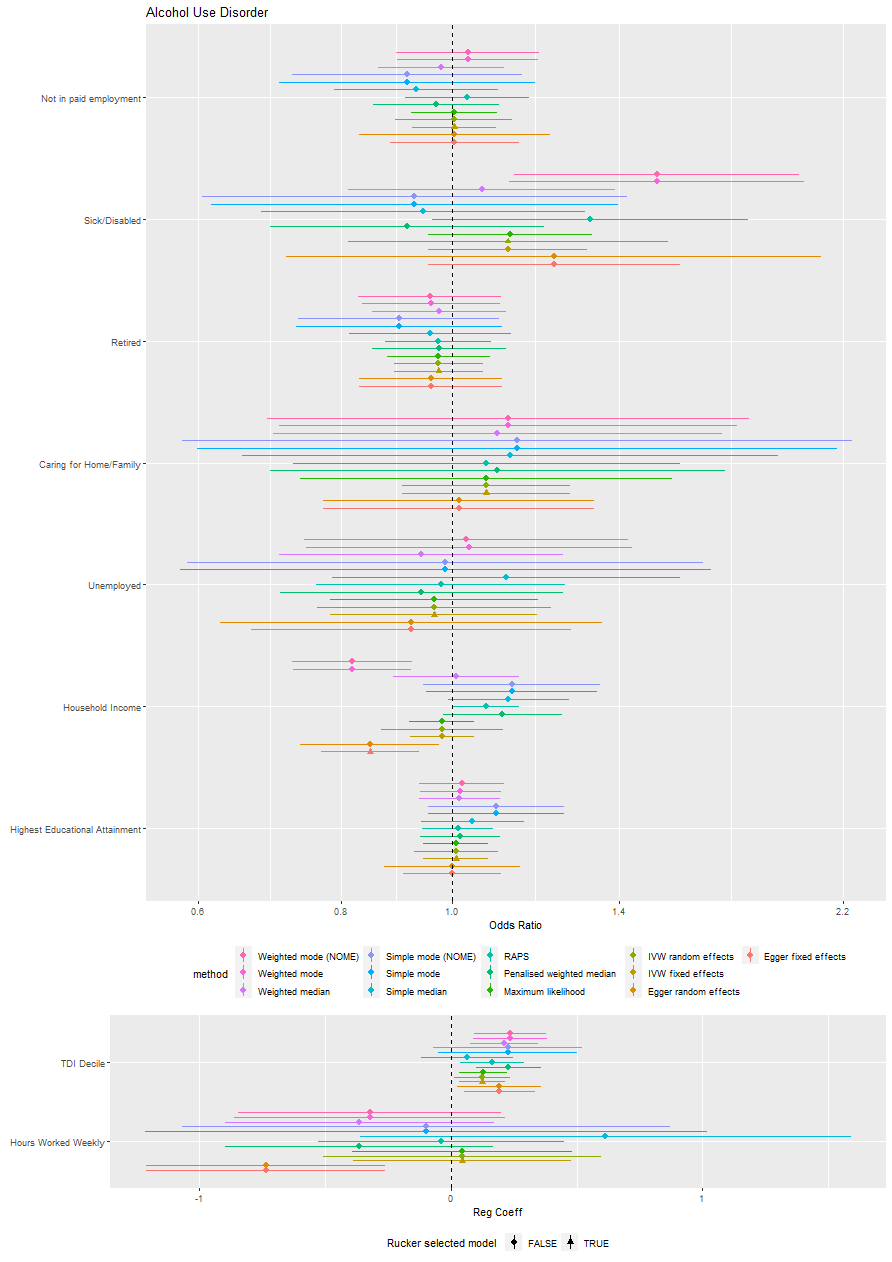


Footnote: Causal effect estimate plus 95% Confidence Interval.

### Figure S13

Figure S13 Forest plots of causal effect estimates of AUD on outcomes, for women.


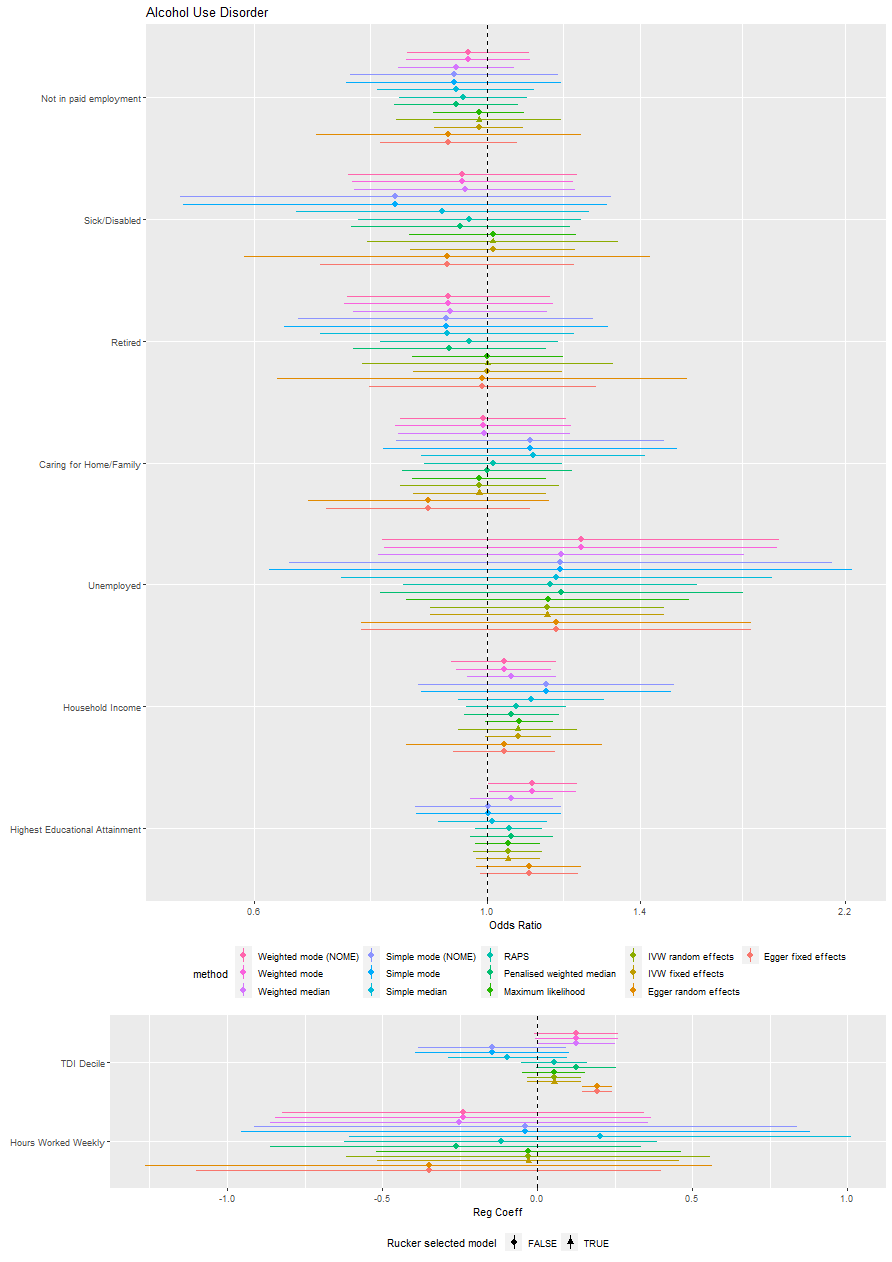


Footnote: Causal effect estimate plus 95% Confidence Interval.

## Plots for specimen exposure-outcome pairing

Figure S14 Scatter plot of SNP - TDI associations, versus SNP - alcohol consumption associations


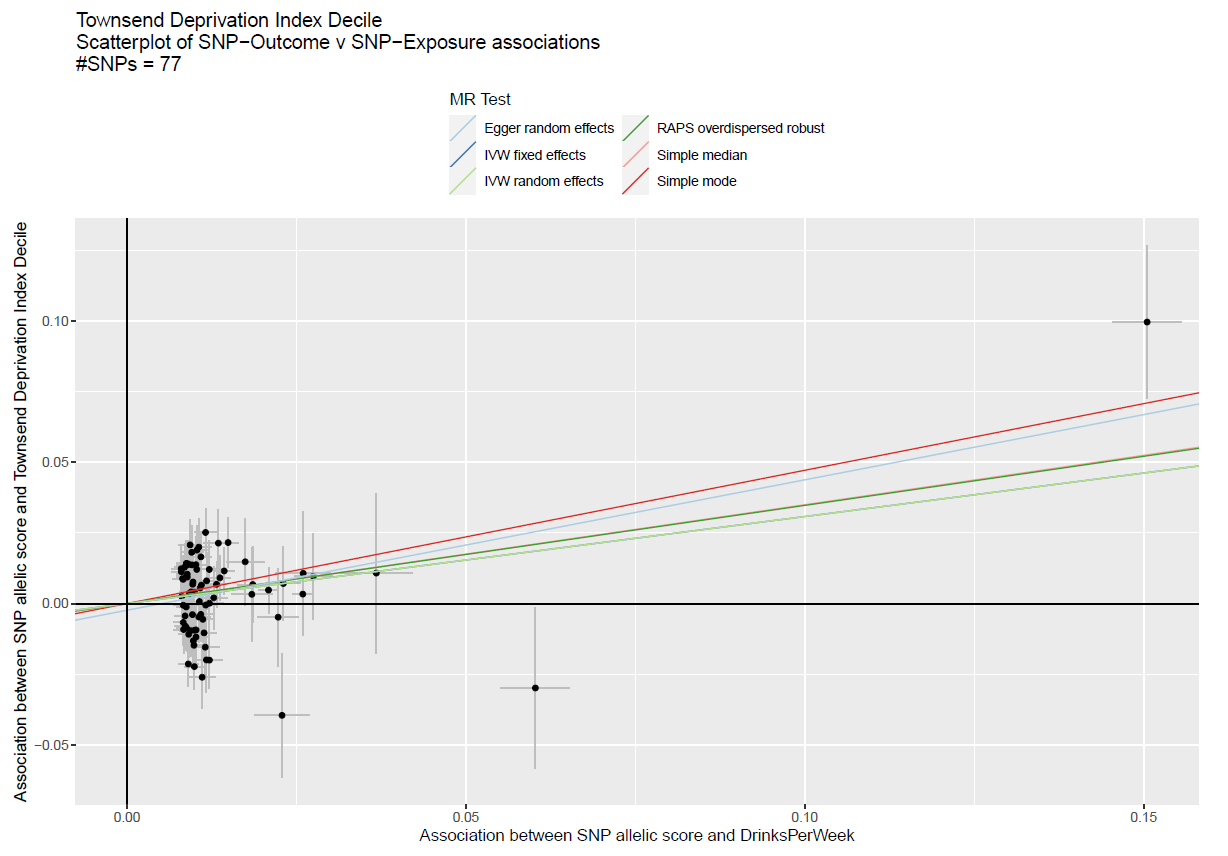


Footnote: X axis – Alcohol consumption - SNP regression coefficient estimates. Y axis – TDI-SNP regression coefficient estimates. Also plotted are the fits for several causal effect estimation methods.

### Figure S15

Figure S15 Quantile-Quantile plots comparing causal effect estimates for alcohol consumption on the TDI outcome against Gaussian distributions.


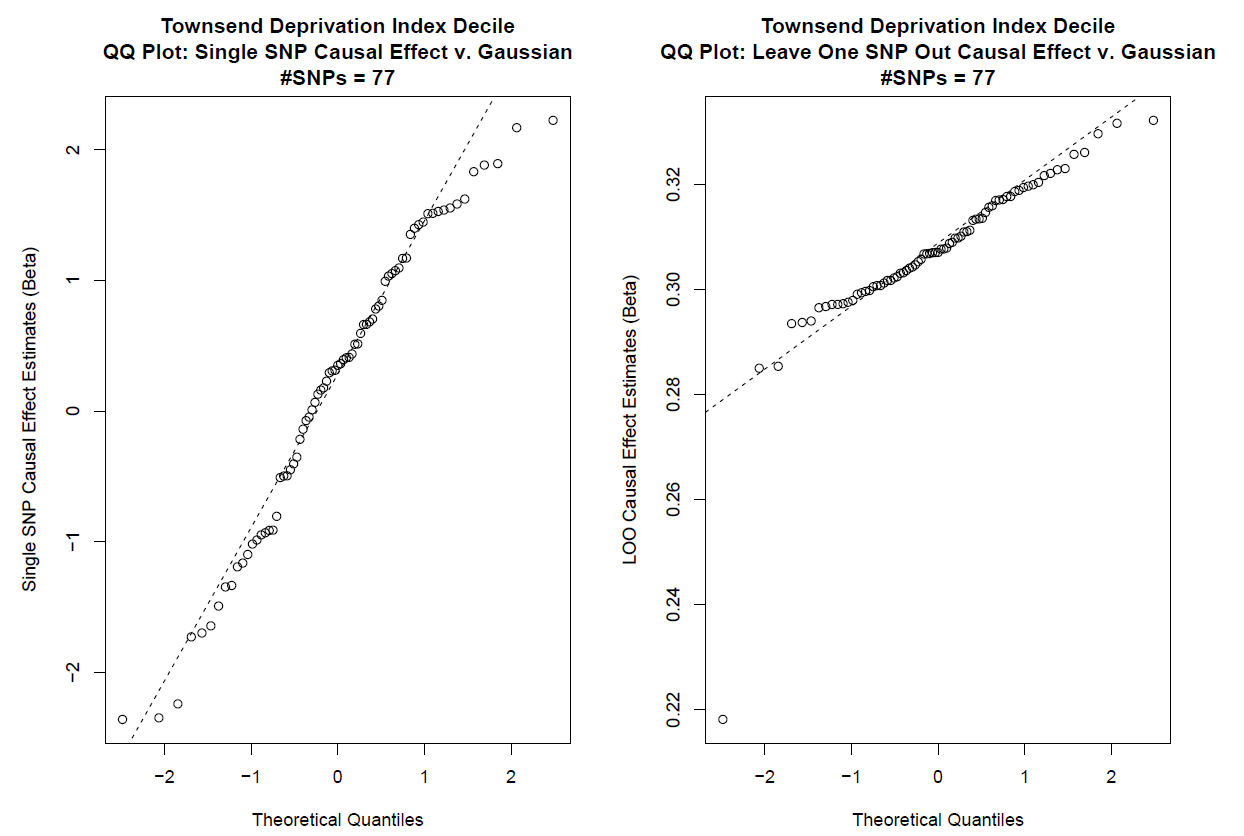


Footnote: Left-hand side - Single SNP analysis. Right-hand side - Leave One SNP Out analysis

### Figure S16

Figure S16 Quantile-Quantile plot comparing SNP contribution to Cochran’s Q to a Chi2 df=1 distribution for Alcohol consumption exposure, TDI outcome.


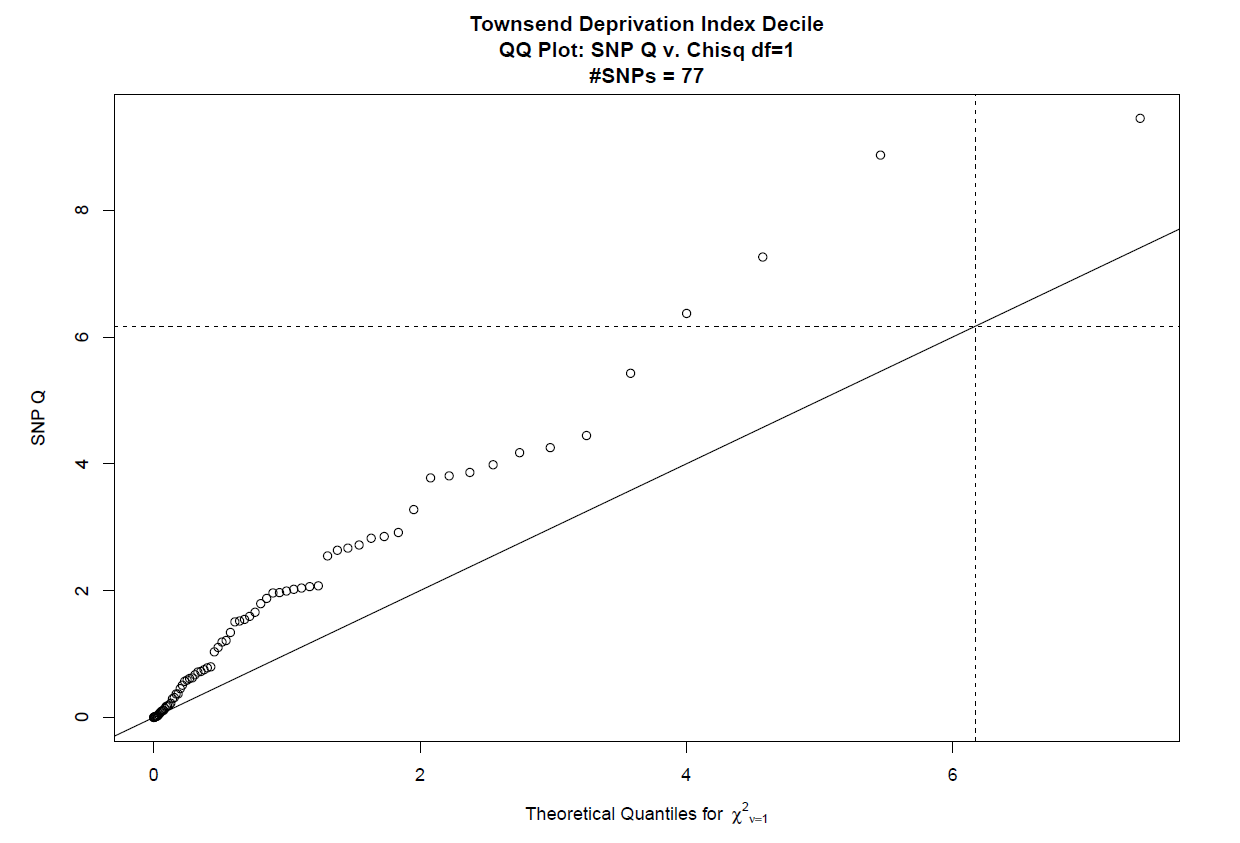


### Figure S17

Figure S17 Rücker model Selection Framework plot for Alcohol consumption exposure, TDI outcome.


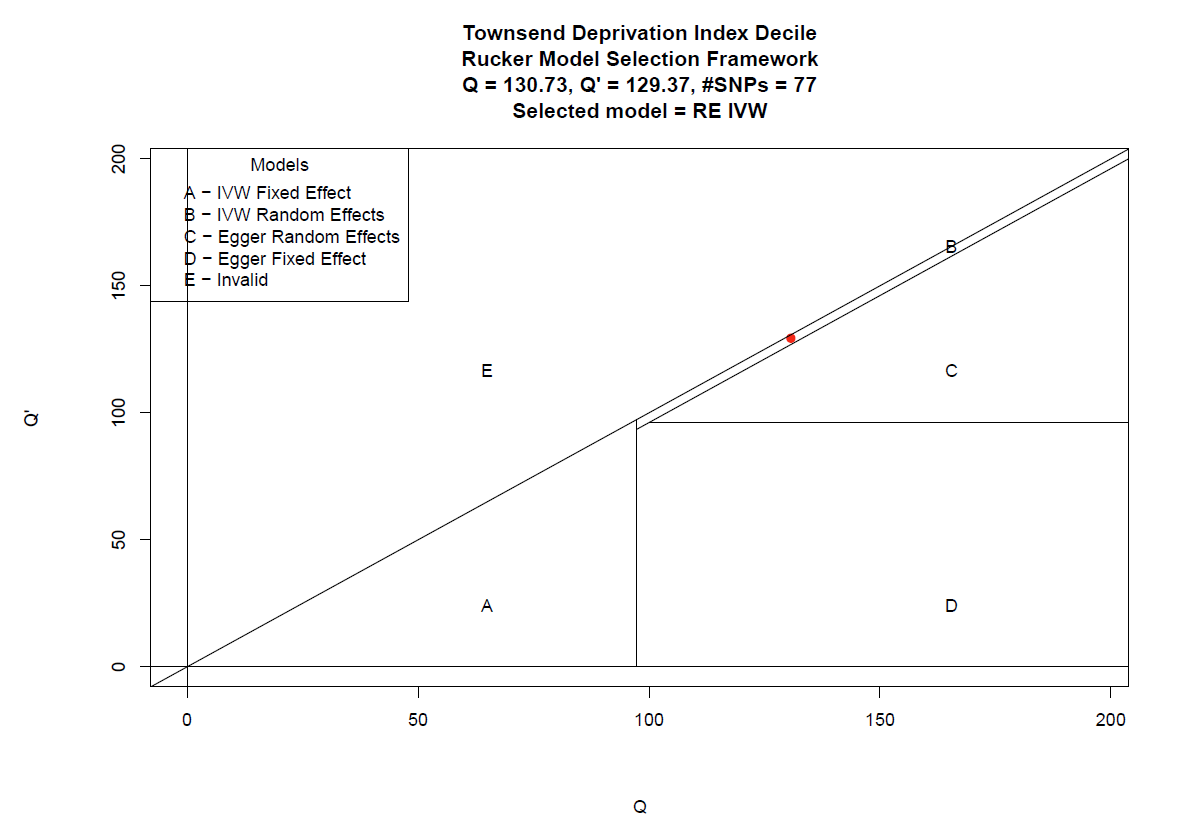


Footnote: The region in which the red dot lies indicates the model selected.

### Figure S18

Figure S18 Distributions of alcohol exposures stratified by sex.


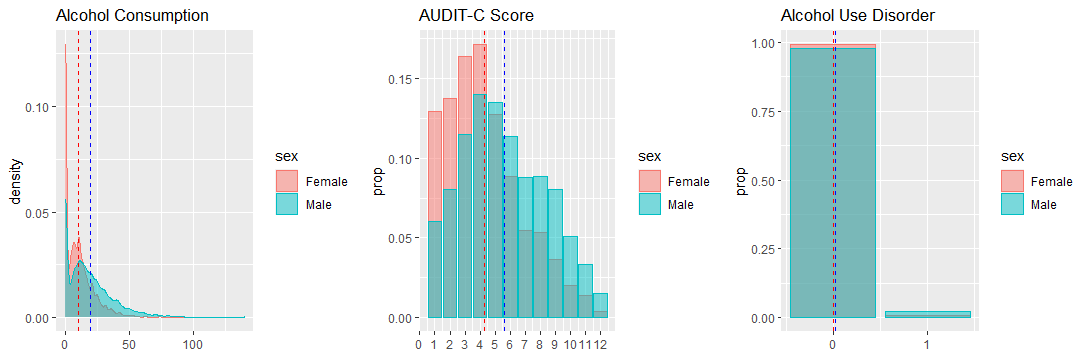


Coloured dashed vertical lines indicate the mean of the alcohol exposure per sex.

## Sample overlap screenshot

Figure S19 Screenshot showing Burgess et al R Shiny app estimate of the expected bias in our alcohol consumption MR analysis.


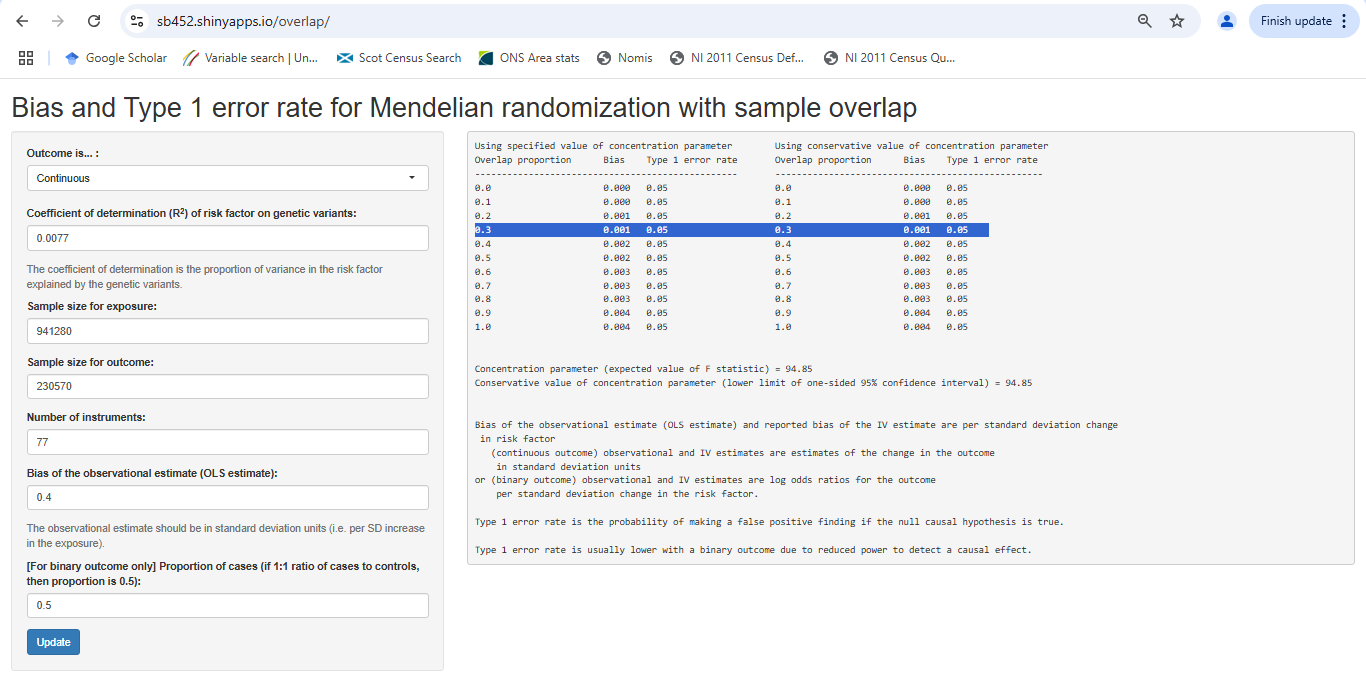


Footnote: The estimates for 30% overlap are highlighted in blue.

# References

BOWDEN, J., SPILLER, W., DEL GRECO, M. F., SHEEHAN, N., THOMPSON, J., MINELLI, C. & DAVEY SMITH, G. 2018. Improving the visualization, interpretation and analysis of two-sample summary data Mendelian randomization via the Radial plot and Radial regression. *Int J Epidemiol,* 47**,** 1264-1278.

BURGESS, S., DAVIES, N. M. & THOMPSON, S. G. 2016. Bias due to participant overlap in two-sample Mendelian randomization. *Genet Epidemiol,* 40**,** 597-608.

CHANG, C. C., CHOW, C. C., TELLIER, L. C., VATTIKUTI, S., PURCELL, S. M. & LEE, J. J. 2015. Second-generation PLINK: rising to the challenge of larger and richer datasets. *Gigascience,* 4**,** 7.

DAVIS, K. A. S., COLEMAN, J. R. I., ADAMS, M., ALLEN, N., BREEN, G., CULLEN, B., DICKENS, C., FOX, E., GRAHAM, N., HOLLIDAY, J., HOWARD, L. M., JOHN, A., LEE, W., MCCABE, R., MCINTOSH, A., PEARSALL, R., SMITH, D. J., SUDLOW, C., WARD, J., ZAMMIT, S. & HOTOPF, M. 2020. Mental health in UK Biobank - development, implementation and results from an online questionnaire completed by 157 366 participants: a reanalysis. *BJPsych Open,* 6**,** e18.

HEMANI, G., ZHENG, J., ELSWORTH, B., WADE, K. H., HABERLAND, V., BAIRD, D., LAURIN, C., BURGESS, S., BOWDEN, J., LANGDON, R., TAN, V. Y., YARMOLINSKY, J., SHIHAB, H. A., TIMPSON, N. J., EVANS, D. M., RELTON, C., MARTIN, R. M., DAVEY SMITH, G., GAUNT, T. R. & HAYCOCK, P. C. 2018. The MR-Base platform supports systematic causal inference across the human phenome. *Elife,* 7.

PEDUZZI, P., CONCATO, J., KEMPER, E., HOLFORD, T. R. & FEINSTEIN, A. R. 1996. A simulation study of the number of events per variable in logistic regression analysis. *Journal of Clinical Epidemiology,* 49**,** 1373-1379.

RANGANATHAN, P., PRAMESH, C. S. & AGGARWAL, R. Common pitfalls in statistical analysis: Logistic regression.

RUCKER, G., SCHWARZER, G., CARPENTER, J. R., BINDER, H. & SCHUMACHER, M. 2011. Treatment-effect estimates adjusted for small-study effects via a limit meta-analysis. *Biostatistics,* 12**,** 122-42.

WILLER, C. J., LI, Y. & ABECASIS, G. R. 2010. METAL: fast and efficient meta-analysis of genomewide association scans. *Bioinformatics,* 26**,** 2190-1.
