# Supplementary material for: Effects of alcohol consumption on employment and social outcomes: a Mendelian randomisation study
Source: Alcohol Alcohol. 2025 Jul 18;60(5):agaf038. doi: 10.1093/alcalc/agaf038 (PMC12271571; doi:10.1093/alcalc/agaf038)
Supplement: Campbell_Green_Davies_et_al_2025_agaf038 [file campbell_green_davies_et_al_2025_agaf038.zip › Campbell_Green_Davies_et_al_2025/All/aud/do2SampleMrAnalyses_bAlcoholUseDisorder_householdIncome_ageSexNinHouseCentreGpc.pdf]

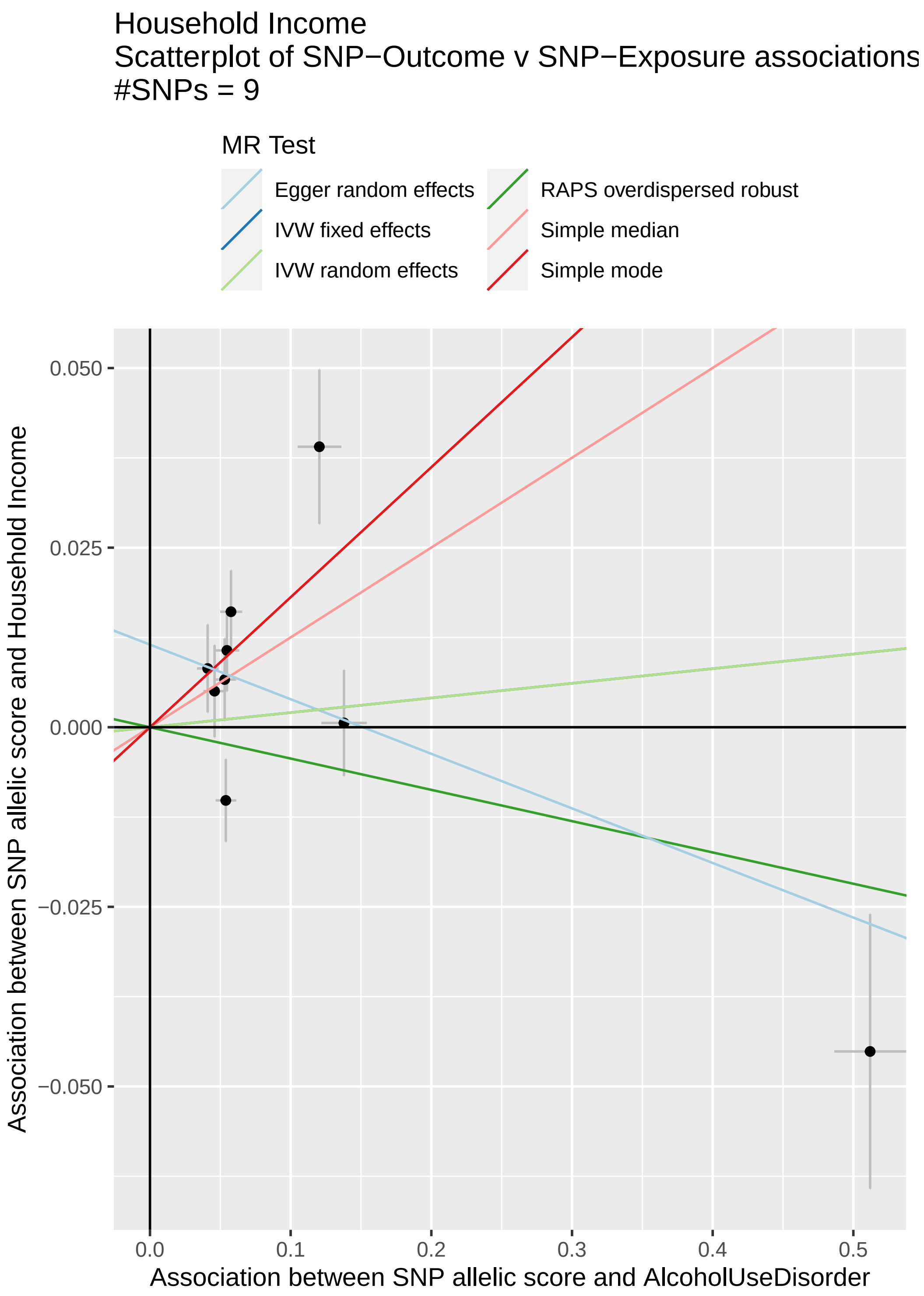
Household Income
Scatterplot of SNP−Outcome v SNP−Exposure associations
#SNPs = 9
MR Test
Egger random effects
IVW fixed effects
IVW random effects
RAPS overdispersed robust
Simple median
Simple mode
0.050
0.025
0.000
−0.025
−0.050
0.0
0.1
0.2
0.3
0.4
0.5
Association between SNP allelic score and Household Income
Association between SNP allelic score and AlcoholUseDisorder

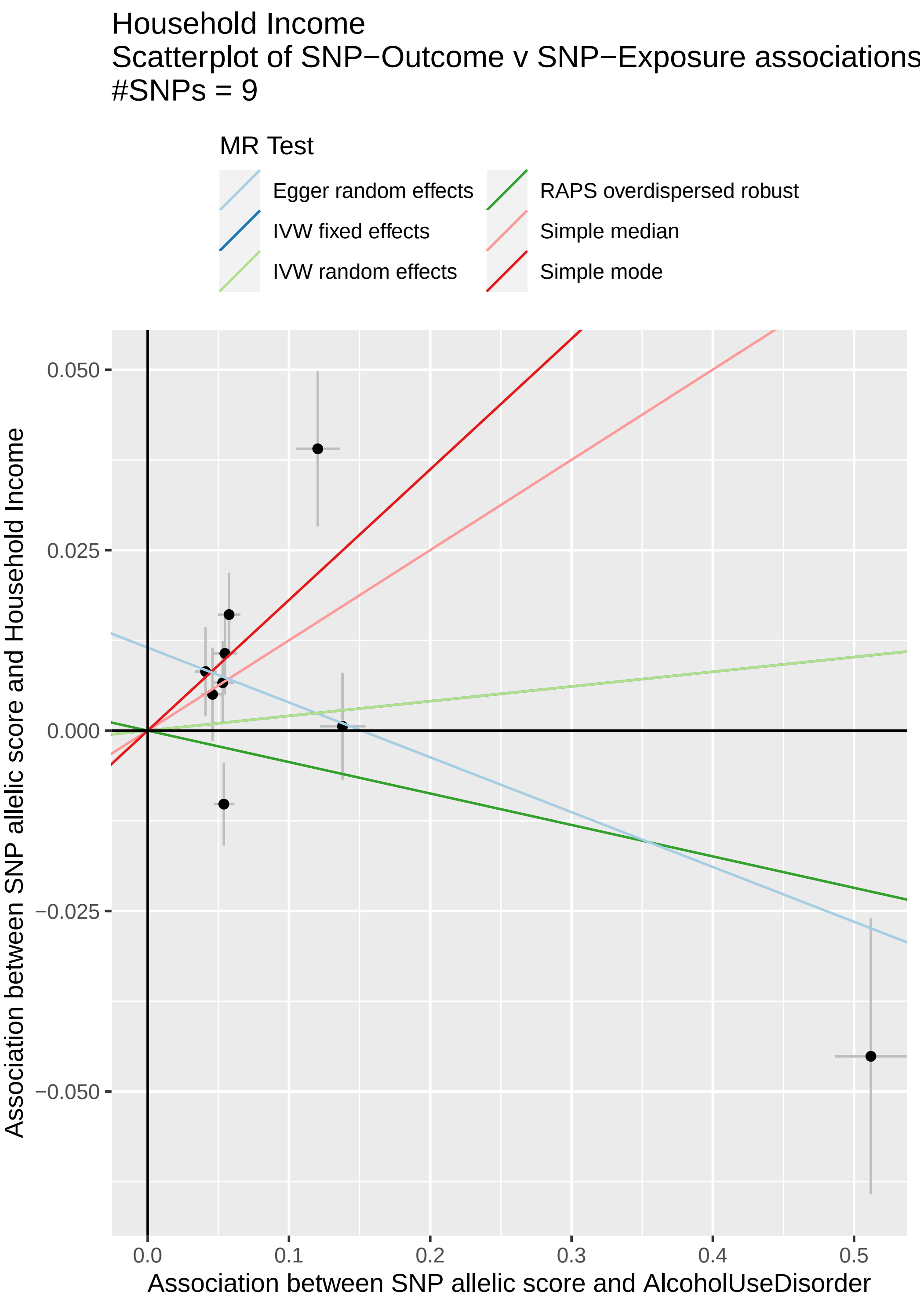
Household Income
Scatterplot of SNP−Outcome v SNP−Exposure associations
#SNPs = 9
MR Test
Egger random effects
IVW fixed effects
IVW random effects
RAPS overdispersed robust
Simple median
Simple mode
0.050
0.025
0.000
−0.025
−0.050
0.0
0.1
0.2
0.3
0.4
0.5
Association between SNP allelic score and Household Income
Association between SNP allelic score and AlcoholUseDisorder

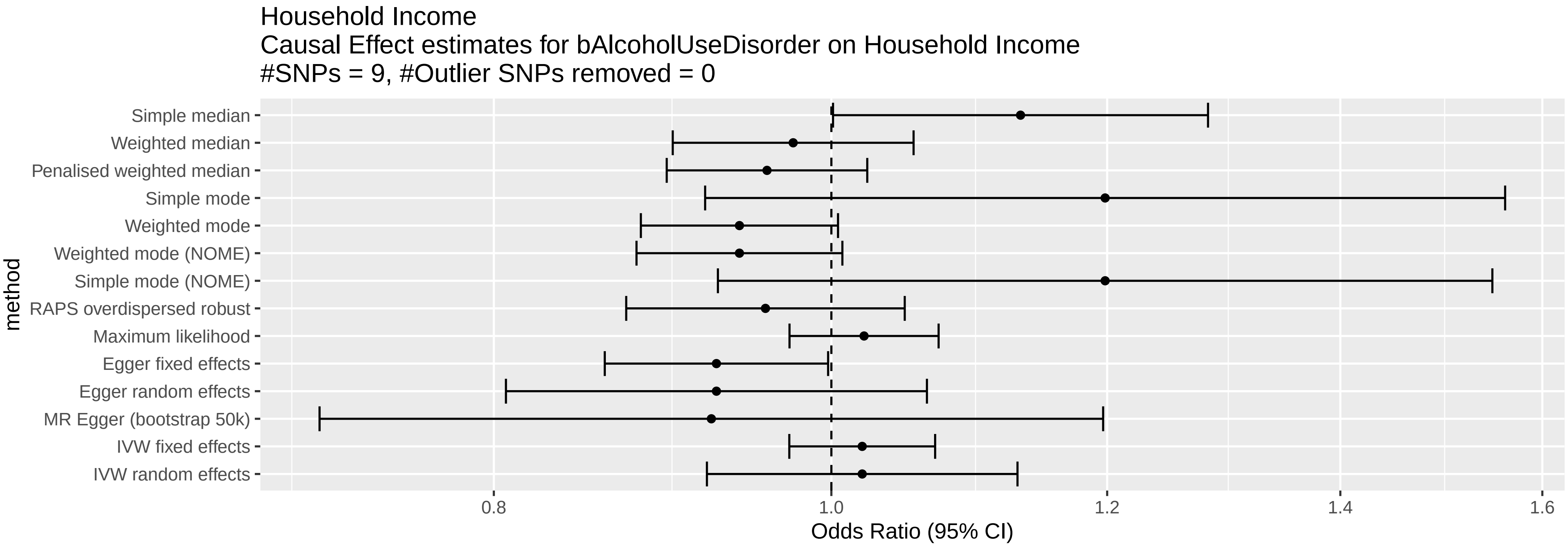
Household Income
Causal Effect estimates for bAlcoholUseDisorder on Household Income
#SNPs = 9, #Outlier SNPs removed = 0
Simple median
Weighted median
Penalised weighted median
Simple mode
Weighted mode
Weighted mode (NOME)
Simple mode (NOME)
RAPS overdispersed robust
Maximum likelihood
Egger fixed effects
Egger random effects
MR Egger (bootstrap 50k)
IVW fixed effects
IVW random effects
method
0.8
1.0
1.2
1.4
1.6
Odds Ratio (95% CI)

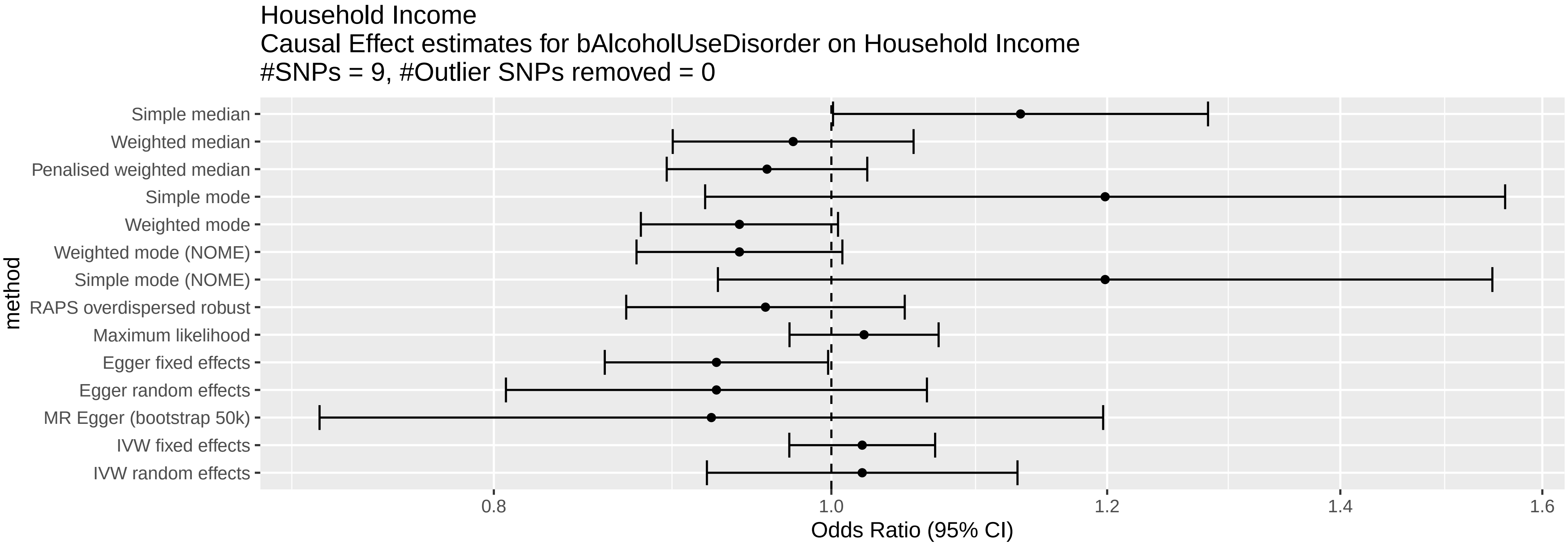
Household Income
Causal Effect estimates for bAlcoholUseDisorder on Household Income
#SNPs = 9, #Outlier SNPs removed = 0
Simple median
Weighted median
Penalised weighted median
Simple mode
Weighted mode
Weighted mode (NOME)
Simple mode (NOME)
RAPS overdispersed robust
Maximum likelihood
Egger fixed effects
Egger random effects
MR Egger (bootstrap 50k)
IVW fixed effects
IVW random effects
method
0.8
1.0
1.2
1.4
1.6
Odds Ratio (95% CI)

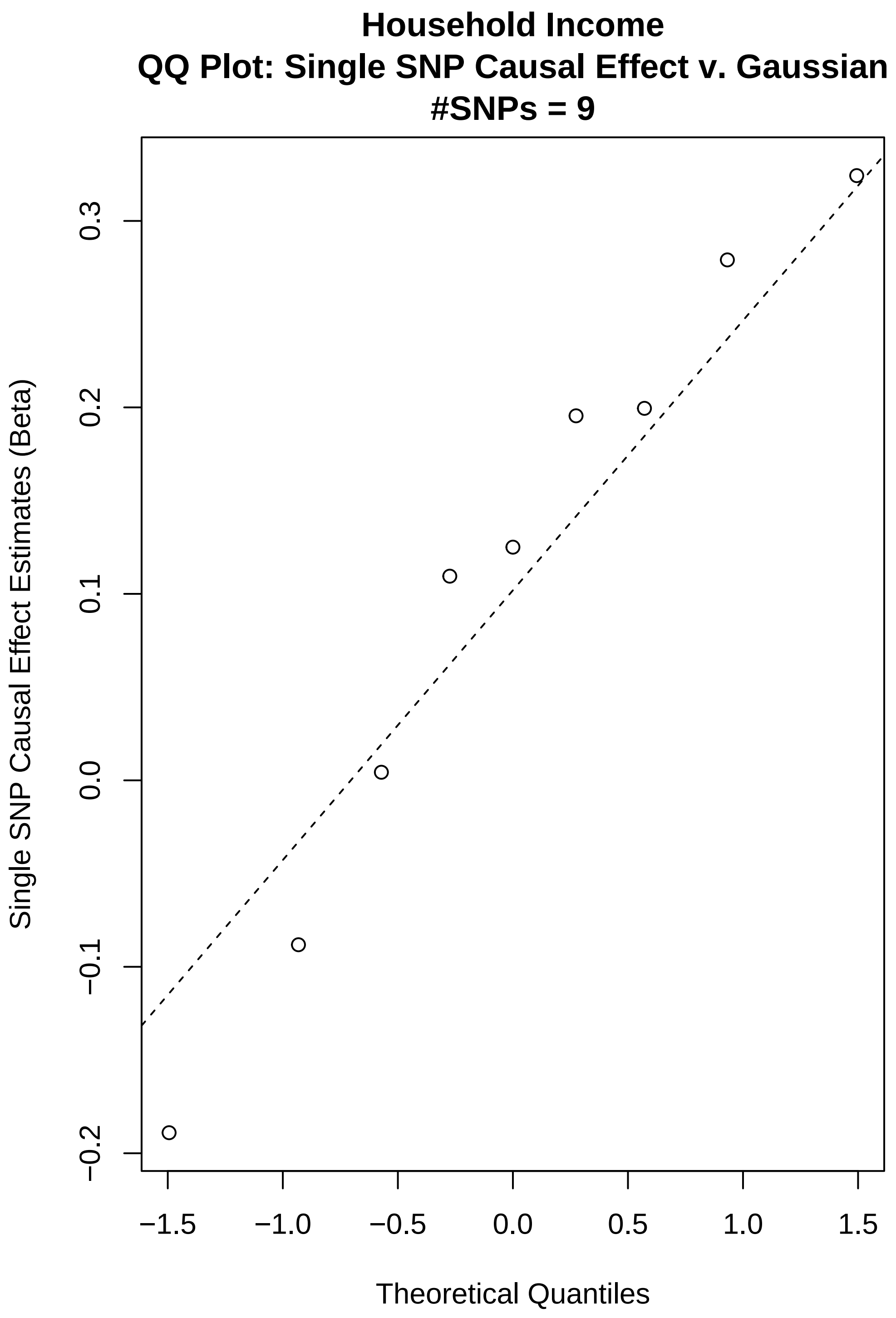
Household Income
QQ Plot: Single SNP Causal Effect v. Gaussian
#SNPs = 9
Single SNP Causal Effect Estimates (Beta)
Theoretical Quantiles
−0.2
−0.1
0.0
0.1
0.2
0.3
−1.5
−1.0
−0.5
0.0
0.5
1.0
1.5

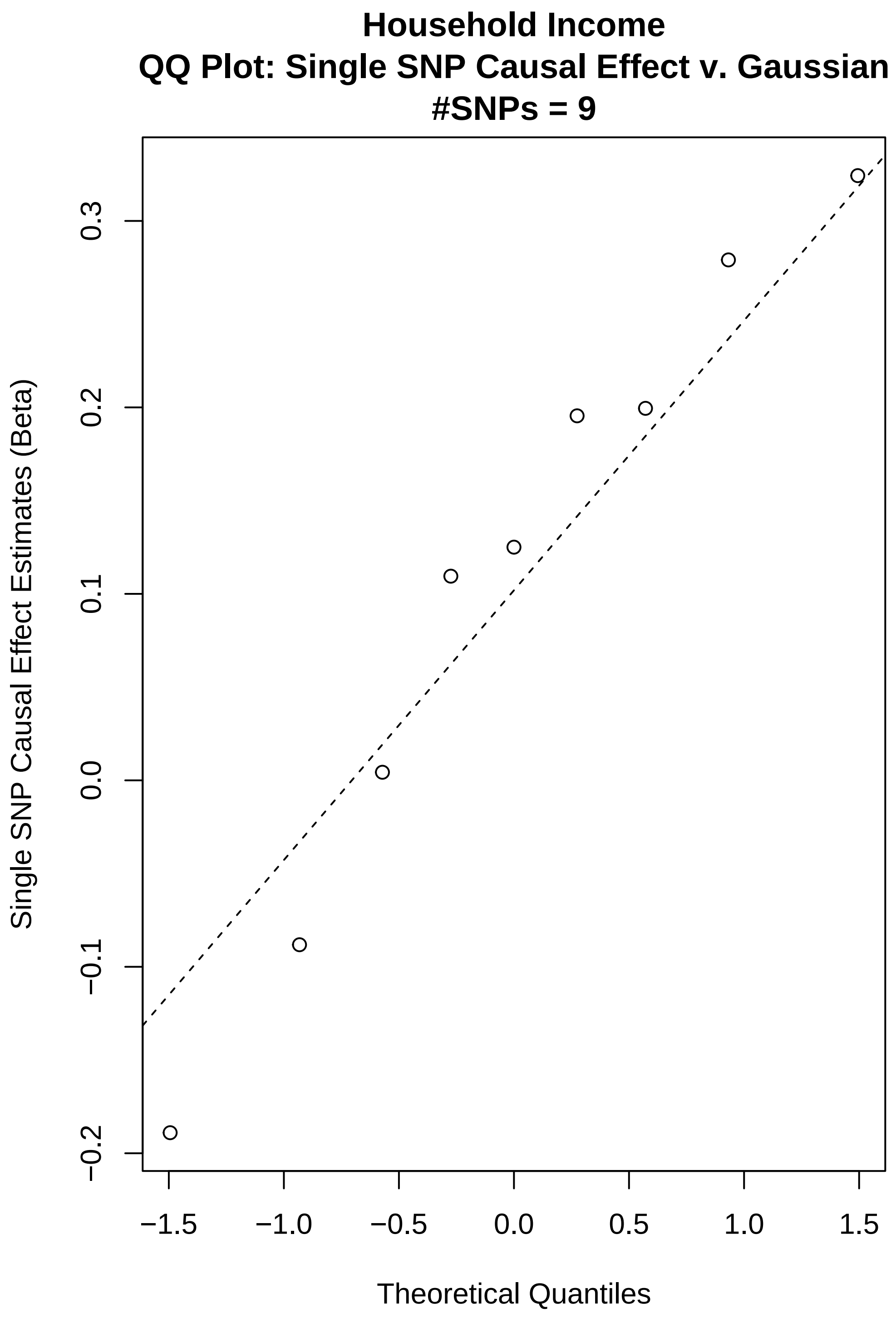
Household Income
QQ Plot: Single SNP Causal Effect v. Gaussian
#SNPs = 9
Single SNP Causal Effect Estimates (Beta)
Theoretical Quantiles
−0.2
−0.1
0.0
0.1
0.2
0.3
−1.5
−1.0
−0.5
0.0
0.5
1.0
1.5

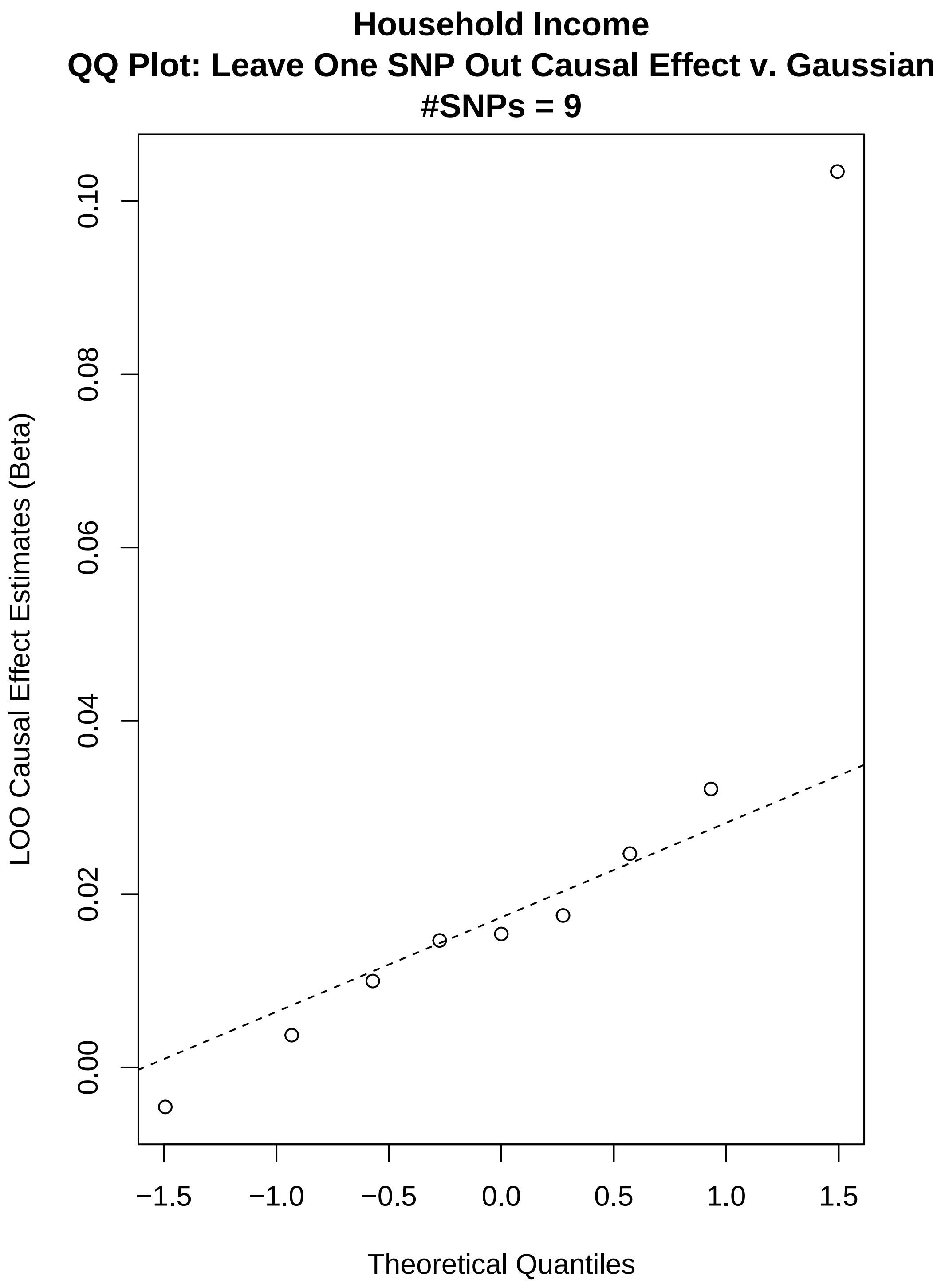
Household Income
QQ Plot: Leave One SNP Out Causal Effect v. Gaussian
#SNPs = 9
LOO Causal Effect Estimates (Beta)
0.00
0.02
0.04
0.06
0.08
0.10
−1.5
−1.0
−0.5
0.0
0.5
1.0
1.5
Theoretical Quantiles

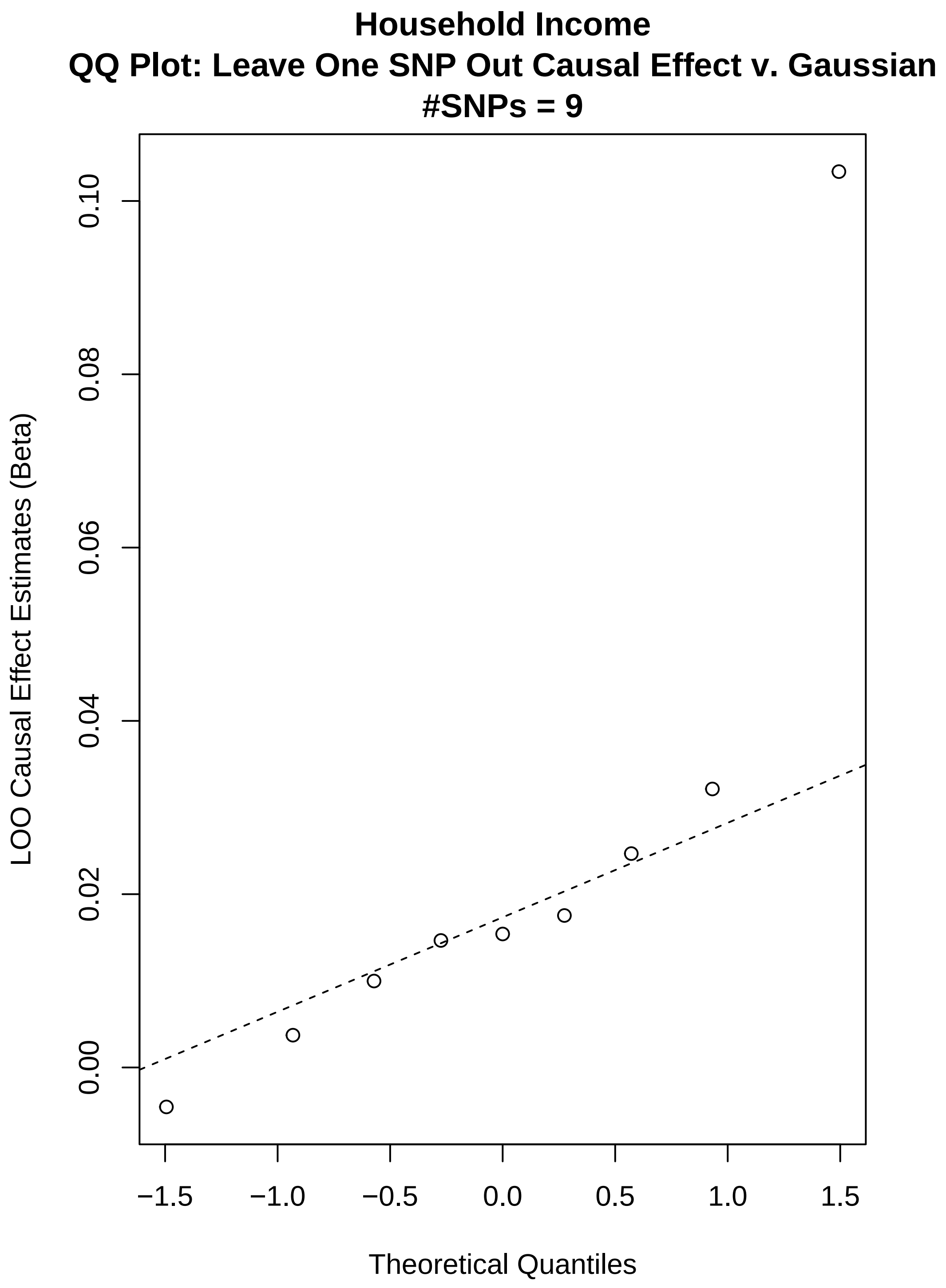
Household Income
QQ Plot: Leave One SNP Out Causal Effect v. Gaussian
#SNPs = 9
LOO Causal Effect Estimates (Beta)
0.00
0.02
0.04
0.06
0.08
0.10
−1.5
−1.0
−0.5
0.0
0.5
1.0
1.5
Theoretical Quantiles

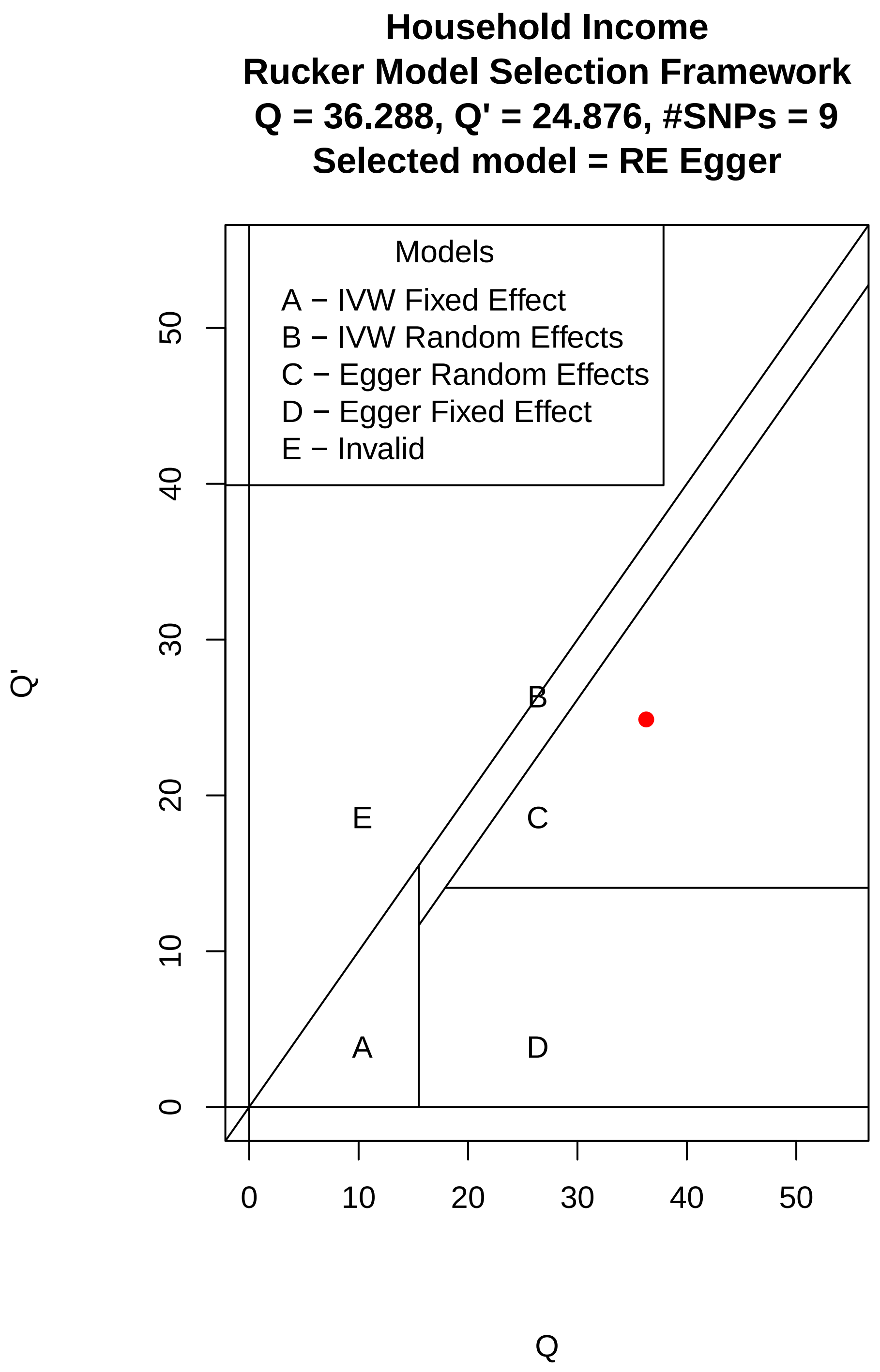
Household Income
Rucker Model Selection Framework
Q = 36.288, Q' = 24.876, #SNPs = 9
Selected model = RE Egger
Models
A − IVW Fixed Effect
B − IVW Random Effects
C − Egger Random Effects
D − Egger Fixed Effect
E − Invalid
A
B
C
D
E
Q
Q'
0
10
20
30
40
50

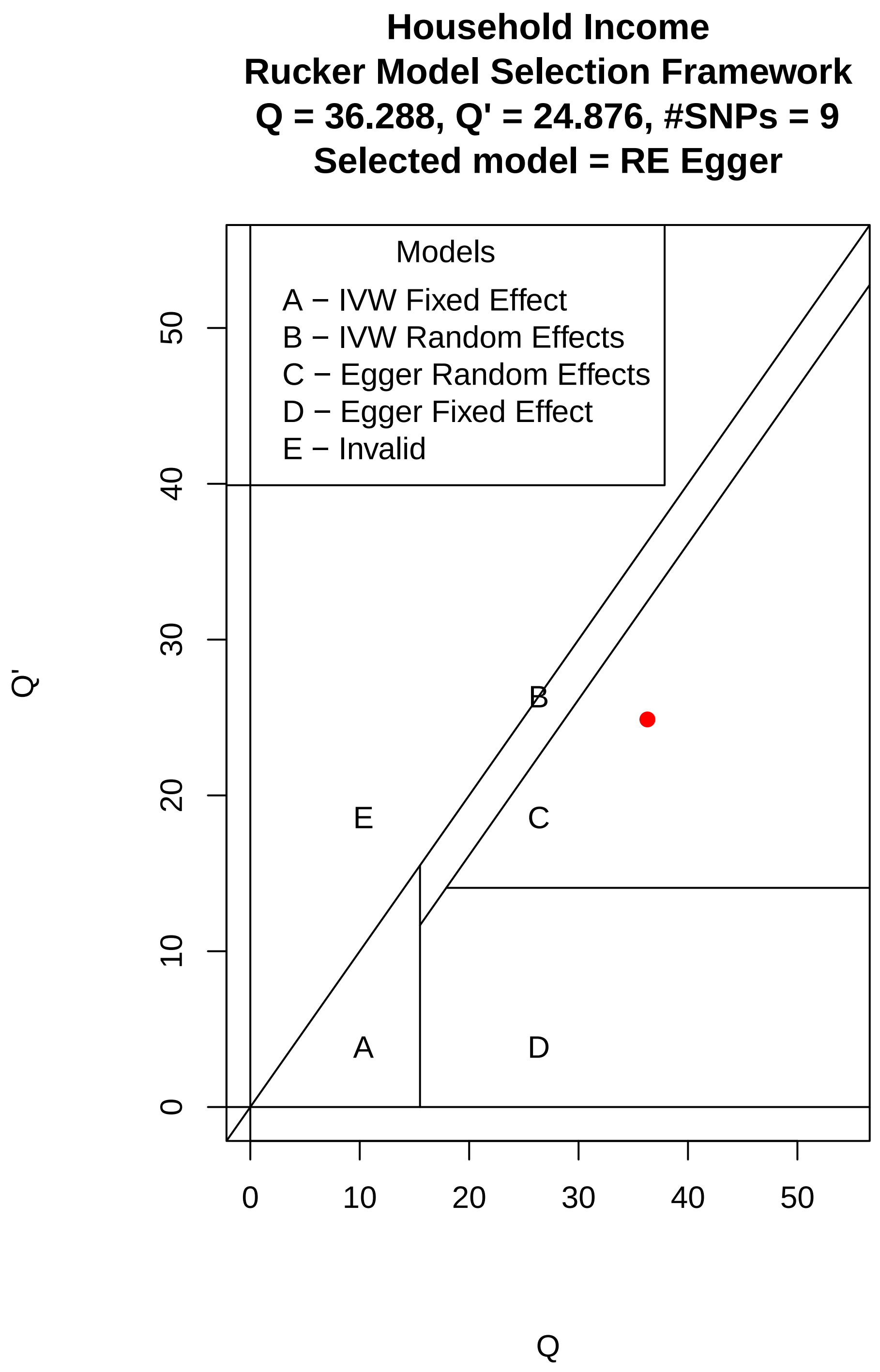
Household Income
Rucker Model Selection Framework
Q = 36.288, Q' = 24.876, #SNPs = 9
Selected model = RE Egger
Models
A − IVW Fixed Effect
B − IVW Random Effects
C − Egger Random Effects
D − Egger Fixed Effect
E − Invalid
A
B
C
D
E
Q
Q'
0
10
20
30
40
50

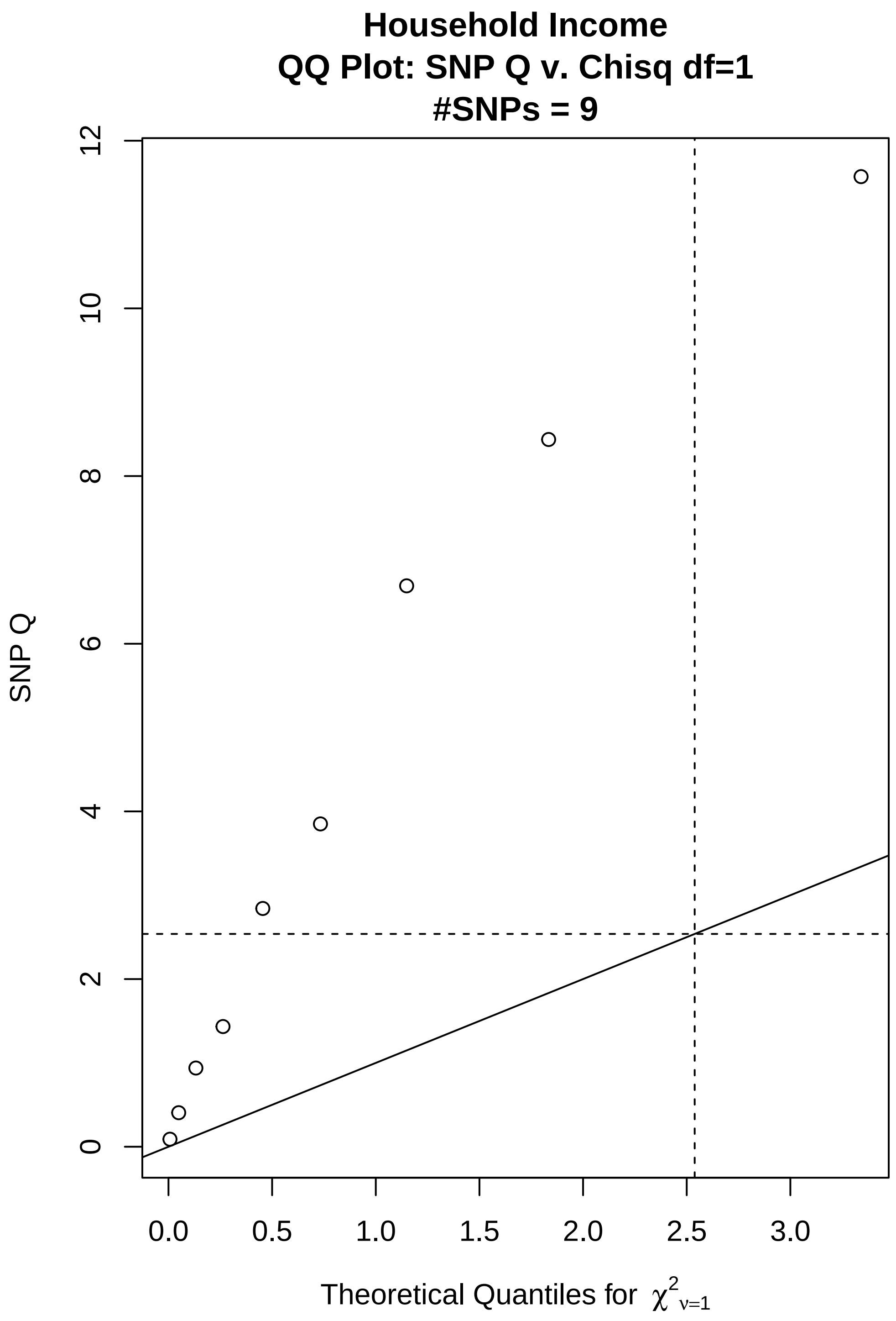
Household Income
QQ Plot: SNP Q v. Chisq df=1
#SNPs = 9
SNP Q
0
2
4
6
8
10
12
0.0
0.5
1.0
1.5
2.0
2.5
3.0
Theoretical Quantiles for $\chi^2_{\nu=1}$

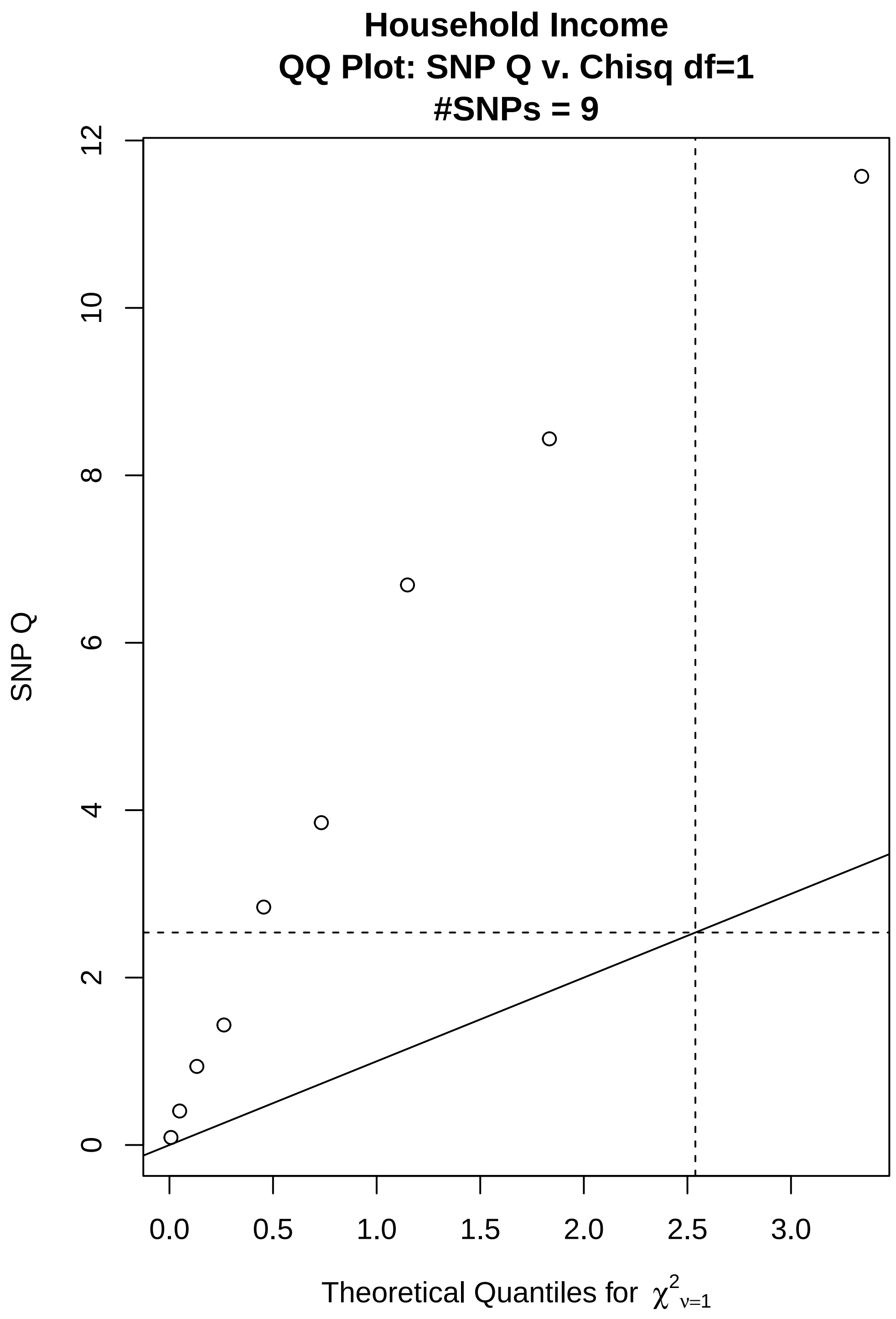
Household Income
QQ Plot: SNP Q v. Chisq df=1
#SNPs = 9
SNP Q
0
2
4
6
8
10
12
0.0
0.5
1.0
1.5
2.0
2.5
3.0
Theoretical Quantiles for $\chi^2_{\nu=1}$
