# Supplementary material for: Effects of alcohol consumption on employment and social outcomes: a Mendelian randomisation study
Source: Alcohol Alcohol. 2025 Jul 18;60(5):agaf038. doi: 10.1093/alcalc/agaf038 (PMC12271571; doi:10.1093/alcalc/agaf038)
Supplement: Campbell_Green_Davies_et_al_2025_agaf038 [file campbell_green_davies_et_al_2025_agaf038.zip › Campbell_Green_Davies_et_al_2025/All/aud/do2SampleMrAnalyses_bAlcoholUseDisorder_iFamilyNotEmp_ageSexCentreGpc.pdf]

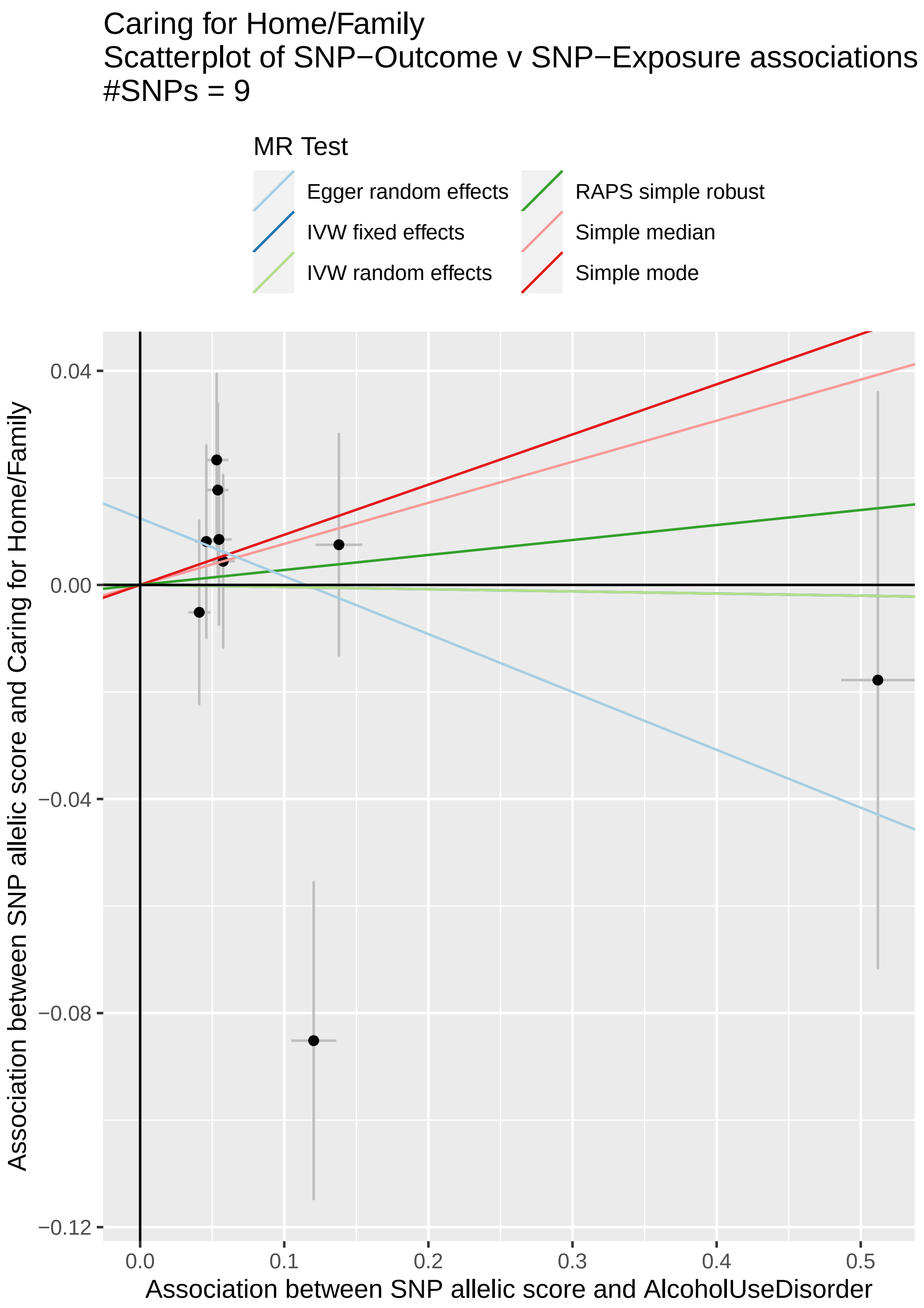
Caring for Home/Family
Scatterplot of SNP−Outcome v SNP−Exposure associations
#SNPs = 9
MR Test
Egger random effects
IVW fixed effects
IVW random effects
RAPS simple robust
Simple median
Simple mode
0.04
0.00
−0.04
−0.08
−0.12
0.0
0.1
0.2
0.3
0.4
0.5
Association between SNP allelic score and Caring for Home/Family
Association between SNP allelic score and AlcoholUseDisorder

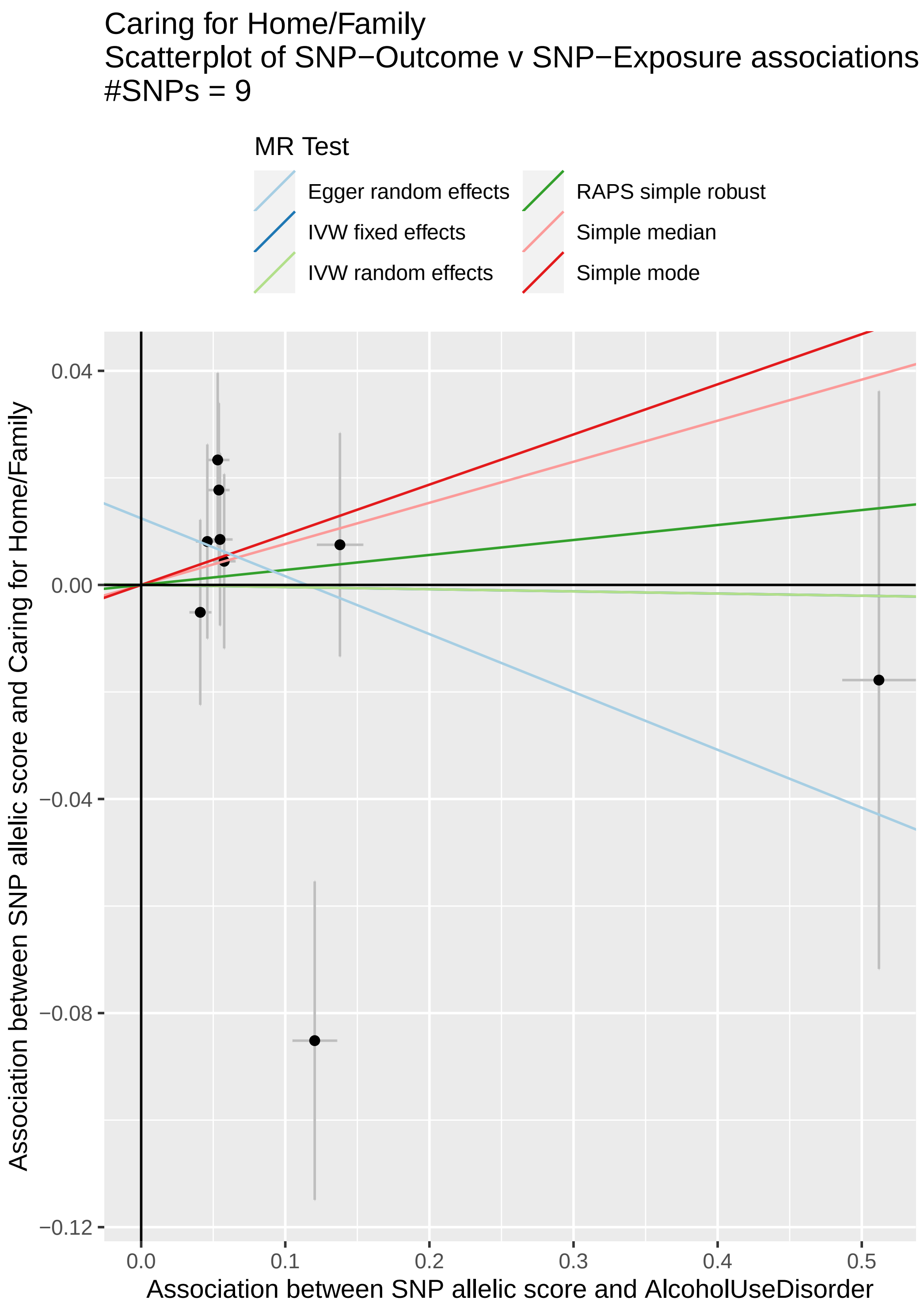
Caring for Home/Family
Scatterplot of SNP−Outcome v SNP−Exposure associations
#SNPs = 9
MR Test
Egger random effects
IVW fixed effects
IVW random effects
RAPS simple robust
Simple median
Simple mode
0.04
0.00
−0.04
−0.08
−0.12
0.0
0.1
0.2
0.3
0.4
0.5
Association between SNP allelic score and Caring for Home/Family
Association between SNP allelic score and AlcoholUseDisorder

Caring for Home/Family
Causal Effect estimates for bAlcoholUseDisorder on Caring for Home/Family
#SNPs = 9, #Outlier SNPs removed = 0

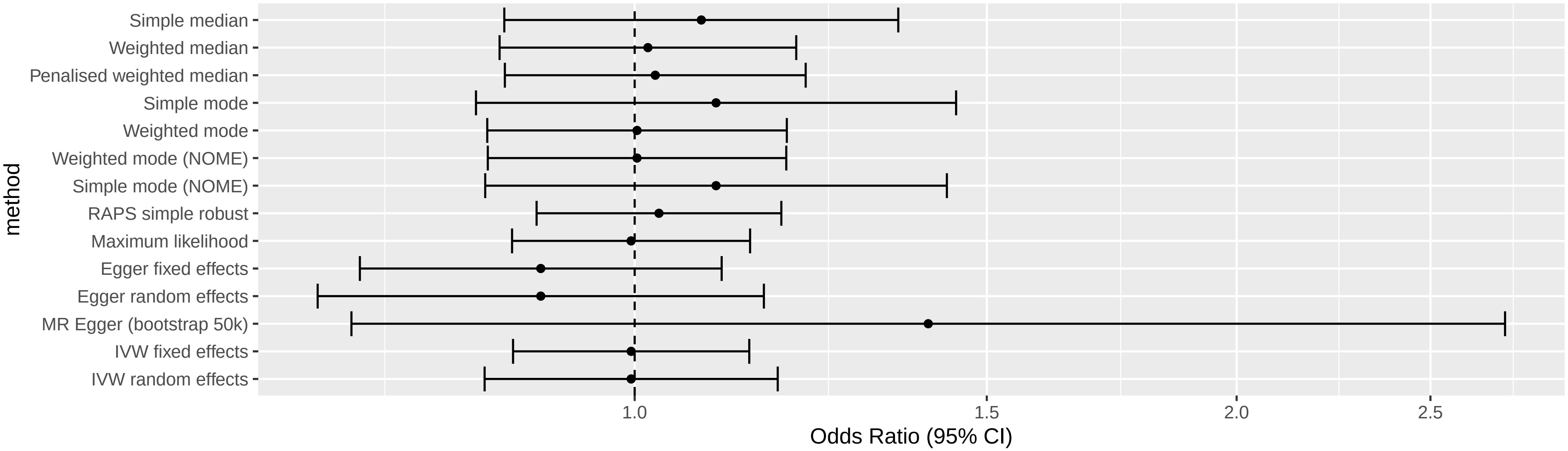

Caring for Home/Family
Causal Effect estimates for bAlcoholUseDisorder on Caring for Home/Family
#SNPs = 9, #Outlier SNPs removed = 0

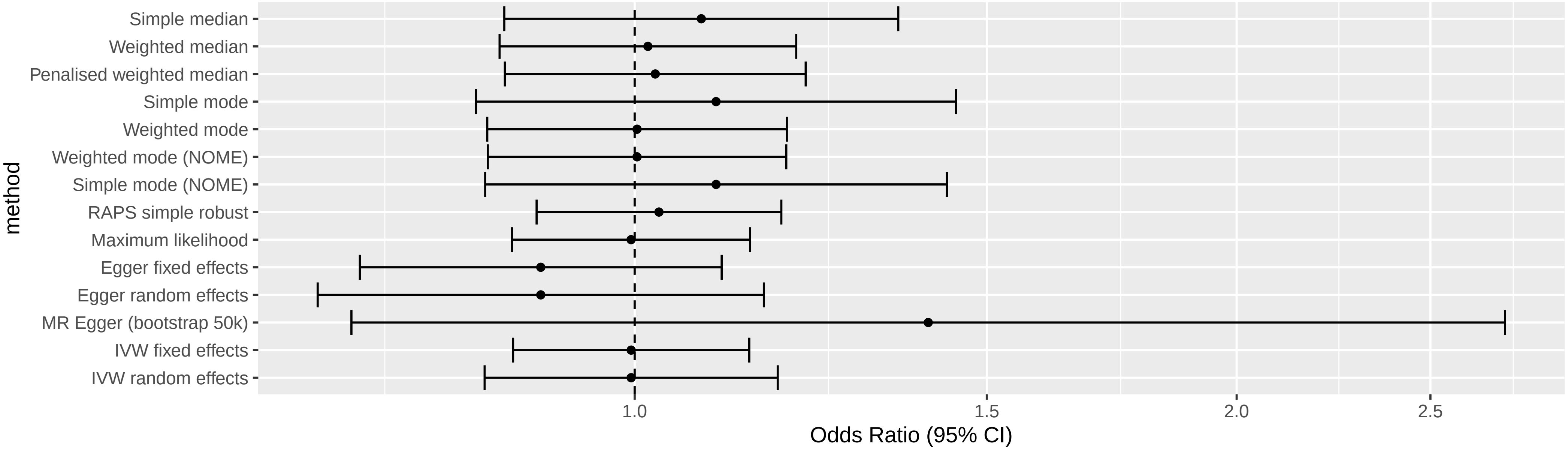

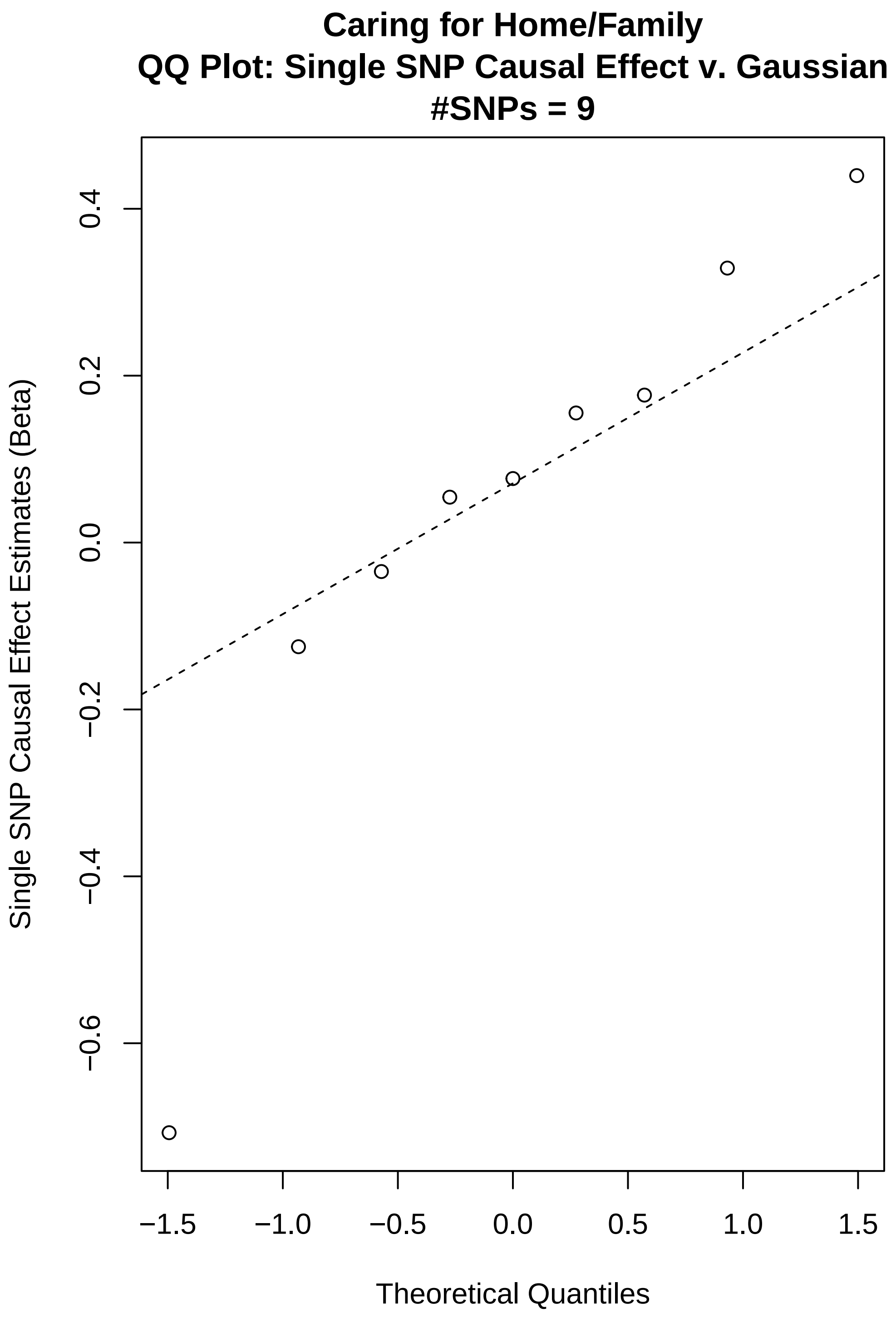
Caring for Home/Family
QQ Plot: Single SNP Causal Effect v. Gaussian
#SNPs = 9
Single SNP Causal Effect Estimates (Beta)
Theoretical Quantiles
−0.6
−0.4
−0.2
0.0
0.2
0.4
−1.5
−1.0
−0.5
0.0
0.5
1.0
1.5

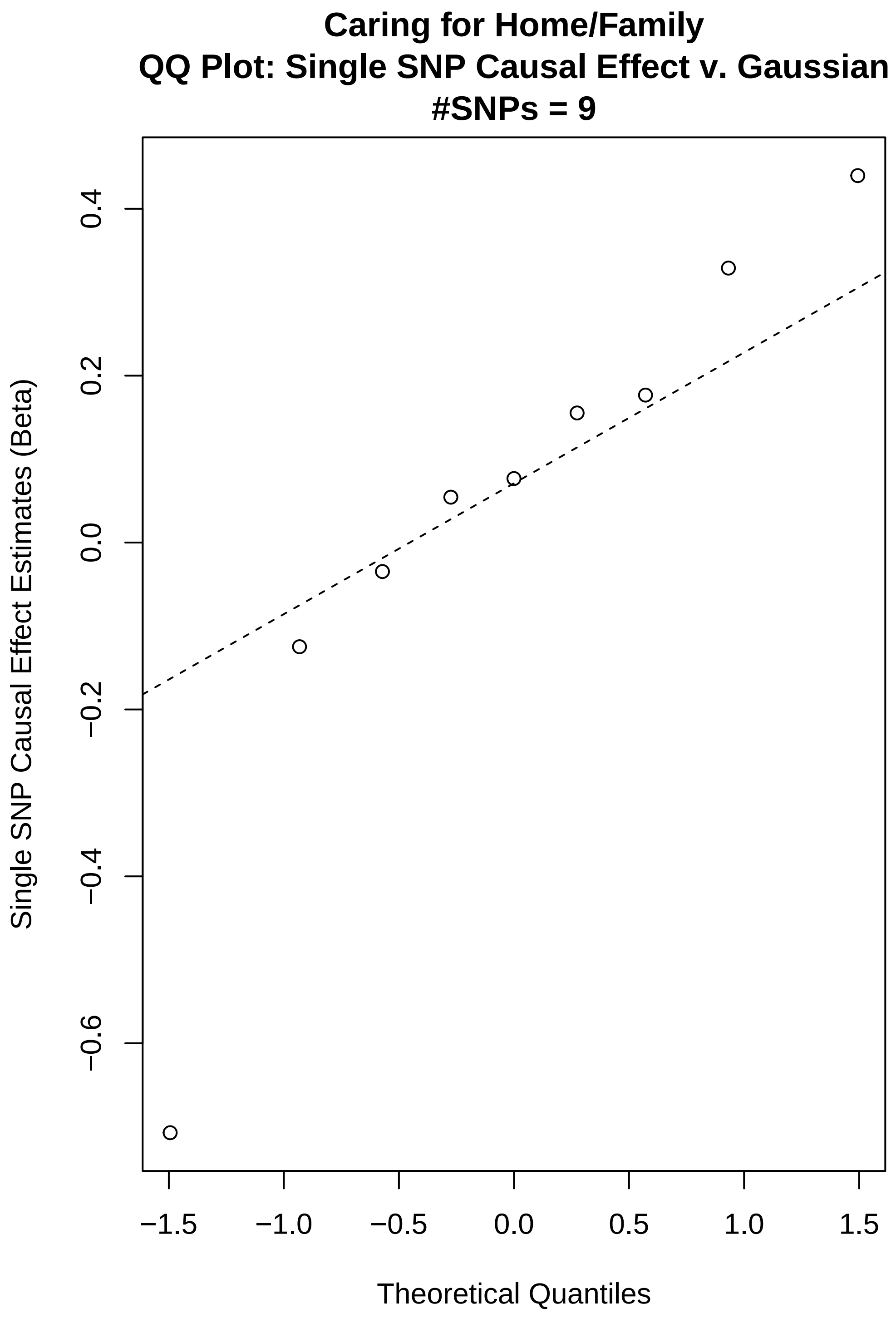
Caring for Home/Family
QQ Plot: Single SNP Causal Effect v. Gaussian
#SNPs = 9
Single SNP Causal Effect Estimates (Beta)
Theoretical Quantiles
−0.6
−0.4
−0.2
0.0
0.2
0.4
−1.5
−1.0
−0.5
0.0
0.5
1.0
1.5

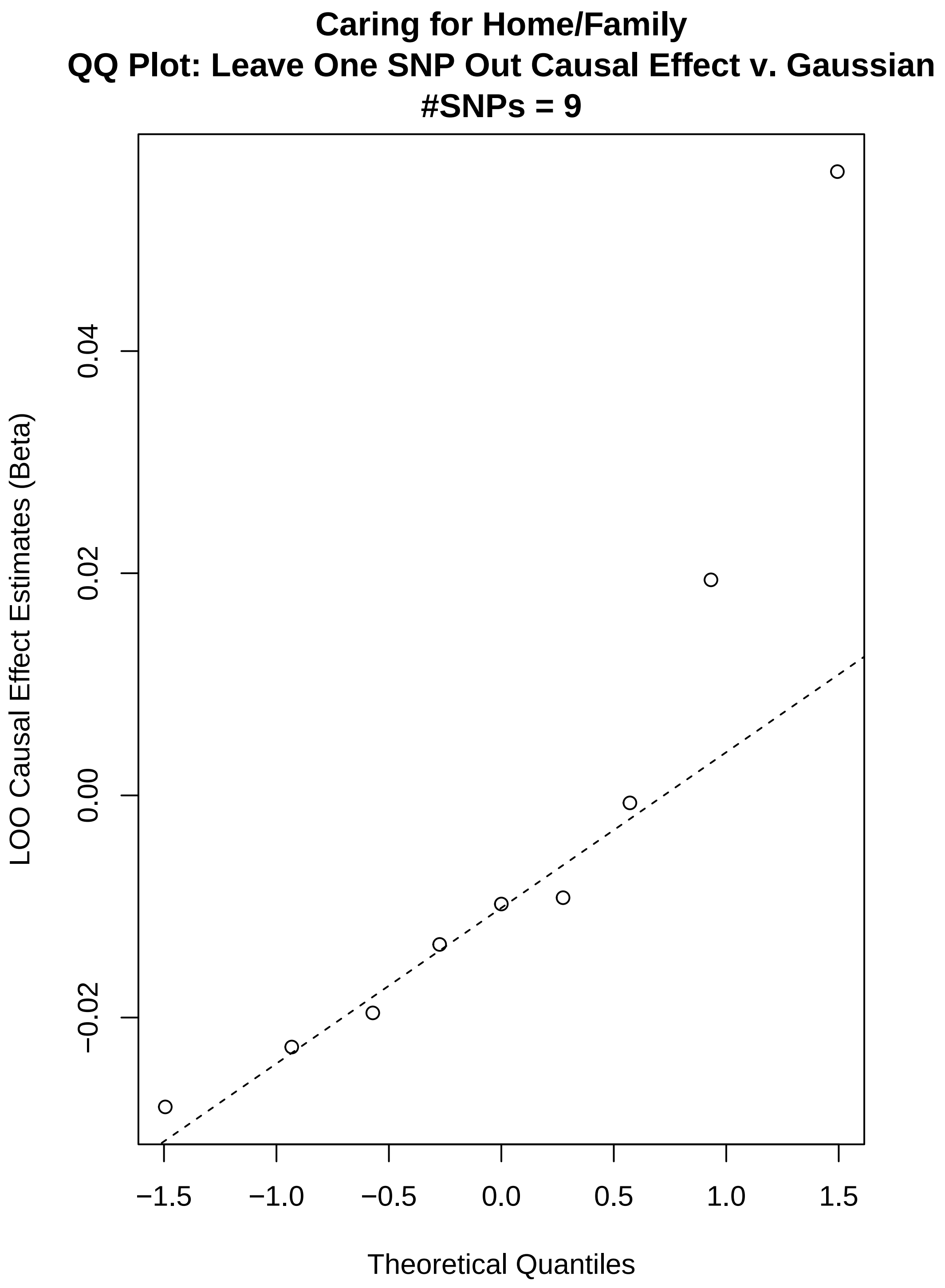
Caring for Home/Family
QQ Plot: Leave One SNP Out Causal Effect v. Gaussian
#SNPs = 9
LOO Causal Effect Estimates (Beta)
Theoretical Quantiles
−0.02
0.00
0.02
0.04
−1.5
−1.0
−0.5
0.0
0.5
1.0
1.5

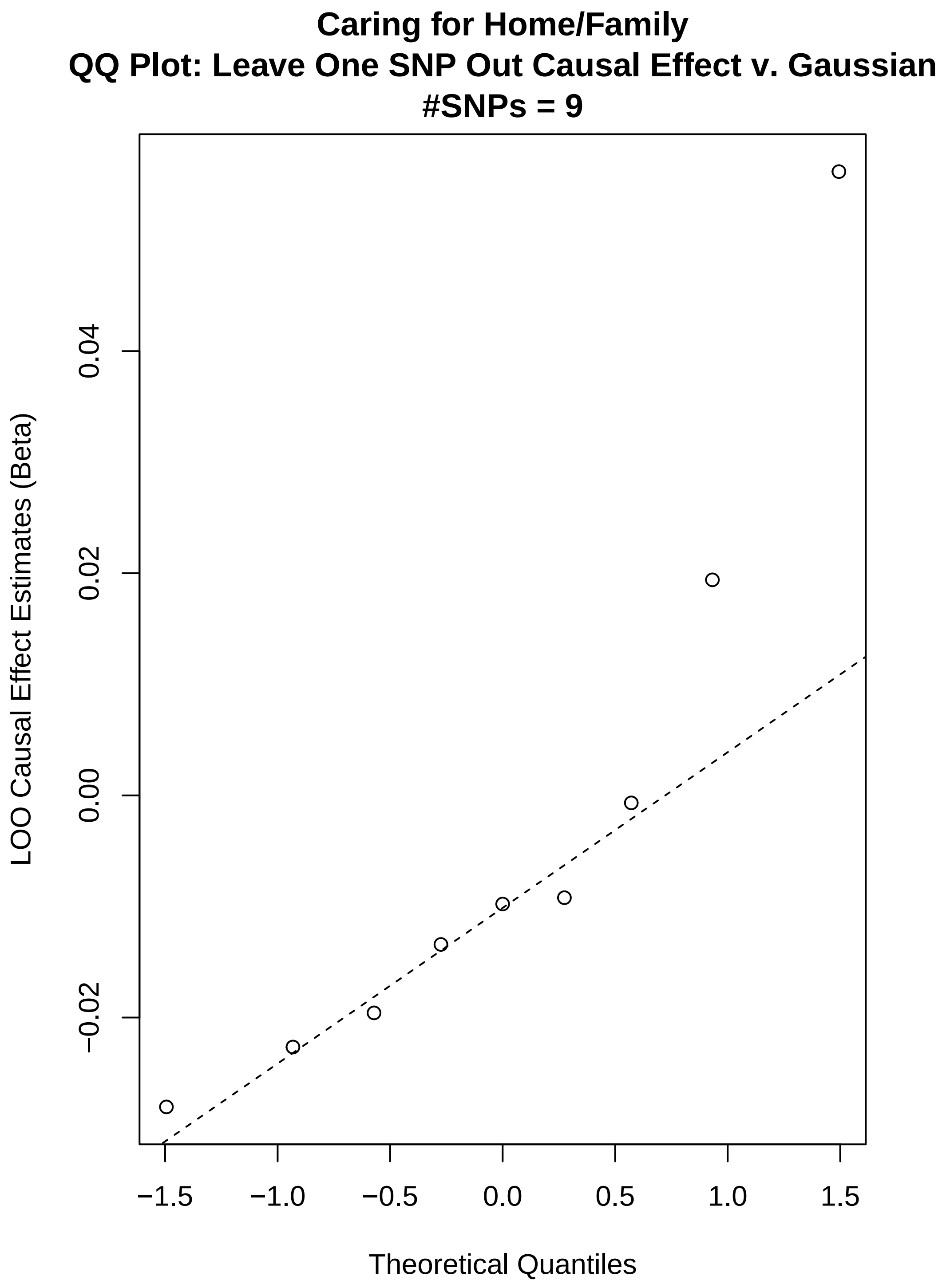
Caring for Home/Family
QQ Plot: Leave One SNP Out Causal Effect v. Gaussian
#SNPs = 9
LOO Causal Effect Estimates (Beta)
Theoretical Quantiles
−0.02
0.00
0.02
0.04
−1.5
−1.0
−0.5
0.0
0.5
1.0
1.5

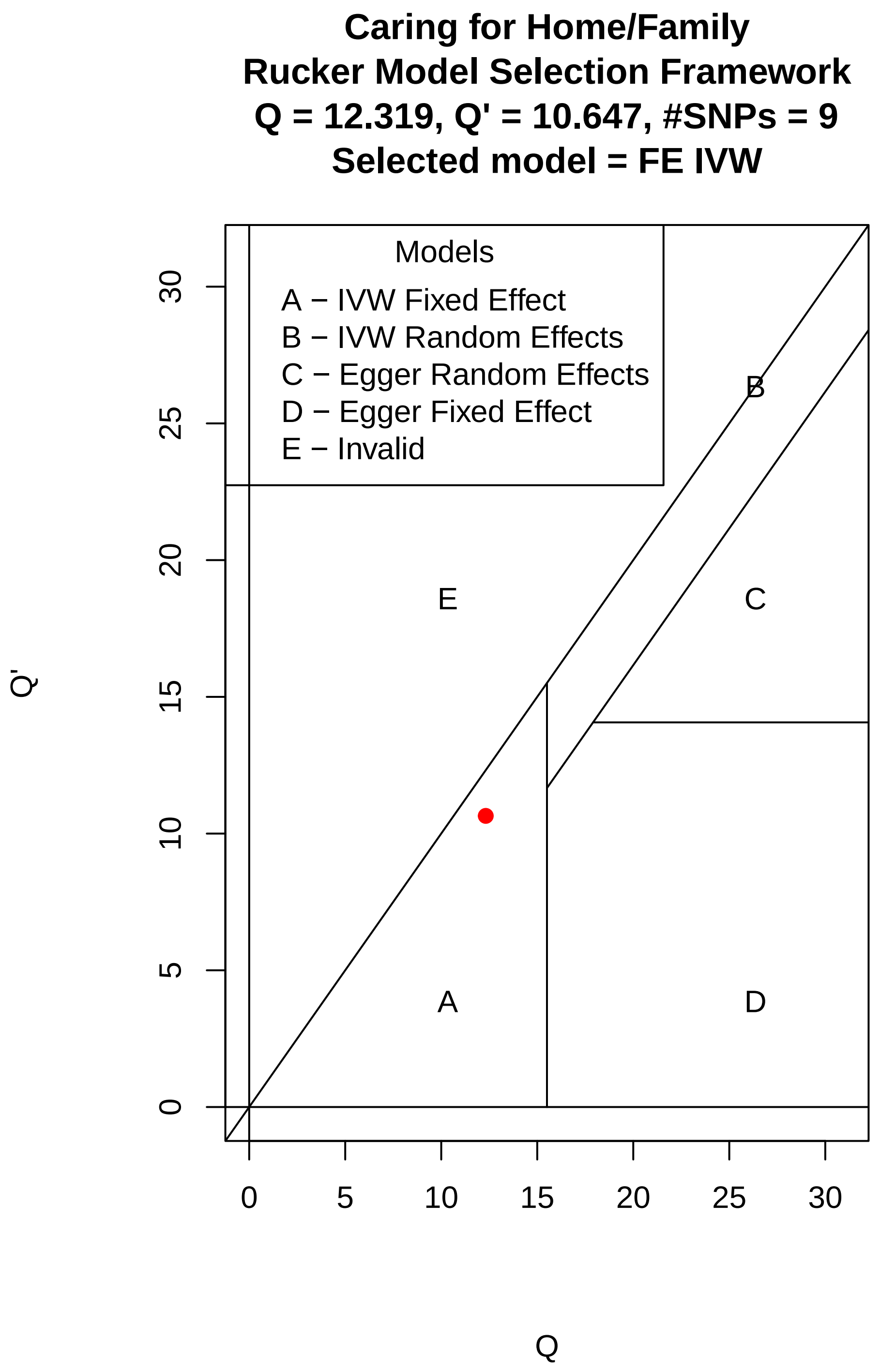
Caring for Home/Family
Rucker Model Selection Framework
Q = 12.319, Q' = 10.647, #SNPs = 9
Selected model = FE IVW
Models
A − IVW Fixed Effect
B − IVW Random Effects
C − Egger Random Effects
D − Egger Fixed Effect
E − Invalid
B
E
C
A
D
Q'
Q
0
5
10
15
20
25
30

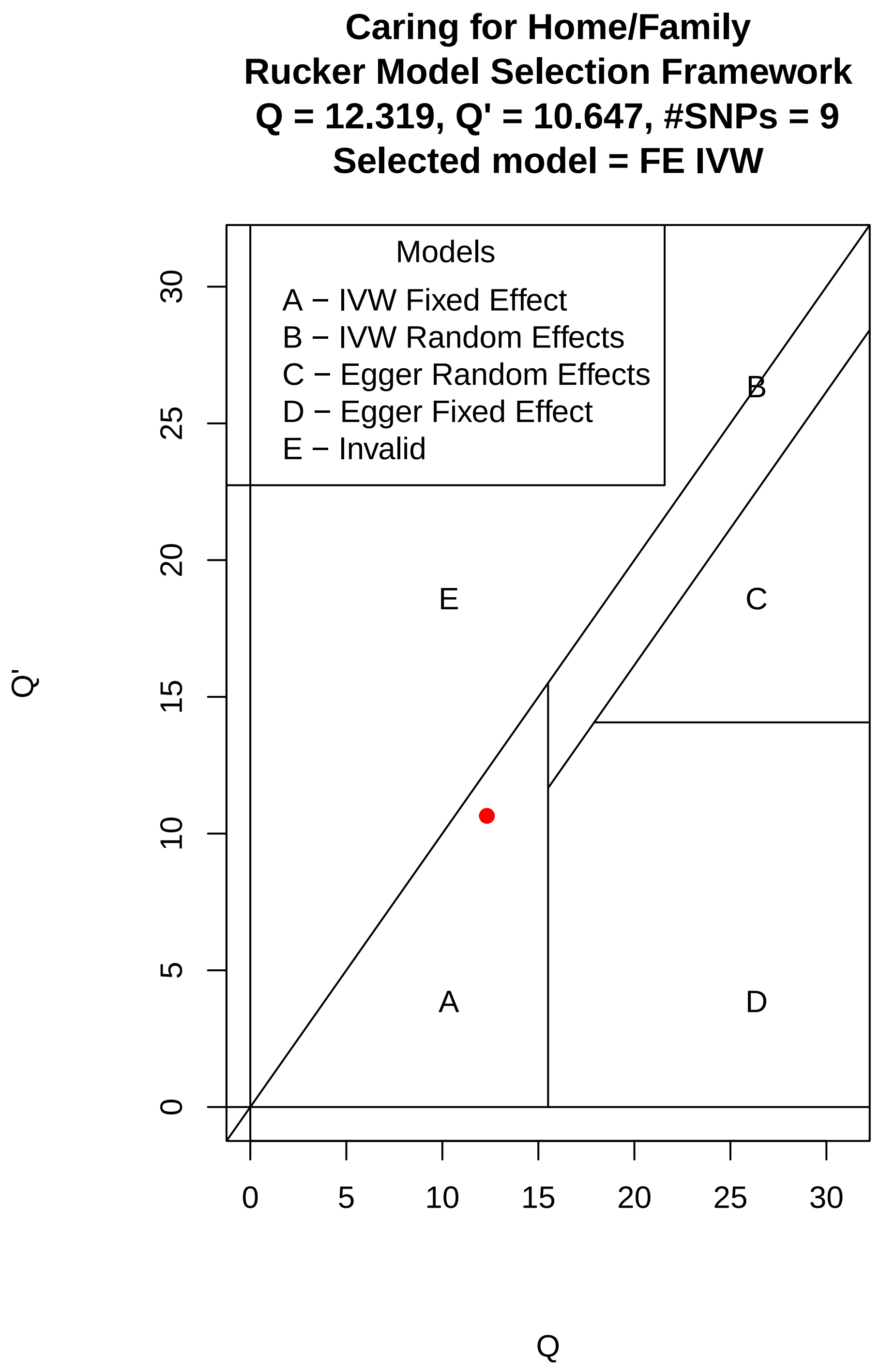
Caring for Home/Family
Rucker Model Selection Framework
Q = 12.319, Q' = 10.647, #SNPs = 9
Selected model = FE IVW
Models
A − IVW Fixed Effect
B − IVW Random Effects
C − Egger Random Effects
D − Egger Fixed Effect
E − Invalid
B
E
C
A
D
Q'
Q
0
5
10
15
20
25
30

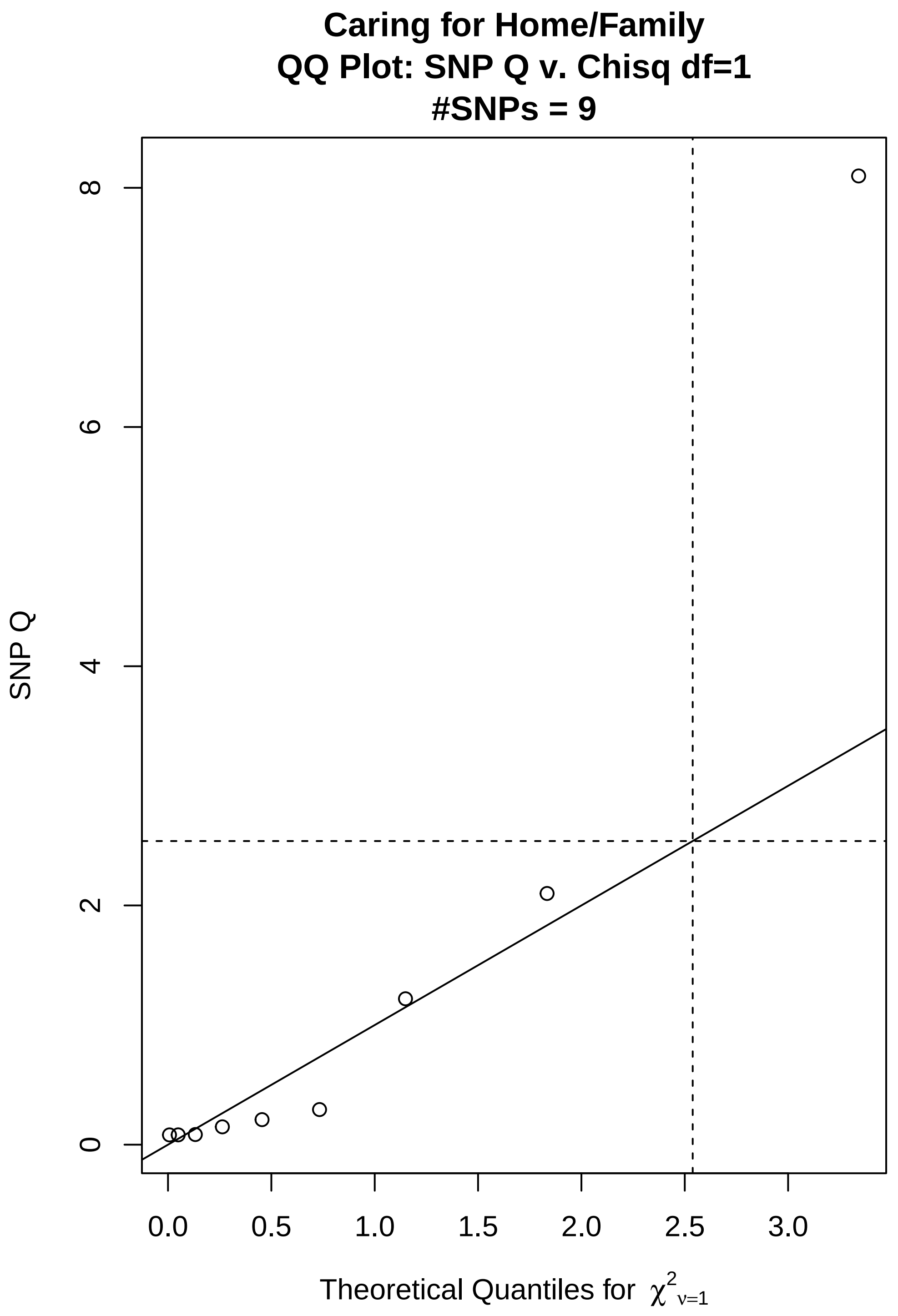
Caring for Home/Family
QQ Plot: SNP Q v. Chisq df=1
#SNPs = 9
SNP Q
0
2
4
6
8
0.0
0.5
1.0
1.5
2.0
2.5
3.0
Theoretical Quantiles for $\chi^2_{\nu=1}$

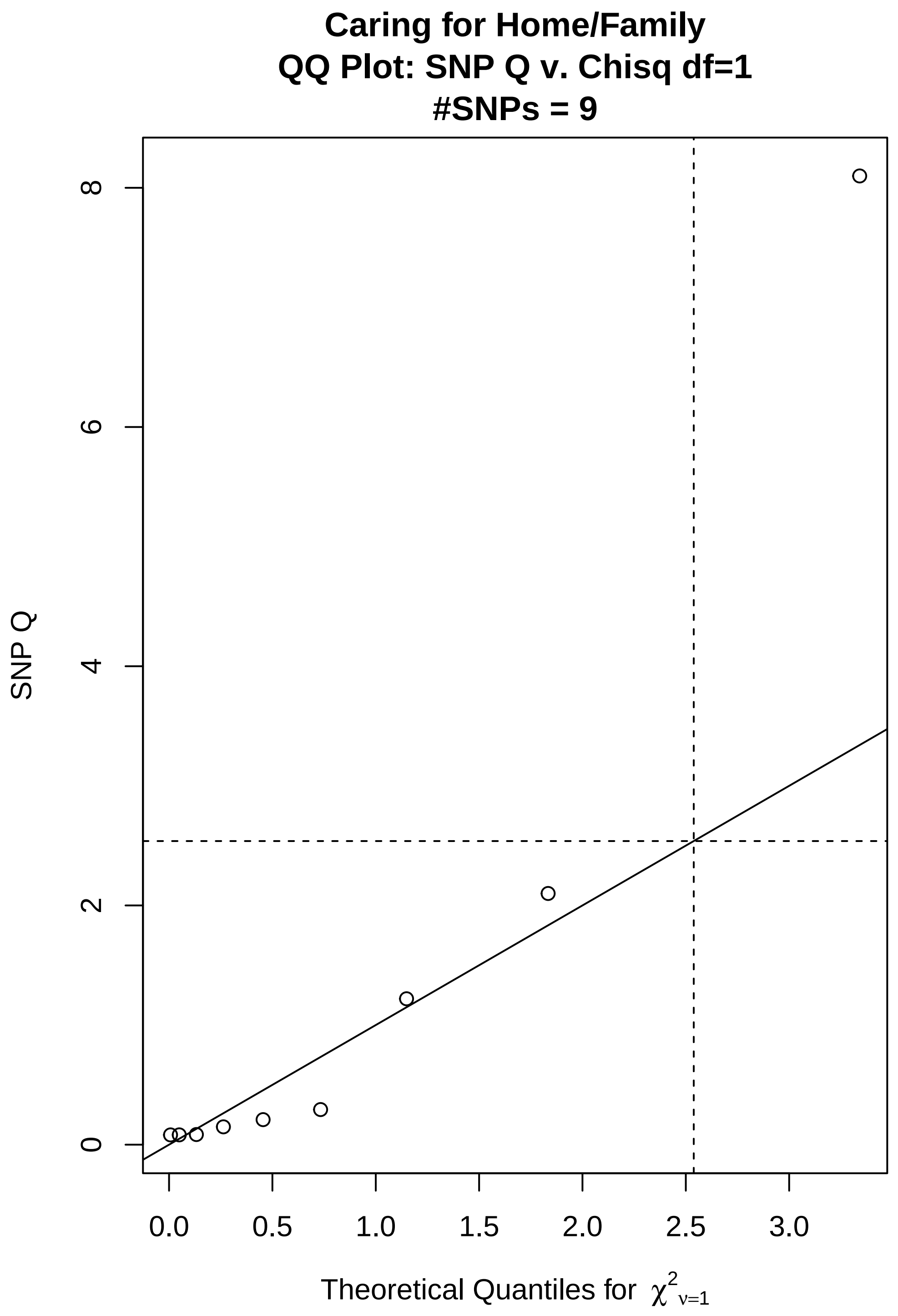
Caring for Home/Family
QQ Plot: SNP Q v. Chisq df=1
#SNPs = 9
SNP Q
0
2
4
6
8
0.0
0.5
1.0
1.5
2.0
2.5
3.0
Theoretical Quantiles for $\chi^2_{\nu=1}$
