# Supplementary material for: Effects of alcohol consumption on employment and social outcomes: a Mendelian randomisation study
Source: Alcohol Alcohol. 2025 Jul 18;60(5):agaf038. doi: 10.1093/alcalc/agaf038 (PMC12271571; doi:10.1093/alcalc/agaf038)
Supplement: Campbell_Green_Davies_et_al_2025_agaf038 [file campbell_green_davies_et_al_2025_agaf038.zip › Campbell_Green_Davies_et_al_2025/All/aud/do2SampleMrAnalyses_bAlcoholUseDisorder_iUnempNotEmp_ageSexCentreGpc.pdf]

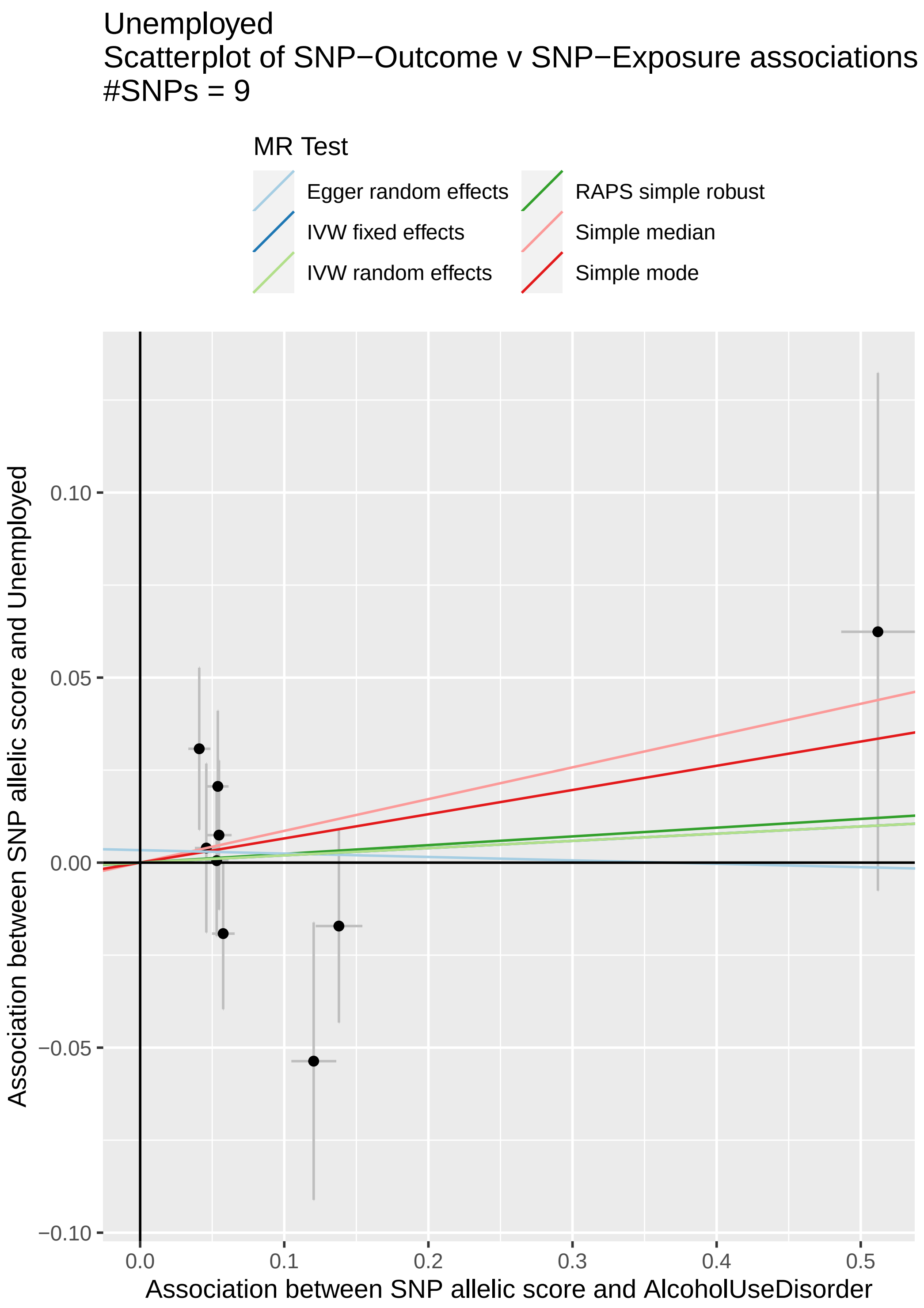

Unemployed
Scatterplot of SNP−Outcome v SNP−Exposure associations
#SNPs = 9
MR Test
Egger random effects
IVW fixed effects
IVW random effects
RAPS simple robust
Simple median
Simple mode
0.10
0.05
0.00
−0.05
−0.10
0.0
0.1
0.2
0.3
0.4
0.5
Association between SNP allelic score and Unemployed
Association between SNP allelic score and AlcoholUseDisorder

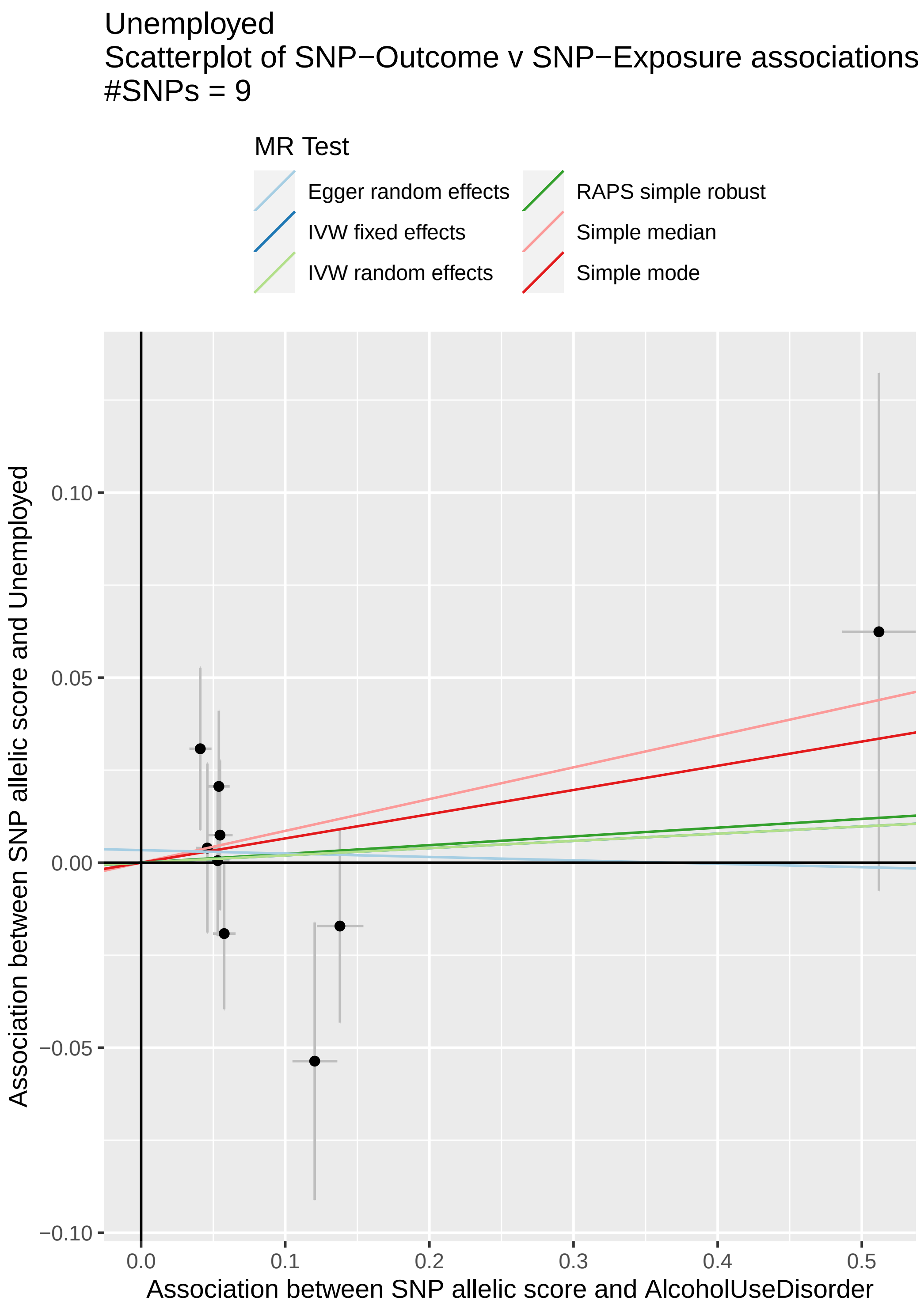

Unemployed
Scatterplot of SNP−Outcome v SNP−Exposure associations
#SNPs = 9
MR Test
Egger random effects
IVW fixed effects
IVW random effects
RAPS simple robust
Simple median
Simple mode
0.10
0.05
0.00
−0.05
−0.10
0.0
0.1
0.2
0.3
0.4
0.5
Association between SNP allelic score and Unemployed
Association between SNP allelic score and AlcoholUseDisorder

Unemployed
Causal Effect estimates for bAlcoholUseDisorder on Unemployed
#SNPs = 9, #Outlier SNPs removed = 0

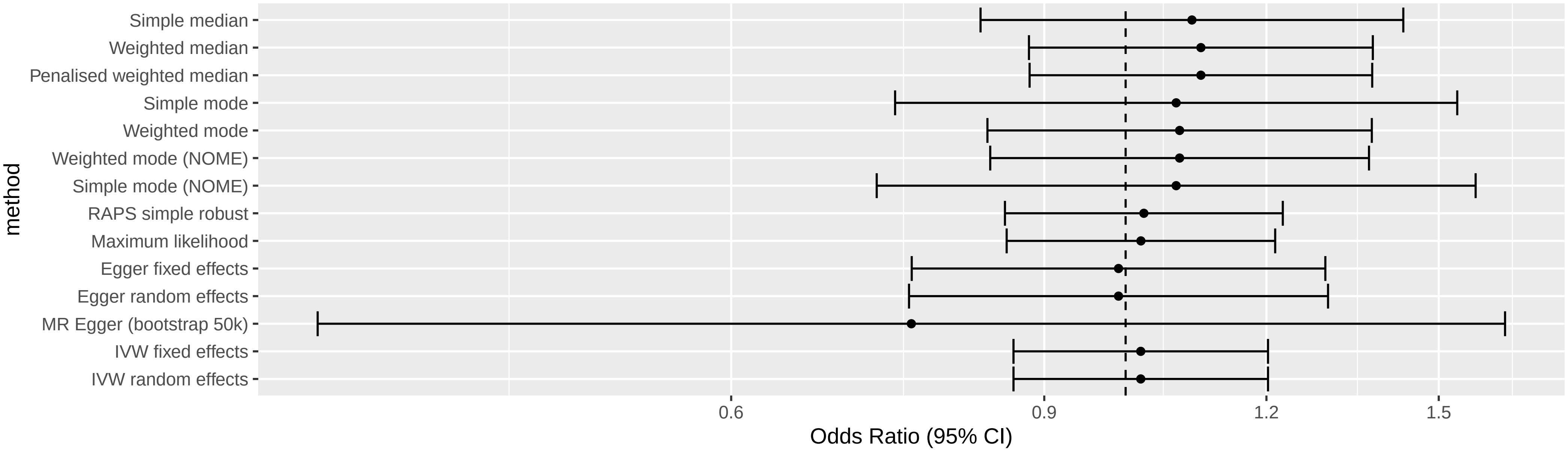

Unemployed
Causal Effect estimates for bAlcoholUseDisorder on Unemployed
#SNPs = 9, #Outlier SNPs removed = 0

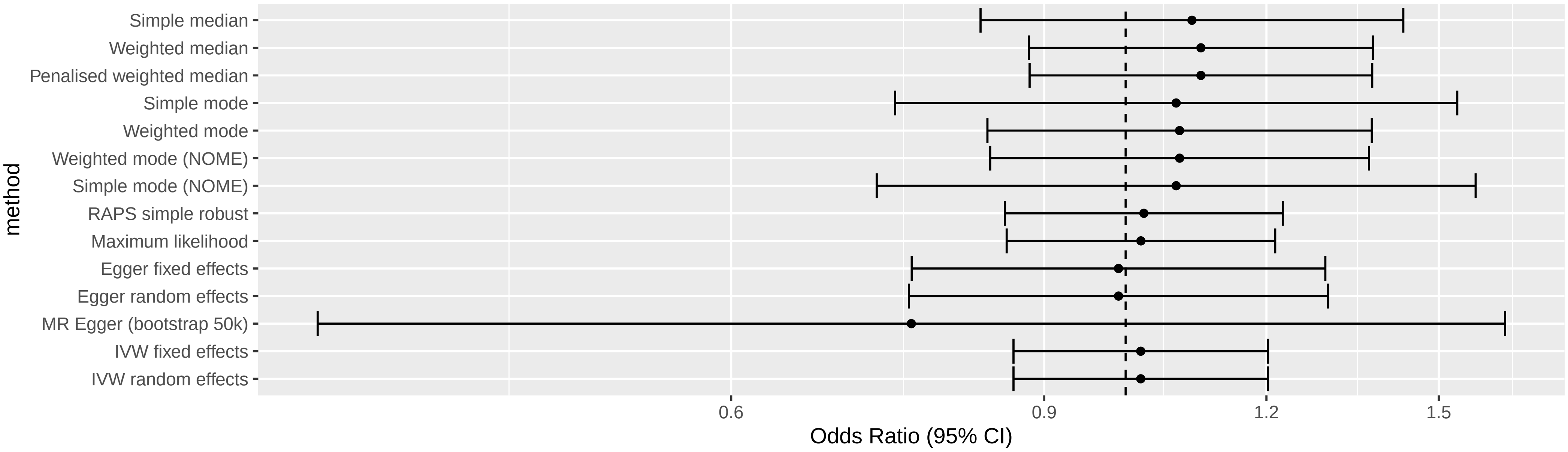

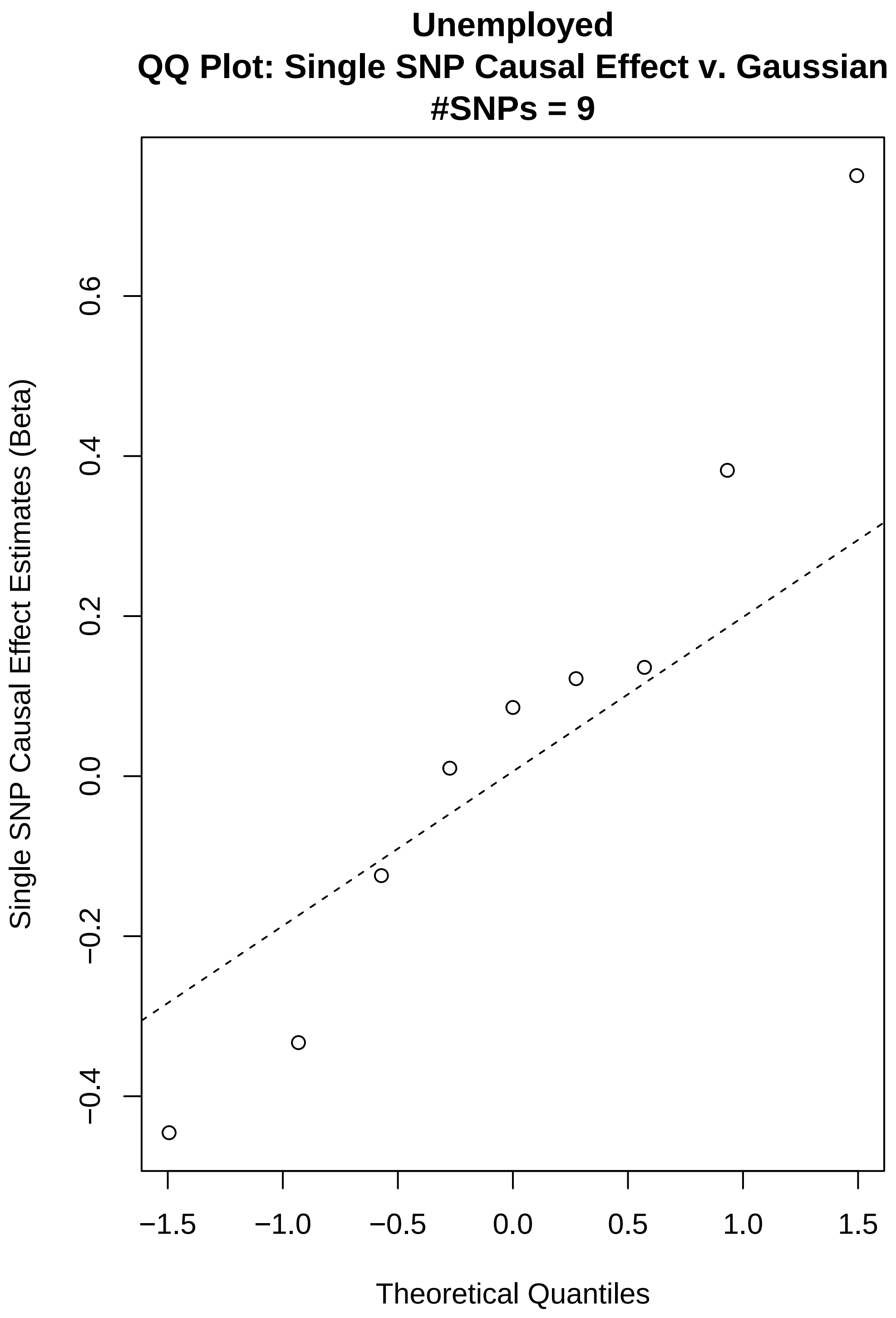
Unemployed
QQ Plot: Single SNP Causal Effect v. Gaussian
#SNPs = 9
Single SNP Causal Effect Estimates (Beta)
−0.4
−0.2
0.0
0.2
0.4
0.6
−1.5
−1.0
−0.5
0.0
0.5
1.0
1.5
Theoretical Quantiles

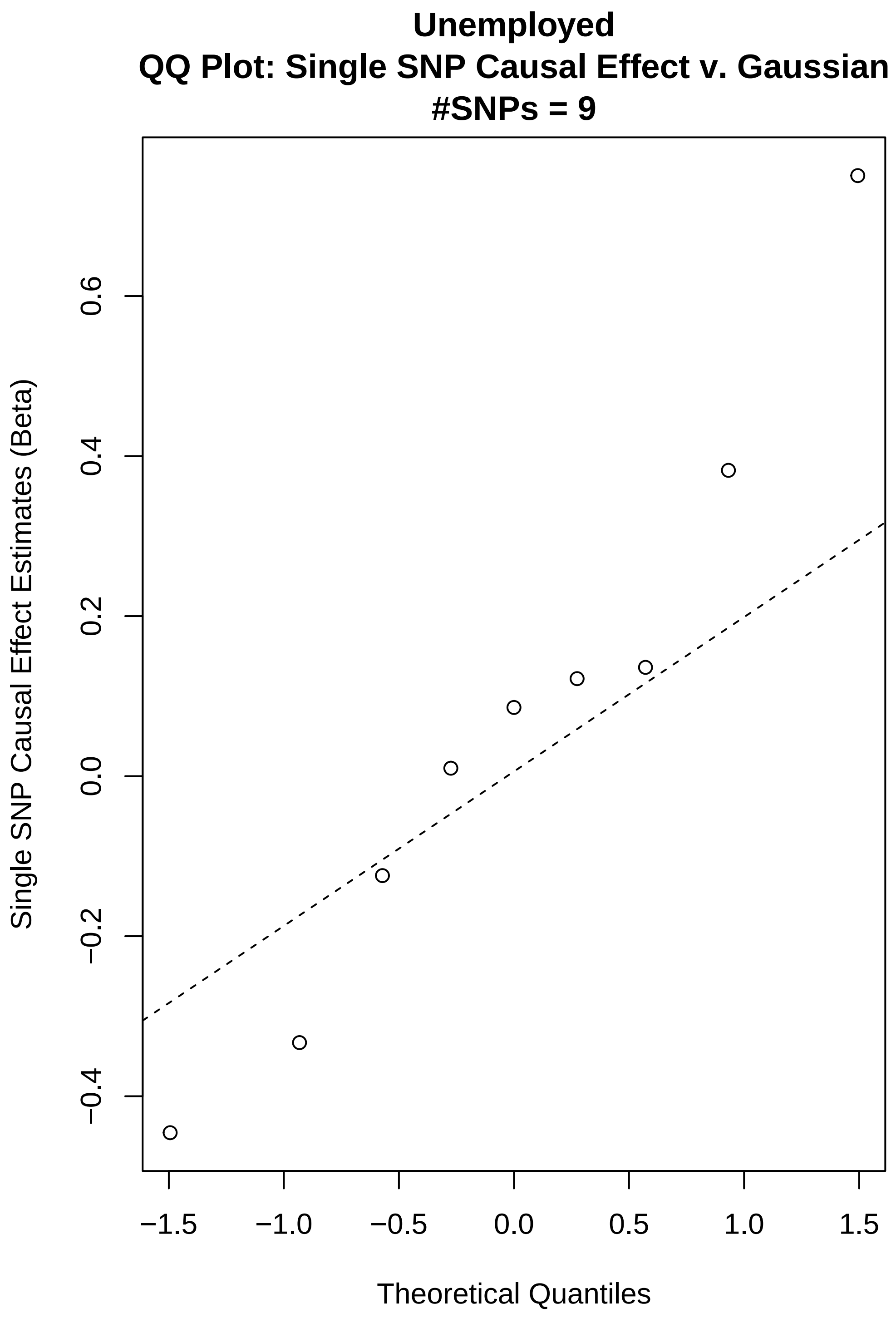
Unemployed
QQ Plot: Single SNP Causal Effect v. Gaussian
#SNPs = 9
Single SNP Causal Effect Estimates (Beta)
−0.4
−0.2
0.0
0.2
0.4
0.6
−1.5
−1.0
−0.5
0.0
0.5
1.0
1.5
Theoretical Quantiles

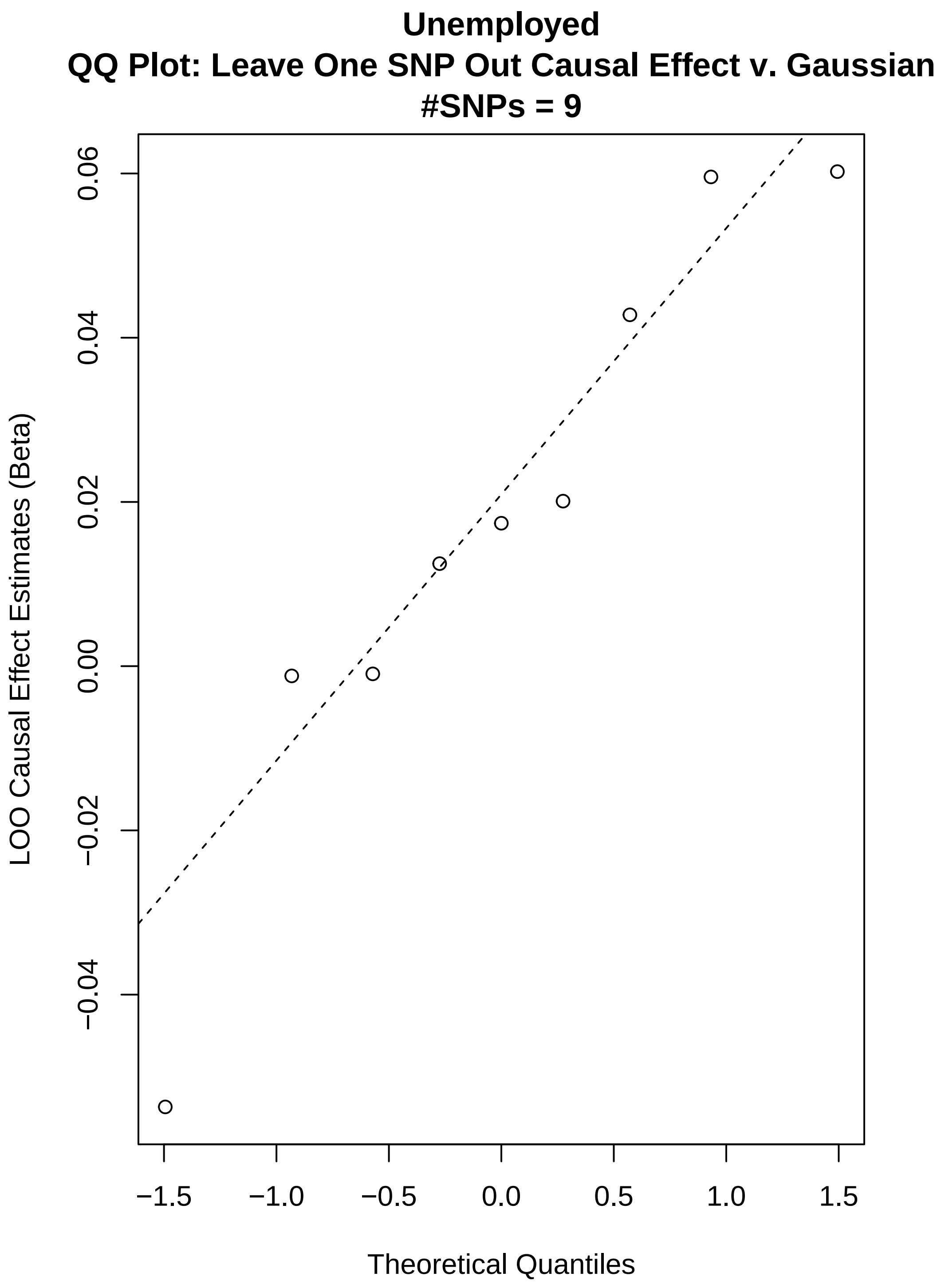
Unemployed
QQ Plot: Leave One SNP Out Causal Effect v. Gaussian
#SNPs = 9
LOO Causal Effect Estimates (Beta)
0.06
0.04
0.02
0.00
−0.02
−0.04
−1.5
−1.0
−0.5
0.0
0.5
1.0
1.5
Theoretical Quantiles

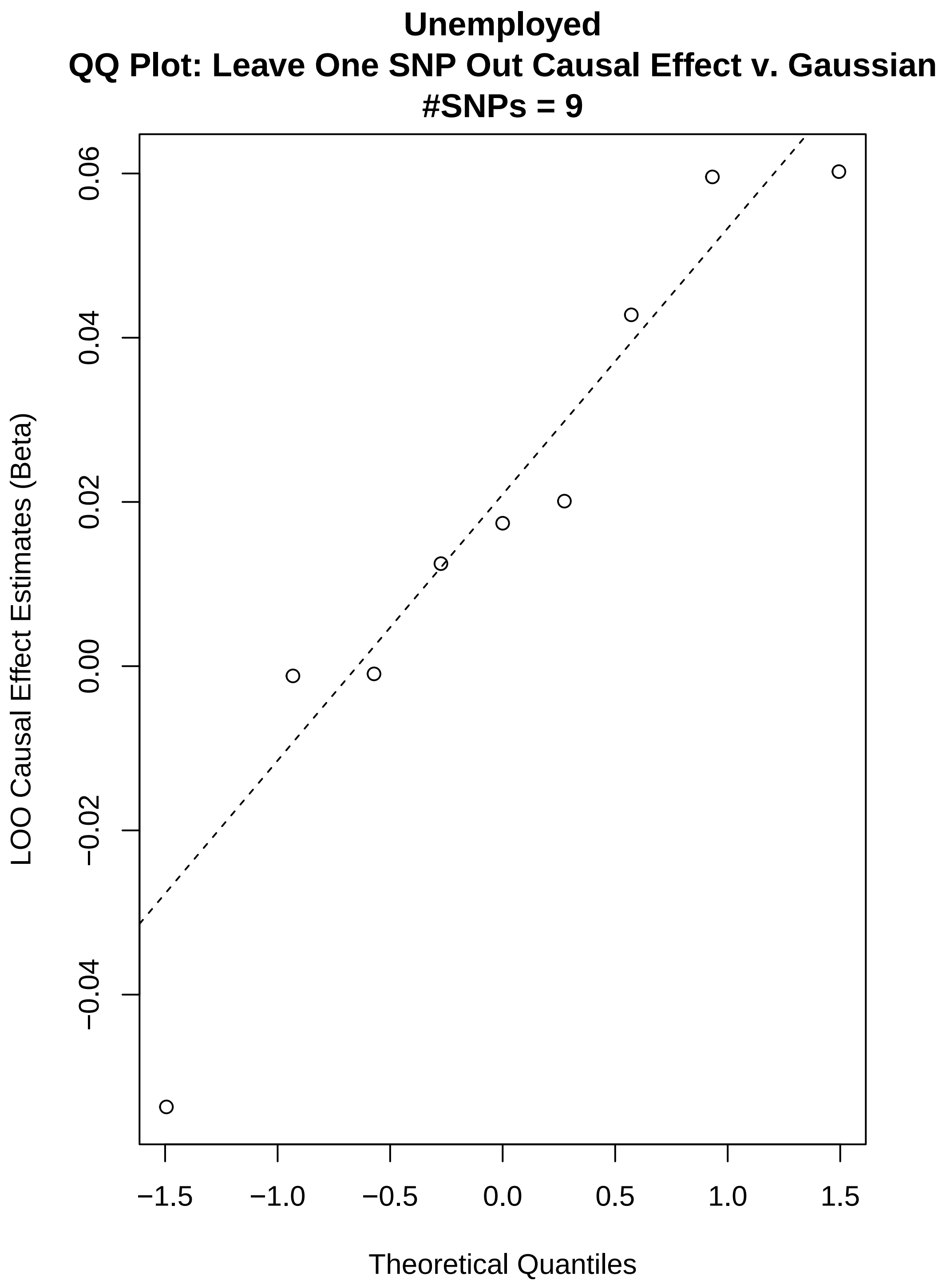
Unemployed
QQ Plot: Leave One SNP Out Causal Effect v. Gaussian
#SNPs = 9
LOO Causal Effect Estimates (Beta)
0.06
0.04
0.02
0.00
−0.02
−0.04
−1.5
−1.0
−0.5
0.0
0.5
1.0
1.5
Theoretical Quantiles

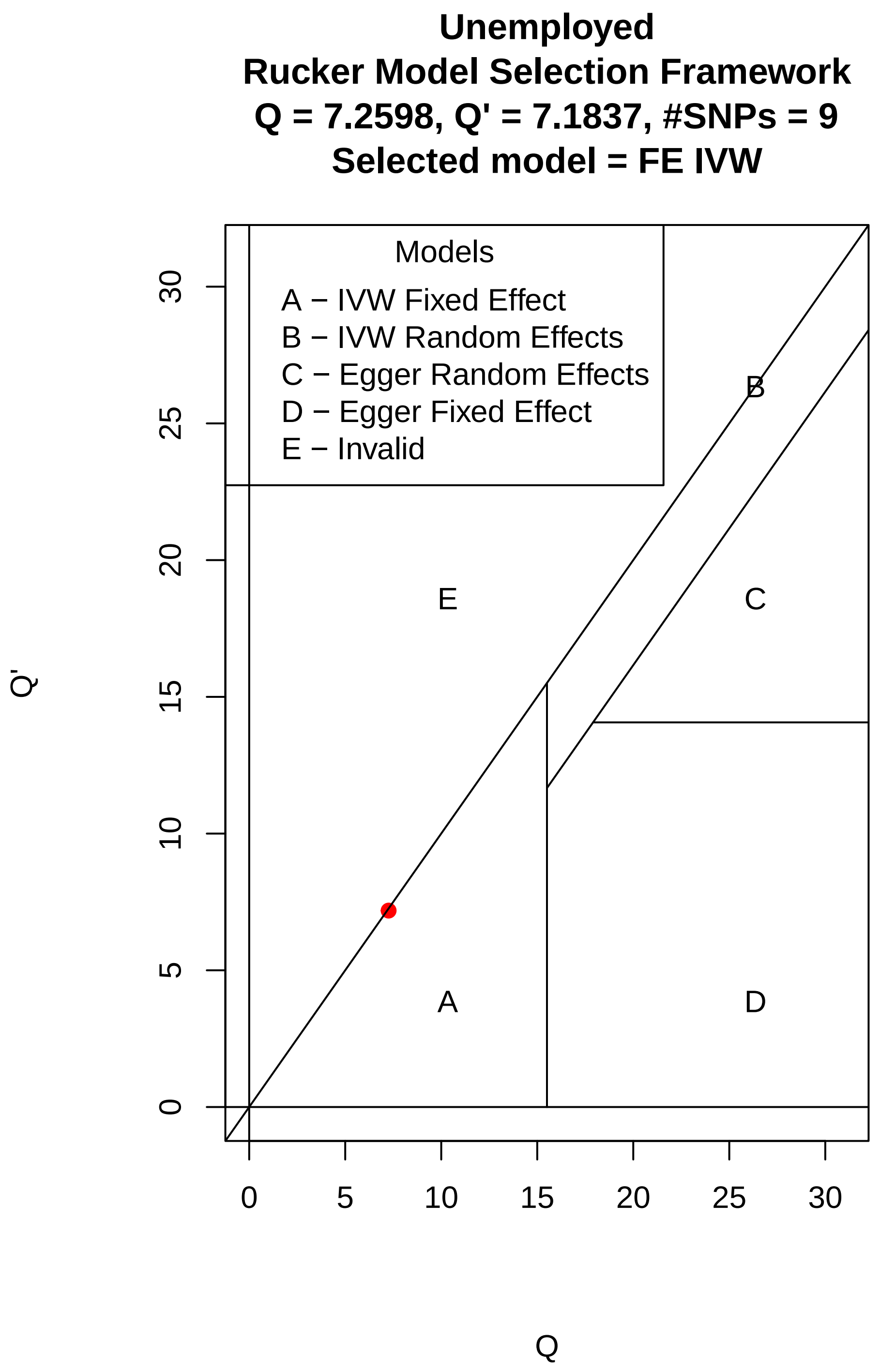
Unemployed
Rucker Model Selection Framework
Q = 7.2598, Q' = 7.1837, #SNPs = 9
Selected model = FE IVW
Models
A − IVW Fixed Effect
B − IVW Random Effects
C − Egger Random Effects
D − Egger Fixed Effect
E − Invalid
A
B
C
D
E
Q
Q'
0
5
10
15
20
25
30

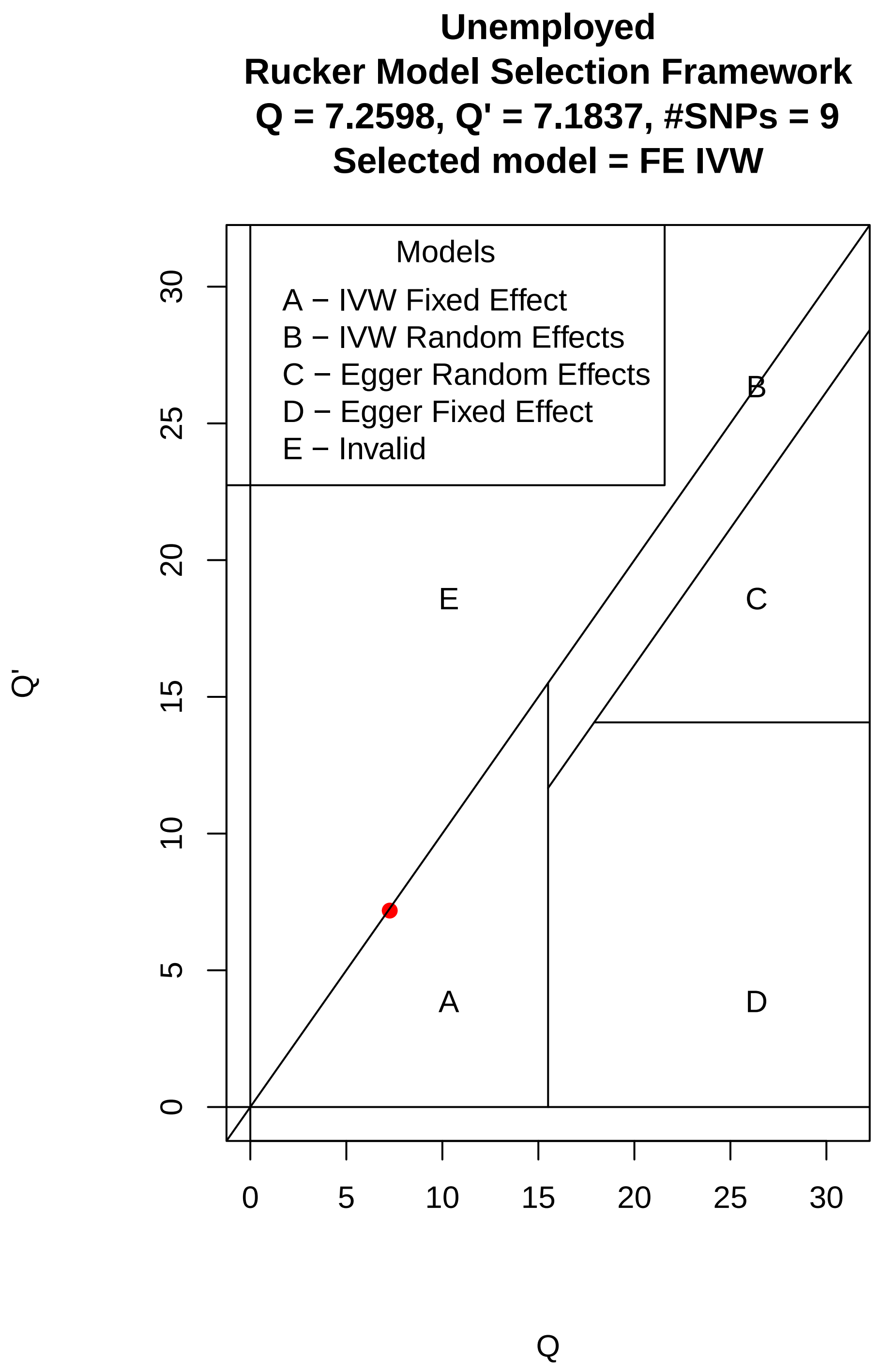
Unemployed
Rucker Model Selection Framework
Q = 7.2598, Q' = 7.1837, #SNPs = 9
Selected model = FE IVW
Models
A − IVW Fixed Effect
B − IVW Random Effects
C − Egger Random Effects
D − Egger Fixed Effect
E − Invalid
A
B
C
D
E
Q
Q'
0
5
10
15
20
25
30

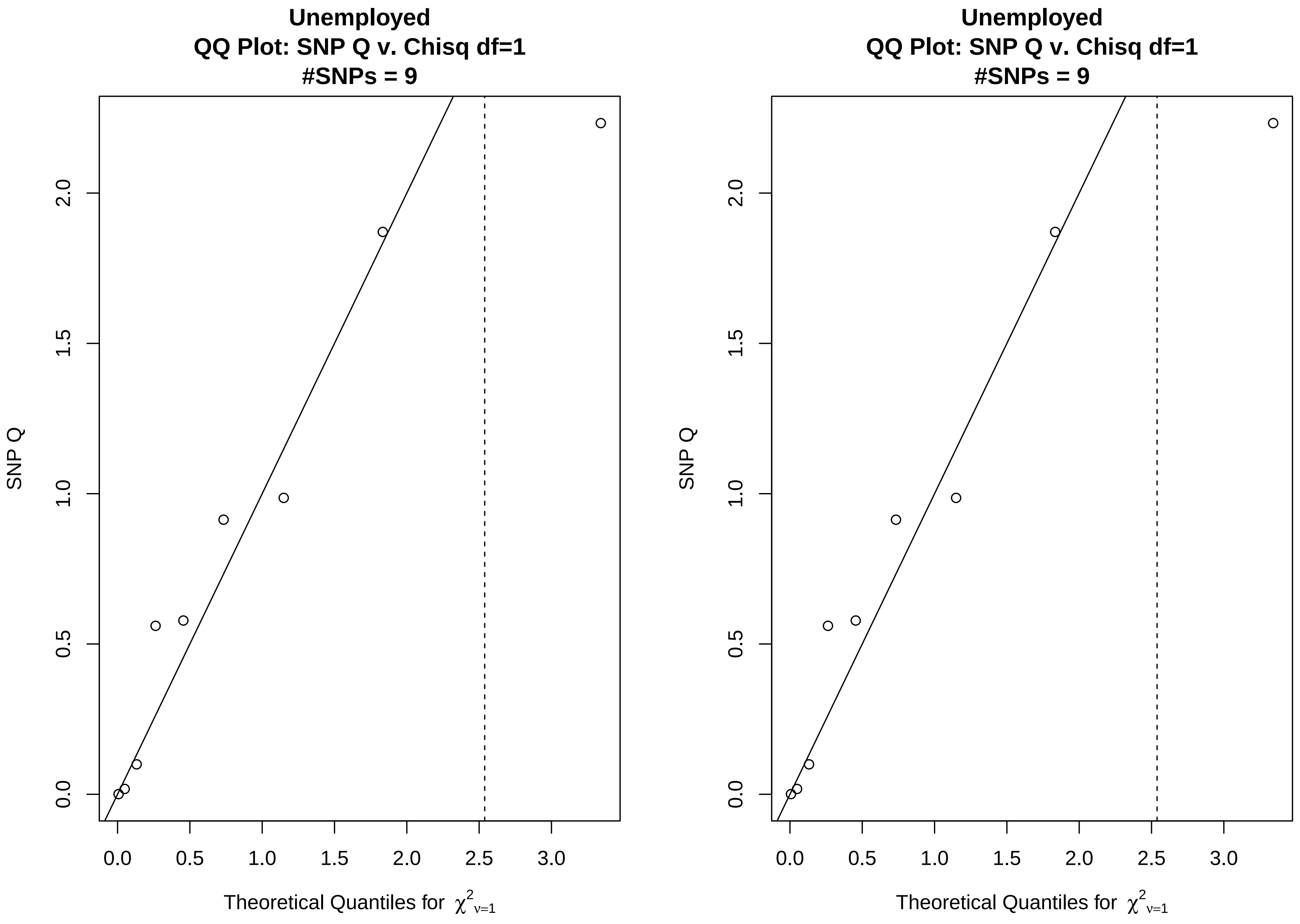
Unemployed
QQ Plot: SNP Q v. Chisq df=1
#SNPs = 9
SNP Q
0.0
0.5
1.0
1.5
2.0
0.0
0.5
1.0
1.5
2.0
2.5
3.0
Theoretical Quantiles for $\chi^2_{\nu=1}$
Unemployed
QQ Plot: SNP Q v. Chisq df=1
#SNPs = 9
SNP Q
0.0
0.5
1.0
1.5
2.0
0.0
0.5
1.0
1.5
2.0
2.5
3.0
Theoretical Quantiles for $\chi^2_{\nu=1}$
