# Supplementary material for: Effects of alcohol consumption on employment and social outcomes: a Mendelian randomisation study
Source: Alcohol Alcohol. 2025 Jul 18;60(5):agaf038. doi: 10.1093/alcalc/agaf038 (PMC12271571; doi:10.1093/alcalc/agaf038)
Supplement: Campbell_Green_Davies_et_al_2025_agaf038 [file campbell_green_davies_et_al_2025_agaf038.zip › Campbell_Green_Davies_et_al_2025/All/drink/do2SampleMrAnalyses_alcoholUnitsWeekly_wins140_iSickNotEmp_ageSexCentreGpc.pdf]

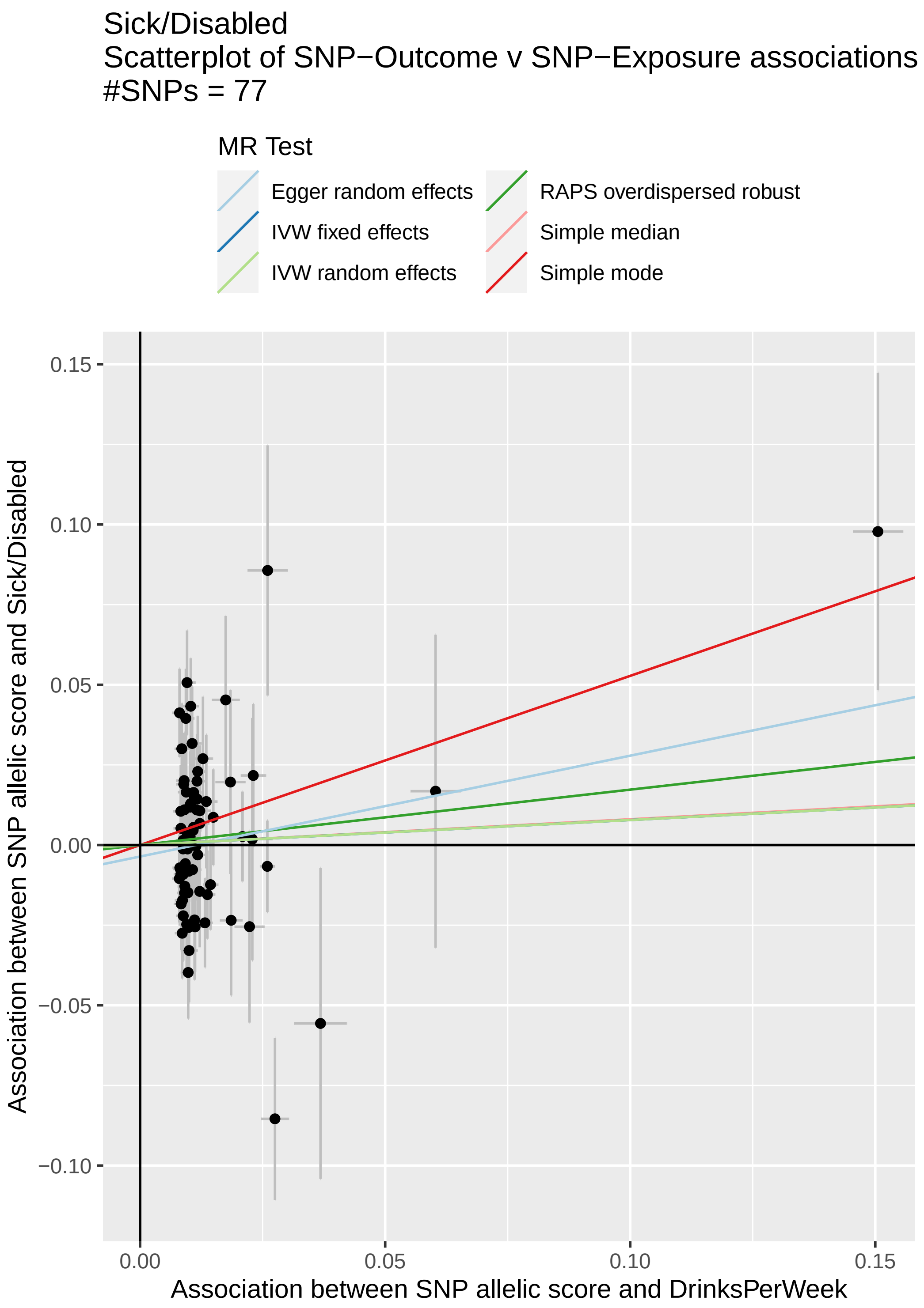
Sick/Disabled
Scatterplot of SNP−Outcome v SNP−Exposure associations
#SNPs = 77
MR Test
Egger random effects
IVW fixed effects
IVW random effects
RAPS overdispersed robust
Simple median
Simple mode
Association between SNP allelic score and Sick/Disabled
Association between SNP allelic score and DrinksPerWeek
0.15
0.10
0.05
0.00
−0.05
−0.10
0.00
0.05
0.10
0.15

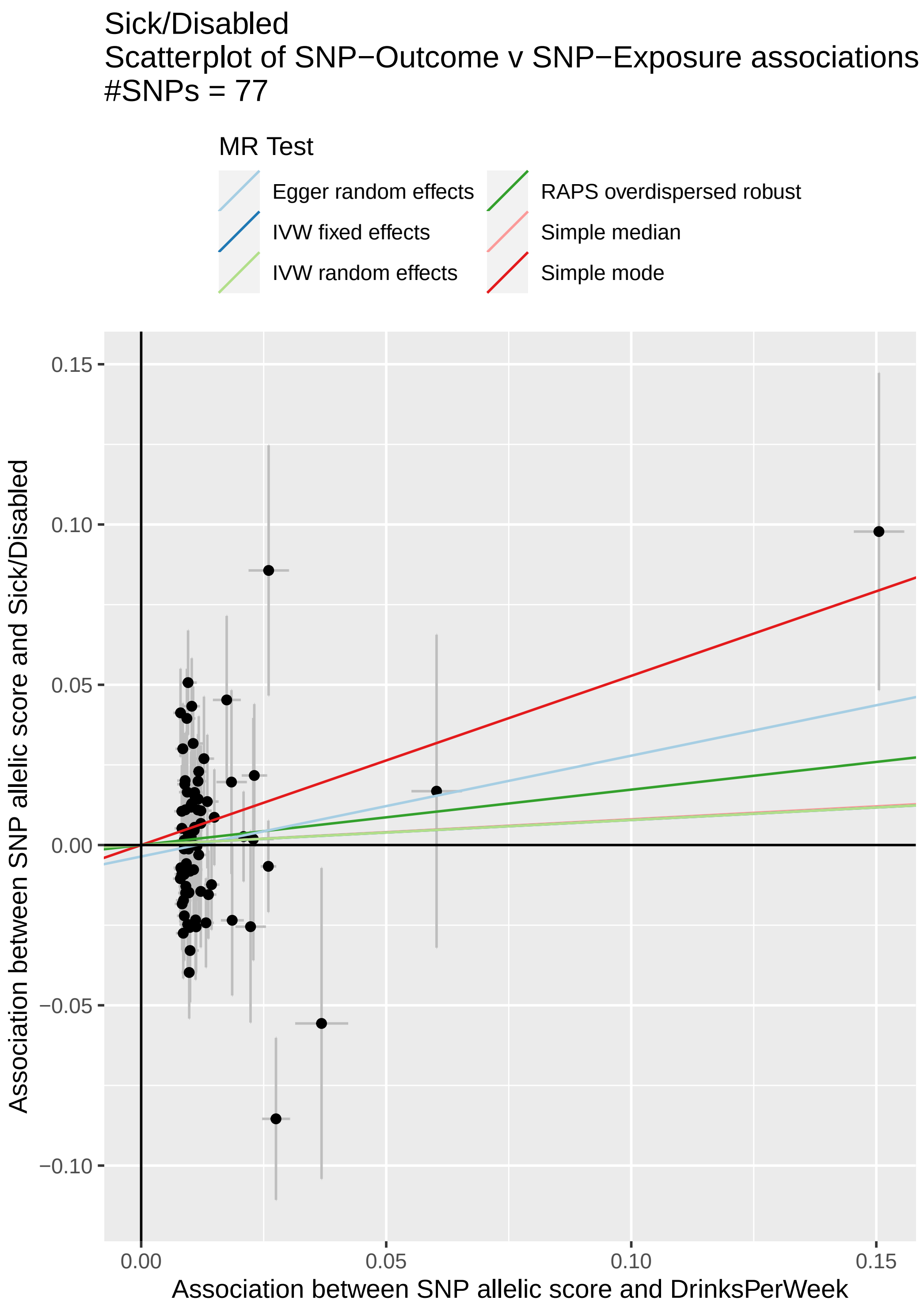
Sick/Disabled
Scatterplot of SNP−Outcome v SNP−Exposure associations
#SNPs = 77
MR Test
Egger random effects
IVW fixed effects
IVW random effects
RAPS overdispersed robust
Simple median
Simple mode
Association between SNP allelic score and Sick/Disabled
Association between SNP allelic score and DrinksPerWeek
0.15
0.10
0.05
0.00
−0.05
−0.10
0.00
0.05
0.10
0.15

Sick/Disabled
Causal Effect estimates for alcoholUnitsWeekly_wins140 on Sick/Disabled
#SNPs = 77, #Outlier SNPs removed = 0

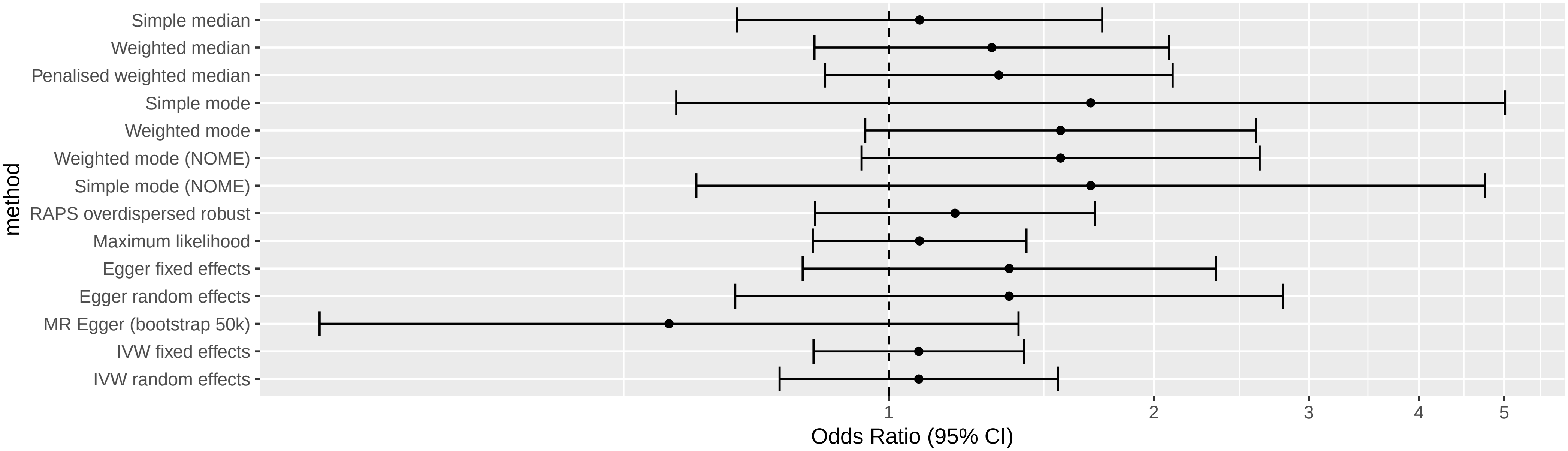

Sick/Disabled
Causal Effect estimates for alcoholUnitsWeekly_wins140 on Sick/Disabled
#SNPs = 77, #Outlier SNPs removed = 0

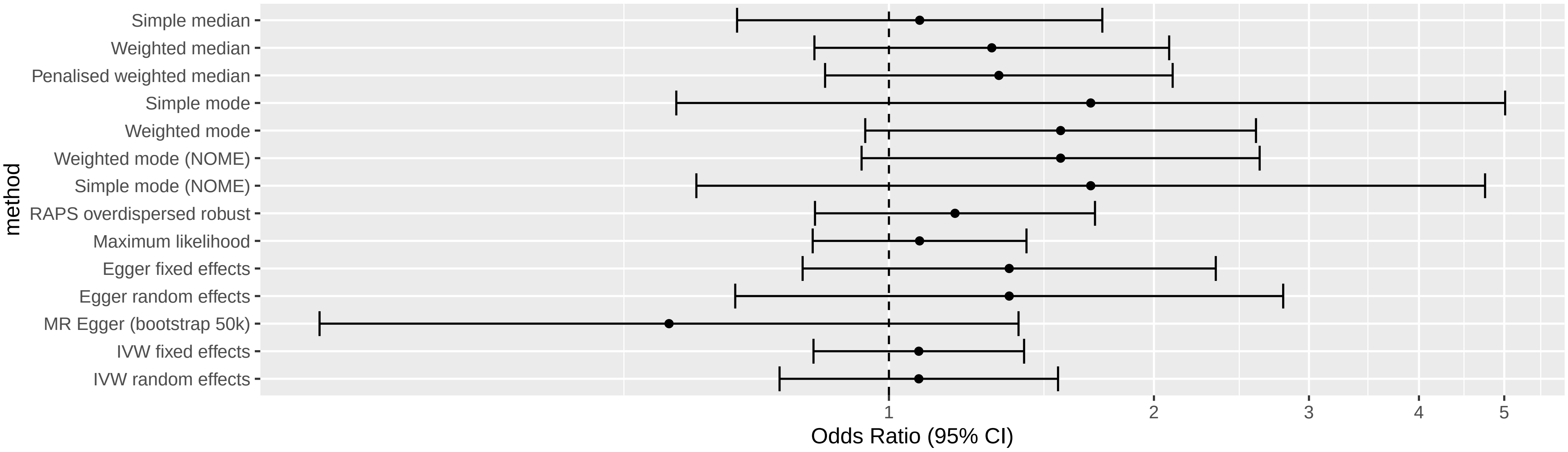

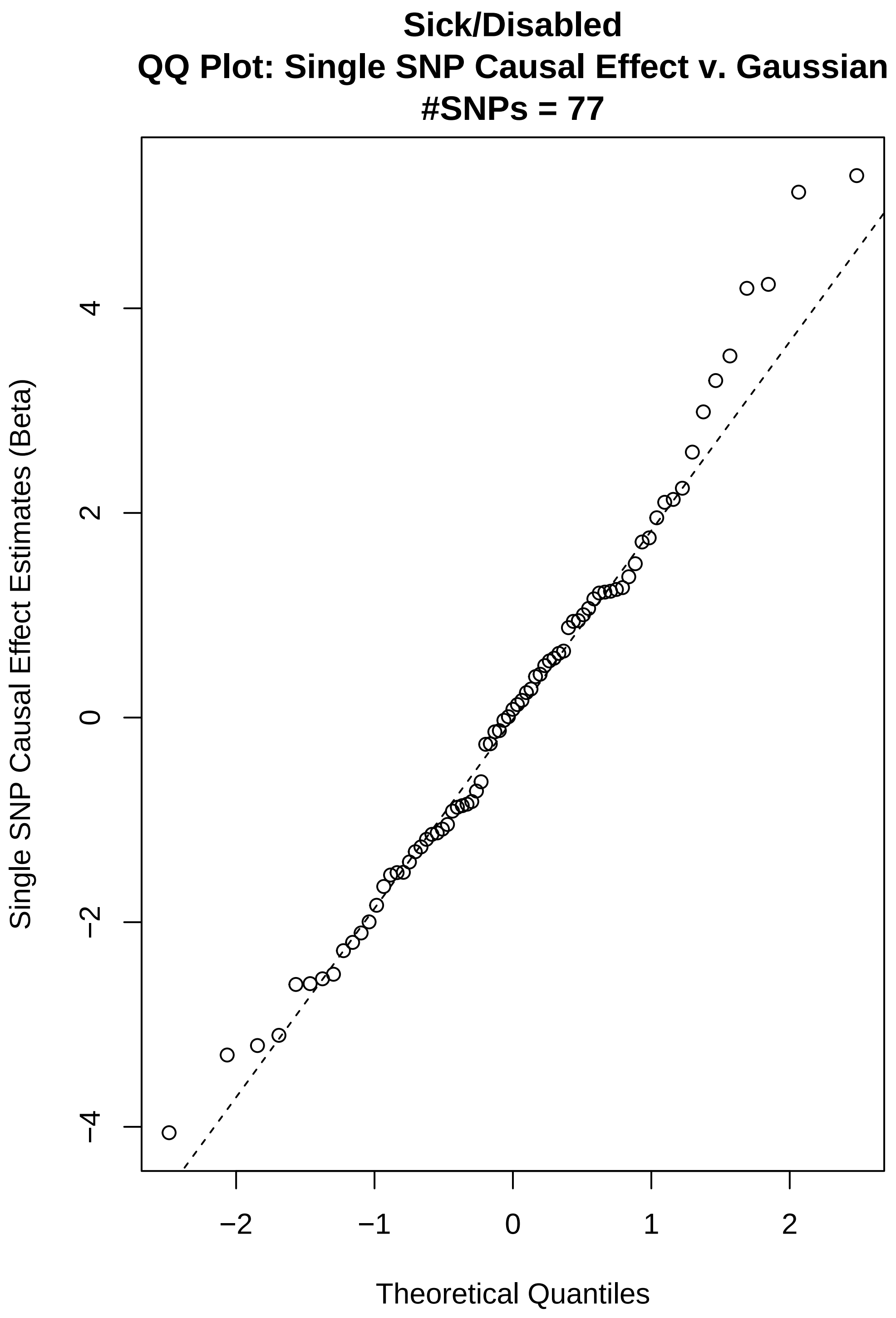
Sick/Disabled
QQ Plot: Single SNP Causal Effect v. Gaussian
#SNPs = 77
Single SNP Causal Effect Estimates (Beta)
Theoretical Quantiles
−4
−2
0
2
4
−2
−1
0
1
2

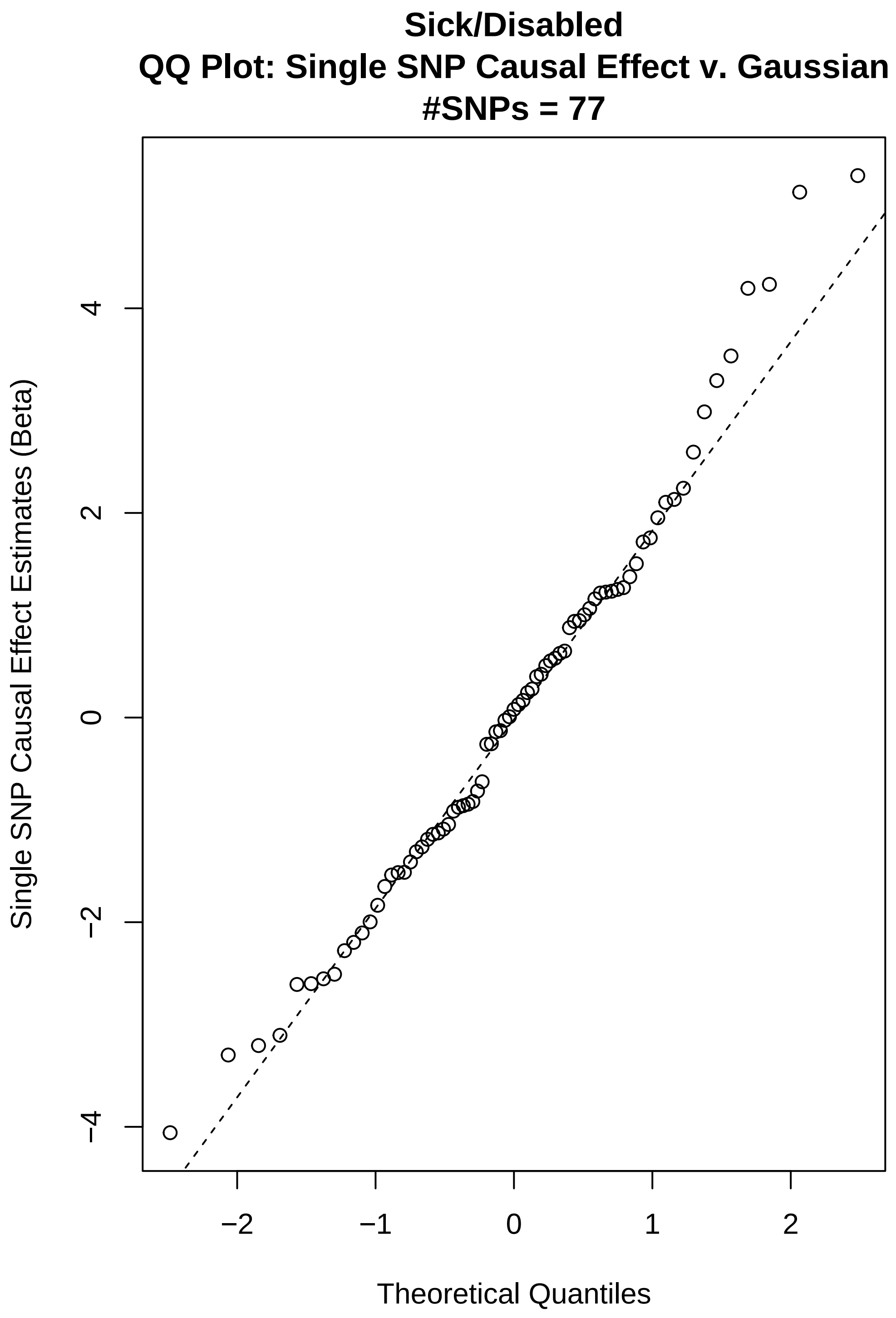
Sick/Disabled
QQ Plot: Single SNP Causal Effect v. Gaussian
#SNPs = 77
Single SNP Causal Effect Estimates (Beta)
Theoretical Quantiles
−4
−2
0
2
4
−2
−1
0
1
2

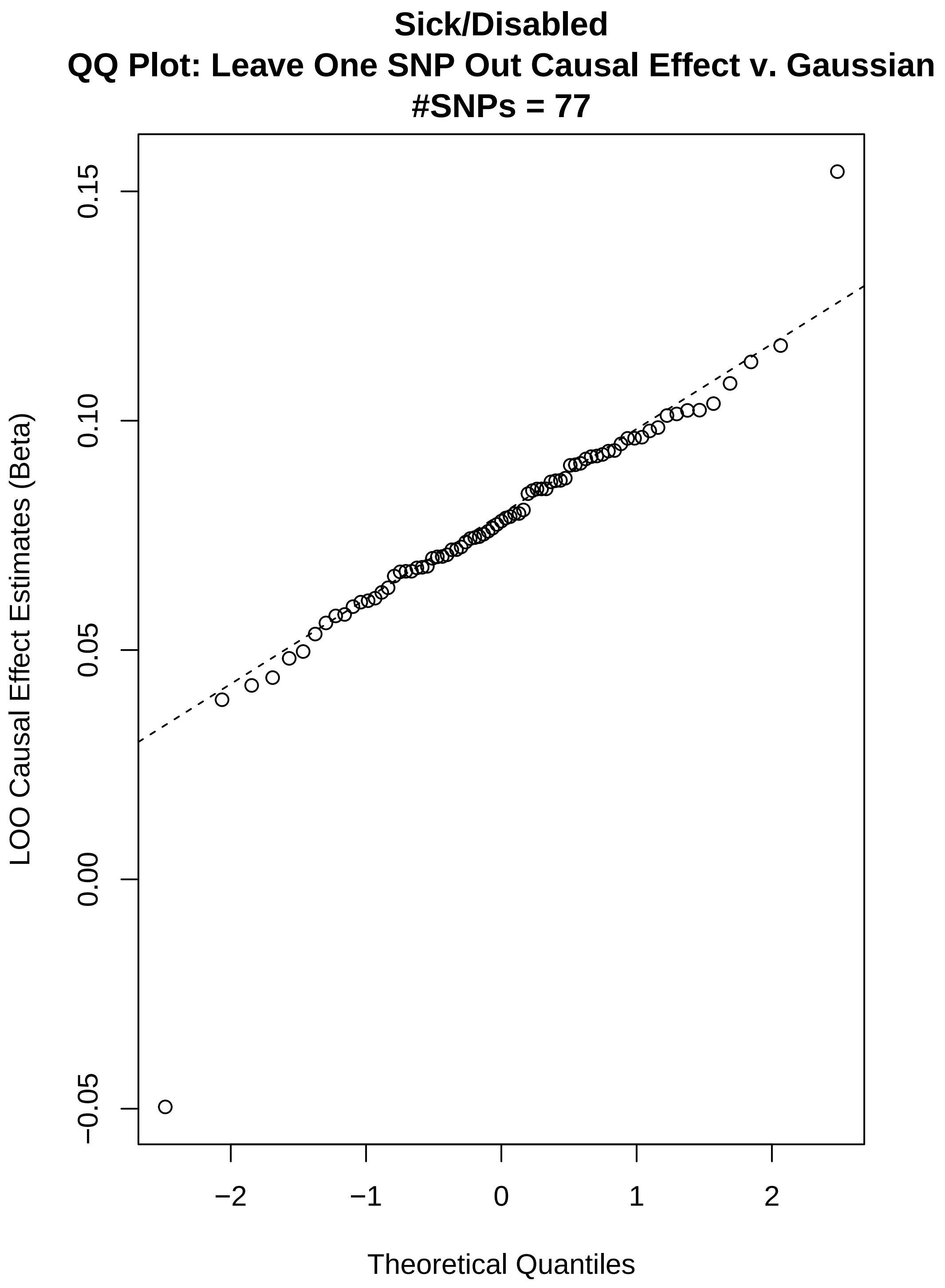
Sick/Disabled
QQ Plot: Leave One SNP Out Causal Effect v. Gaussian
#SNPs = 77
LOO Causal Effect Estimates (Beta)
Theoretical Quantiles
−0.05
0.00
0.05
0.10
0.15
−2
−1
0
1
2

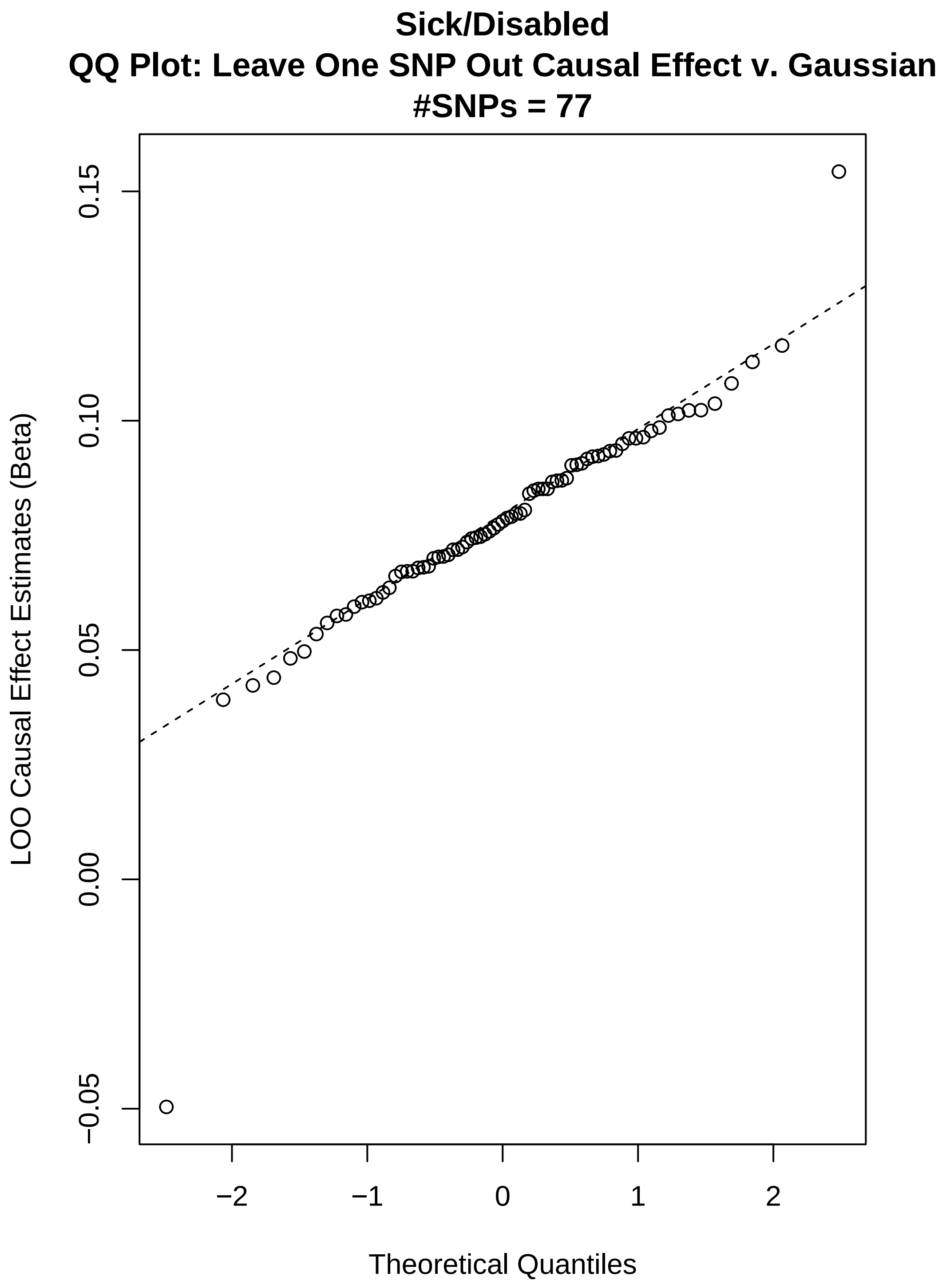
Sick/Disabled
QQ Plot: Leave One SNP Out Causal Effect v. Gaussian
#SNPs = 77
LOO Causal Effect Estimates (Beta)
Theoretical Quantiles
−0.05
0.00
0.05
0.10
0.15
−2
−1
0
1
2

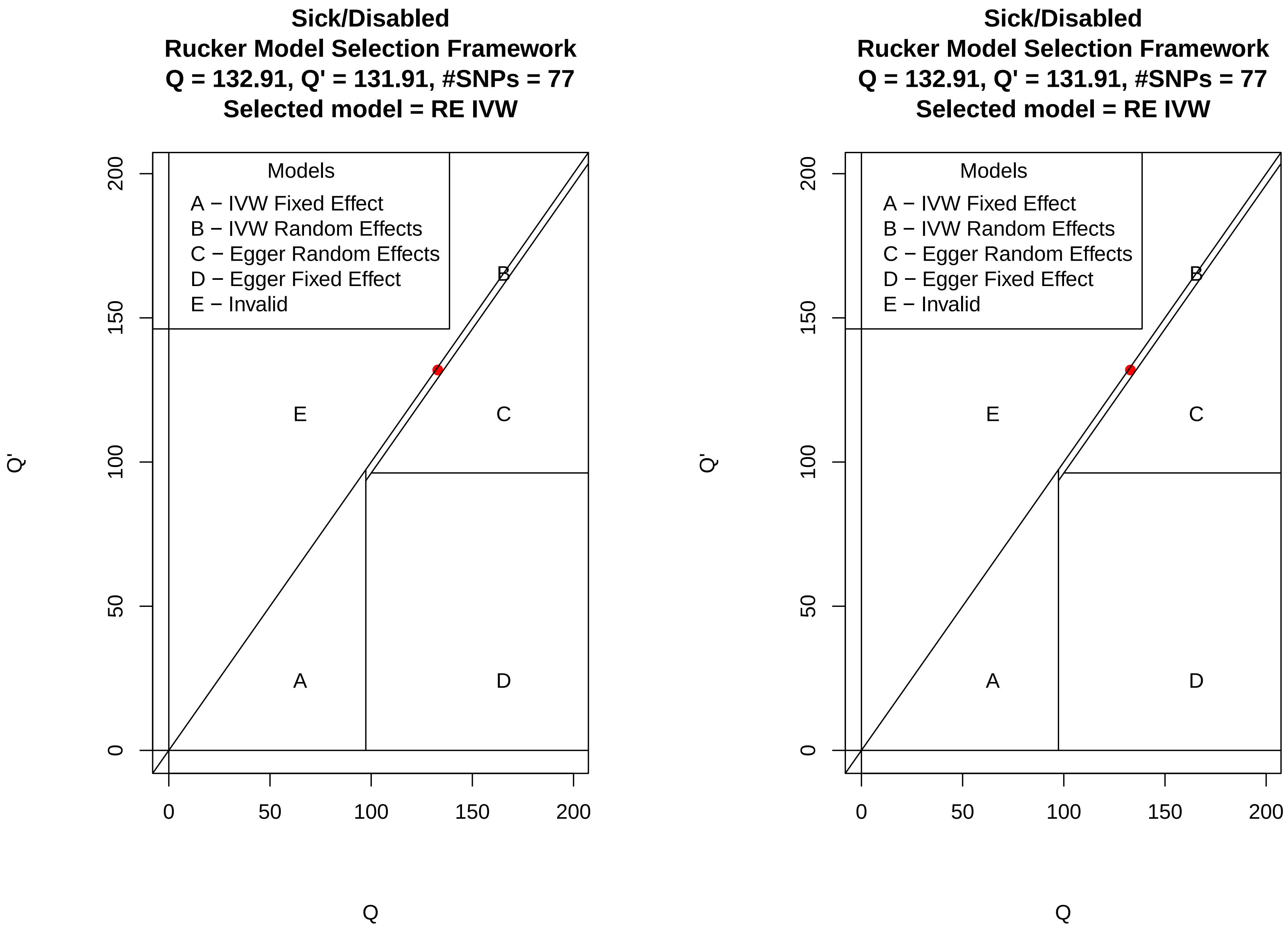
Sick/Disabled
Rucker Model Selection Framework
Q = 132.91, Q' = 131.91, #SNPs = 77
Selected model = RE IVW
Models
A − IVW Fixed Effect
B − IVW Random Effects
C − Egger Random Effects
D − Egger Fixed Effect
E − Invalid
A
B
C
D
E
Q
Q'
0
50
100
150
200
0
50
100
150
200
Sick/Disabled
Rucker Model Selection Framework
Q = 132.91, Q' = 131.91, #SNPs = 77
Selected model = RE IVW
Models
A − IVW Fixed Effect
B − IVW Random Effects
C − Egger Random Effects
D − Egger Fixed Effect
E − Invalid
A
B
C
D
E
Q
Q'
0
50
100
150
200
0
50
100
150
200

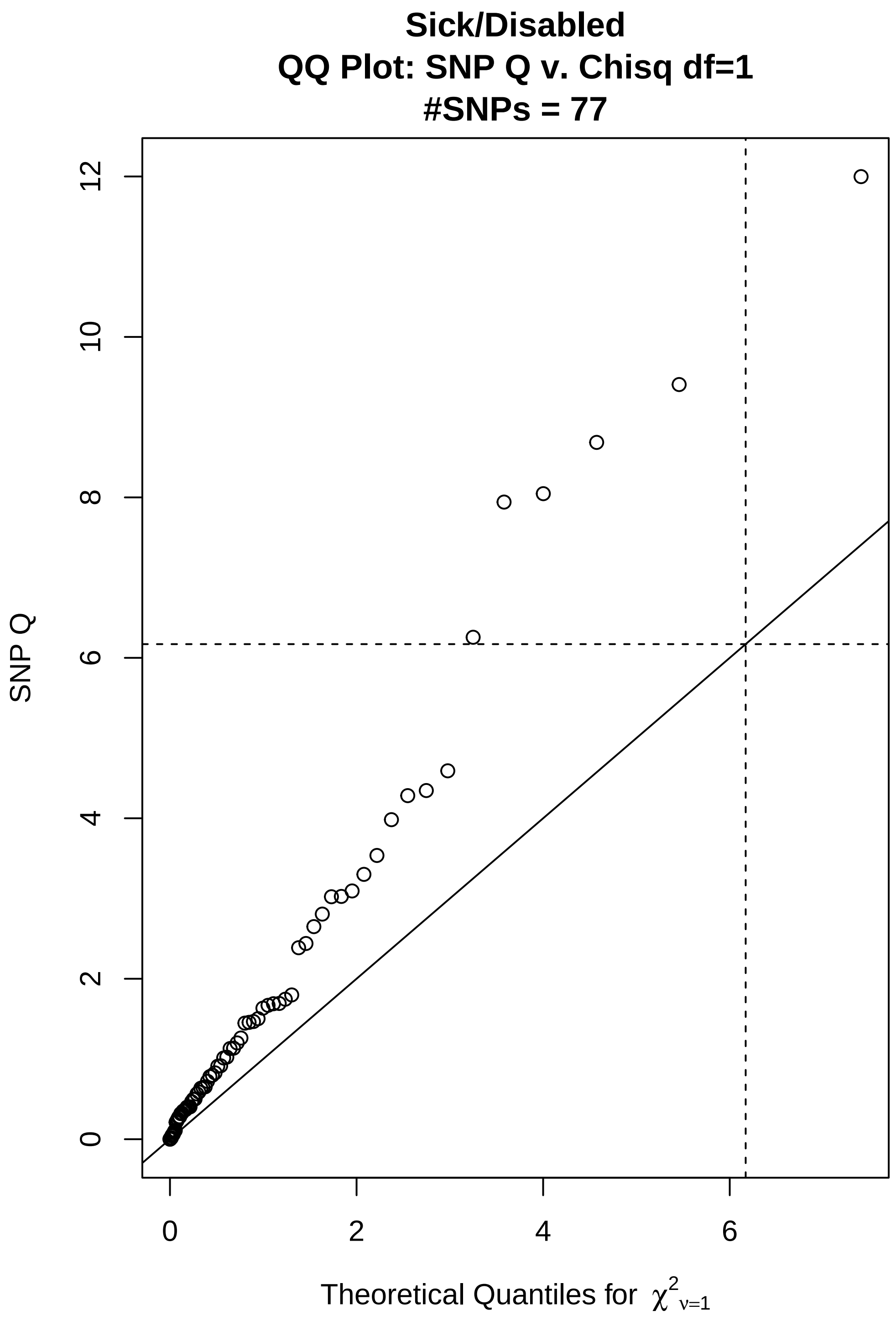
Sick/Disabled
QQ Plot: SNP Q v. Chisq df=1
#SNPs = 77
SNP Q
0
2
4
6
8
10
12
Theoretical Quantiles for $\chi^2_{\nu=1}$

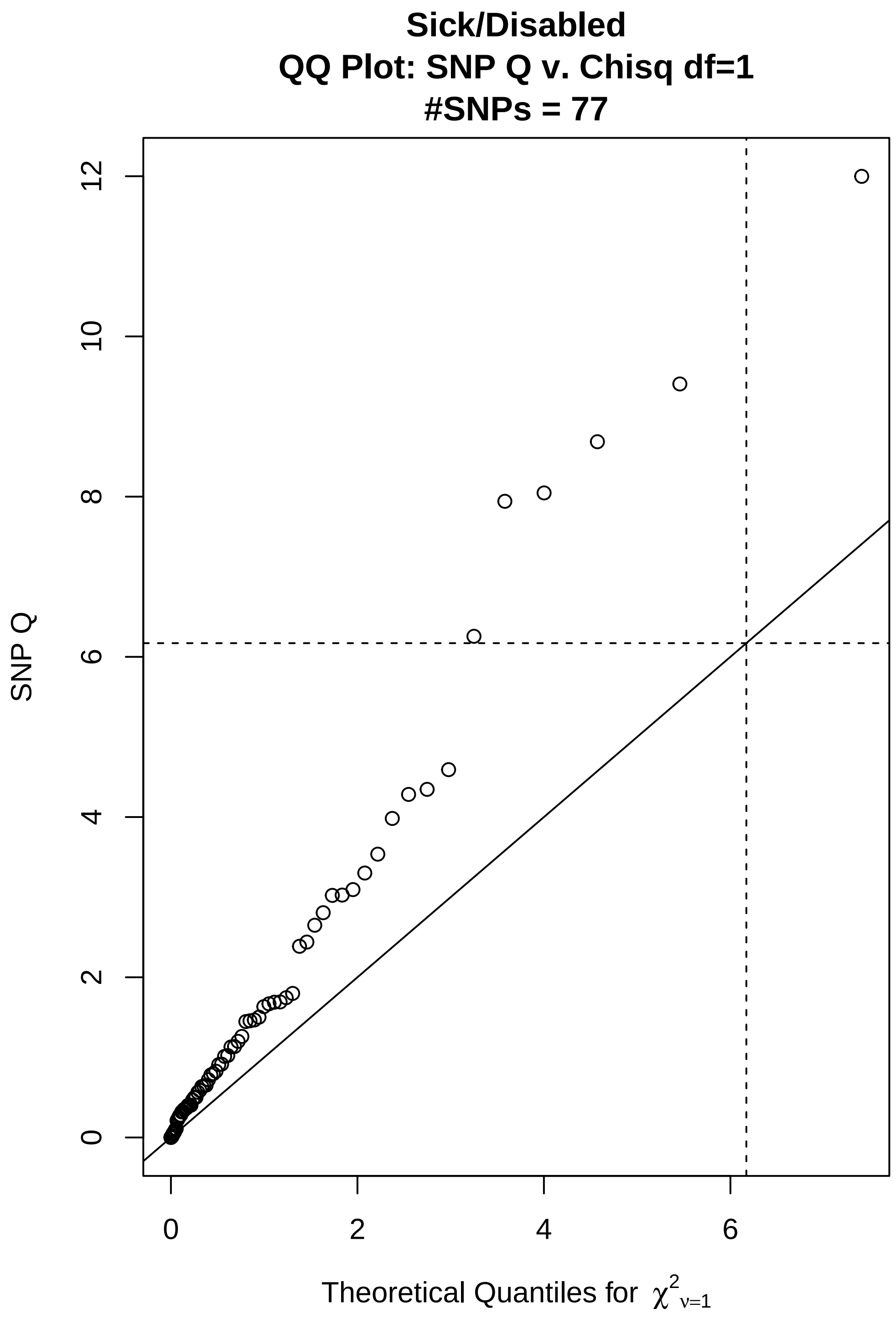
Sick/Disabled
QQ Plot: SNP Q v. Chisq df=1
#SNPs = 77
SNP Q
0
2
4
6
8
10
12
Theoretical Quantiles for $\chi^2_{\nu=1}$
