# Supplementary material for: Effects of alcohol consumption on employment and social outcomes: a Mendelian randomisation study
Source: Alcohol Alcohol. 2025 Jul 18;60(5):agaf038. doi: 10.1093/alcalc/agaf038 (PMC12271571; doi:10.1093/alcalc/agaf038)
Supplement: Campbell_Green_Davies_et_al_2025_agaf038 [file campbell_green_davies_et_al_2025_agaf038.zip › Campbell_Green_Davies_et_al_2025/Female/aud/do2SampleMrAnalyses_bAlcoholUseDisorder_highestEducAttainment_ageCentreGpc.pdf]

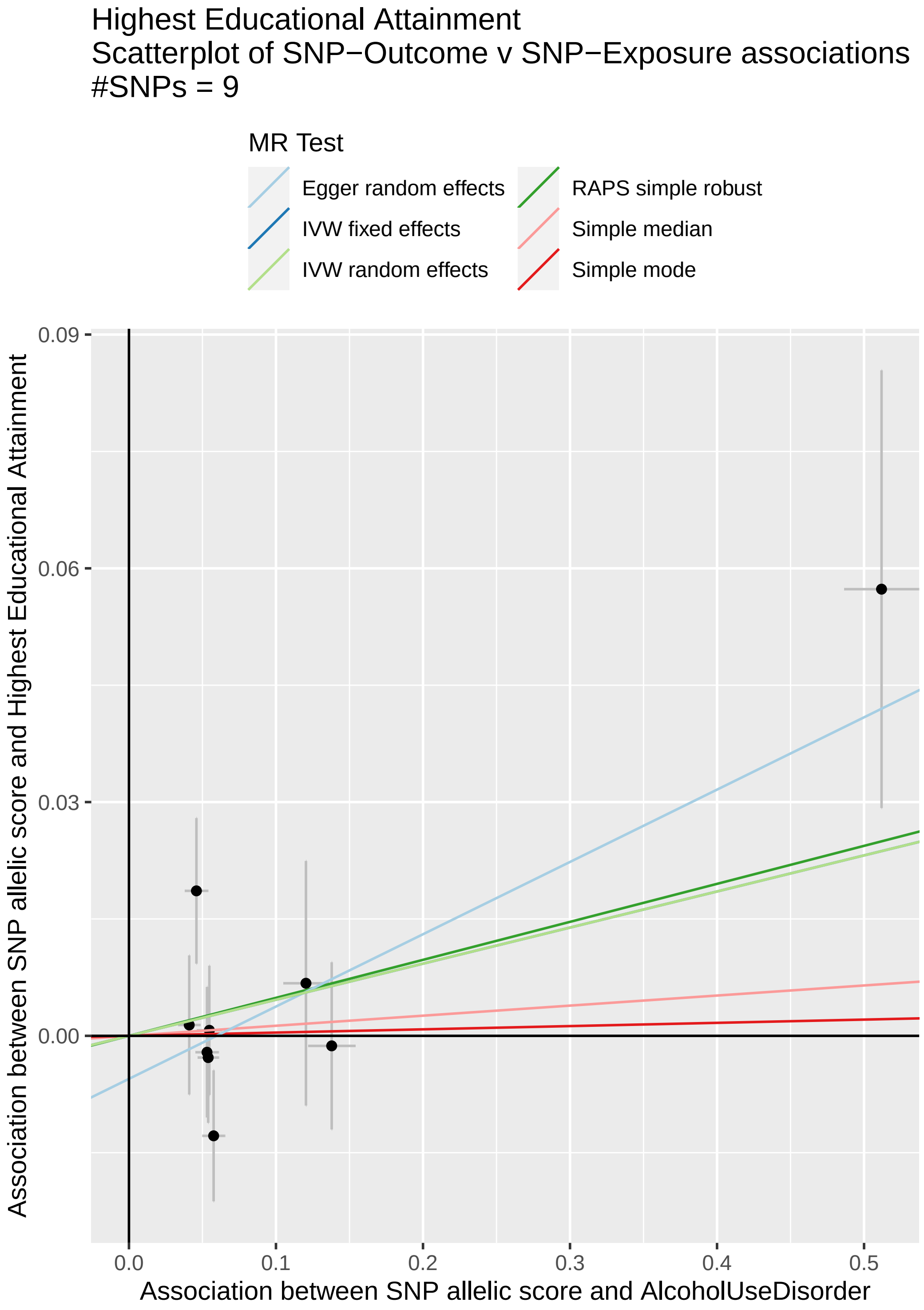
Highest Educational Attainment
Scatterplot of SNP−Outcome v SNP−Exposure associations
#SNPs = 9
MR Test
Egger random effects
IVW fixed effects
IVW random effects
RAPS simple robust
Simple median
Simple mode
0.09
0.06
0.03
0.00
0.0
0.1
0.2
0.3
0.4
0.5
Association between SNP allelic score and Highest Educational Attainment
Association between SNP allelic score and AlcoholUseDisorder

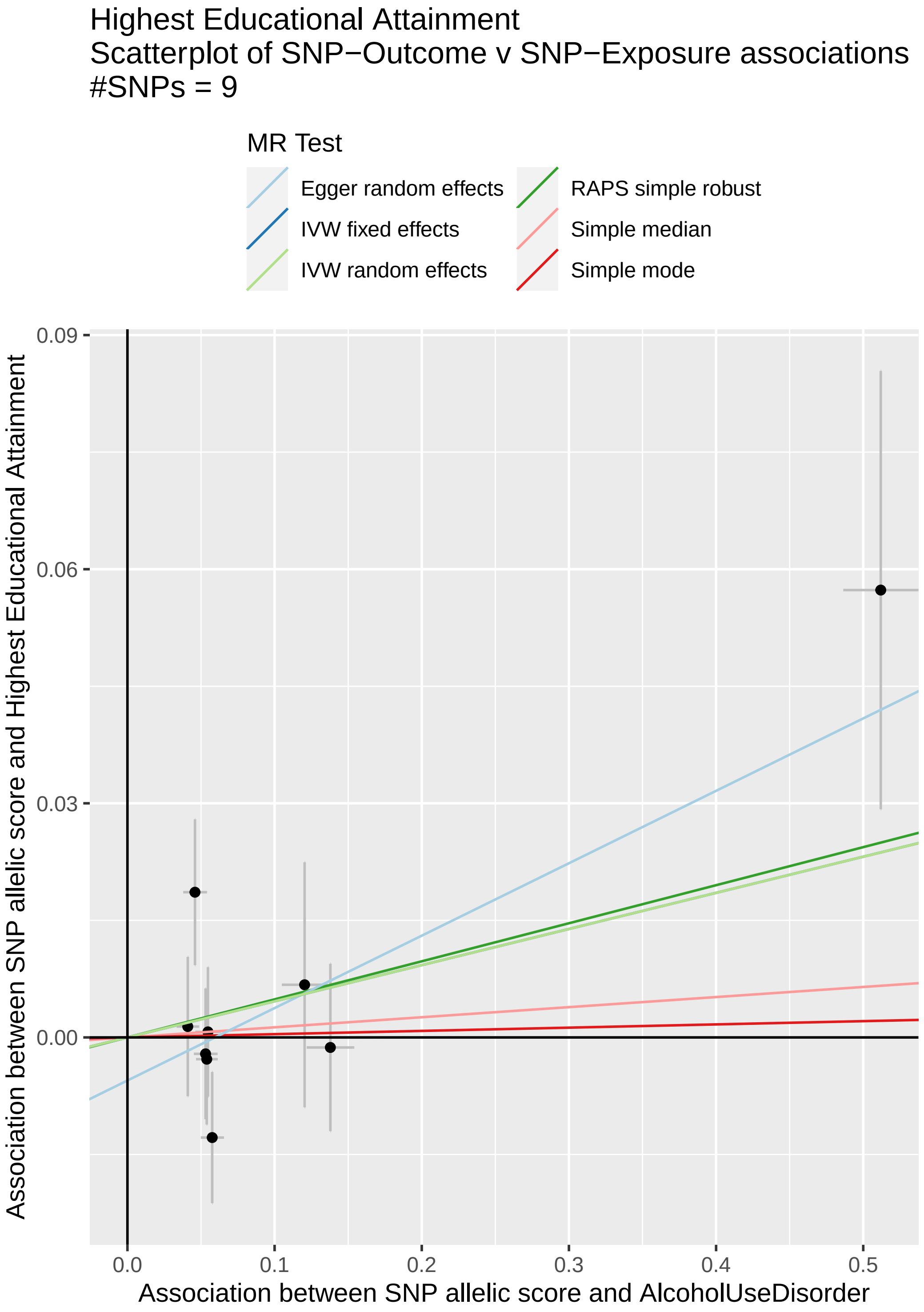
Highest Educational Attainment
Scatterplot of SNP−Outcome v SNP−Exposure associations
#SNPs = 9
MR Test
Egger random effects
IVW fixed effects
IVW random effects
RAPS simple robust
Simple median
Simple mode
0.09
0.06
0.03
0.00
0.0
0.1
0.2
0.3
0.4
0.5
Association between SNP allelic score and Highest Educational Attainment
Association between SNP allelic score and AlcoholUseDisorder

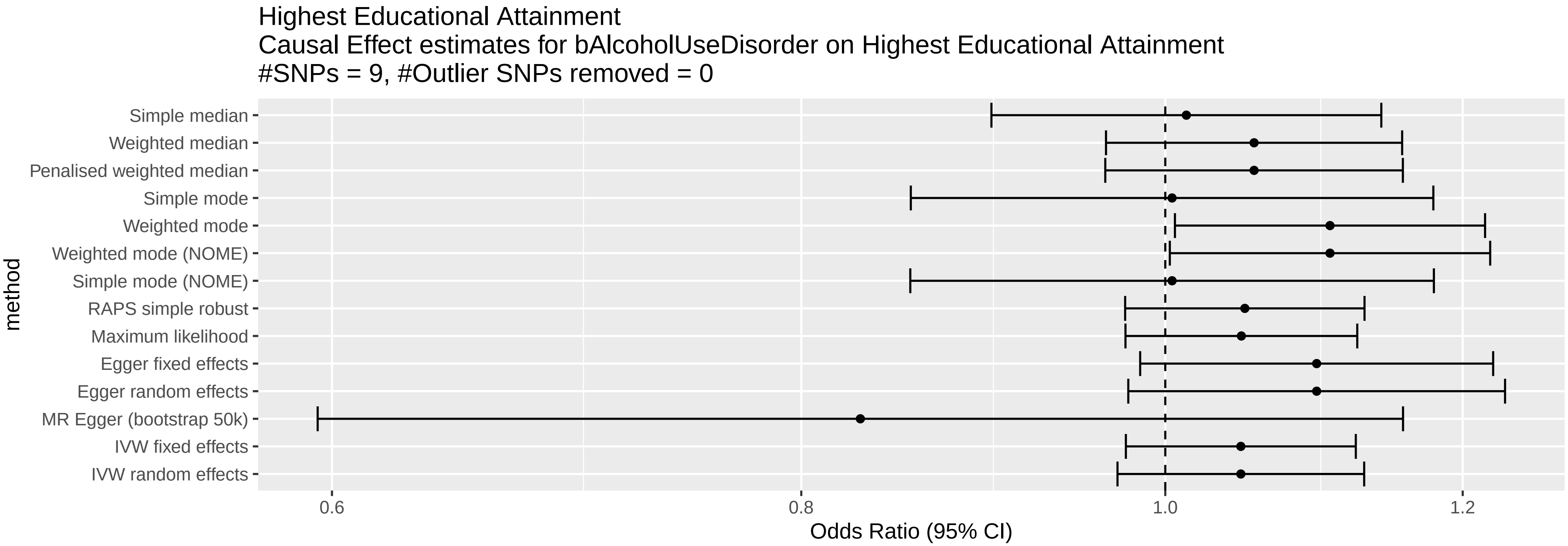
Highest Educational Attainment
Causal Effect estimates for bAlcoholUseDisorder on Highest Educational Attainment
#SNPs = 9, #Outlier SNPs removed = 0
method
Simple median
Weighted median
Penalised weighted median
Simple mode
Weighted mode
Weighted mode (NOME)
Simple mode (NOME)
RAPS simple robust
Maximum likelihood
Egger fixed effects
Egger random effects
MR Egger (bootstrap 50k)
IVW fixed effects
IVW random effects
0.6
0.8
1.0
1.2
Odds Ratio (95% CI)

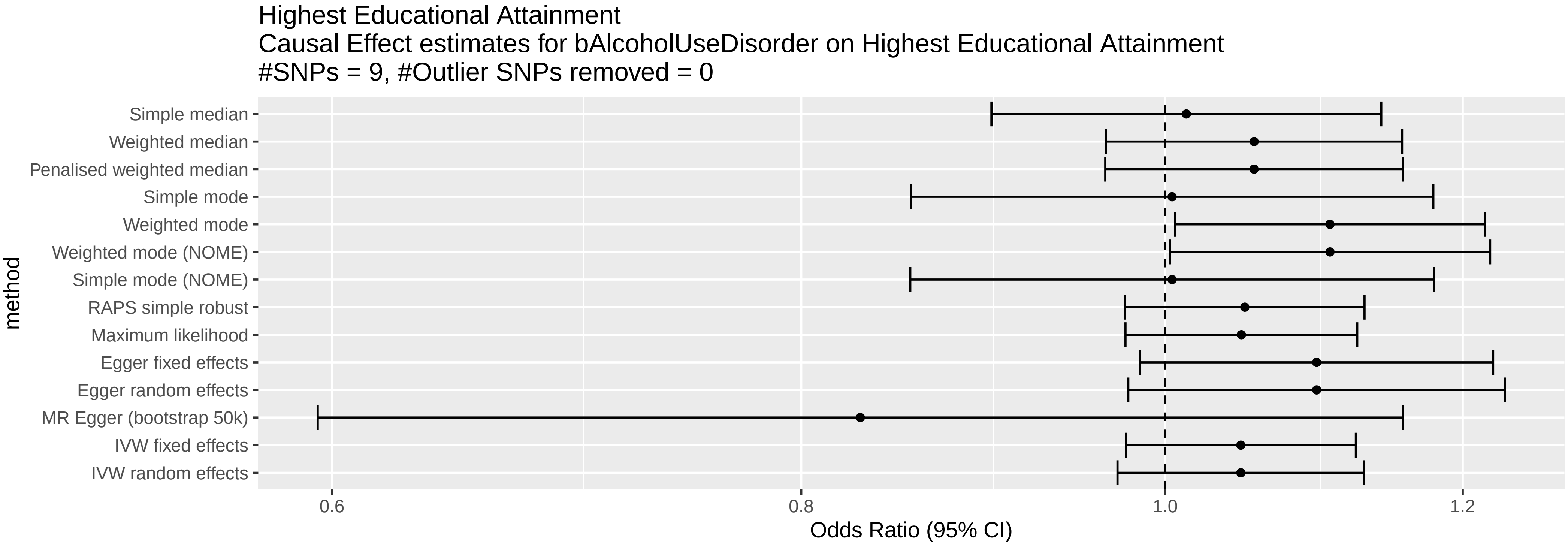
Highest Educational Attainment
Causal Effect estimates for bAlcoholUseDisorder on Highest Educational Attainment
#SNPs = 9, #Outlier SNPs removed = 0
method
Simple median
Weighted median
Penalised weighted median
Simple mode
Weighted mode
Weighted mode (NOME)
Simple mode (NOME)
RAPS simple robust
Maximum likelihood
Egger fixed effects
Egger random effects
MR Egger (bootstrap 50k)
IVW fixed effects
IVW random effects
0.6
0.8
1.0
1.2
Odds Ratio (95% CI)

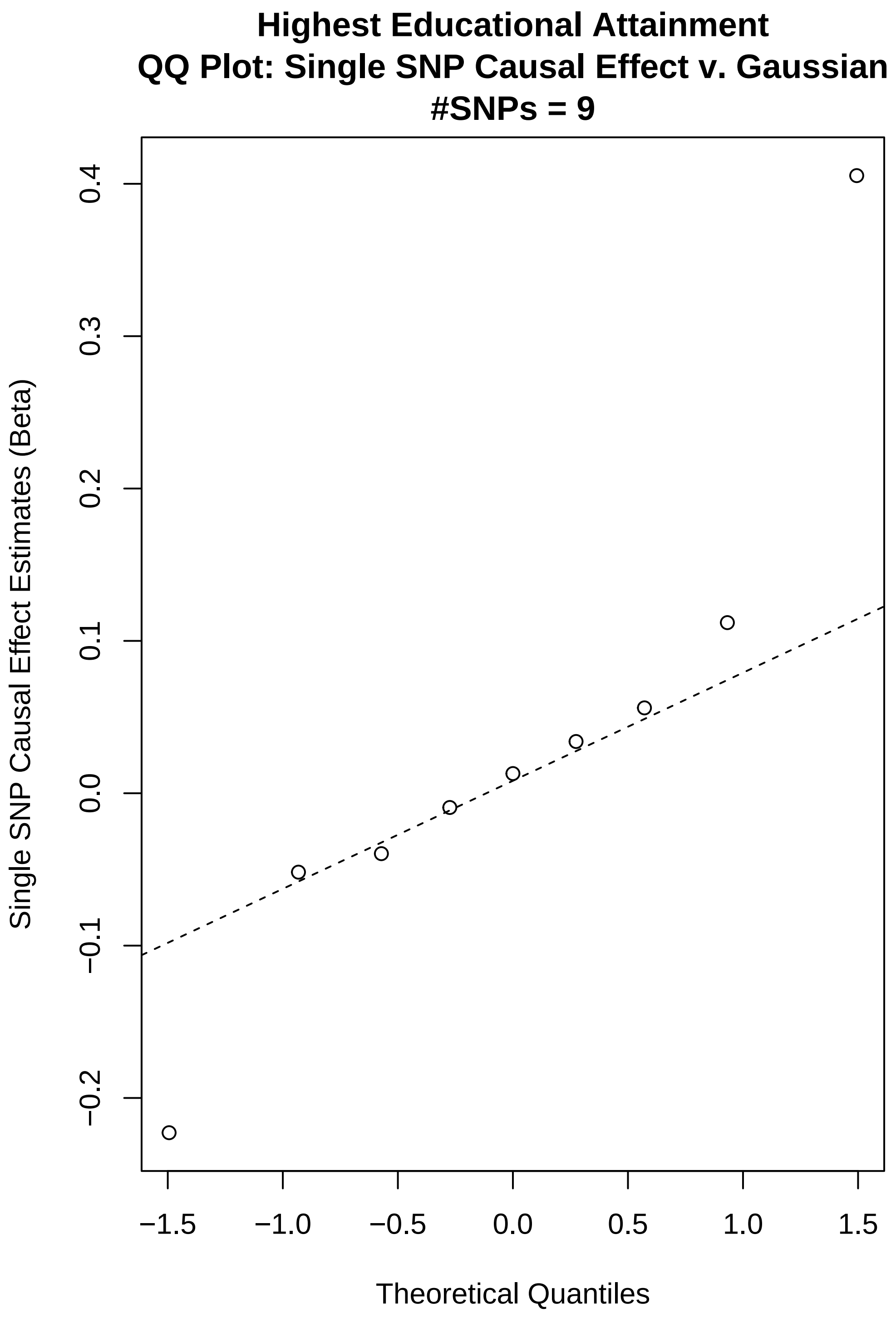
Highest Educational Attainment
QQ Plot: Single SNP Causal Effect v. Gaussian
#SNPs = 9
Single SNP Causal Effect Estimates (Beta)
Theoretical Quantiles
−0.2
−0.1
0.0
0.1
0.2
0.3
0.4
−1.5
−1.0
−0.5
0.0
0.5
1.0
1.5

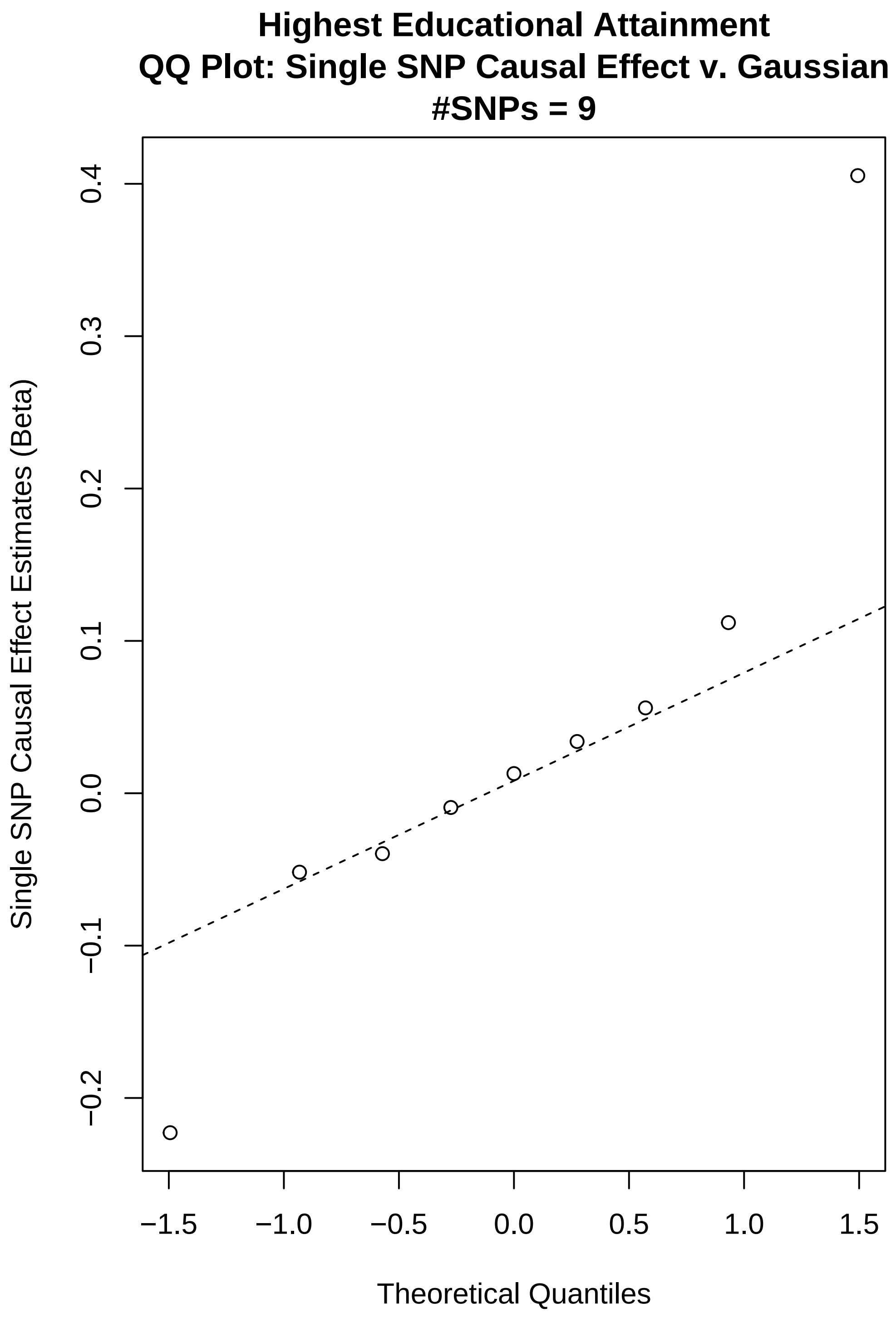
Highest Educational Attainment
QQ Plot: Single SNP Causal Effect v. Gaussian
#SNPs = 9
Single SNP Causal Effect Estimates (Beta)
Theoretical Quantiles
−0.2
−0.1
0.0
0.1
0.2
0.3
0.4
−1.5
−1.0
−0.5
0.0
0.5
1.0
1.5

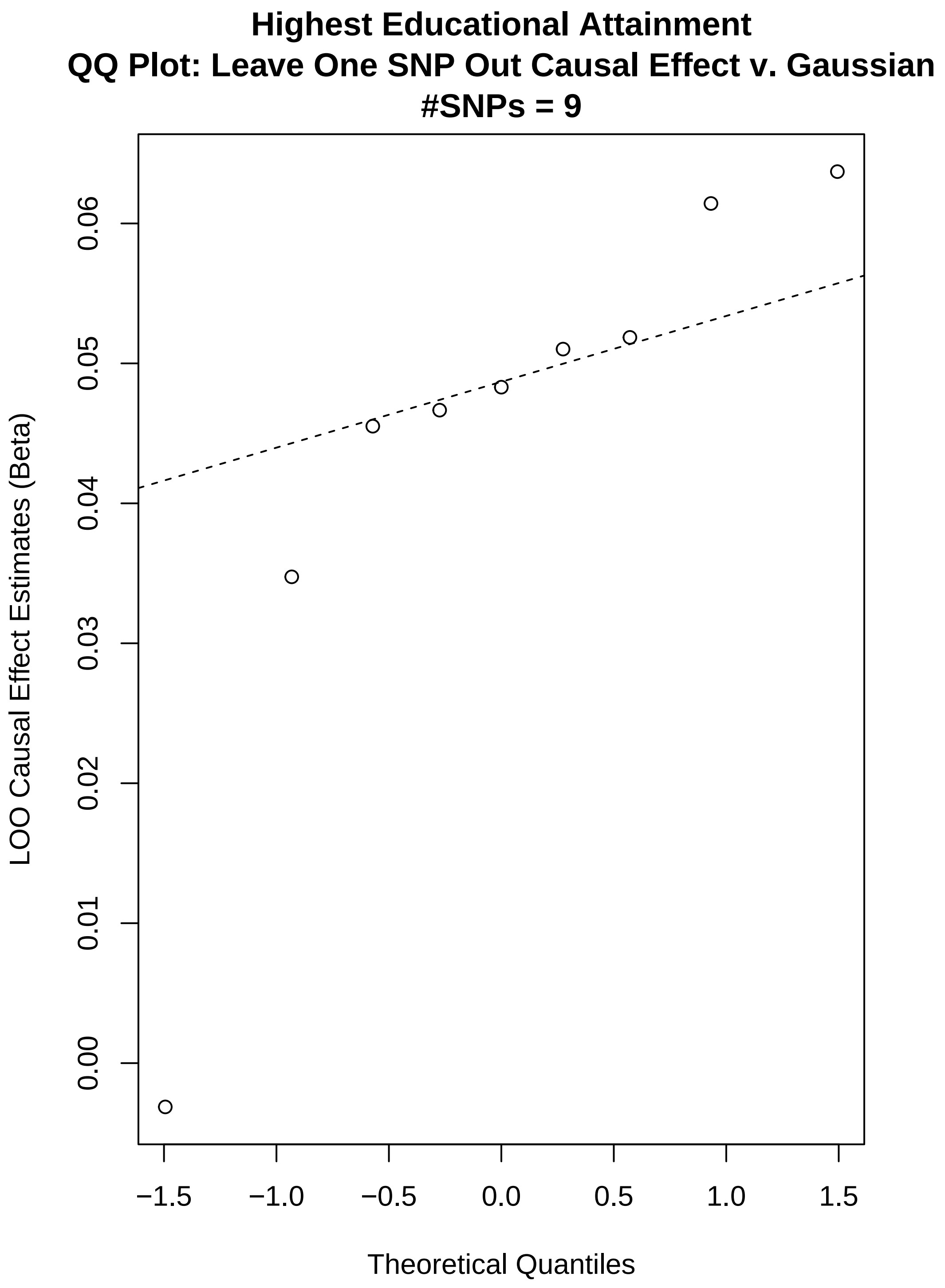
Highest Educational Attainment
QQ Plot: Leave One SNP Out Causal Effect v. Gaussian
#SNPs = 9
LOO Causal Effect Estimates (Beta)
0.00
0.01
0.02
0.03
0.04
0.05
0.06
−1.5
−1.0
−0.5
0.0
0.5
1.0
1.5
Theoretical Quantiles

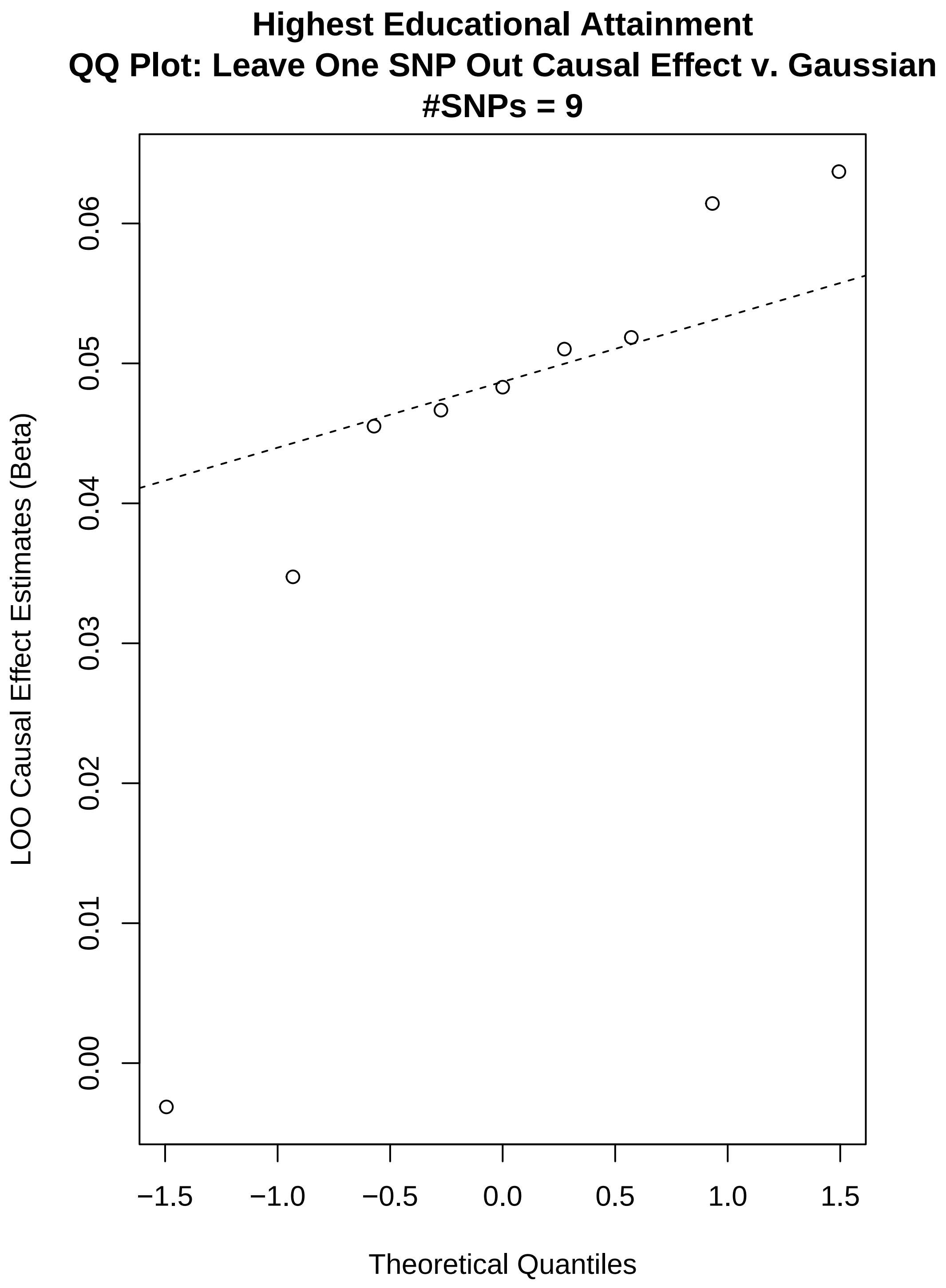
Highest Educational Attainment
QQ Plot: Leave One SNP Out Causal Effect v. Gaussian
#SNPs = 9
LOO Causal Effect Estimates (Beta)
0.00
0.01
0.02
0.03
0.04
0.05
0.06
−1.5
−1.0
−0.5
0.0
0.5
1.0
1.5
Theoretical Quantiles

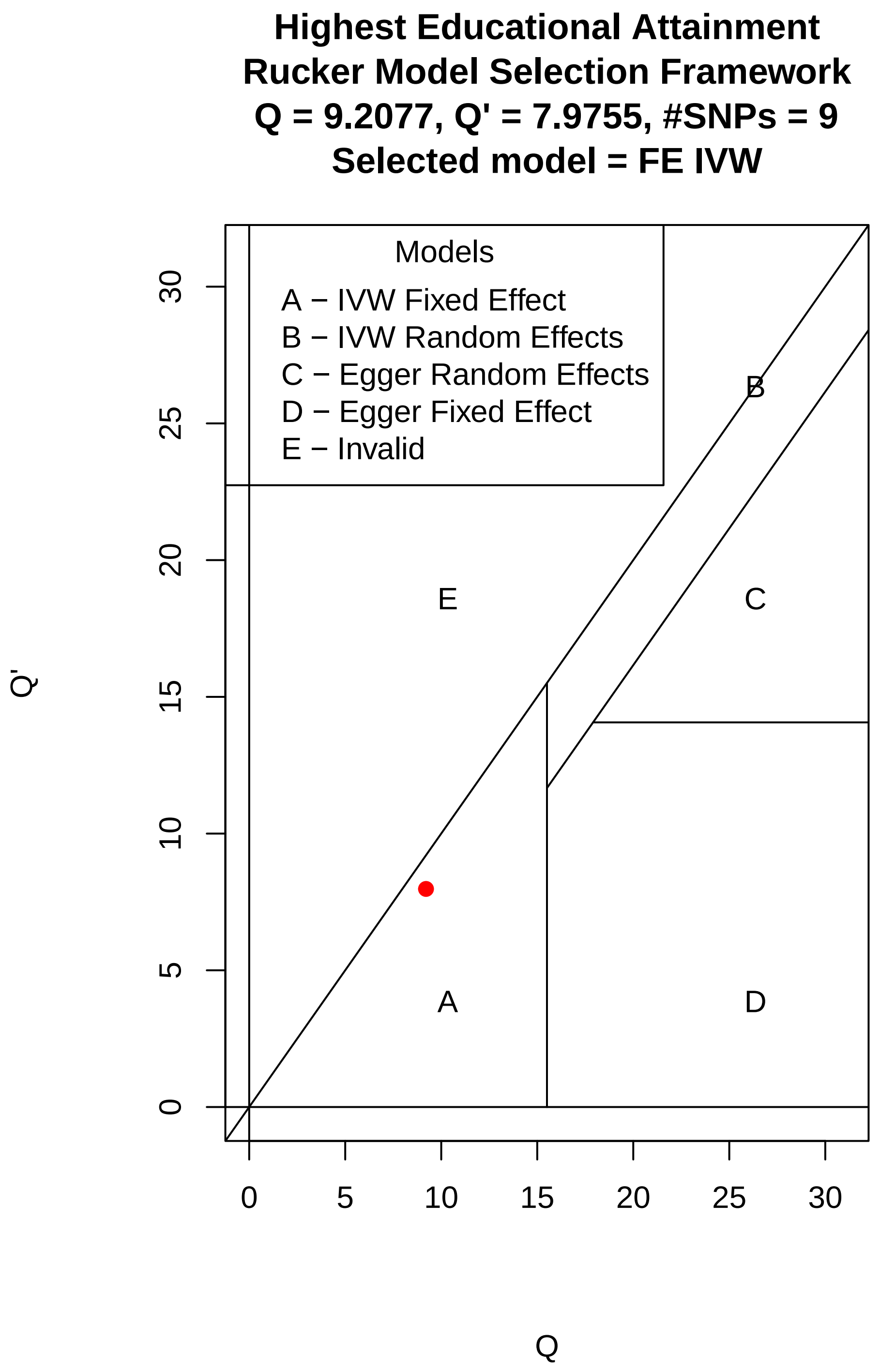

Highest Educational Attainment
Rucker Model Selection Framework
Q = 9.2077, Q' = 7.9755, #SNPs = 9
Selected model = FE IVW
Models
A − IVW Fixed Effect
B − IVW Random Effects
C − Egger Random Effects
D − Egger Fixed Effect
E − Invalid
A
B
C
D
E
Q
Q'

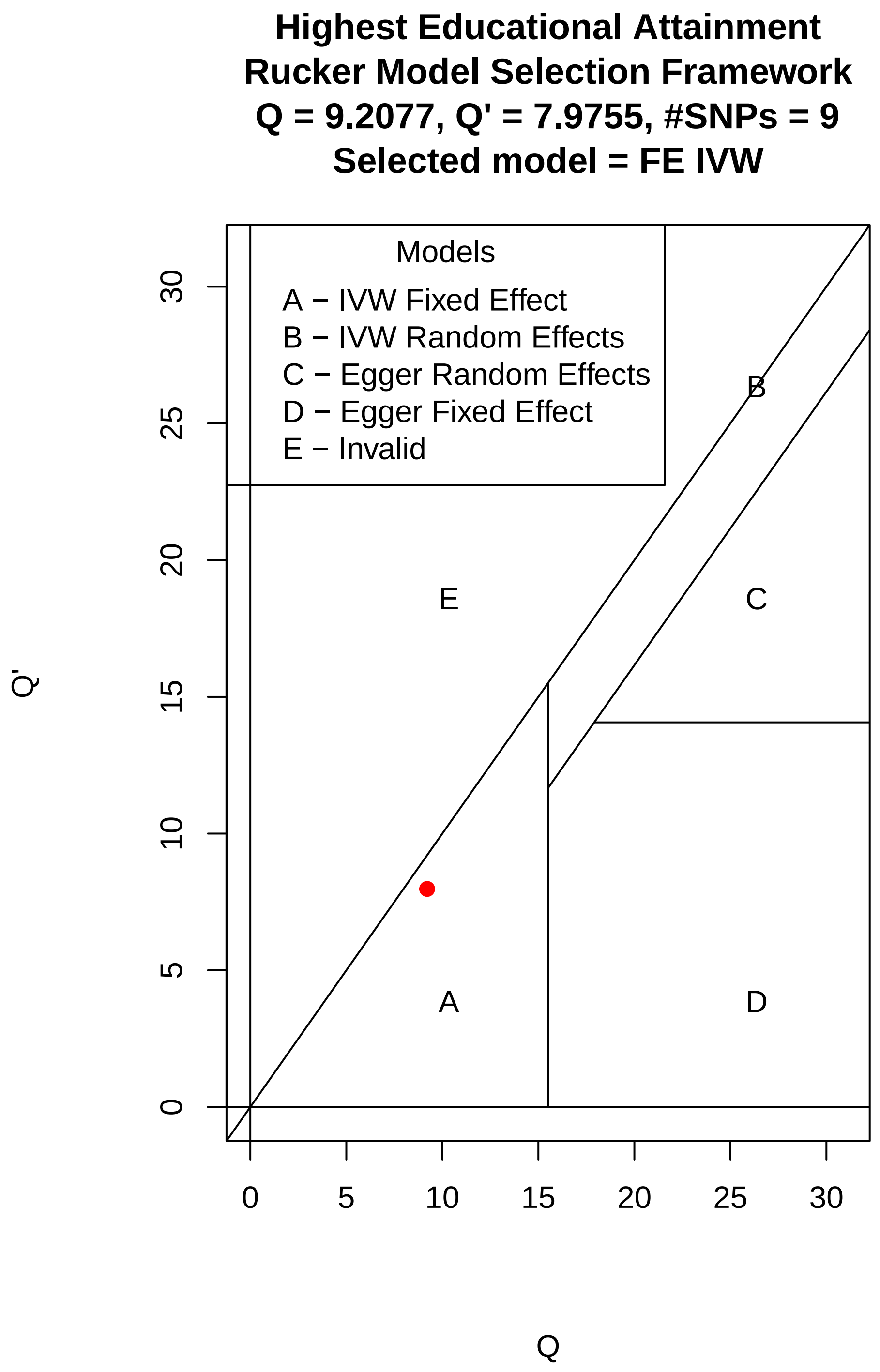

Highest Educational Attainment
Rucker Model Selection Framework
Q = 9.2077, Q' = 7.9755, #SNPs = 9
Selected model = FE IVW
Models
A − IVW Fixed Effect
B − IVW Random Effects
C − Egger Random Effects
D − Egger Fixed Effect
E − Invalid
A
B
C
D
E
Q
Q'

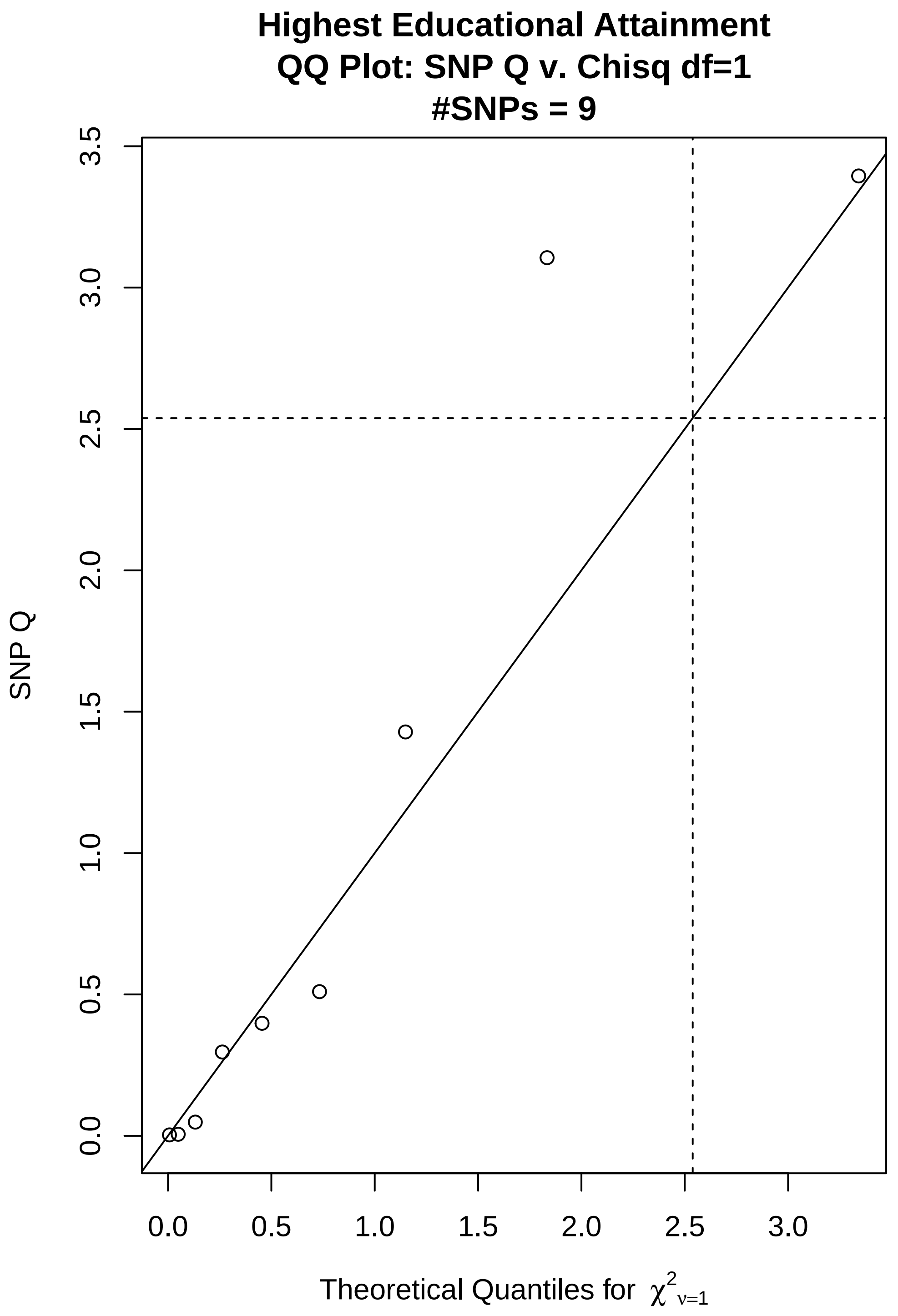
Highest Educational Attainment
QQ Plot: SNP Q v. Chisq df=1
#SNPs = 9
SNP Q
Theoretical Quantiles for $\chi^2_{\nu=1}$
0.0
0.5
1.0
1.5
2.0
2.5
3.0
3.5

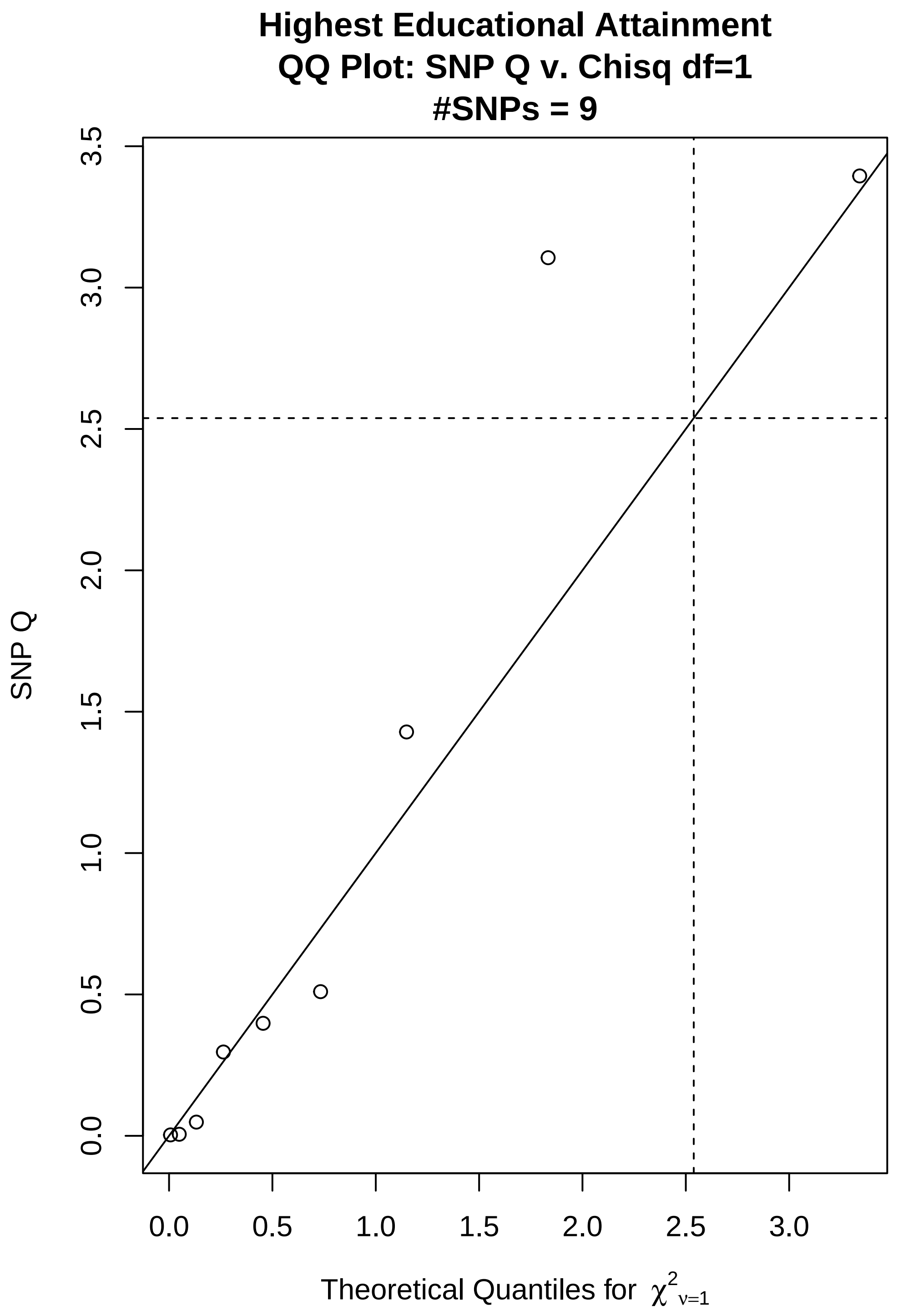
Highest Educational Attainment
QQ Plot: SNP Q v. Chisq df=1
#SNPs = 9
SNP Q
Theoretical Quantiles for $\chi^2_{\nu=1}$
0.0
0.5
1.0
1.5
2.0
2.5
3.0
3.5
