# Supplementary material for: Effects of alcohol consumption on employment and social outcomes: a Mendelian randomisation study
Source: Alcohol Alcohol. 2025 Jul 18;60(5):agaf038. doi: 10.1093/alcalc/agaf038 (PMC12271571; doi:10.1093/alcalc/agaf038)
Supplement: Campbell_Green_Davies_et_al_2025_agaf038 [file campbell_green_davies_et_al_2025_agaf038.zip › Campbell_Green_Davies_et_al_2025/Female/aud/do2SampleMrAnalyses_bAlcoholUseDisorder_iUnempNotEmp_ageCentreGpc.pdf]

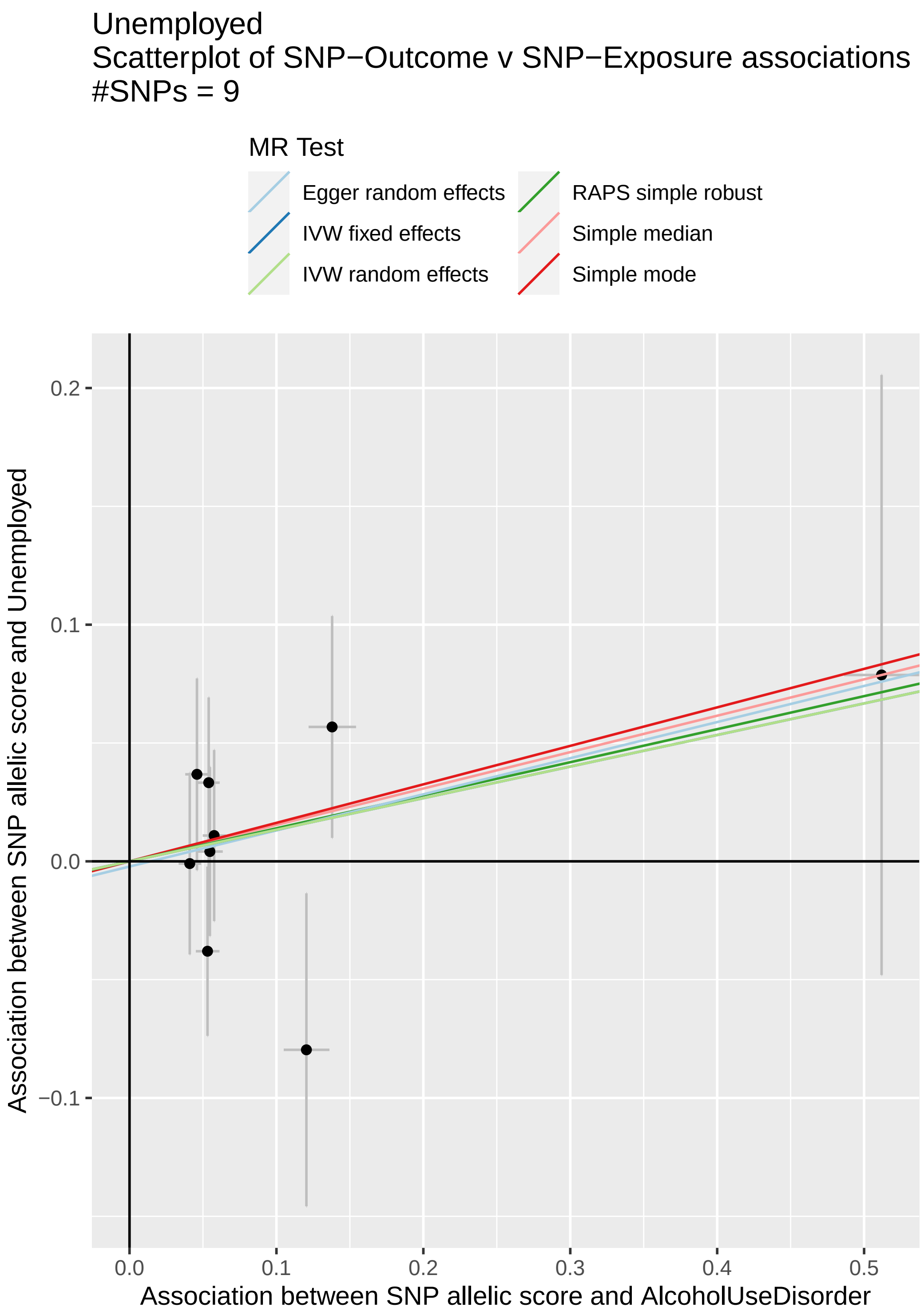

Unemployed
Scatterplot of SNP−Outcome v SNP−Exposure associations
#SNPs = 9
MR Test
Egger random effects
IVW fixed effects
IVW random effects
RAPS simple robust
Simple median
Simple mode
0.2
0.1
0.0
−0.1
0.0
0.1
0.2
0.3
0.4
0.5
Association between SNP allelic score and Unemployed
Association between SNP allelic score and AlcoholUseDisorder

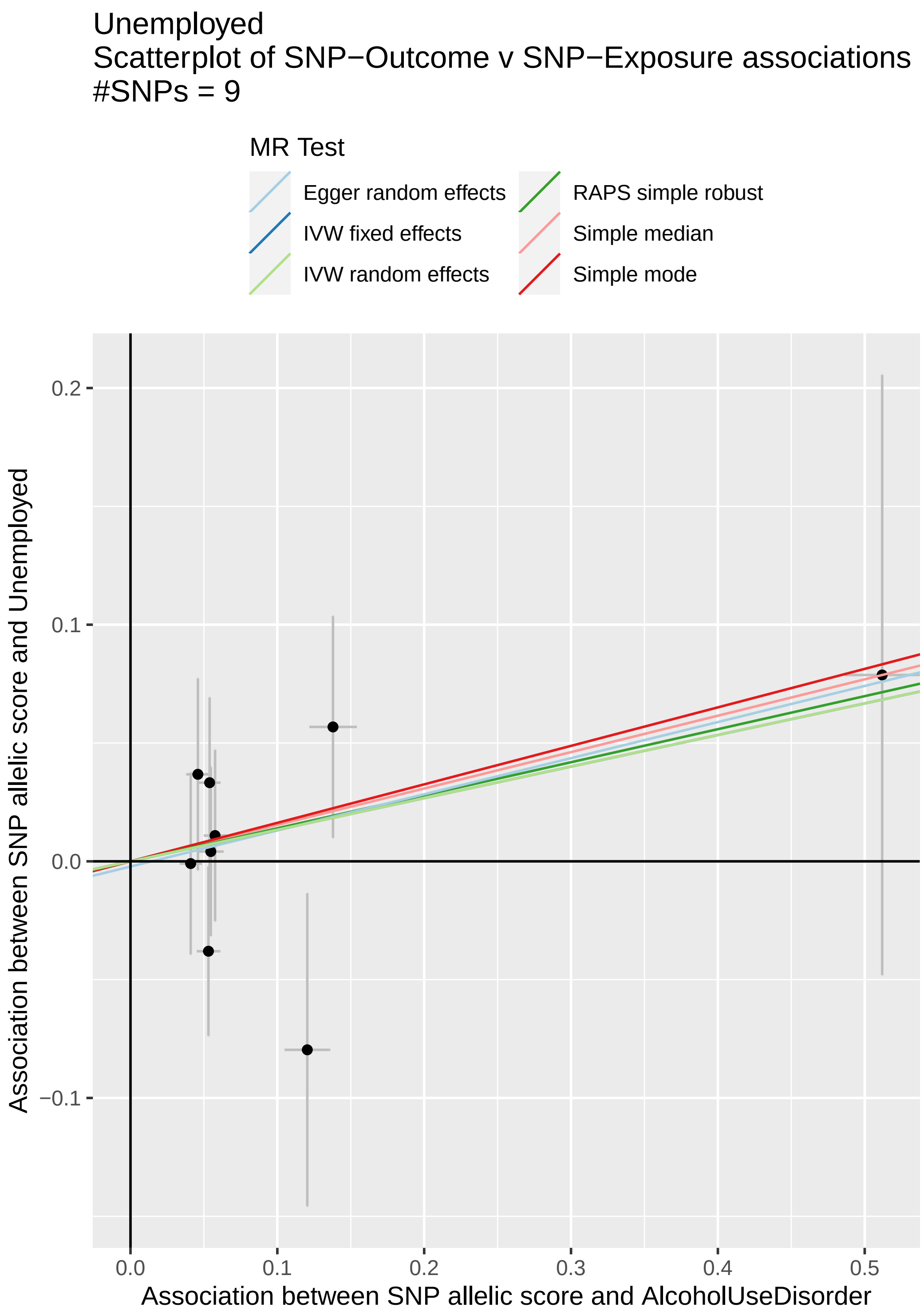

Unemployed
Scatterplot of SNP−Outcome v SNP−Exposure associations
#SNPs = 9
MR Test
Egger random effects
IVW fixed effects
IVW random effects
RAPS simple robust
Simple median
Simple mode
0.2
0.1
0.0
−0.1
0.0
0.1
0.2
0.3
0.4
0.5
Association between SNP allelic score and Unemployed
Association between SNP allelic score and AlcoholUseDisorder

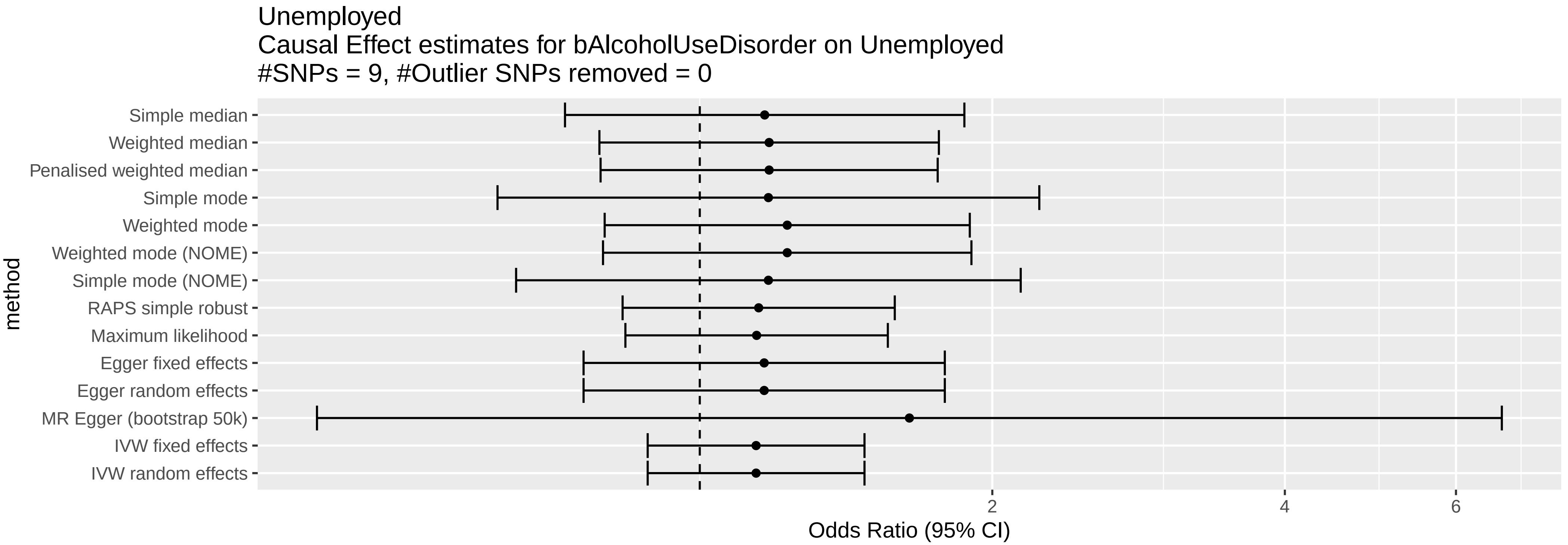
Unemployed
Causal Effect estimates for bAlcoholUseDisorder on Unemployed
#SNPs = 9, #Outlier SNPs removed = 0
method
Simple median
Weighted median
Penalised weighted median
Simple mode
Weighted mode
Weighted mode (NOME)
Simple mode (NOME)
RAPS simple robust
Maximum likelihood
Egger fixed effects
Egger random effects
MR Egger (bootstrap 50k)
IVW fixed effects
IVW random effects
2
4
6
Odds Ratio (95% CI)

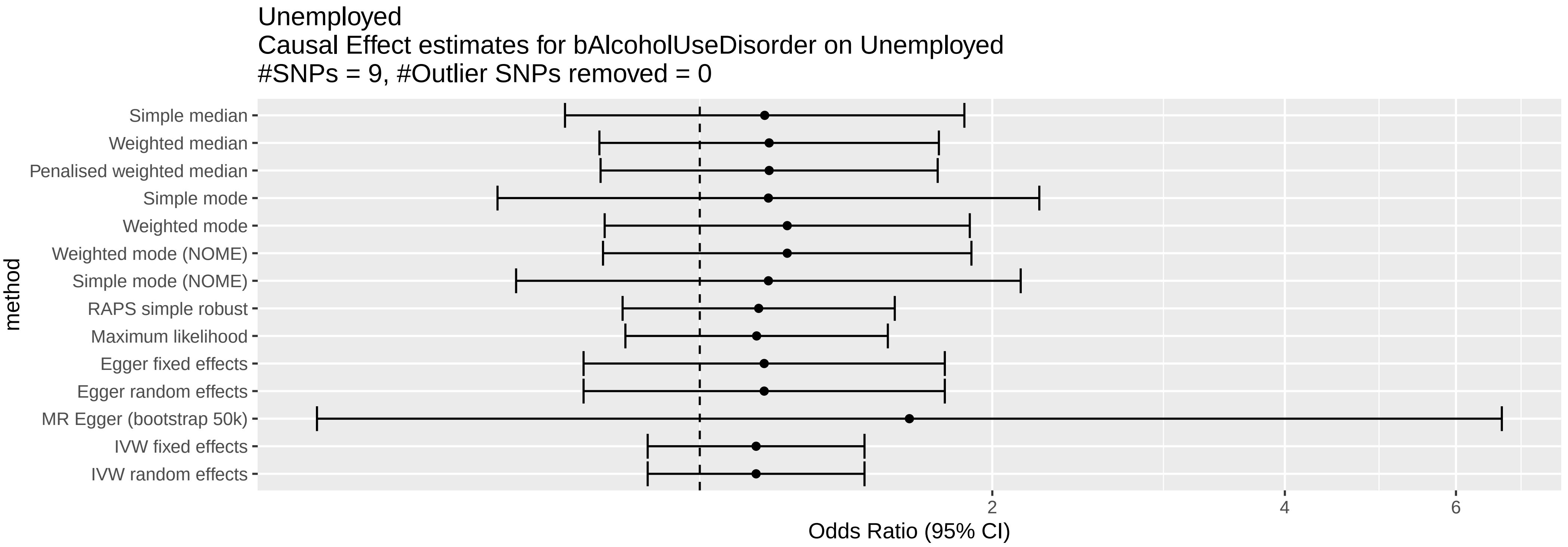
Unemployed
Causal Effect estimates for bAlcoholUseDisorder on Unemployed
#SNPs = 9, #Outlier SNPs removed = 0
method
Simple median
Weighted median
Penalised weighted median
Simple mode
Weighted mode
Weighted mode (NOME)
Simple mode (NOME)
RAPS simple robust
Maximum likelihood
Egger fixed effects
Egger random effects
MR Egger (bootstrap 50k)
IVW fixed effects
IVW random effects
2
4
6
Odds Ratio (95% CI)

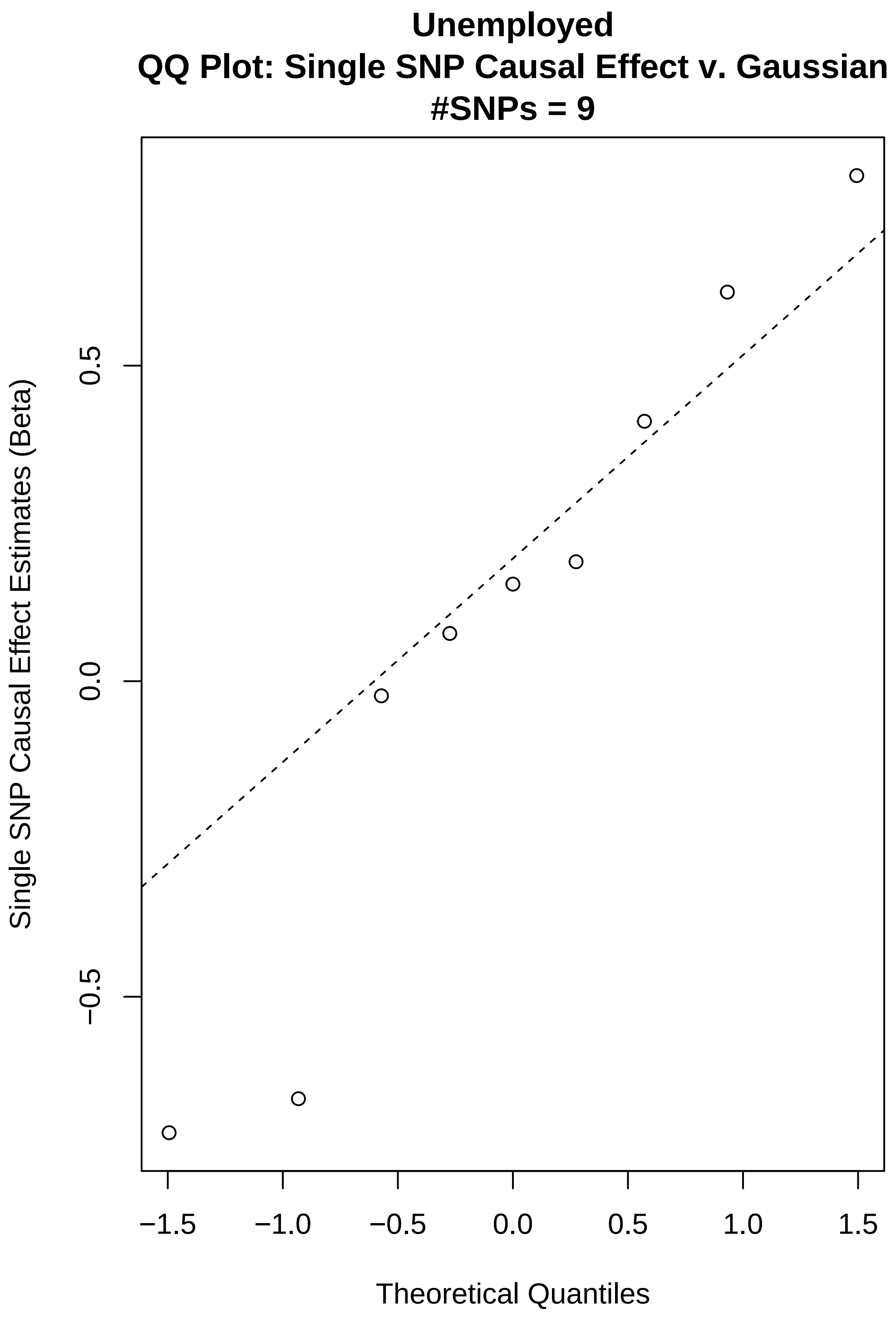

Unemployed
QQ Plot: Single SNP Causal Effect v. Gaussian
#SNPs = 9
Single SNP Causal Effect Estimates (Beta)
Theoretical Quantiles
−1.5
−1.0
−0.5
0.0
0.5
1.0
1.5
−0.5
0.0
0.5

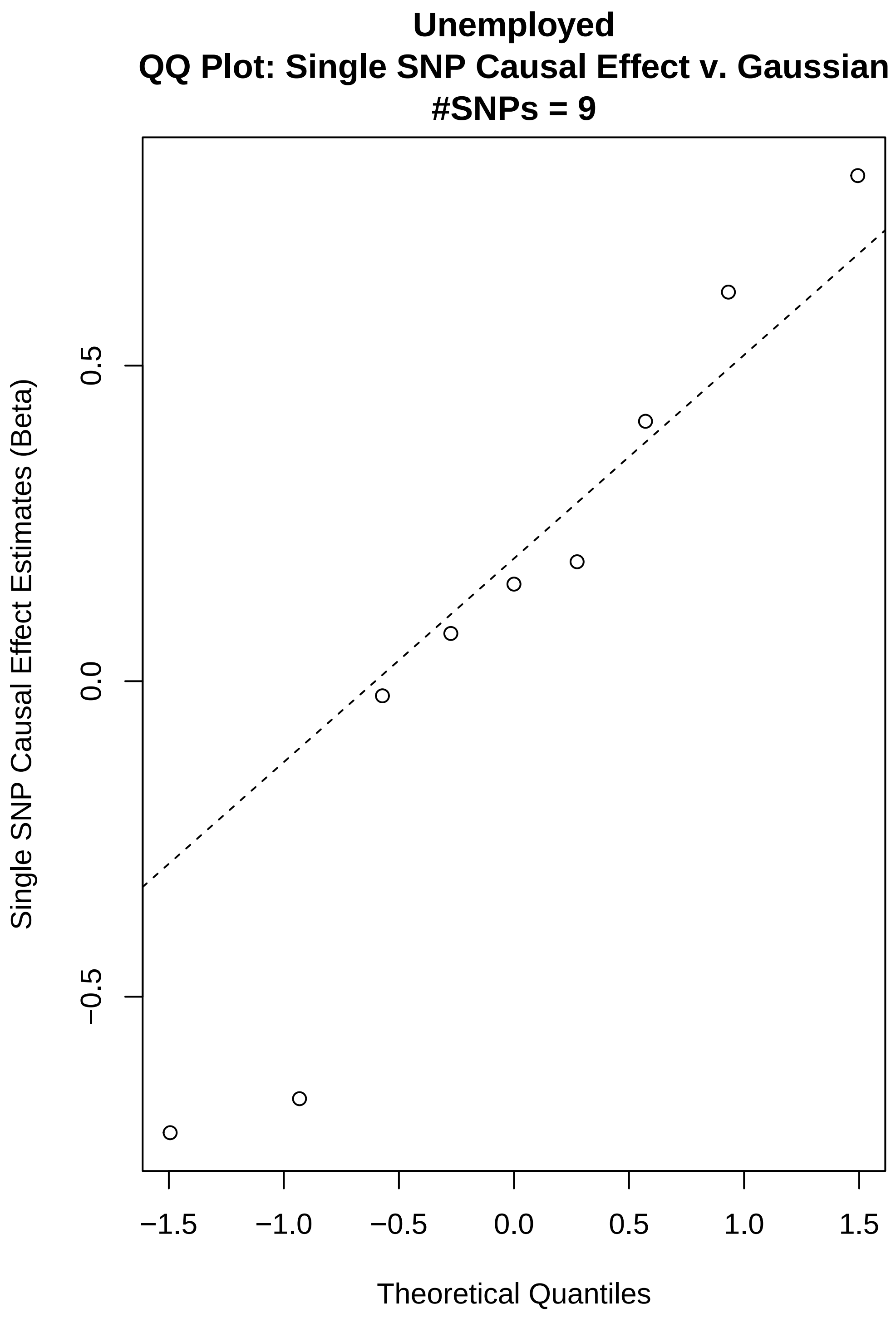

Unemployed
QQ Plot: Single SNP Causal Effect v. Gaussian
#SNPs = 9
Single SNP Causal Effect Estimates (Beta)
Theoretical Quantiles
−1.5
−1.0
−0.5
0.0
0.5
1.0
1.5
−0.5
0.0
0.5

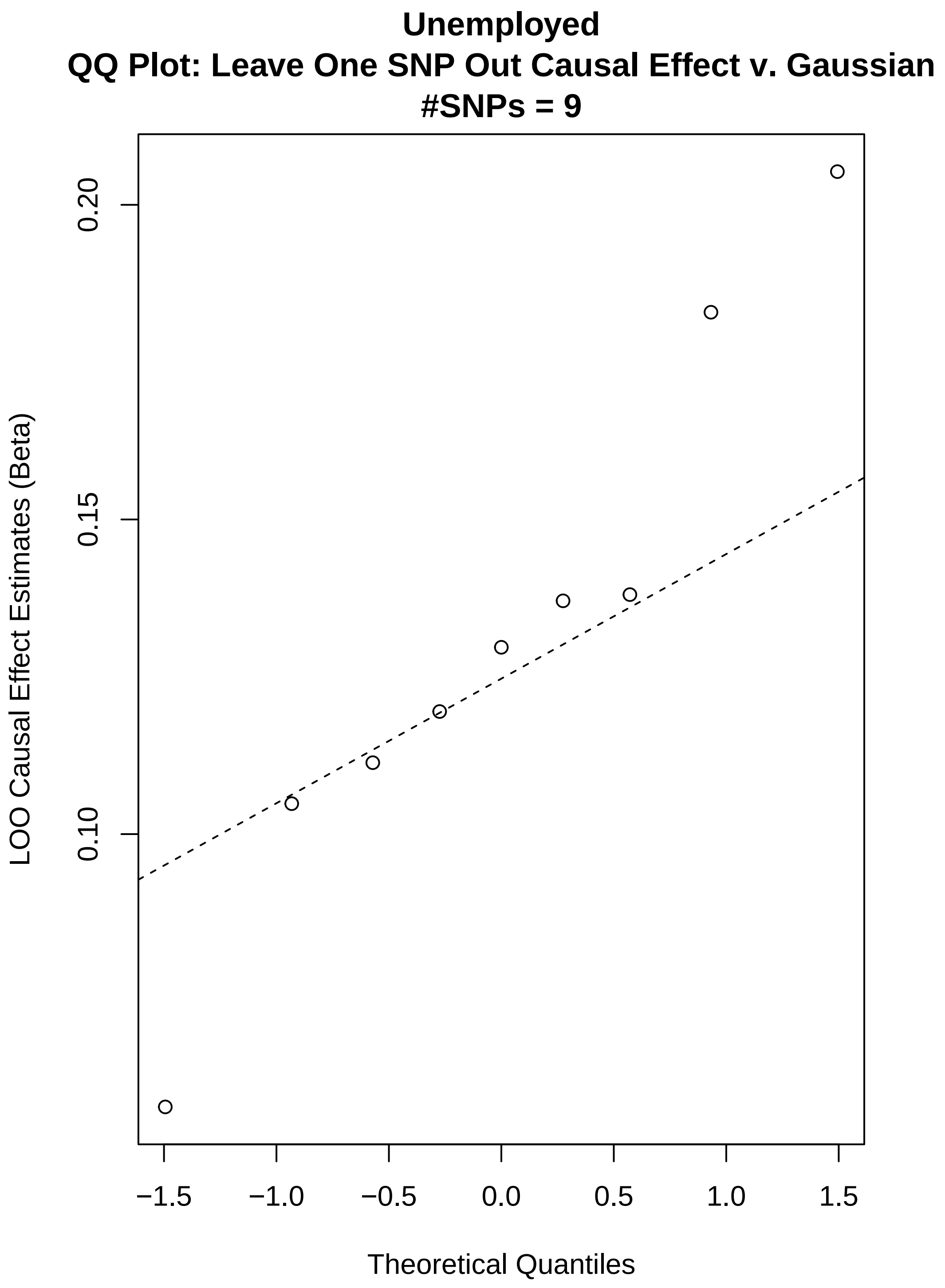

Unemployed
QQ Plot: Leave One SNP Out Causal Effect v. Gaussian
#SNPs = 9
LOO Causal Effect Estimates (Beta)
0.10
0.15
0.20
−1.5
−1.0
−0.5
0.0
0.5
1.0
1.5
Theoretical Quantiles

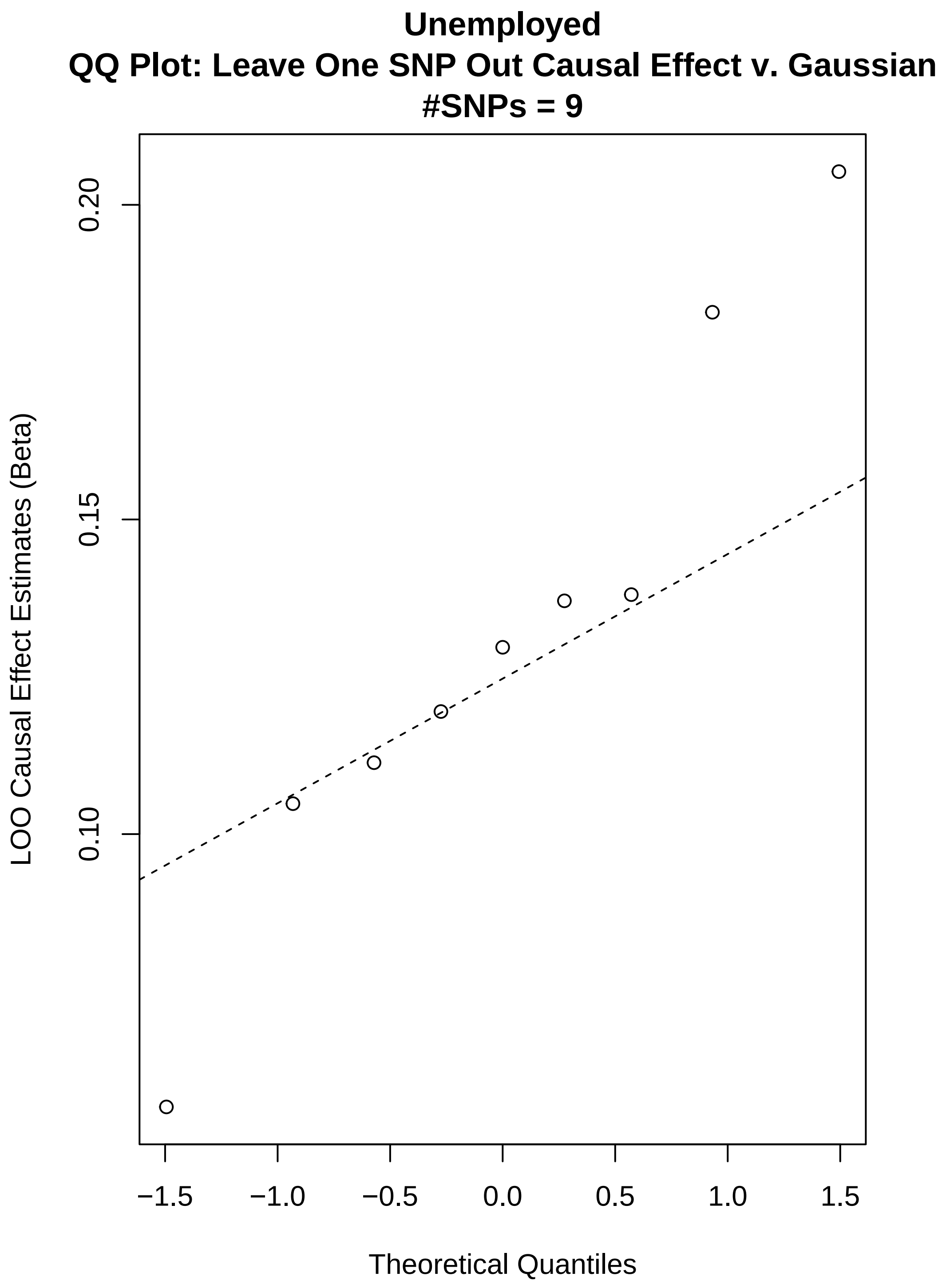

Unemployed
QQ Plot: Leave One SNP Out Causal Effect v. Gaussian
#SNPs = 9
LOO Causal Effect Estimates (Beta)
0.10
0.15
0.20
−1.5
−1.0
−0.5
0.0
0.5
1.0
1.5
Theoretical Quantiles

Unemployed
Rucker Model Selection Framework
Q = 5.4994, Q' = 5.4892, #SNPs = 9
Selected model = FE IVW

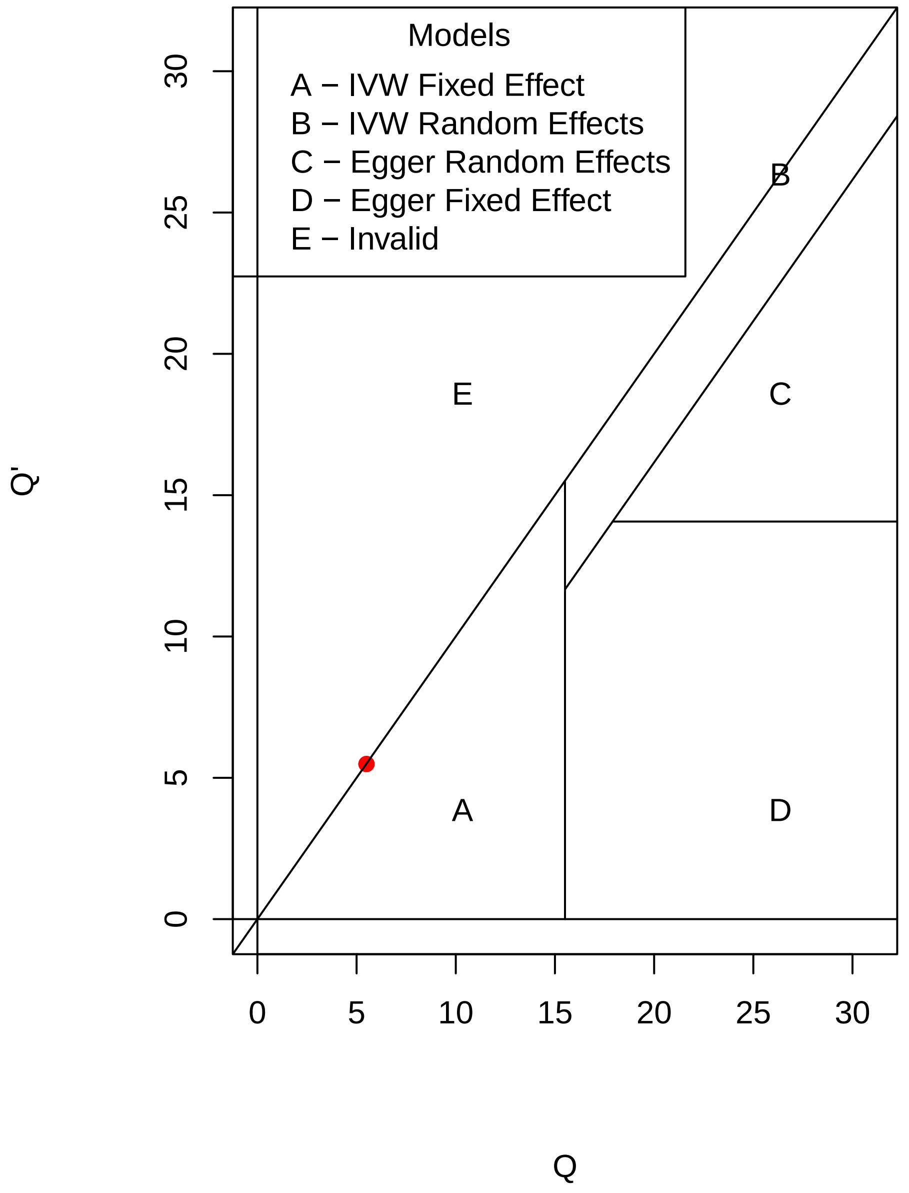

Unemployed
Rucker Model Selection Framework
Q = 5.4994, Q' = 5.4892, #SNPs = 9
Selected model = FE IVW

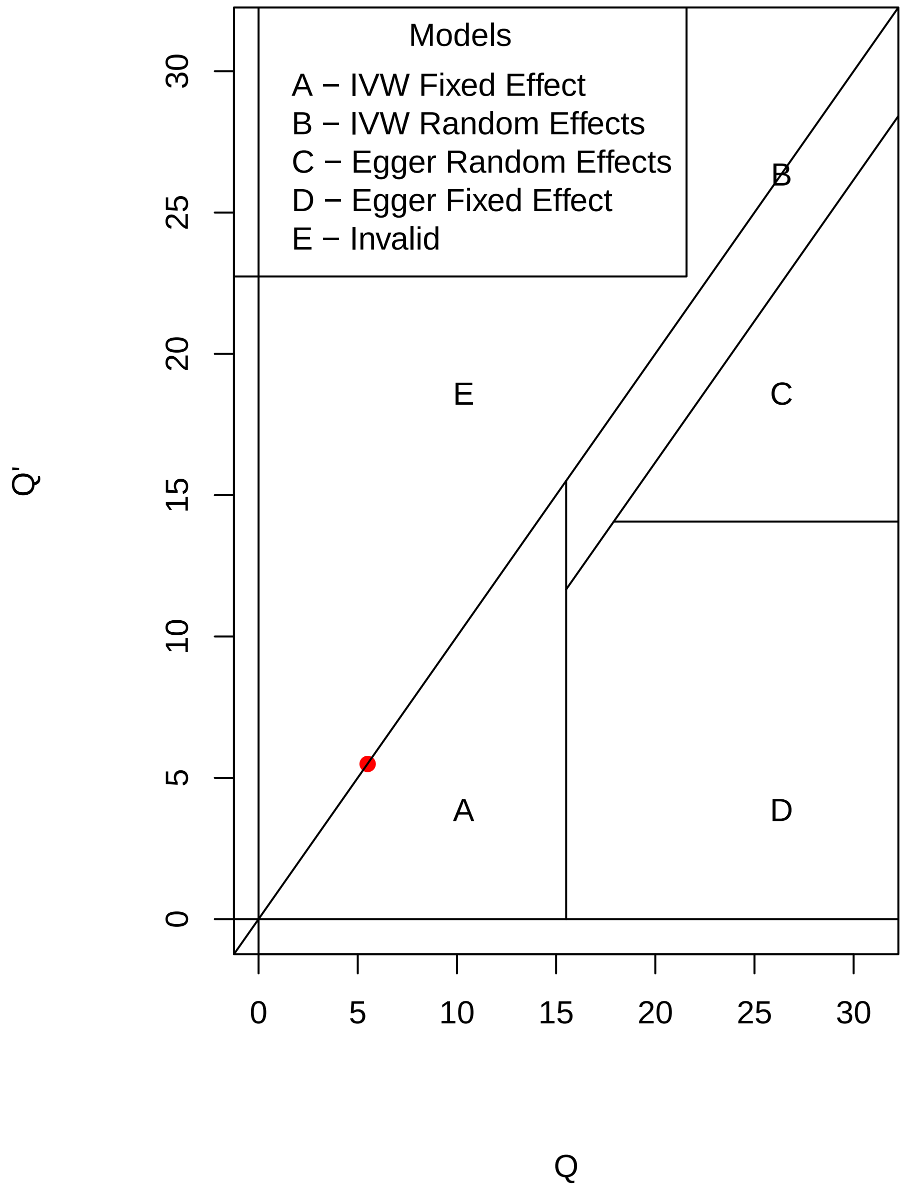

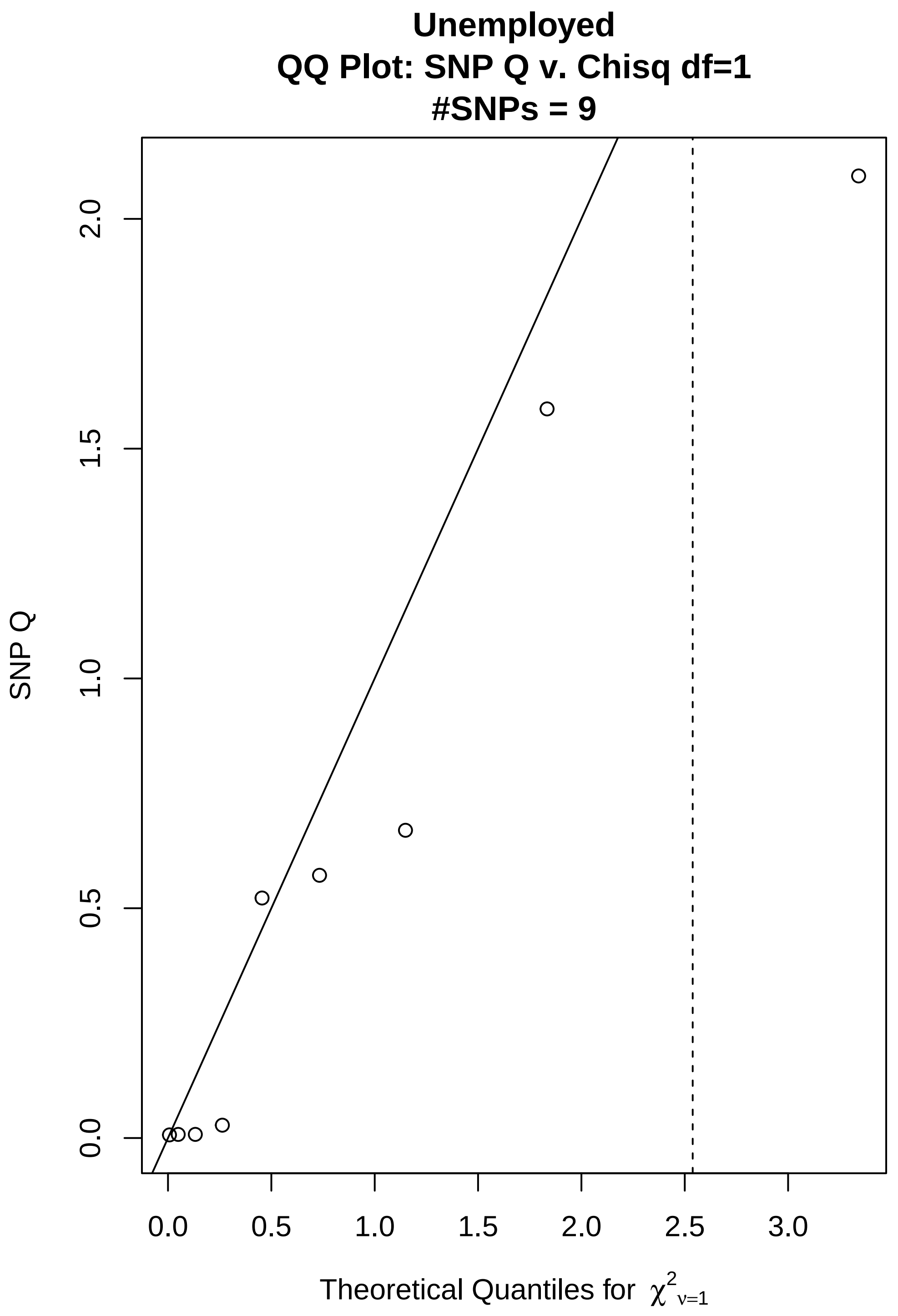
Unemployed
QQ Plot: SNP Q v. Chisq df=1
#SNPs = 9
SNP Q
0.0
0.5
1.0
1.5
2.0
0.0
0.5
1.0
1.5
2.0
2.5
3.0
Theoretical Quantiles for $\chi^2_{\nu=1}$

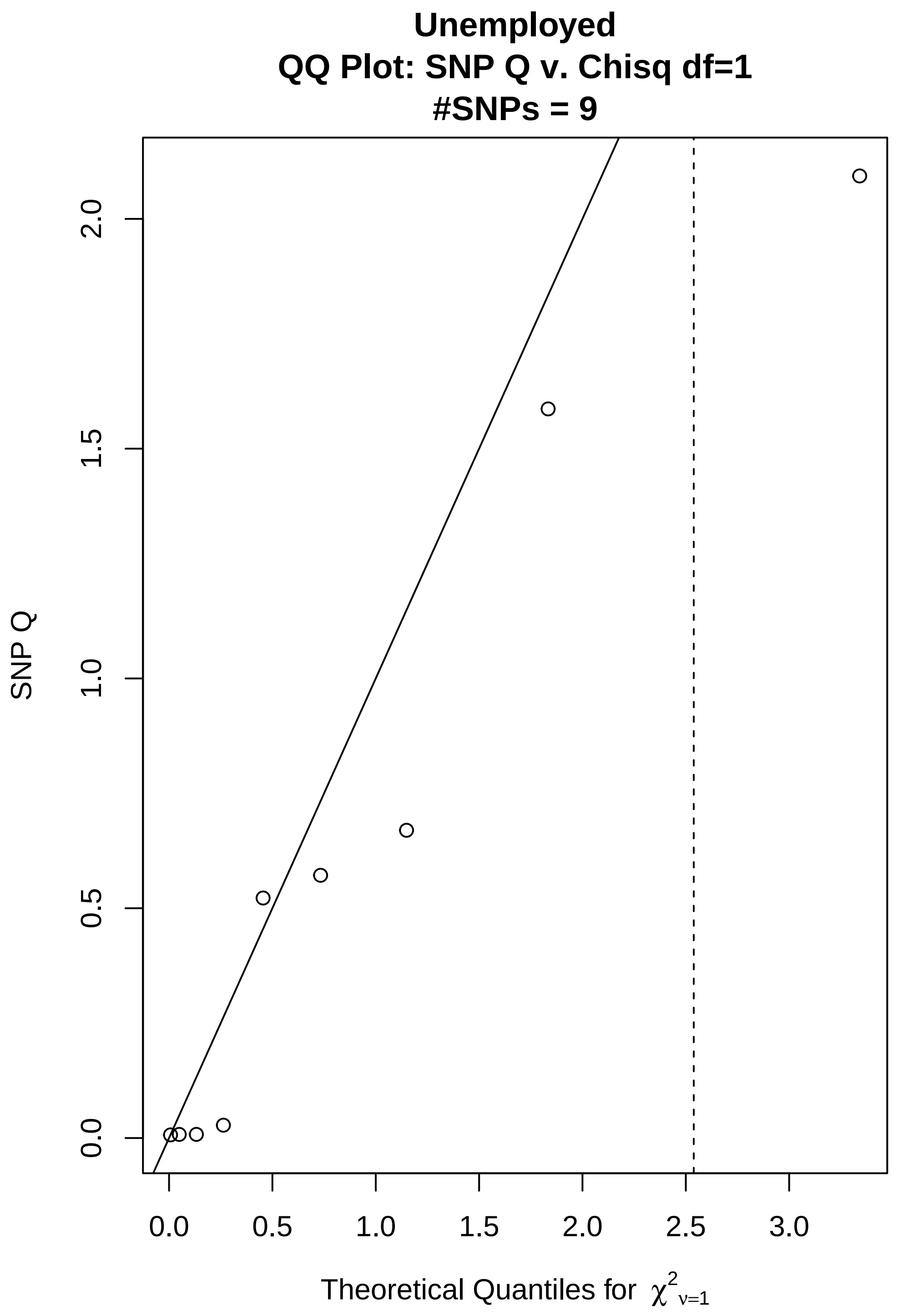
Unemployed
QQ Plot: SNP Q v. Chisq df=1
#SNPs = 9
SNP Q
0.0
0.5
1.0
1.5
2.0
0.0
0.5
1.0
1.5
2.0
2.5
3.0
Theoretical Quantiles for $\chi^2_{\nu=1}$
