# Supplementary material for: Effects of alcohol consumption on employment and social outcomes: a Mendelian randomisation study
Source: Alcohol Alcohol. 2025 Jul 18;60(5):agaf038. doi: 10.1093/alcalc/agaf038 (PMC12271571; doi:10.1093/alcalc/agaf038)
Supplement: Campbell_Green_Davies_et_al_2025_agaf038 [file campbell_green_davies_et_al_2025_agaf038.zip › Campbell_Green_Davies_et_al_2025/Female/auditc/do2SampleMrAnalyses_auditc_score_iOtherNotEmp_ageCentreGpc.pdf]

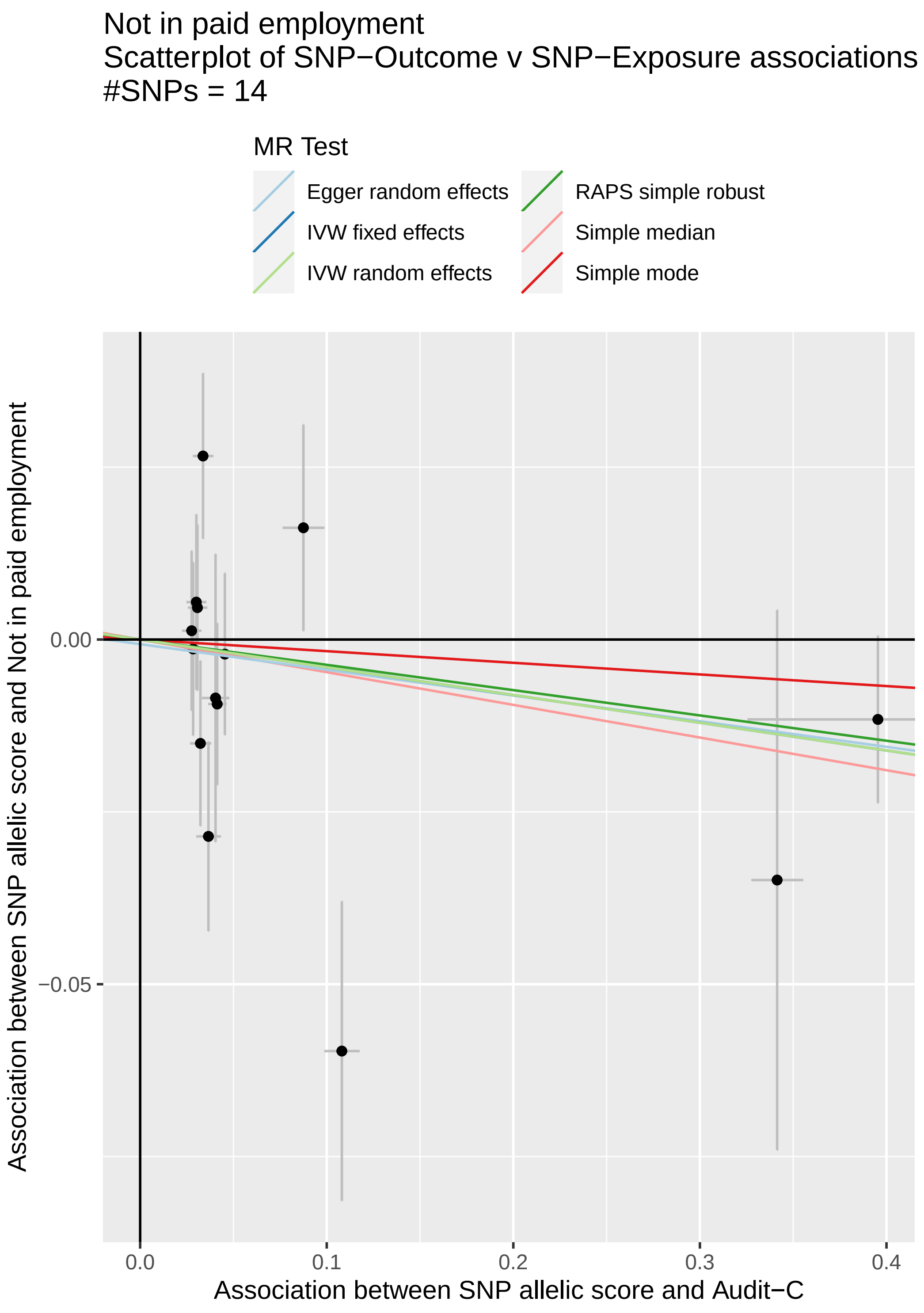

Not in paid employment
Scatterplot of SNP−Outcome v SNP−Exposure associations
#SNPs = 14
MR Test
Egger random effects
IVW fixed effects
IVW random effects
RAPS simple robust
Simple median
Simple mode
Association between SNP allelic score and Not in paid employment
0.00
−0.05
0.0
0.1
0.2
0.3
0.4
Association between SNP allelic score and Audit−C

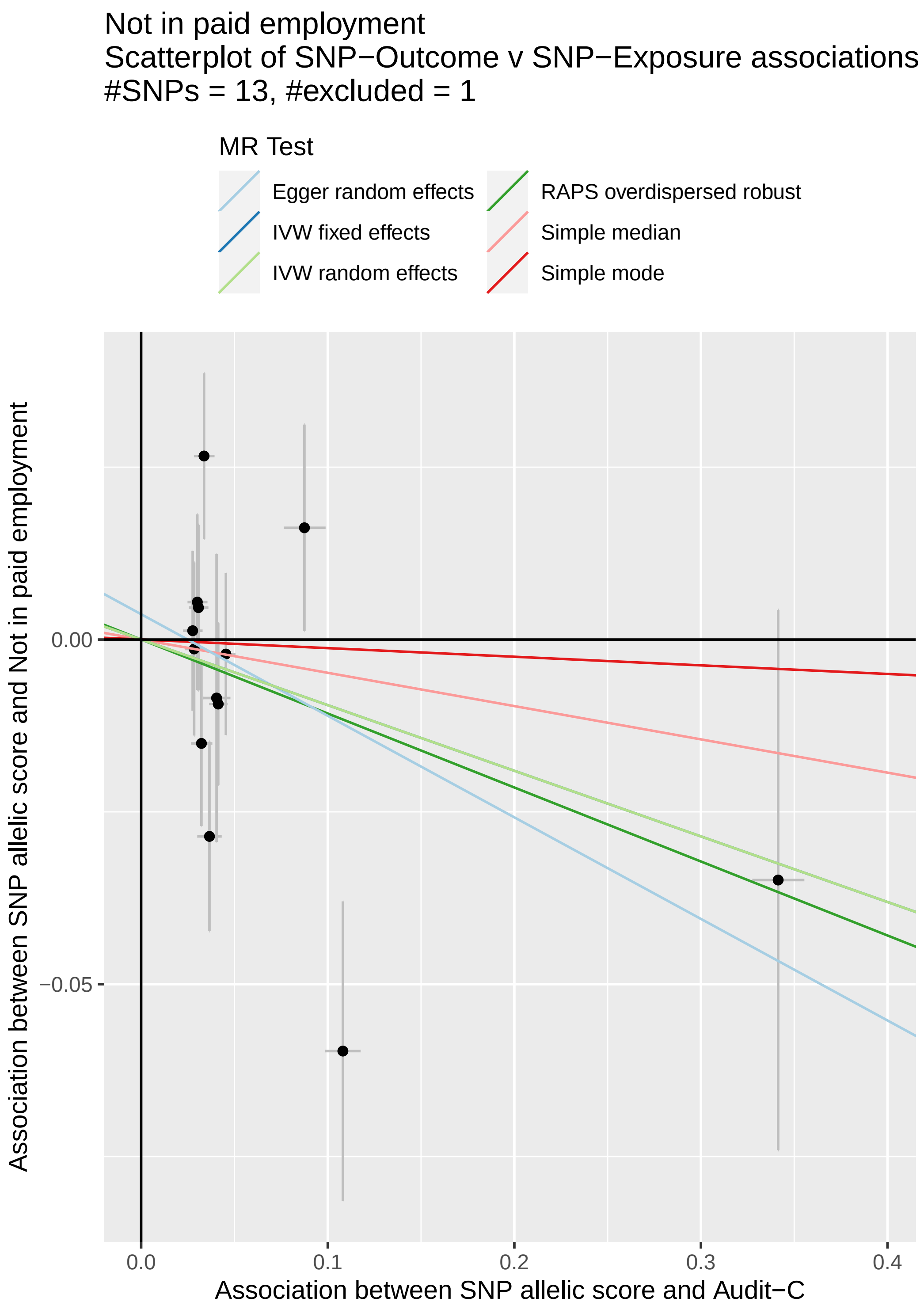

Not in paid employment
Scatterplot of SNP−Outcome v SNP−Exposure associations
#SNPs = 13, #excluded = 1
MR Test
Egger random effects
IVW fixed effects
IVW random effects
RAPS overdispersed robust
Simple median
Simple mode
Association between SNP allelic score and Not in paid employment
0.00
−0.05
0.0
0.1
0.2
0.3
0.4
Association between SNP allelic score and Audit−C

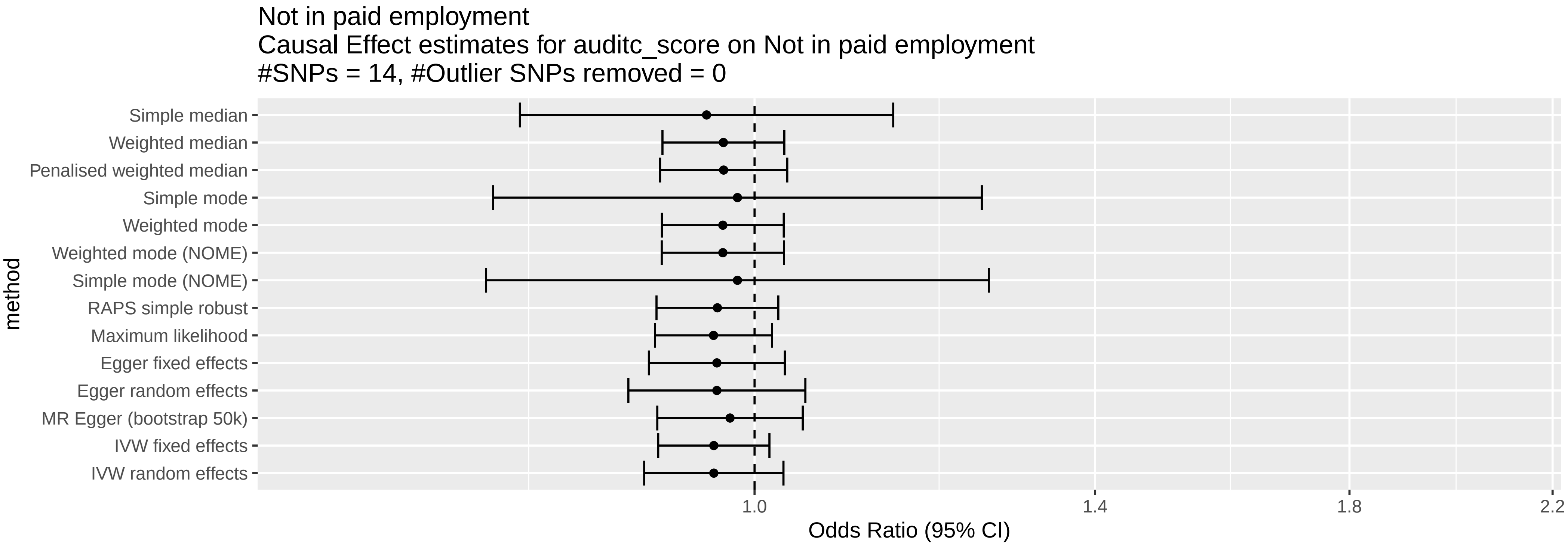
Not in paid employment
Causal Effect estimates for auditc_score on Not in paid employment
#SNPs = 14, #Outlier SNPs removed = 0
Simple median
Weighted median
Penalised weighted median
Simple mode
Weighted mode
Weighted mode (NOME)
Simple mode (NOME)
RAPS simple robust
Maximum likelihood
Egger fixed effects
Egger random effects
MR Egger (bootstrap 50k)
IVW fixed effects
IVW random effects
method
1.0
1.4
1.8
2.2
Odds Ratio (95% CI)

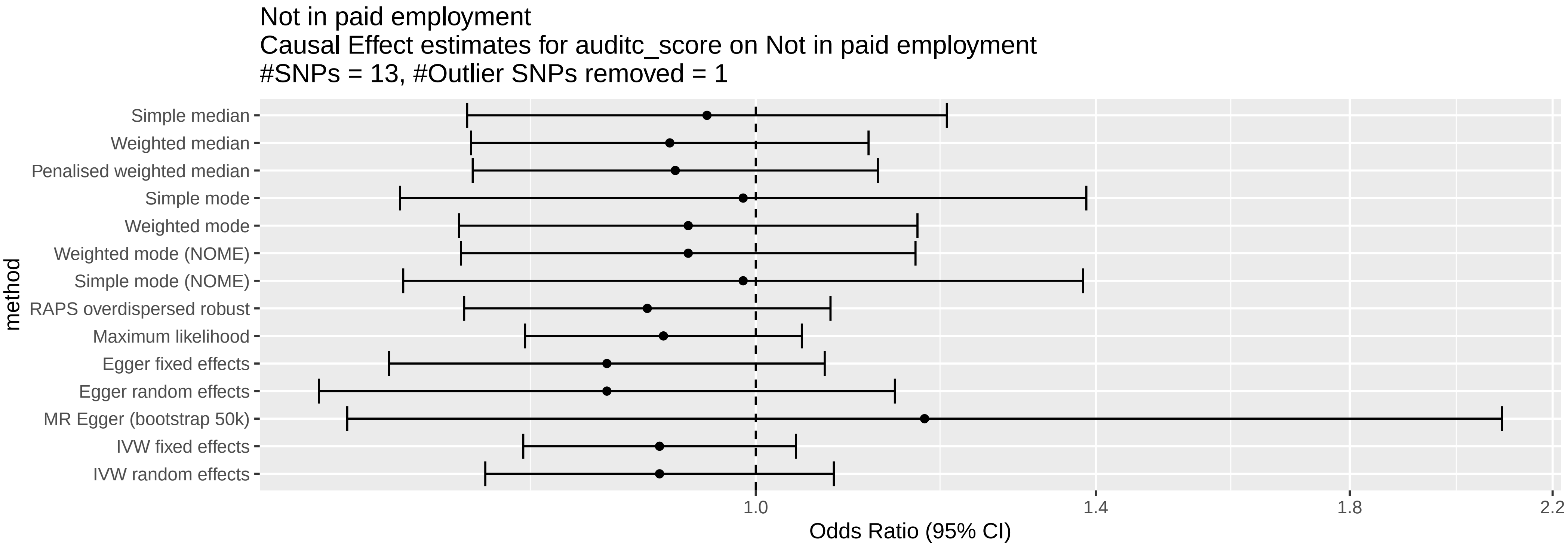
Not in paid employment
Causal Effect estimates for auditc_score on Not in paid employment
#SNPs = 13, #Outlier SNPs removed = 1
Simple median
Weighted median
Penalised weighted median
Simple mode
Weighted mode
Weighted mode (NOME)
Simple mode (NOME)
RAPS overdispersed robust
Maximum likelihood
Egger fixed effects
Egger random effects
MR Egger (bootstrap 50k)
IVW fixed effects
IVW random effects
method
1.0
1.4
1.8
2.2
Odds Ratio (95% CI)

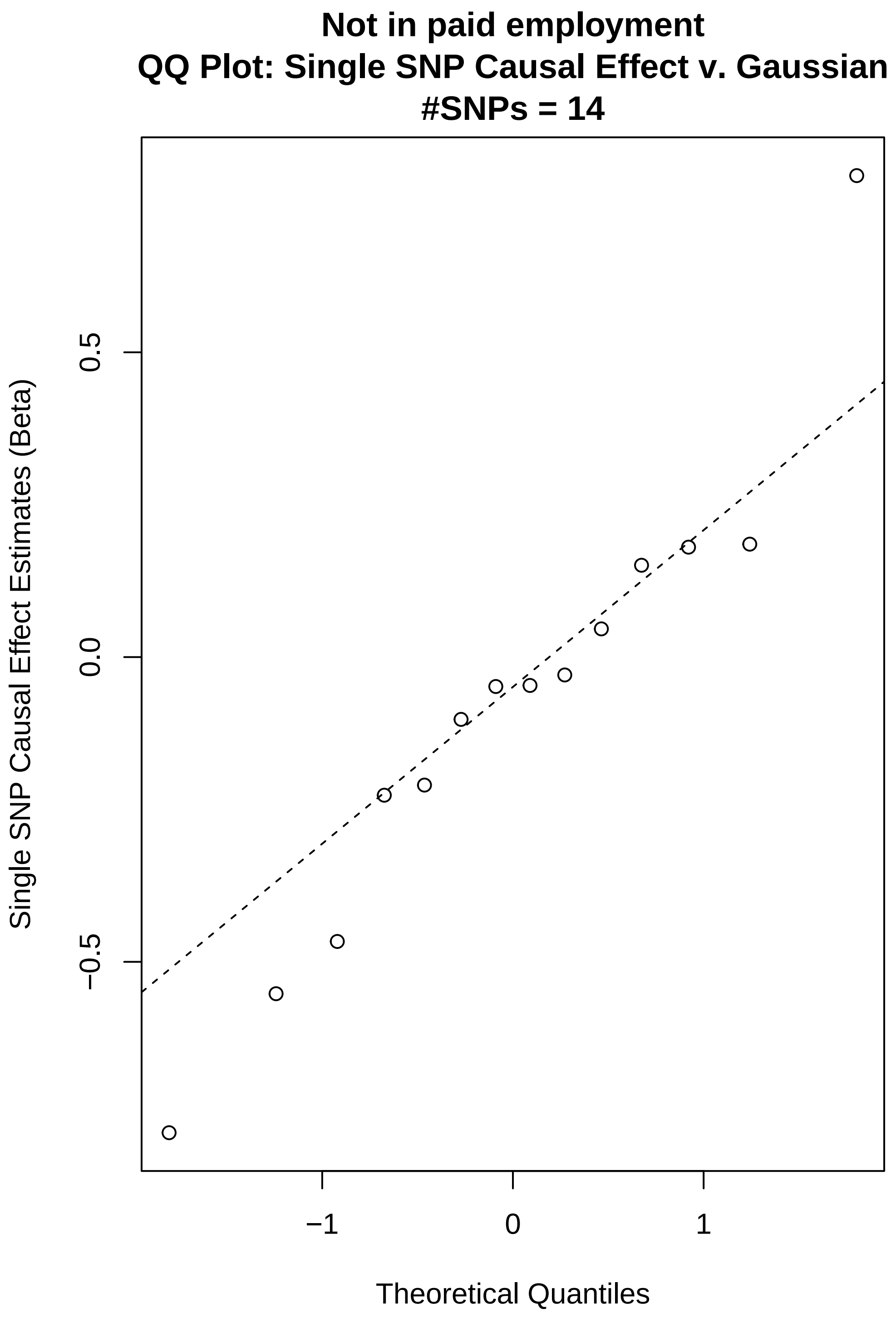
Not in paid employment
QQ Plot: Single SNP Causal Effect v. Gaussian
#SNPs = 14
Single SNP Causal Effect Estimates (Beta)
0.5
0.0
−0.5
−1
0
1
Theoretical Quantiles

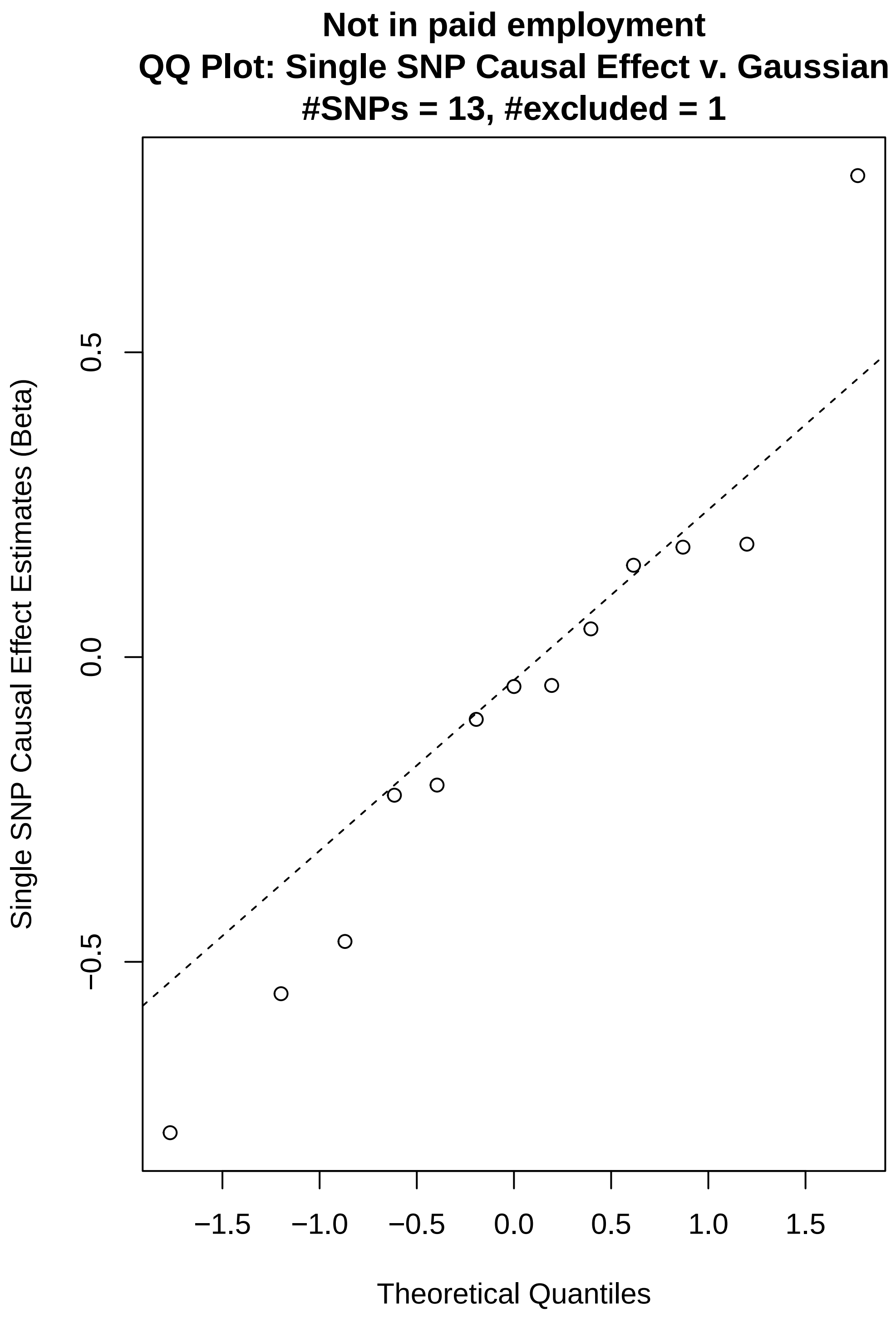
Not in paid employment
QQ Plot: Single SNP Causal Effect v. Gaussian
#SNPs = 13, #excluded = 1
Single SNP Causal Effect Estimates (Beta)
0.5
0.0
−0.5
−1.5
−1.0
−0.5
0.0
0.5
1.0
1.5
Theoretical Quantiles

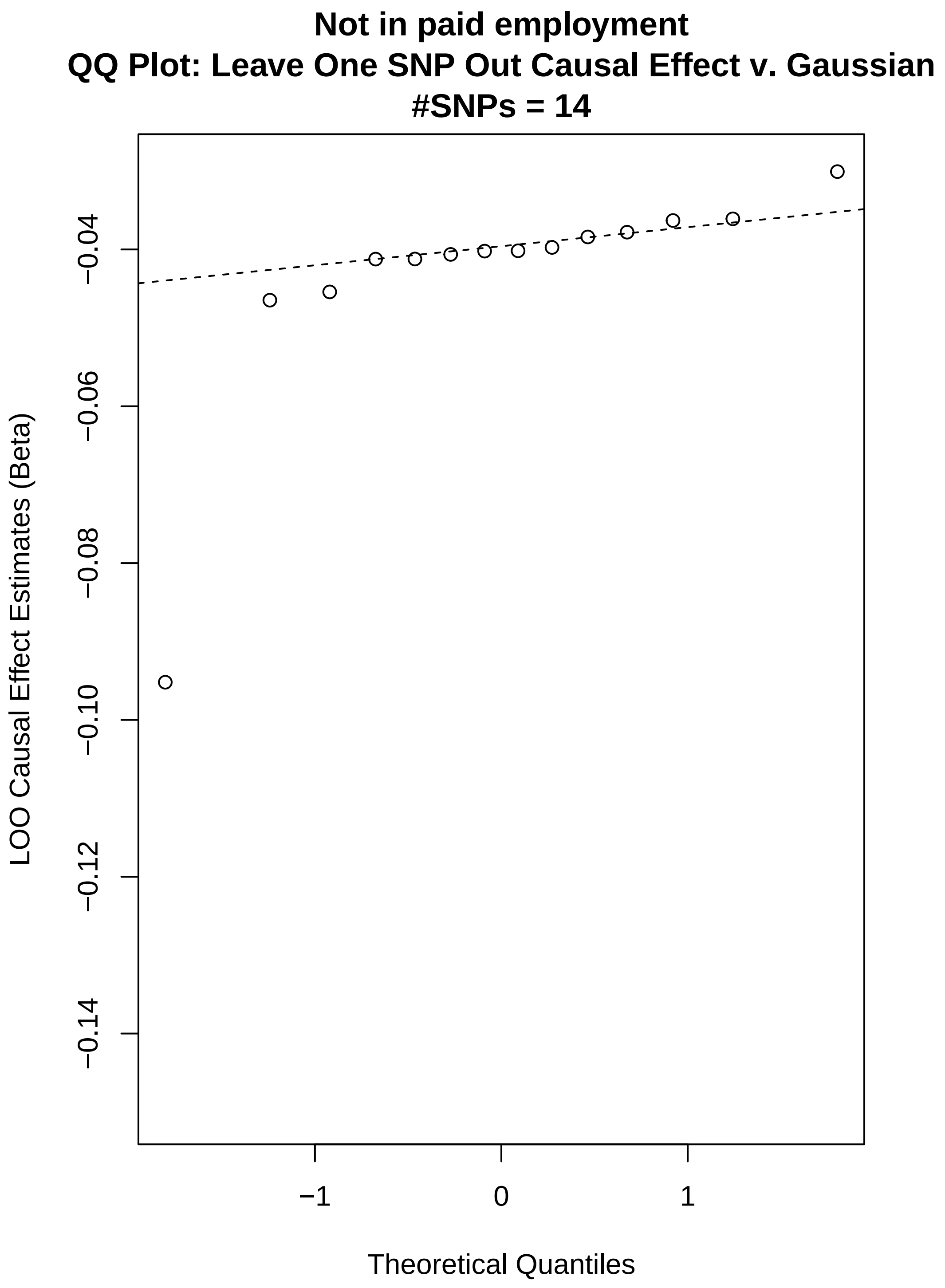
Not in paid employment
QQ Plot: Leave One SNP Out Causal Effect v. Gaussian
#SNPs = 14
LOO Causal Effect Estimates (Beta)
−0.04
−0.06
−0.08
−0.10
−0.12
−0.14
−1
0
1
Theoretical Quantiles

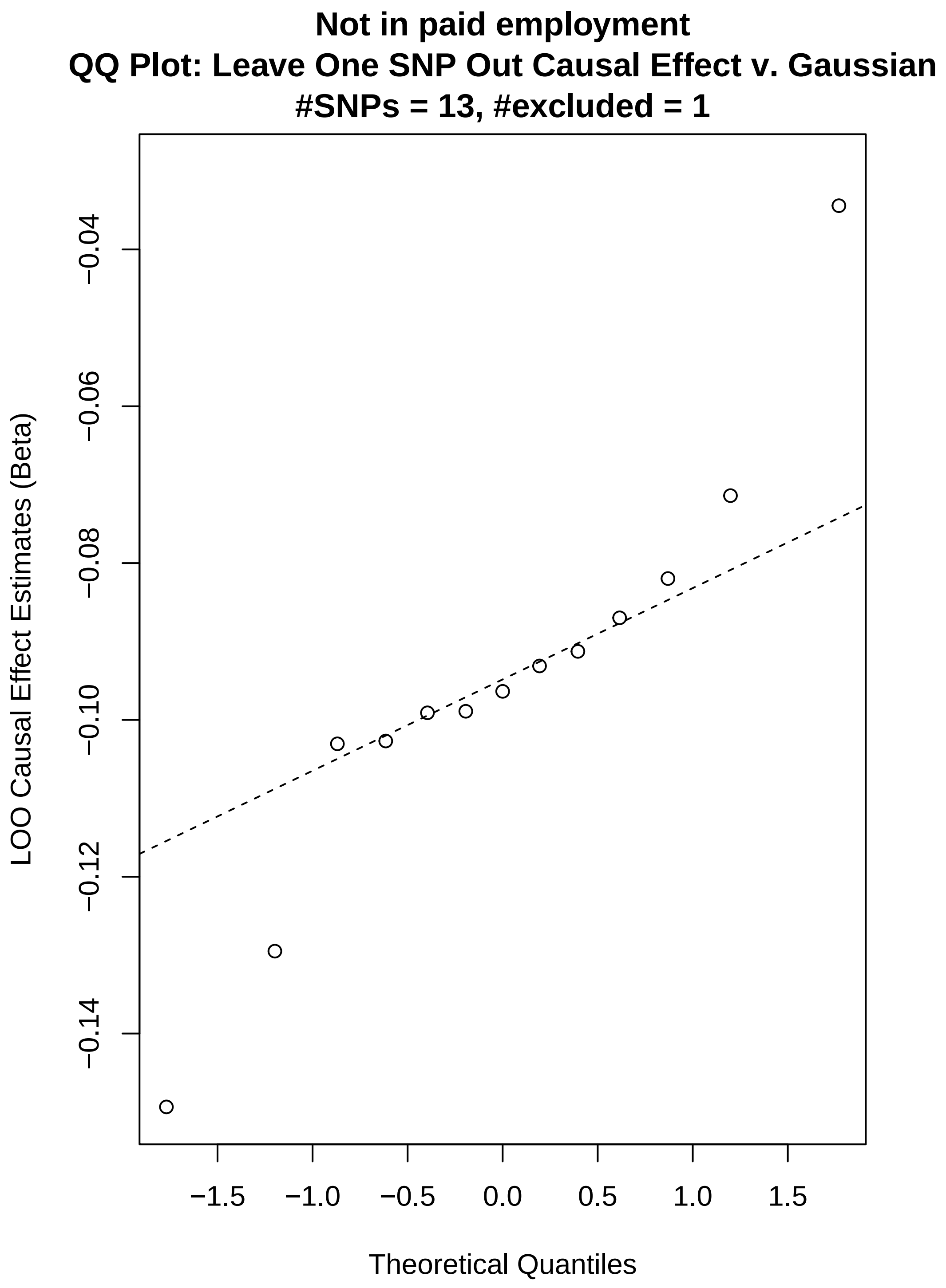
Not in paid employment
QQ Plot: Leave One SNP Out Causal Effect v. Gaussian
#SNPs = 13, #excluded = 1
LOO Causal Effect Estimates (Beta)
−0.04
−0.06
−0.08
−0.10
−0.12
−0.14
−1.5
−1.0
−0.5
0.0
0.5
1.0
1.5
Theoretical Quantiles

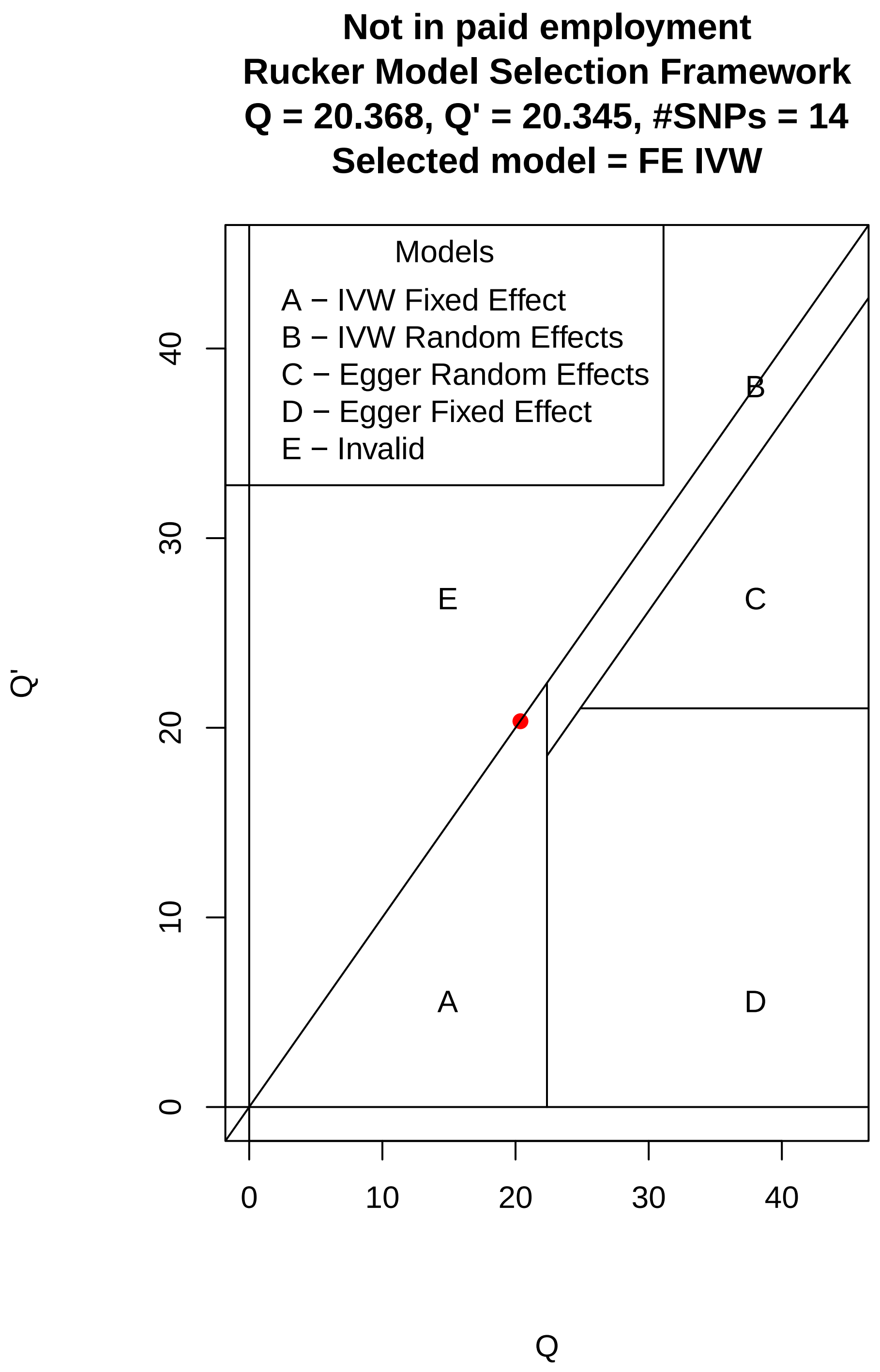

Not in paid employment
Rucker Model Selection Framework
Q = 20.368, Q' = 20.345, #SNPs = 14
Selected model = FE IVW
Models
A − IVW Fixed Effect
B − IVW Random Effects
C − Egger Random Effects
D − Egger Fixed Effect
E − Invalid
A
B
C
D
E
Q
Q'
0
10
20
30
40

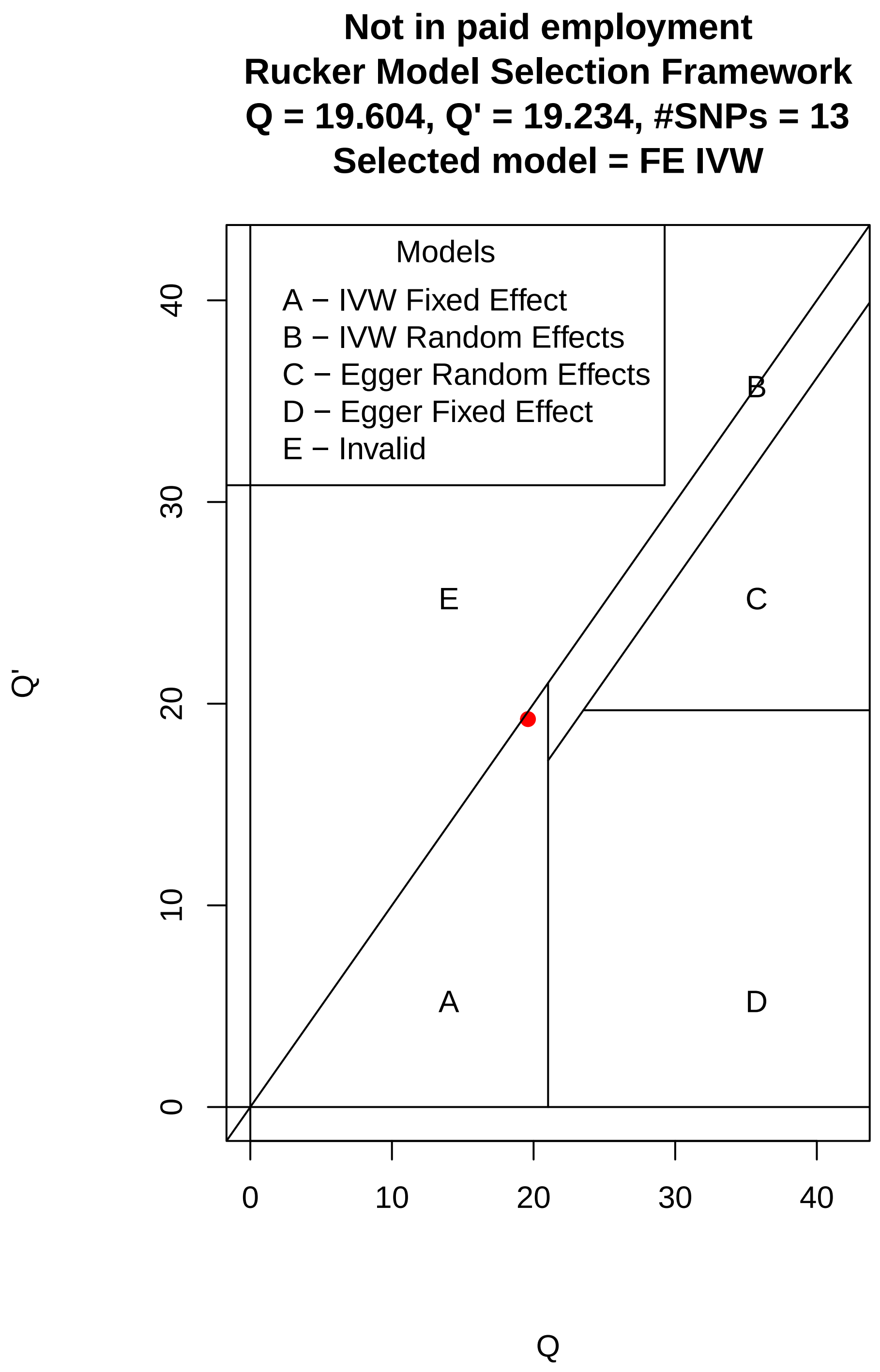

Not in paid employment
Rucker Model Selection Framework
Q = 19.604, Q' = 19.234, #SNPs = 13
Selected model = FE IVW
Models
A − IVW Fixed Effect
B − IVW Random Effects
C − Egger Random Effects
D − Egger Fixed Effect
E − Invalid
A
B
C
D
E
Q
Q'
0
10
20
30
40

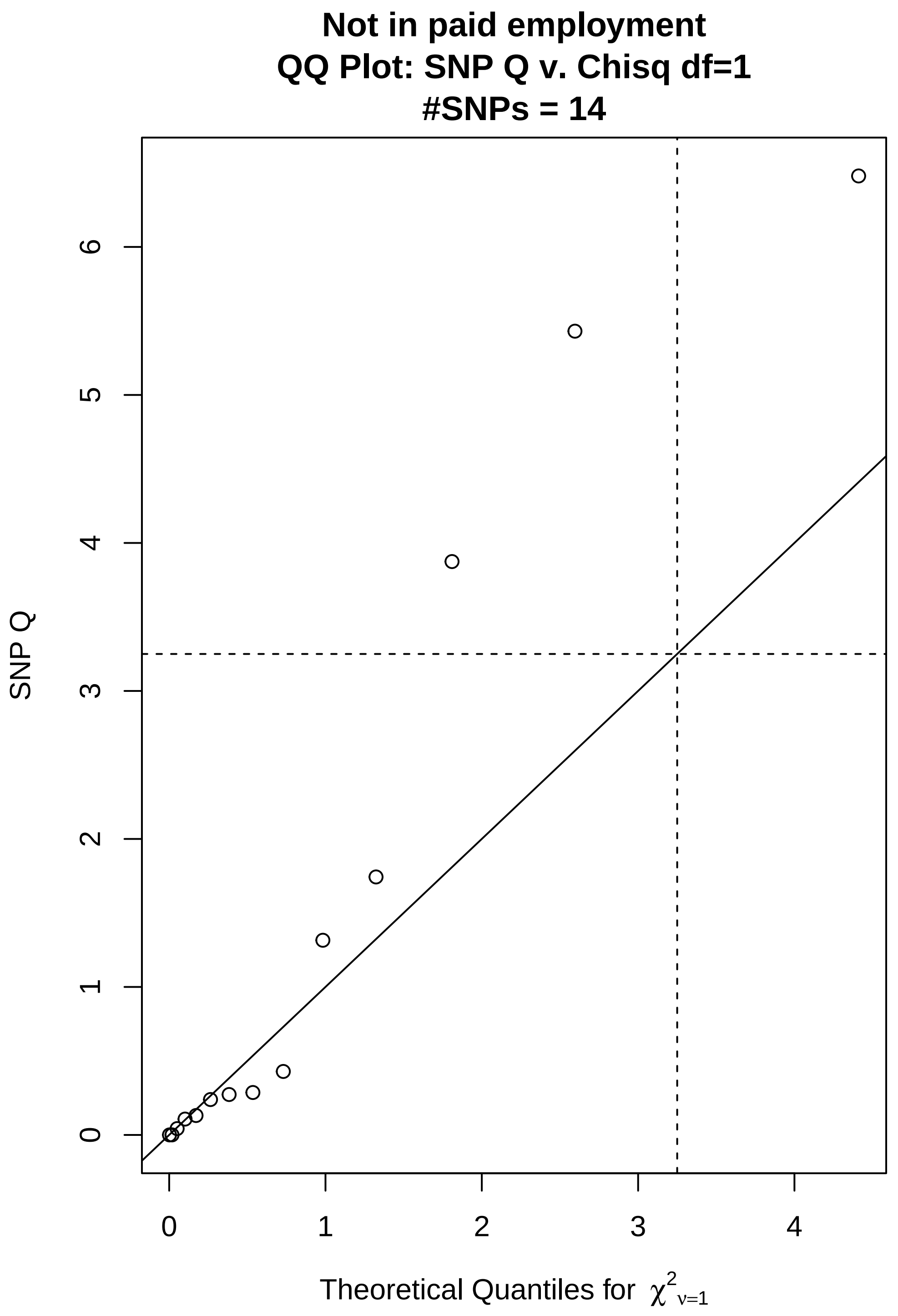

Not in paid employment
QQ Plot: SNP Q v. Chisq df=1
#SNPs = 14
SNP Q
0
1
2
3
4
5
6
0
1
2
3
4
Theoretical Quantiles for $\chi^2_{\nu=1}$

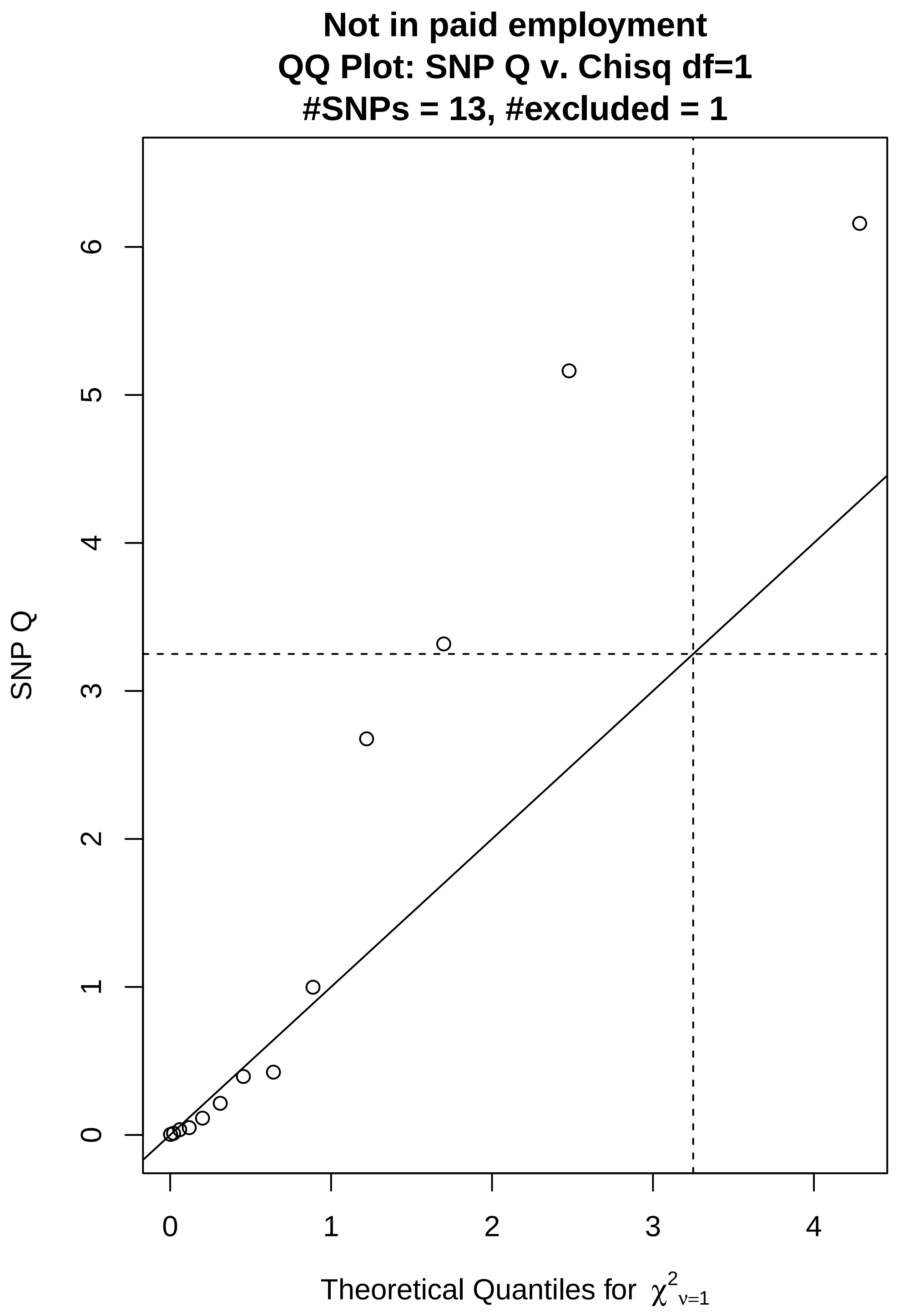

Not in paid employment
QQ Plot: SNP Q v. Chisq df=1
#SNPs = 13, #excluded = 1
SNP Q
0
1
2
3
4
5
6
0
1
2
3
4
Theoretical Quantiles for $\chi^2_{\nu=1}$
