# Supplementary material for: Effects of alcohol consumption on employment and social outcomes: a Mendelian randomisation study
Source: Alcohol Alcohol. 2025 Jul 18;60(5):agaf038. doi: 10.1093/alcalc/agaf038 (PMC12271571; doi:10.1093/alcalc/agaf038)
Supplement: Campbell_Green_Davies_et_al_2025_agaf038 [file campbell_green_davies_et_al_2025_agaf038.zip › Campbell_Green_Davies_et_al_2025/Female/auditc/do2SampleMrAnalyses_auditc_score_iRetiredNotEmp_ageCentreGpc.pdf]

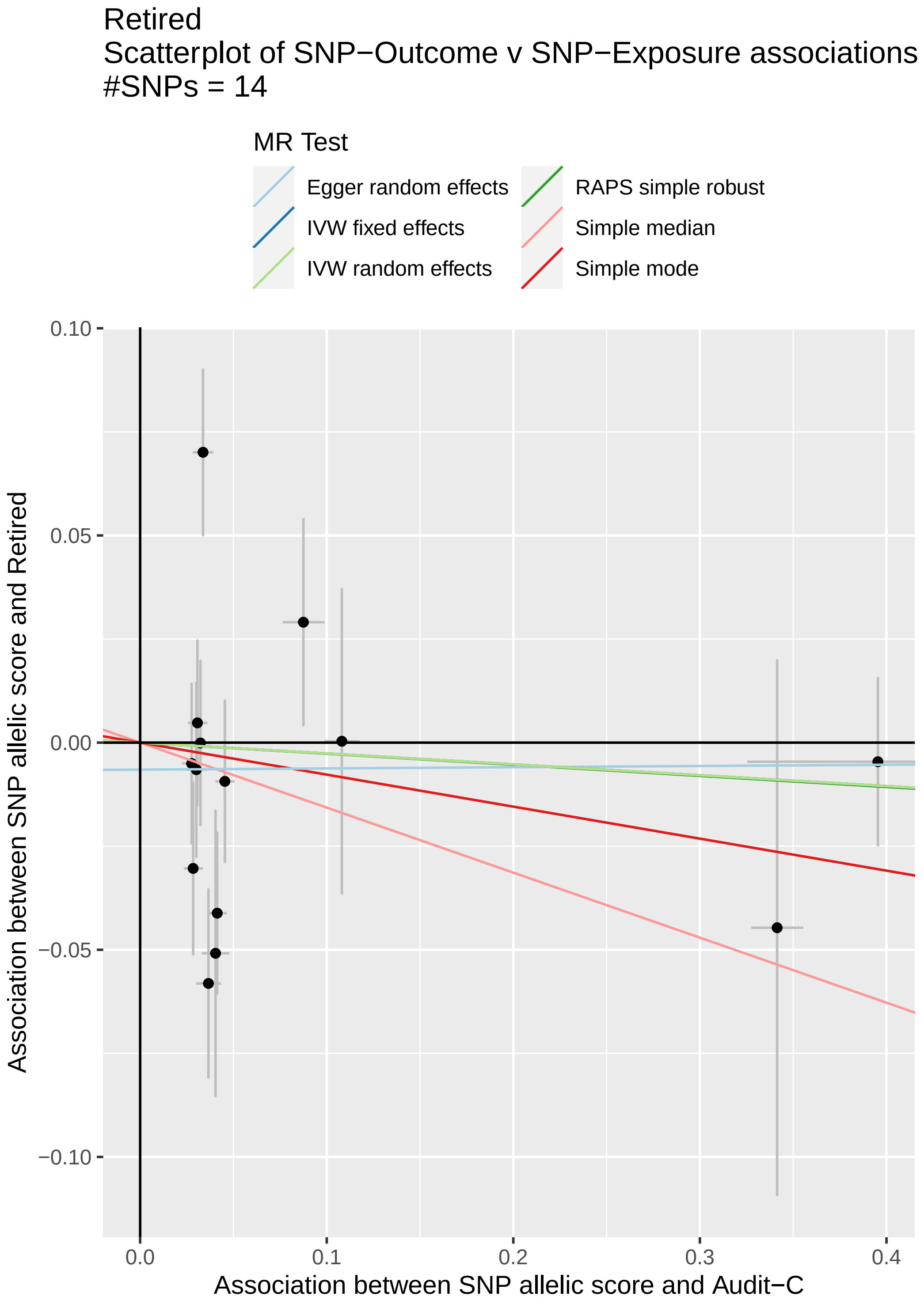
Retired
Scatterplot of SNP−Outcome v SNP−Exposure associations
#SNPs = 14
MR Test
Egger random effects
IVW fixed effects
IVW random effects
RAPS simple robust
Simple median
Simple mode
0.10
0.05
0.00
−0.05
−0.10
0.0
0.1
0.2
0.3
0.4
Association between SNP allelic score and Retired
Association between SNP allelic score and Audit−C

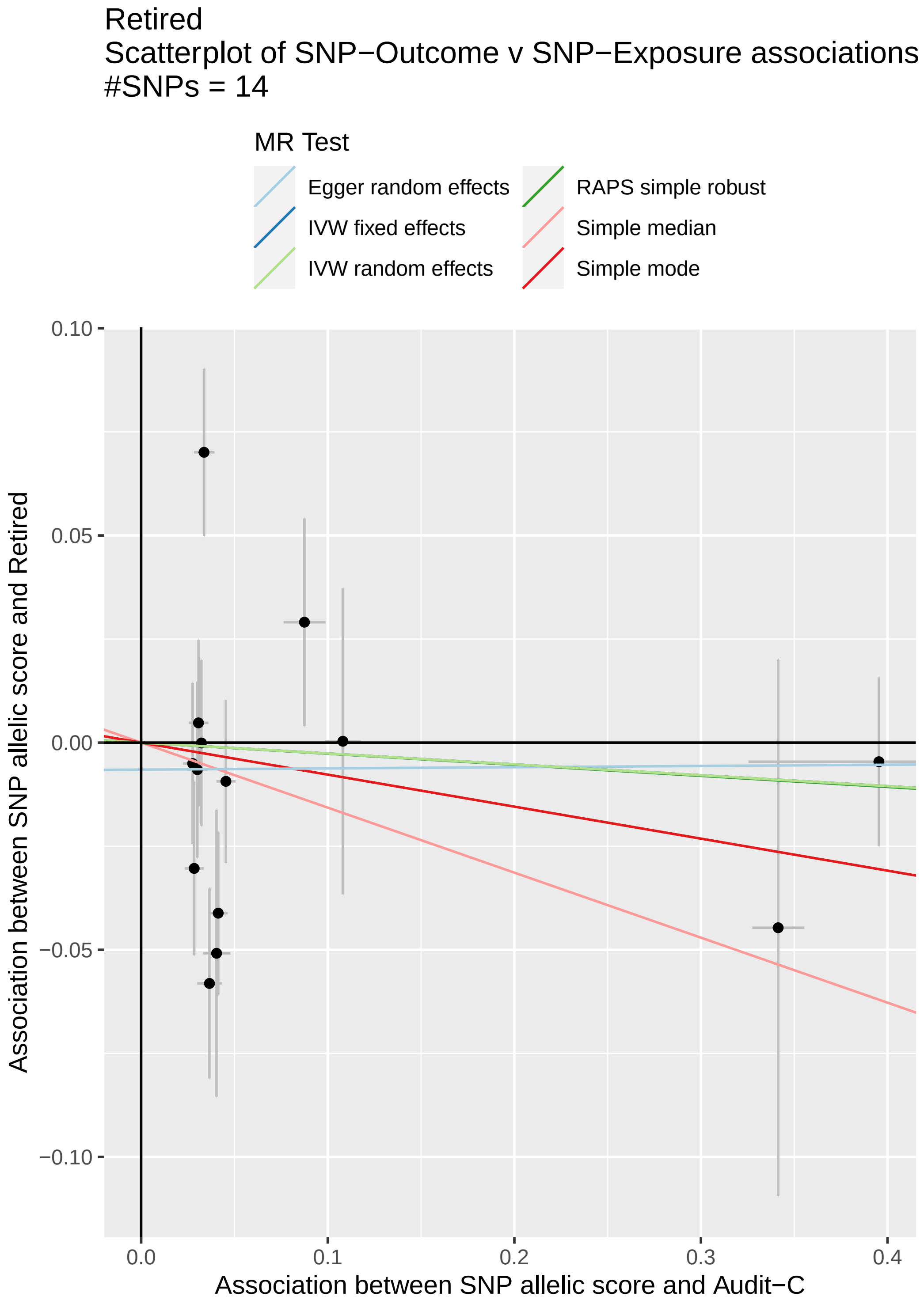
Retired
Scatterplot of SNP−Outcome v SNP−Exposure associations
#SNPs = 14
MR Test
Egger random effects
IVW fixed effects
IVW random effects
RAPS simple robust
Simple median
Simple mode
0.10
0.05
0.00
−0.05
−0.10
0.0
0.1
0.2
0.3
0.4
Association between SNP allelic score and Retired
Association between SNP allelic score and Audit−C

Retired
Causal Effect estimates for auditc_score on Retired
#SNPs = 14, #Outlier SNPs removed = 0

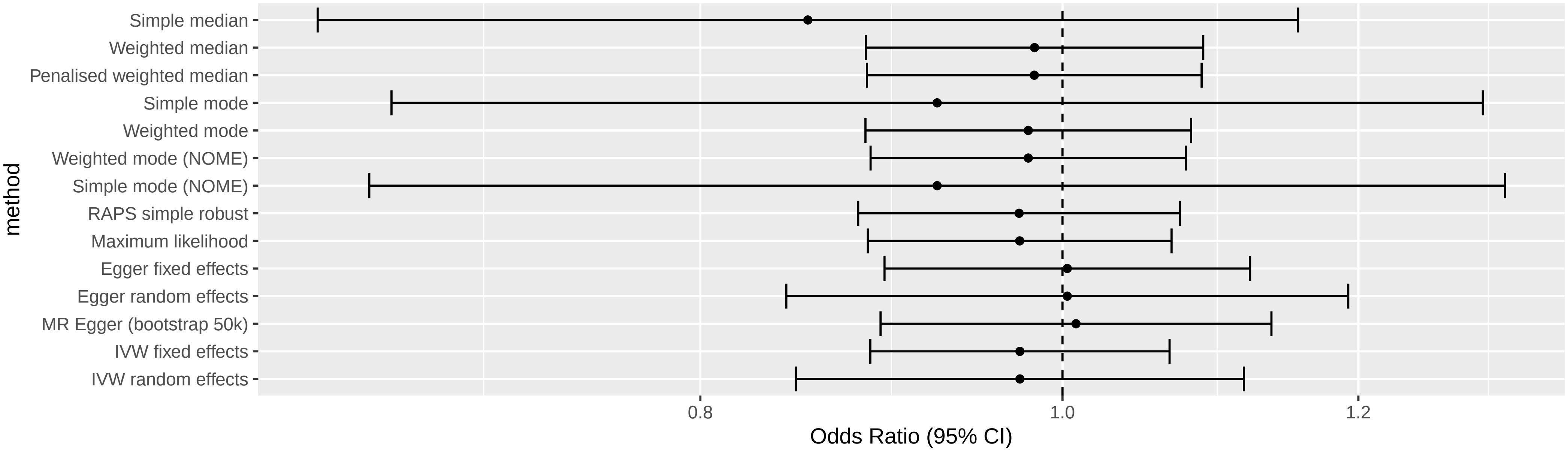

Retired
Causal Effect estimates for auditc_score on Retired
#SNPs = 14, #Outlier SNPs removed = 0

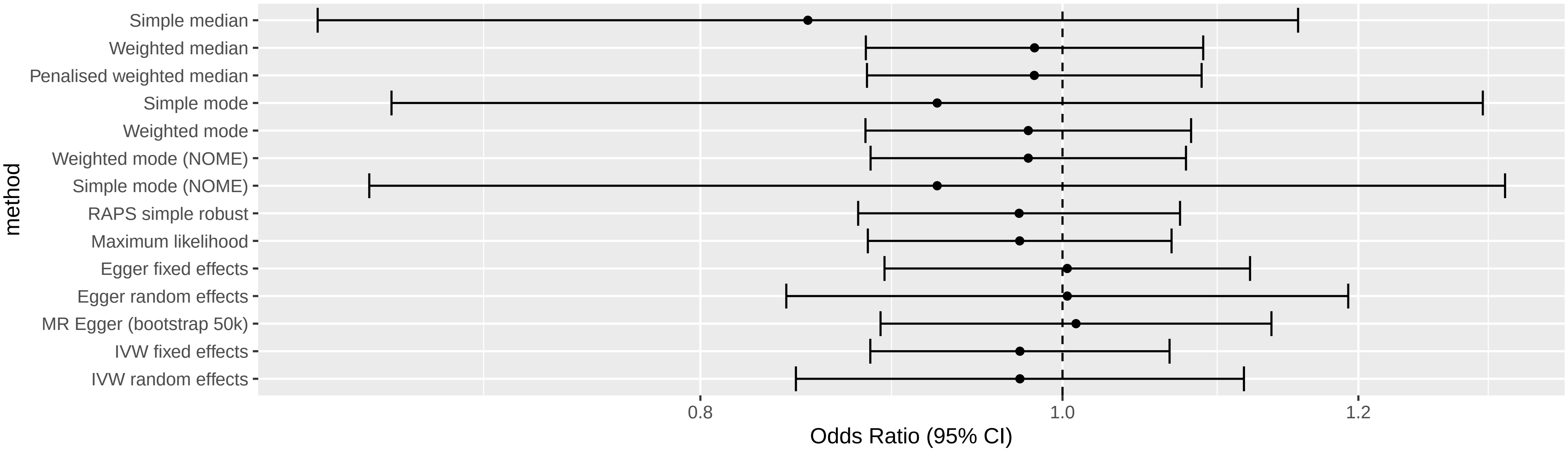

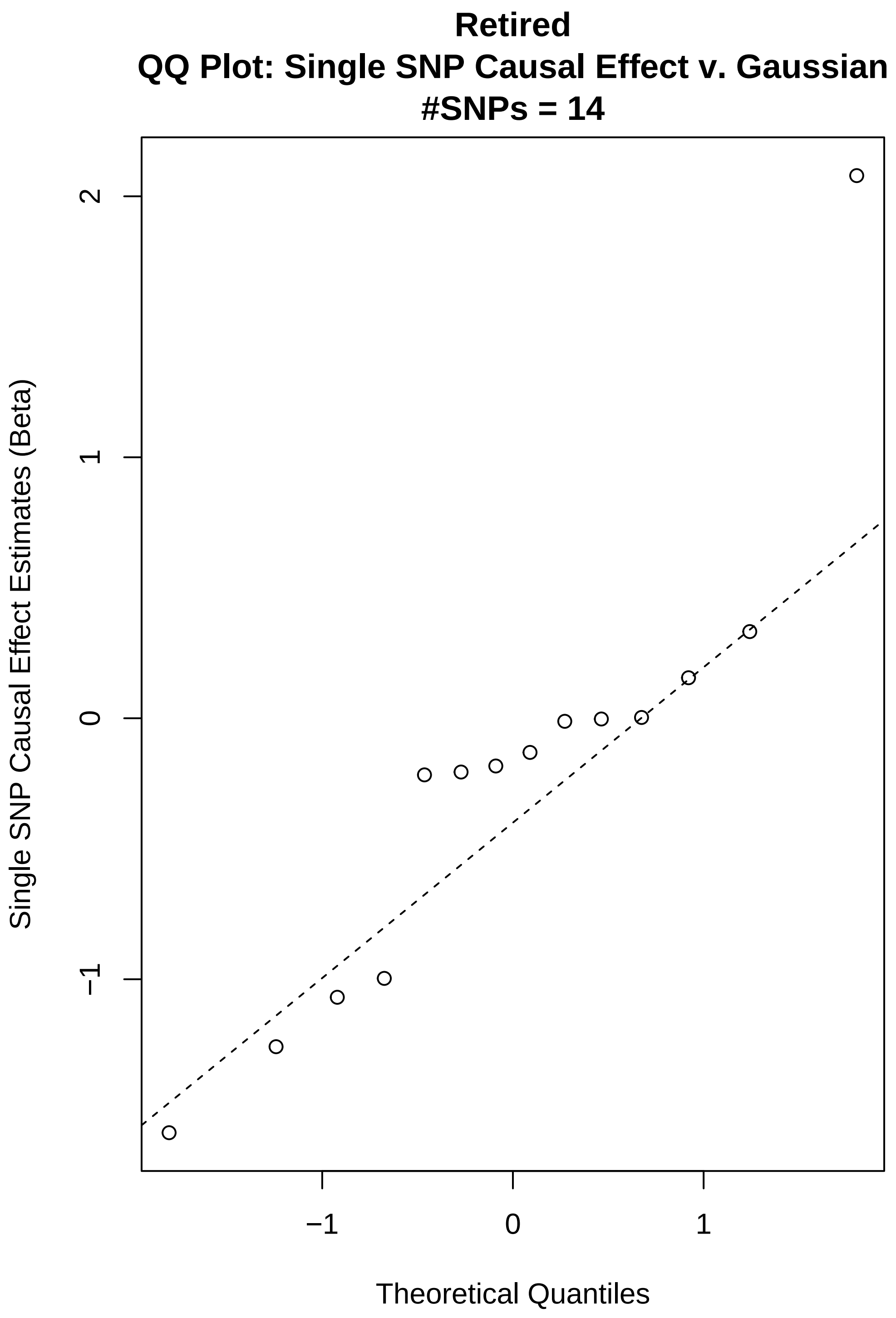
Retired
QQ Plot: Single SNP Causal Effect v. Gaussian
#SNPs = 14
Single SNP Causal Effect Estimates (Beta)
Theoretical Quantiles
−1
0
1
2

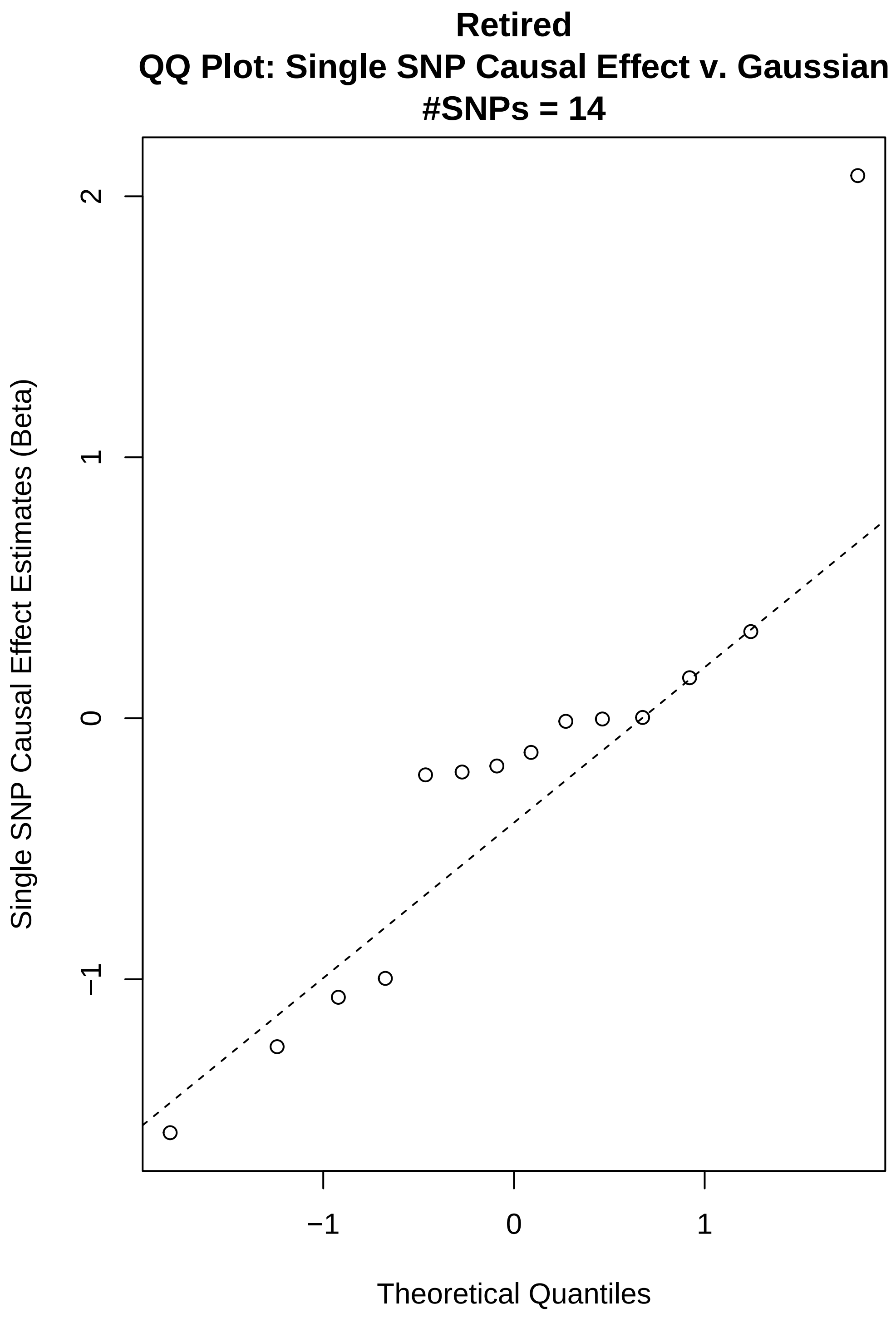
Retired
QQ Plot: Single SNP Causal Effect v. Gaussian
#SNPs = 14
Single SNP Causal Effect Estimates (Beta)
Theoretical Quantiles
−1
0
1
2

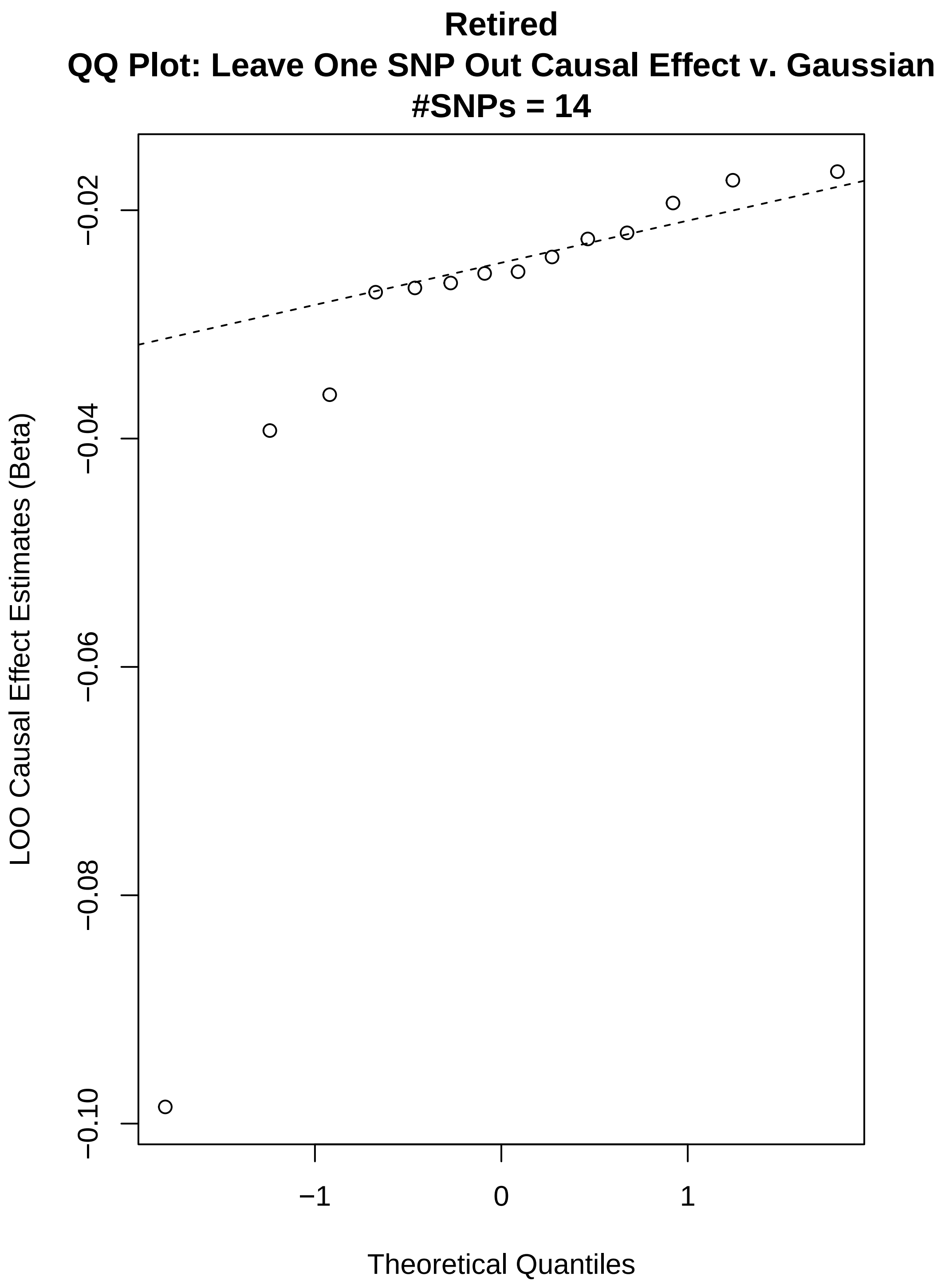
Retired
QQ Plot: Leave One SNP Out Causal Effect v. Gaussian
#SNPs = 14
LOO Causal Effect Estimates (Beta)
−0.02
−0.04
−0.06
−0.08
−0.10
−1
0
1
Theoretical Quantiles

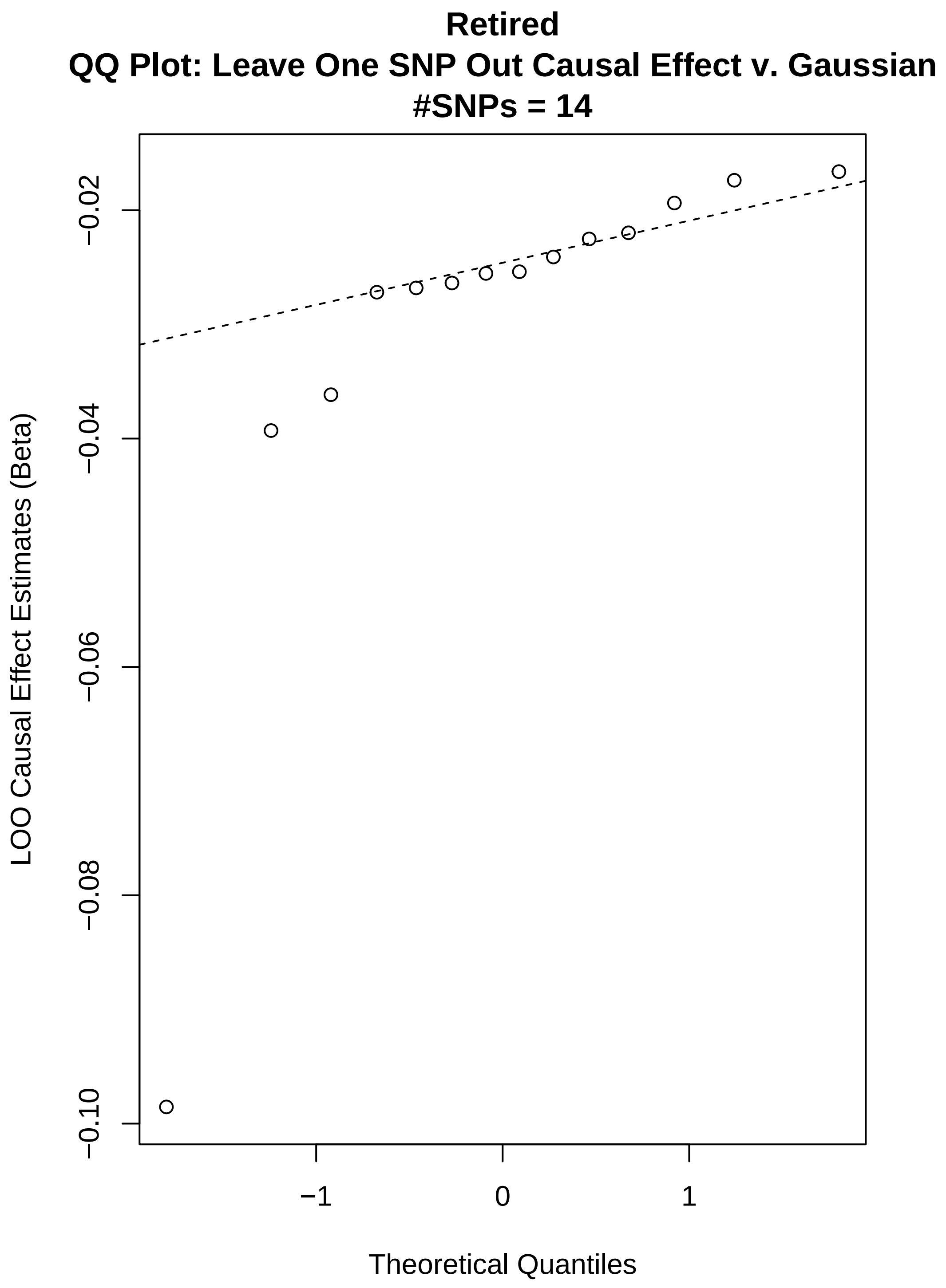
Retired
QQ Plot: Leave One SNP Out Causal Effect v. Gaussian
#SNPs = 14
LOO Causal Effect Estimates (Beta)
−0.02
−0.04
−0.06
−0.08
−0.10
−1
0
1
Theoretical Quantiles

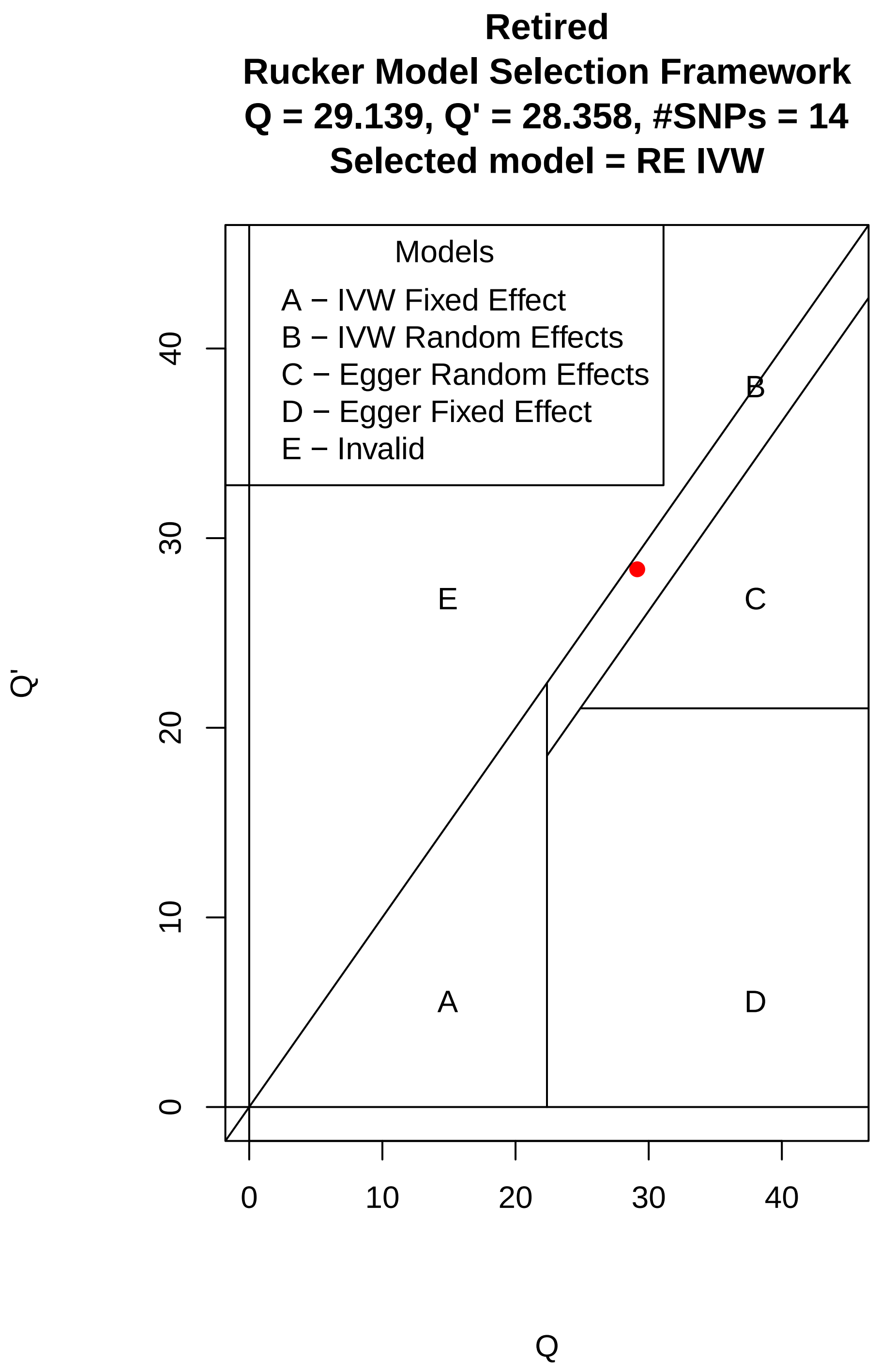
Retired
Rucker Model Selection Framework
Q = 29.139, Q' = 28.358, #SNPs = 14
Selected model = RE IVW
Models
A − IVW Fixed Effect
B − IVW Random Effects
C − Egger Random Effects
D − Egger Fixed Effect
E − Invalid
A
B
C
D
E
Q'
Q
0
10
20
30
40

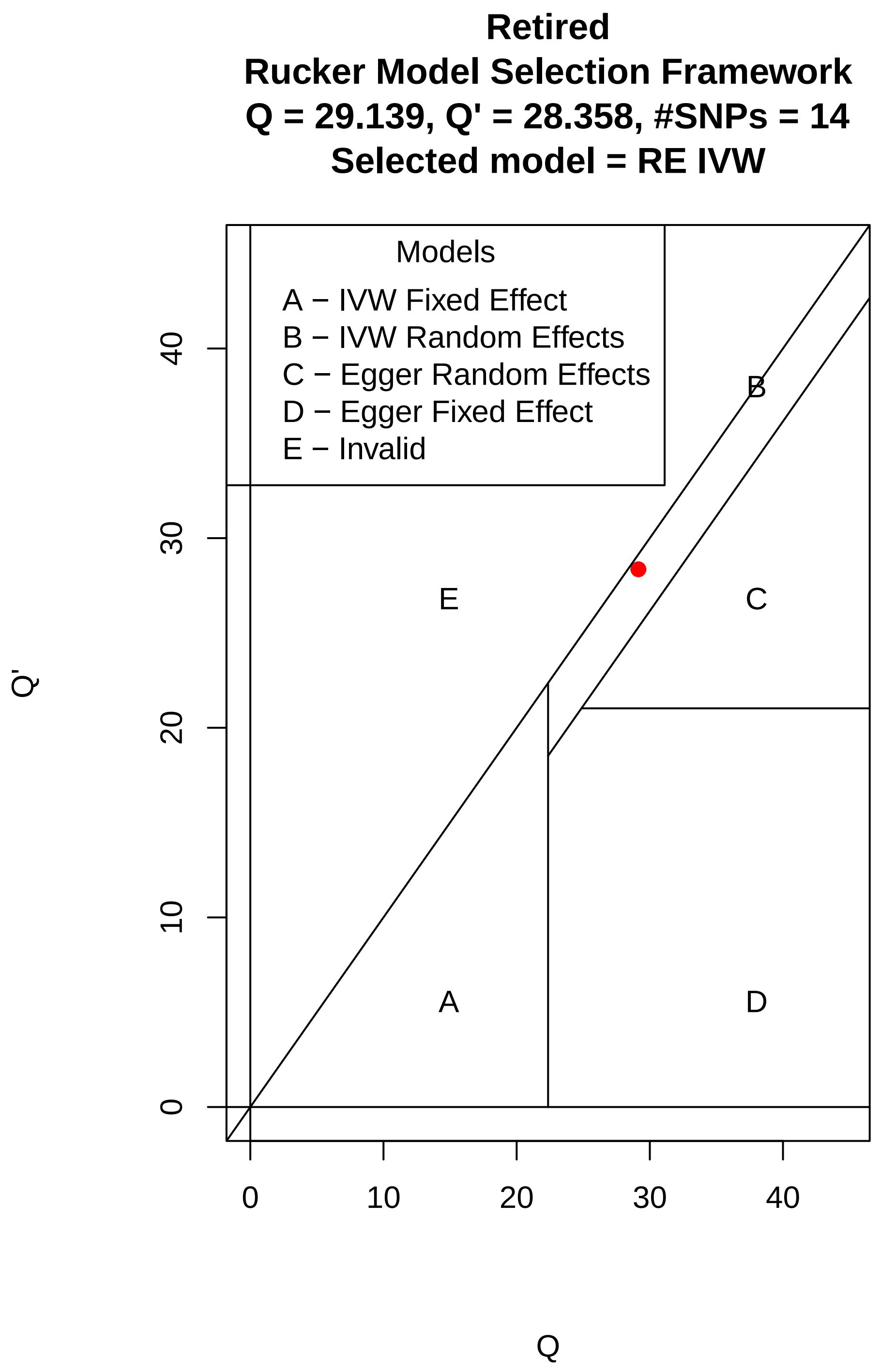
Retired
Rucker Model Selection Framework
Q = 29.139, Q' = 28.358, #SNPs = 14
Selected model = RE IVW
Models
A − IVW Fixed Effect
B − IVW Random Effects
C − Egger Random Effects
D − Egger Fixed Effect
E − Invalid
A
B
C
D
E
Q'
Q
0
10
20
30
40

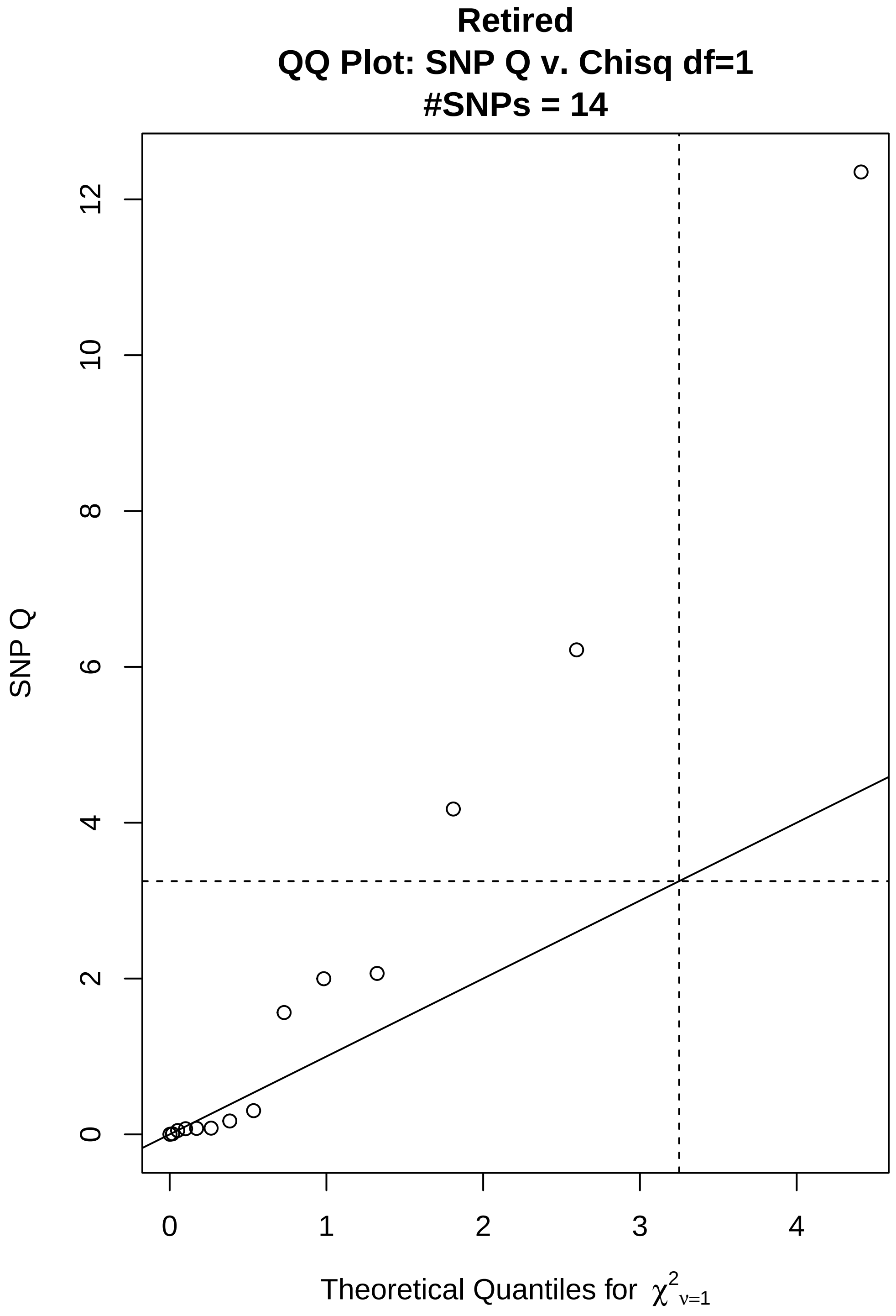
Retired
QQ Plot: SNP Q v. Chisq df=1
#SNPs = 14
SNP Q
Theoretical Quantiles for $\chi^2_{\nu=1}$
0
2
4
6
8
10
12
0
1
2
3
4

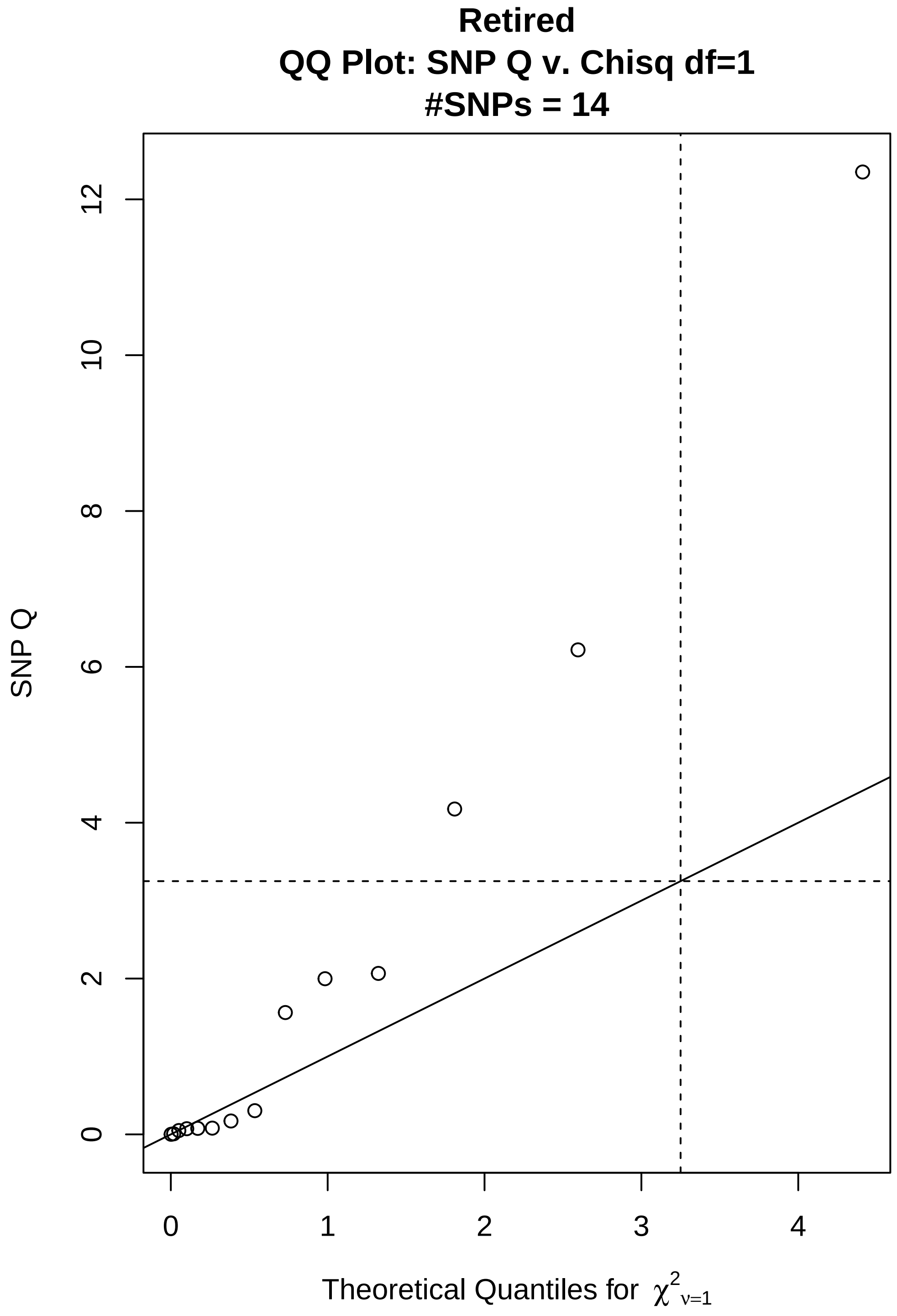
Retired
QQ Plot: SNP Q v. Chisq df=1
#SNPs = 14
SNP Q
Theoretical Quantiles for $\chi^2_{\nu=1}$
0
2
4
6
8
10
12
0
1
2
3
4
