# Supplementary material for: Effects of alcohol consumption on employment and social outcomes: a Mendelian randomisation study
Source: Alcohol Alcohol. 2025 Jul 18;60(5):agaf038. doi: 10.1093/alcalc/agaf038 (PMC12271571; doi:10.1093/alcalc/agaf038)
Supplement: Campbell_Green_Davies_et_al_2025_agaf038 [file campbell_green_davies_et_al_2025_agaf038.zip › Campbell_Green_Davies_et_al_2025/Female/drink/do2SampleMrAnalyses_alcoholUnitsWeekly_wins140_highestEducAttainment_ageCentreGpc.pdf]

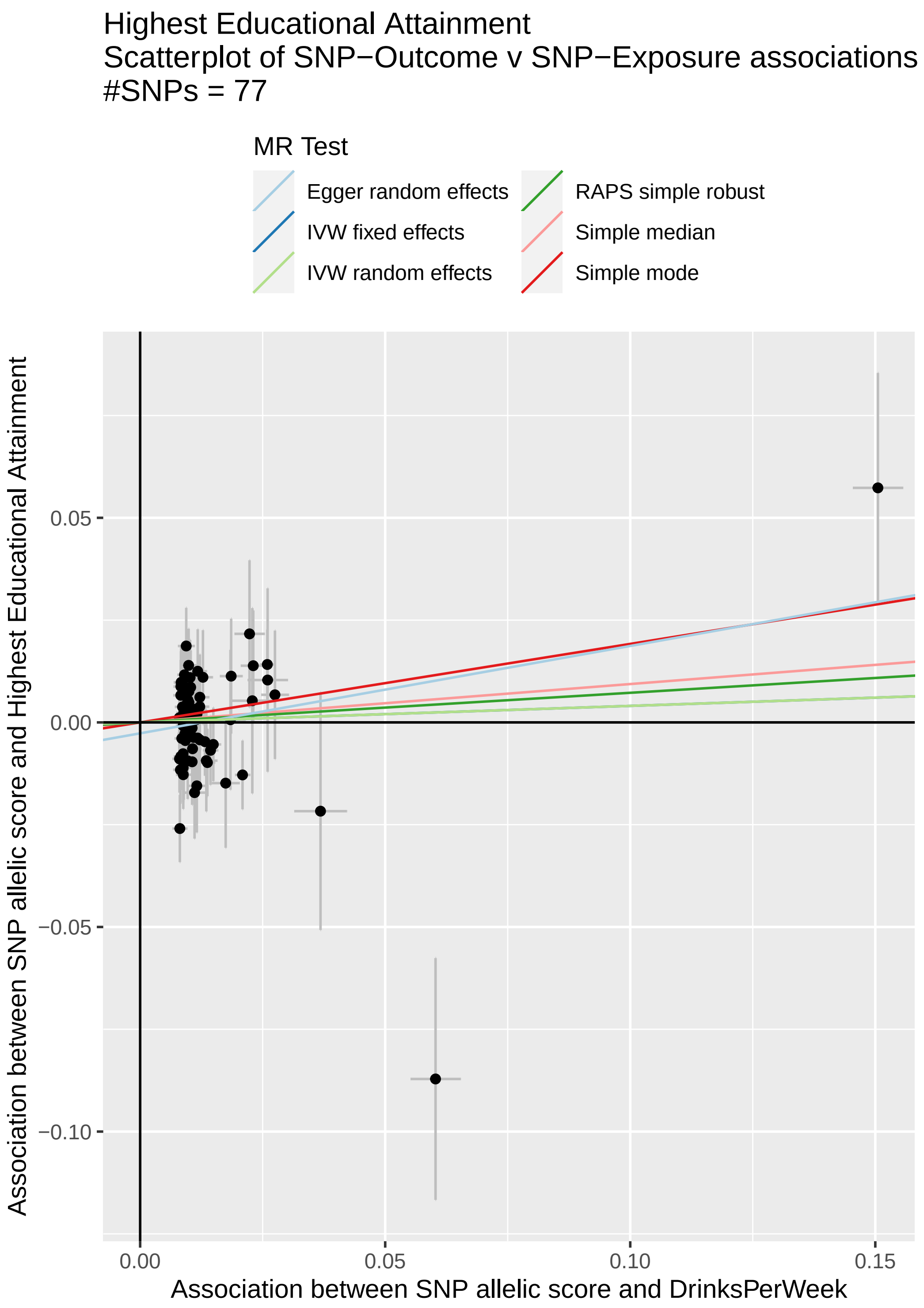
Highest Educational Attainment
Scatterplot of SNP−Outcome v SNP−Exposure associations
#SNPs = 77
MR Test
Egger random effects
IVW fixed effects
IVW random effects
RAPS simple robust
Simple median
Simple mode
Association between SNP allelic score and Highest Educational Attainment
0.05
0.00
−0.05
−0.10
0.00
0.05
0.10
0.15
Association between SNP allelic score and DrinksPerWeek

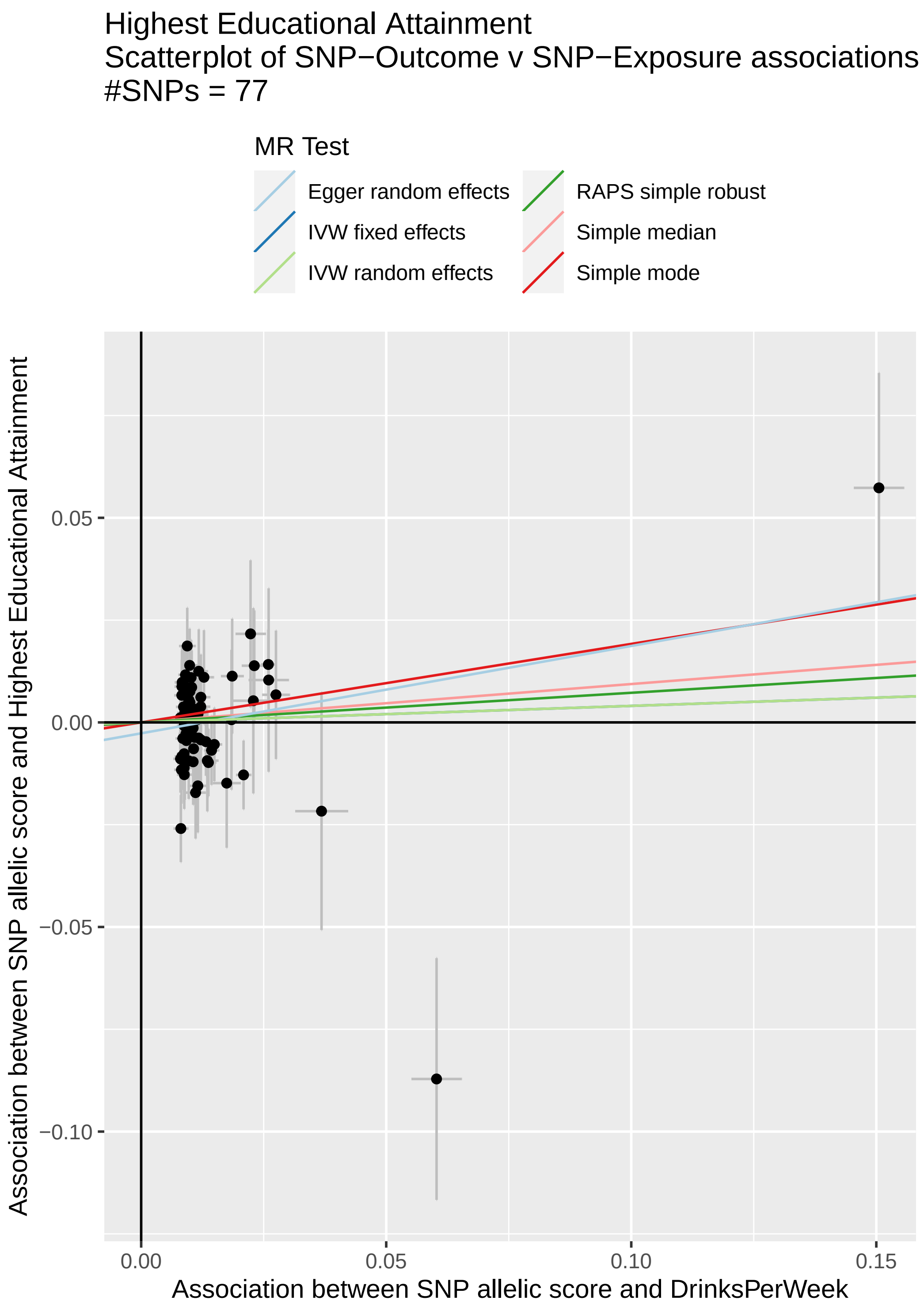
Highest Educational Attainment
Scatterplot of SNP−Outcome v SNP−Exposure associations
#SNPs = 77
MR Test
Egger random effects
IVW fixed effects
IVW random effects
RAPS simple robust
Simple median
Simple mode
Association between SNP allelic score and Highest Educational Attainment
0.05
0.00
−0.05
−0.10
0.00
0.05
0.10
0.15
Association between SNP allelic score and DrinksPerWeek

Highest Educational Attainment
Causal Effect estimates for alcoholUnitsWeekly_wins140 on Highest Educational Attainment
#SNPs = 77, #Outlier SNPs removed = 0

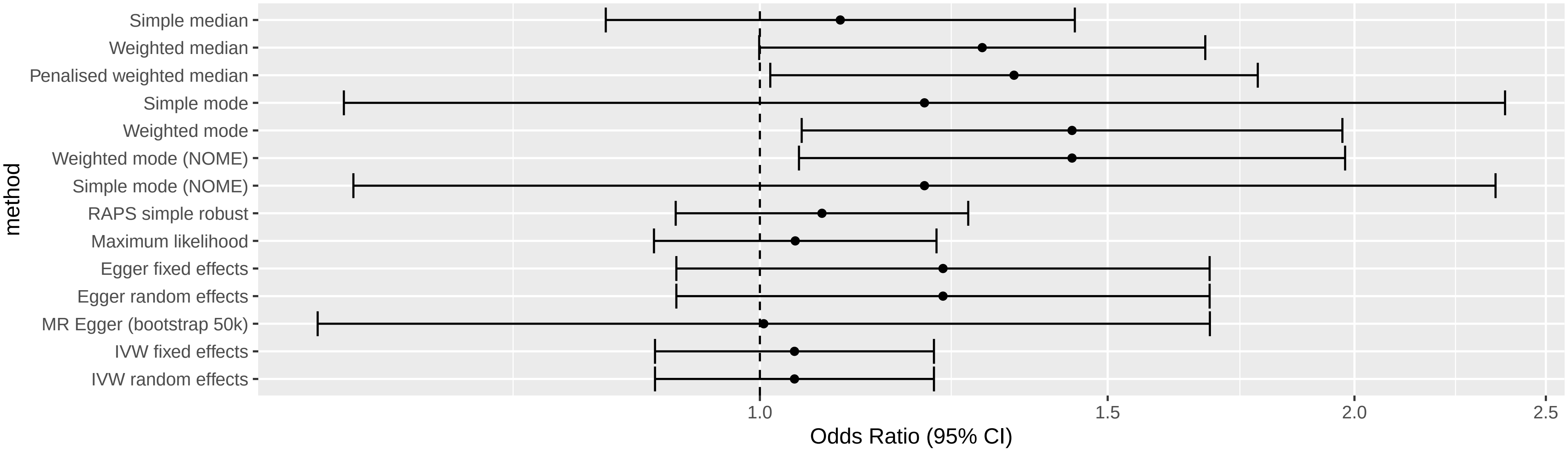

Highest Educational Attainment
Causal Effect estimates for alcoholUnitsWeekly_wins140 on Highest Educational Attainment
#SNPs = 77, #Outlier SNPs removed = 0

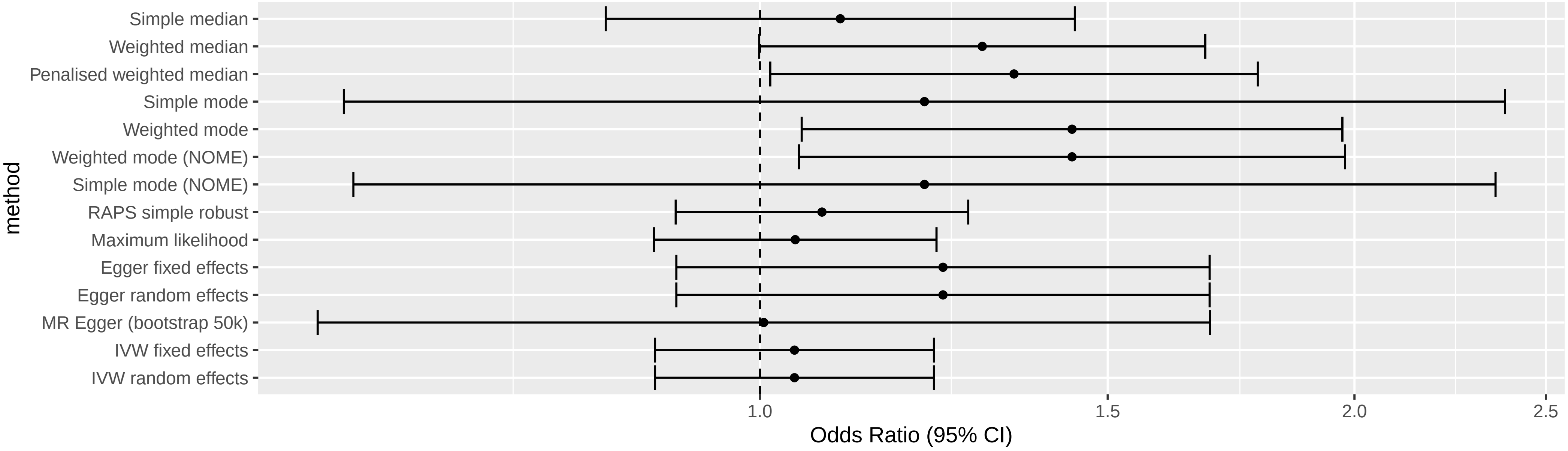

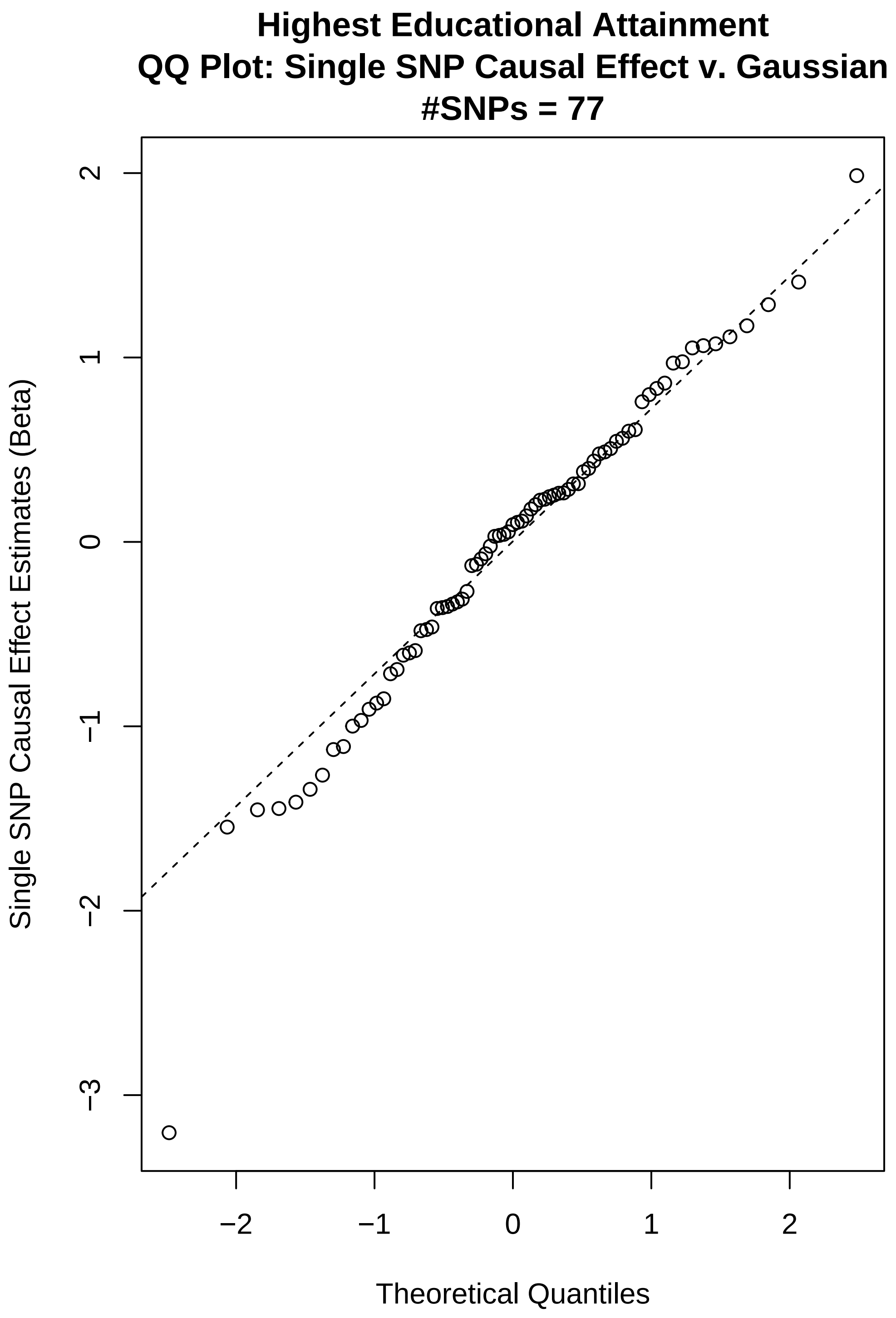
Highest Educational Attainment
QQ Plot: Single SNP Causal Effect v. Gaussian
#SNPs = 77
Single SNP Causal Effect Estimates (Beta)
Theoretical Quantiles
−3
−2
−1
0
1
2
−2
−1
0
1
2

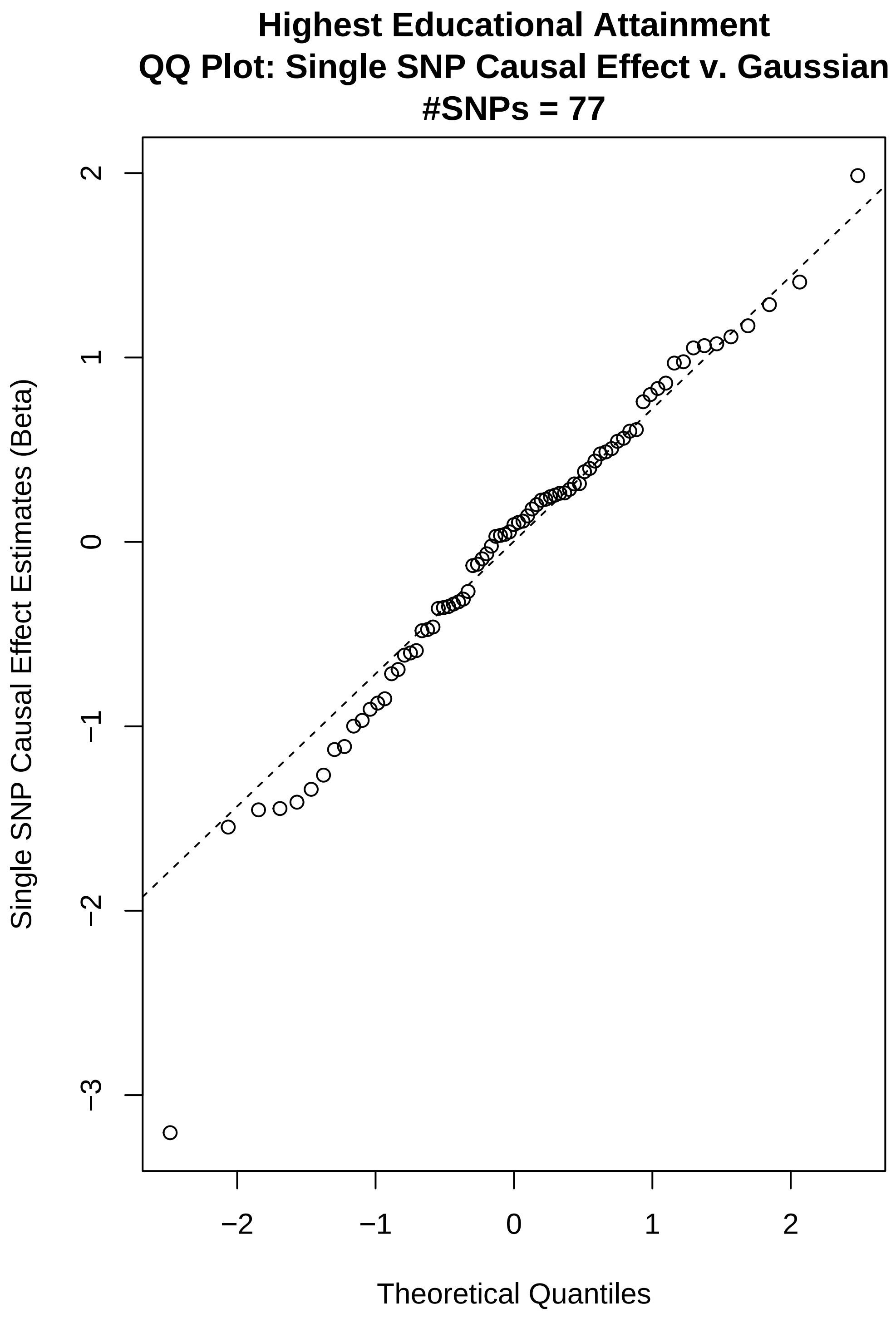
Highest Educational Attainment
QQ Plot: Single SNP Causal Effect v. Gaussian
#SNPs = 77
Single SNP Causal Effect Estimates (Beta)
Theoretical Quantiles
−3
−2
−1
0
1
2
−2
−1
0
1
2

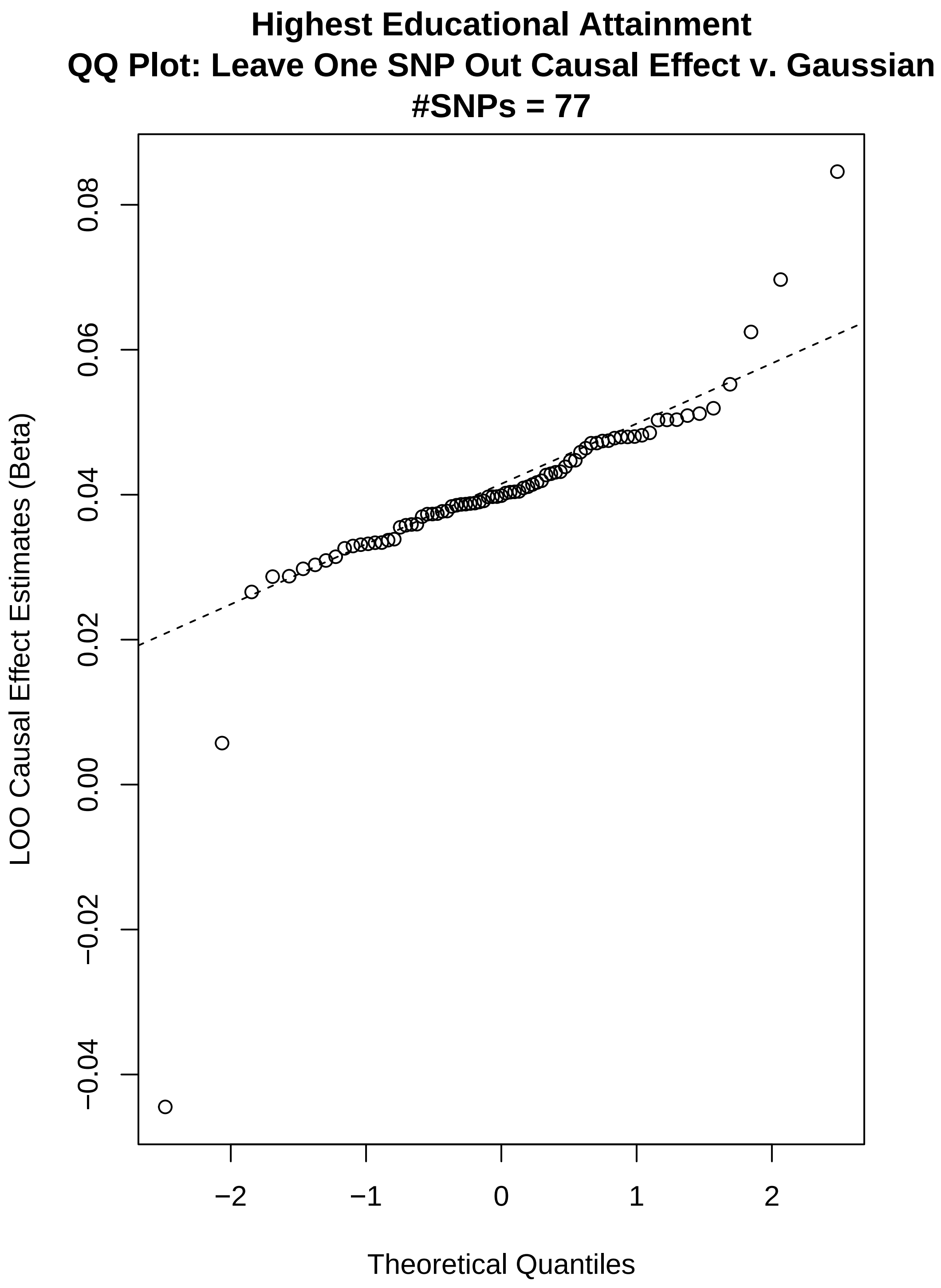
Highest Educational Attainment
QQ Plot: Leave One SNP Out Causal Effect v. Gaussian
#SNPs = 77
LOO Causal Effect Estimates (Beta)
Theoretical Quantiles
−0.04
−0.02
0.00
0.02
0.04
0.06
0.08
−2
−1
0
1
2

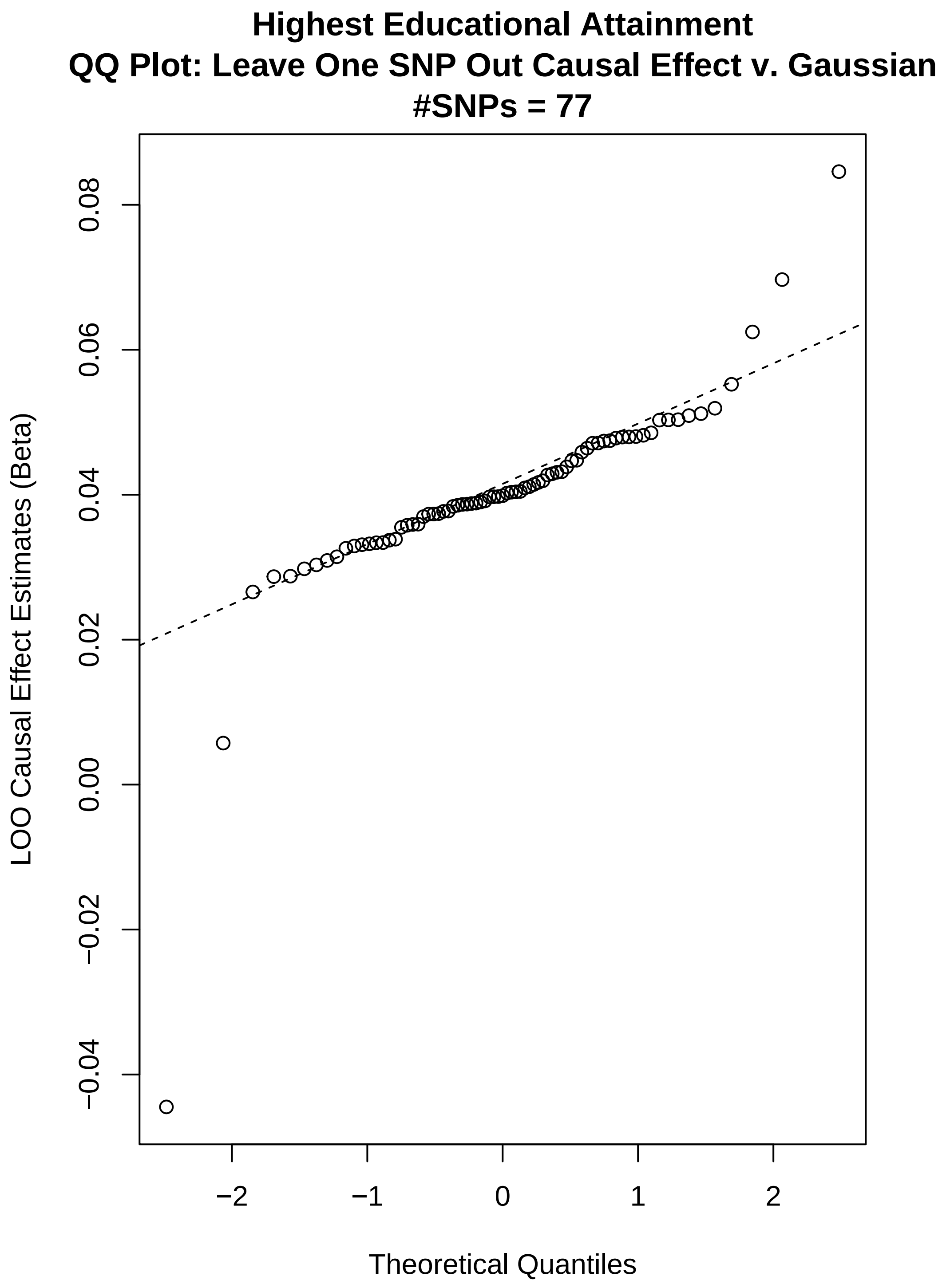
Highest Educational Attainment
QQ Plot: Leave One SNP Out Causal Effect v. Gaussian
#SNPs = 77
LOO Causal Effect Estimates (Beta)
Theoretical Quantiles
−0.04
−0.02
0.00
0.02
0.04
0.06
0.08
−2
−1
0
1
2

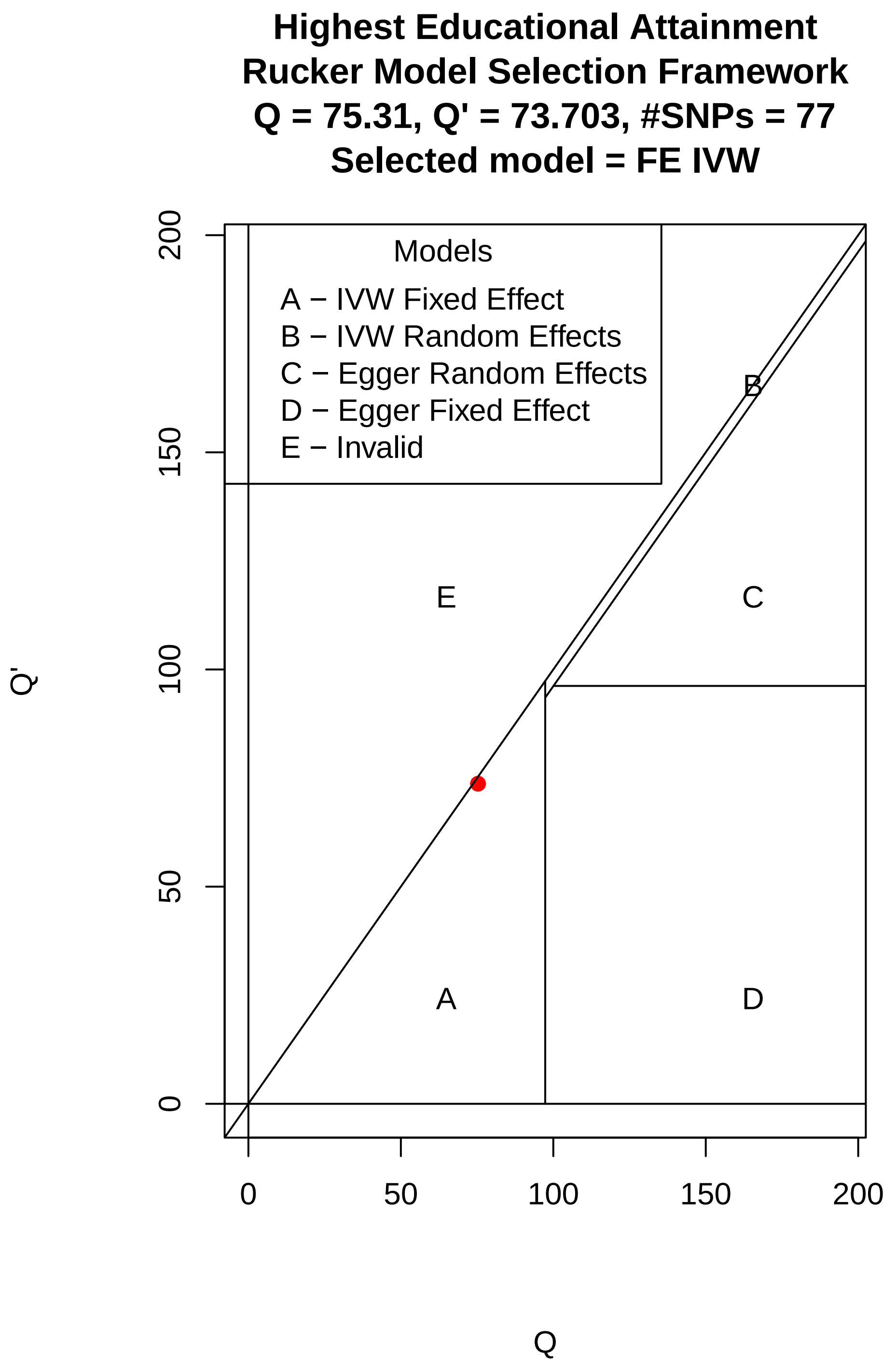
Highest Educational Attainment
Rucker Model Selection Framework
Q = 75.31, Q' = 73.703, #SNPs = 77
Selected model = FE IVW
Models
A − IVW Fixed Effect
B − IVW Random Effects
C − Egger Random Effects
D − Egger Fixed Effect
E − Invalid
Q'
Q
0
50
100
150
200
A
B
C
D
E
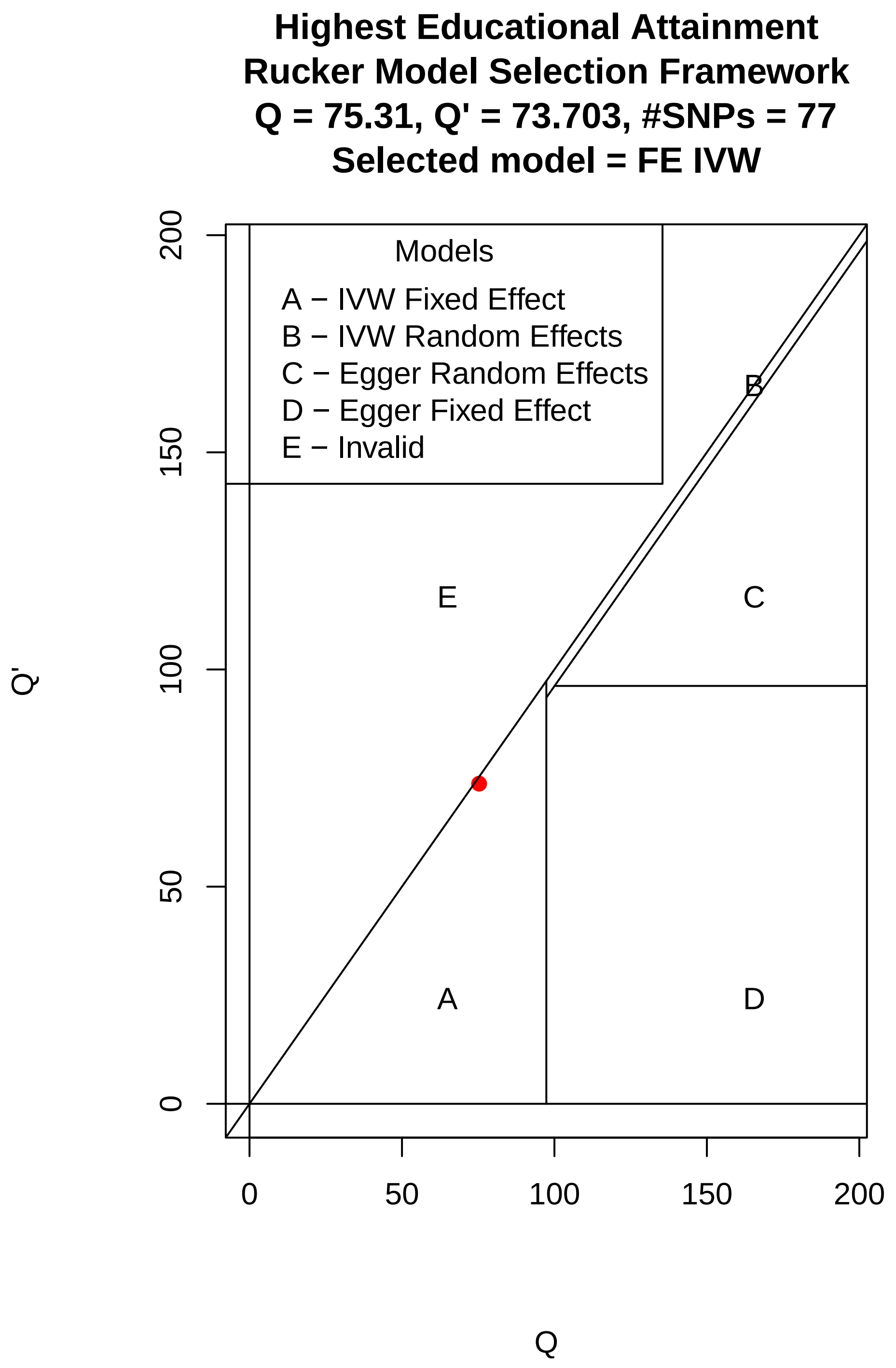
Highest Educational Attainment
Rucker Model Selection Framework
Q = 75.31, Q' = 73.703, #SNPs = 77
Selected model = FE IVW
Models
A − IVW Fixed Effect
B − IVW Random Effects
C − Egger Random Effects
D − Egger Fixed Effect
E − Invalid
Q'
Q
0
50
100
150
200
A
B
C
D
E

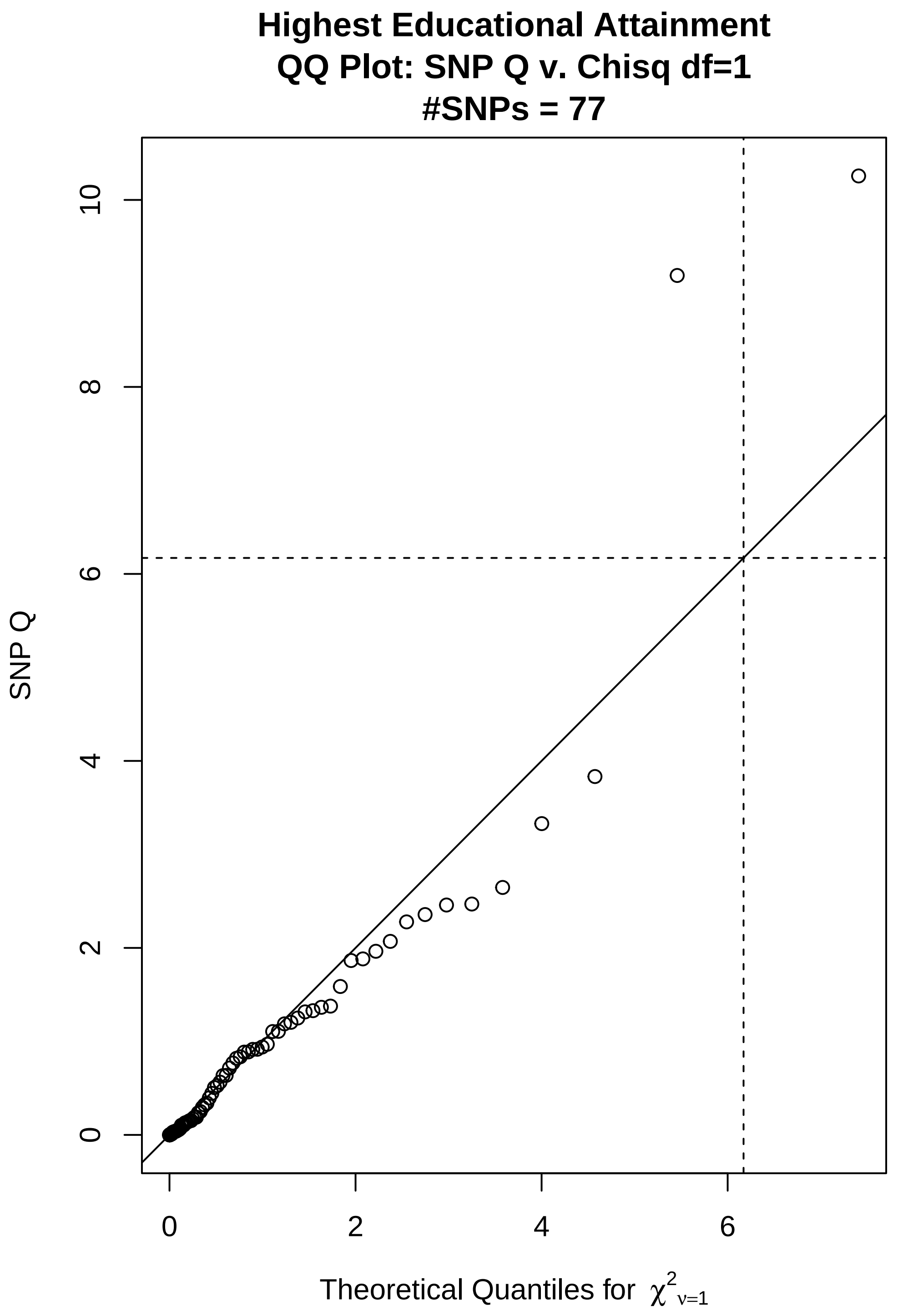
Highest Educational Attainment
QQ Plot: SNP Q v. Chisq df=1
#SNPs = 77
SNP Q
0
2
4
6
8
10
0
2
4
6
Theoretical Quantiles for $\chi^2_{\nu=1}$

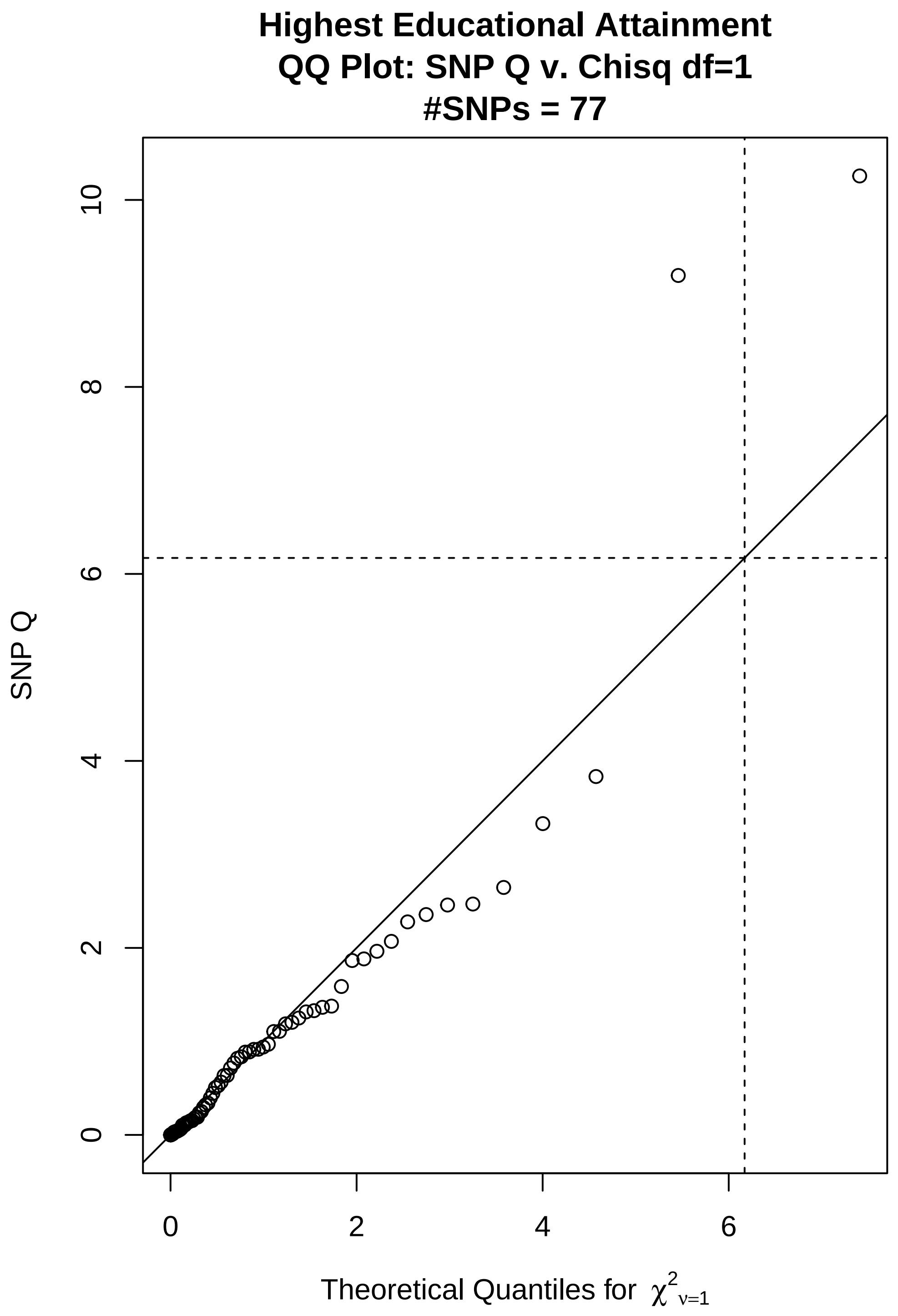
Highest Educational Attainment
QQ Plot: SNP Q v. Chisq df=1
#SNPs = 77
SNP Q
0
2
4
6
8
10
0
2
4
6
Theoretical Quantiles for $\chi^2_{\nu=1}$
