# Supplementary material for: Effects of alcohol consumption on employment and social outcomes: a Mendelian randomisation study
Source: Alcohol Alcohol. 2025 Jul 18;60(5):agaf038. doi: 10.1093/alcalc/agaf038 (PMC12271571; doi:10.1093/alcalc/agaf038)
Supplement: Campbell_Green_Davies_et_al_2025_agaf038 [file campbell_green_davies_et_al_2025_agaf038.zip › Campbell_Green_Davies_et_al_2025/Female/drink/do2SampleMrAnalyses_alcoholUnitsWeekly_wins140_iUnempNotEmp_ageCentreGpc.pdf]

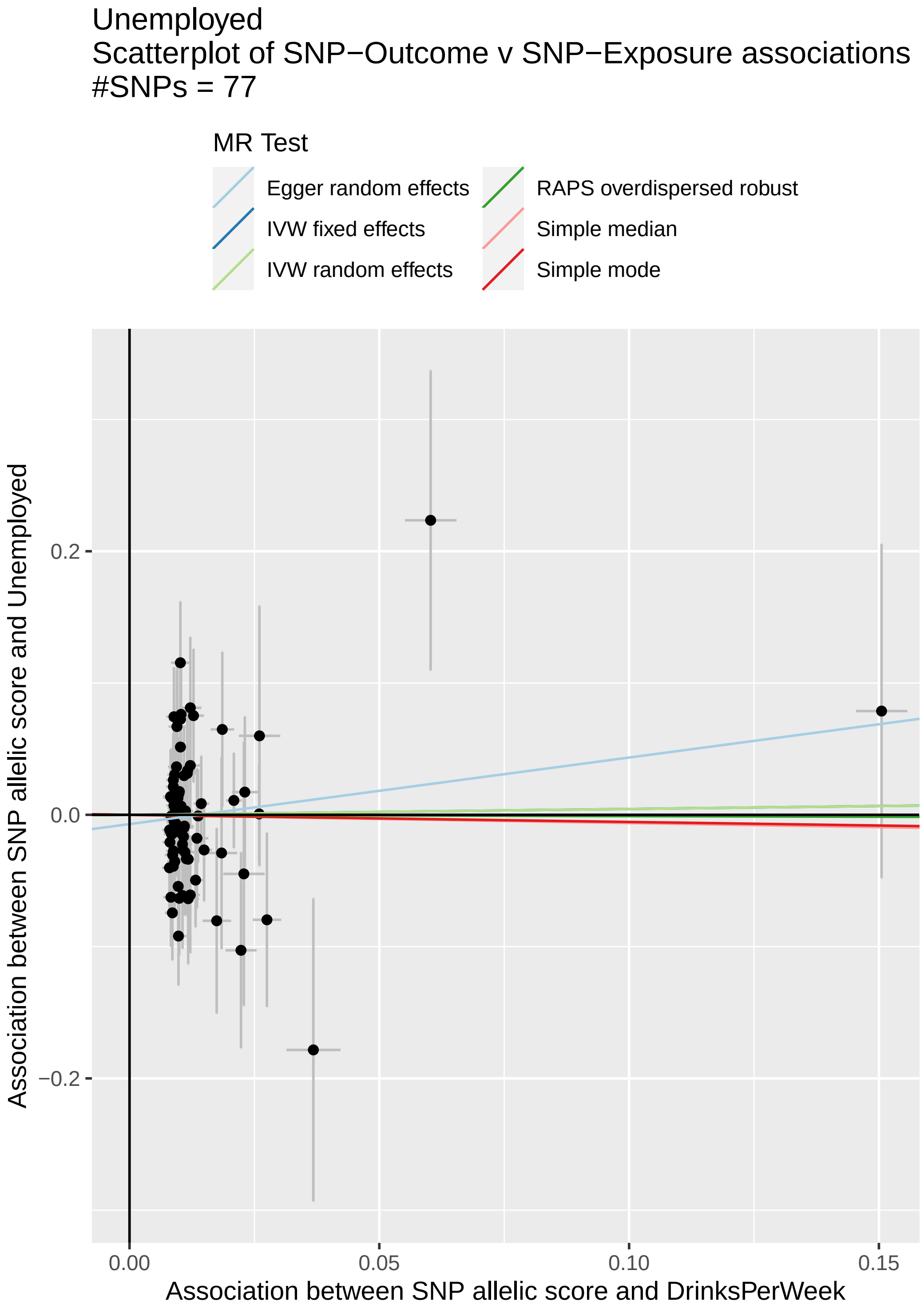
Unemployed
Scatterplot of SNP−Outcome v SNP−Exposure associations
#SNPs = 77
MR Test
Egger random effects
IVW fixed effects
IVW random effects
RAPS overdispersed robust
Simple median
Simple mode
Association between SNP allelic score and Unemployed
0.2
0.0
−0.2
0.00
0.05
0.10
0.15
Association between SNP allelic score and DrinksPerWeek

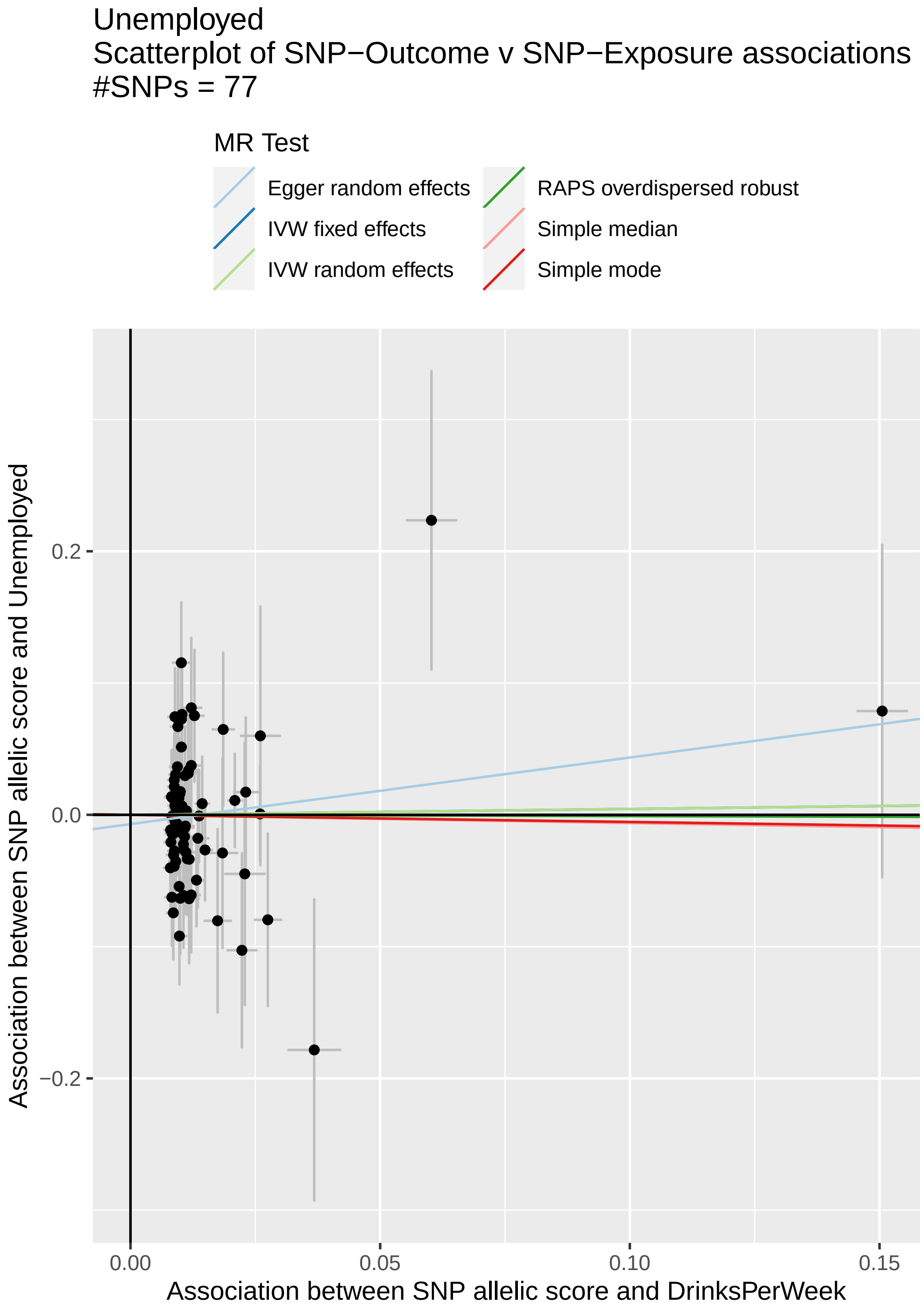
Unemployed
Scatterplot of SNP−Outcome v SNP−Exposure associations
#SNPs = 77
MR Test
Egger random effects
IVW fixed effects
IVW random effects
RAPS overdispersed robust
Simple median
Simple mode
Association between SNP allelic score and Unemployed
0.2
0.0
−0.2
0.00
0.05
0.10
0.15
Association between SNP allelic score and DrinksPerWeek

Unemployed
Causal Effect estimates for alcoholUnitsWeekly_wins140 on Unemployed
#SNPs = 77, #Outlier SNPs removed = 0

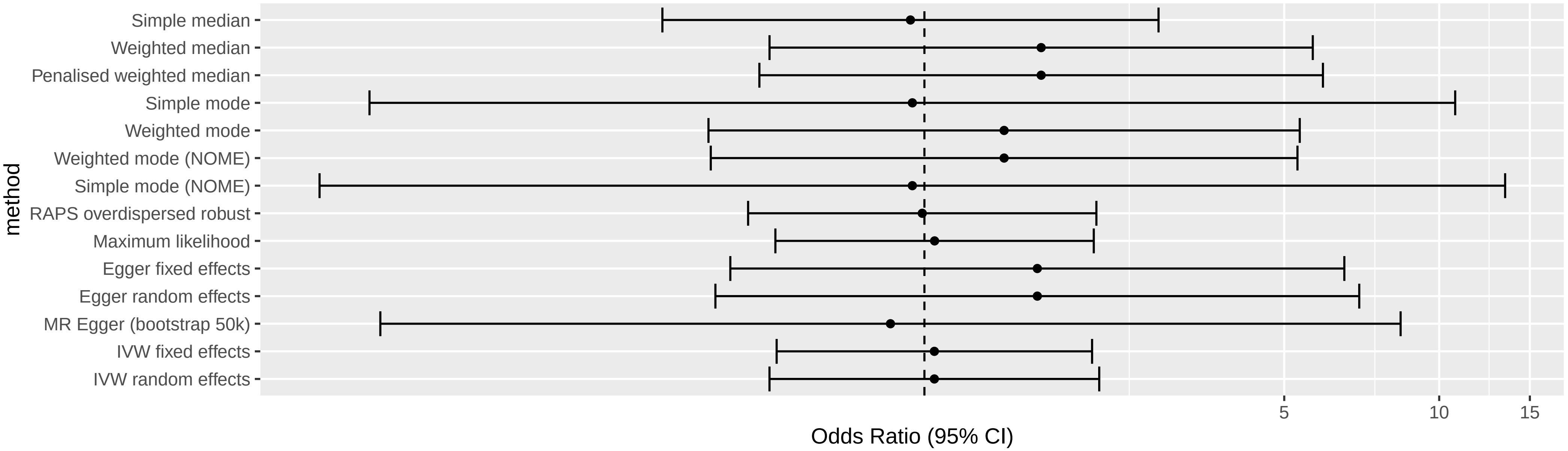

Unemployed
Causal Effect estimates for alcoholUnitsWeekly_wins140 on Unemployed
#SNPs = 77, #Outlier SNPs removed = 0

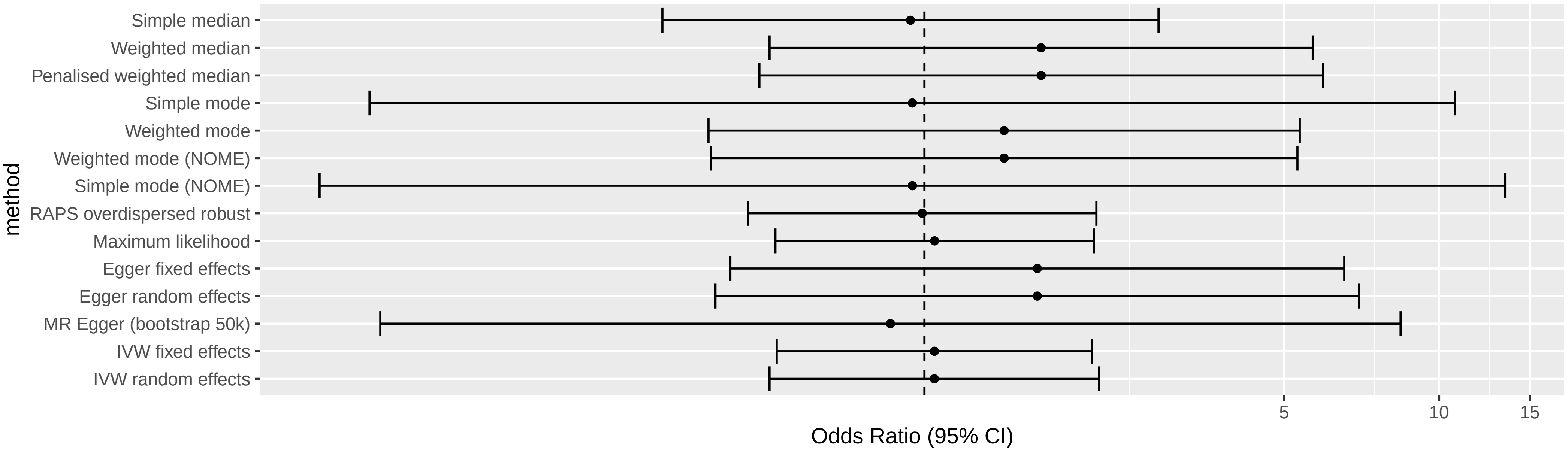

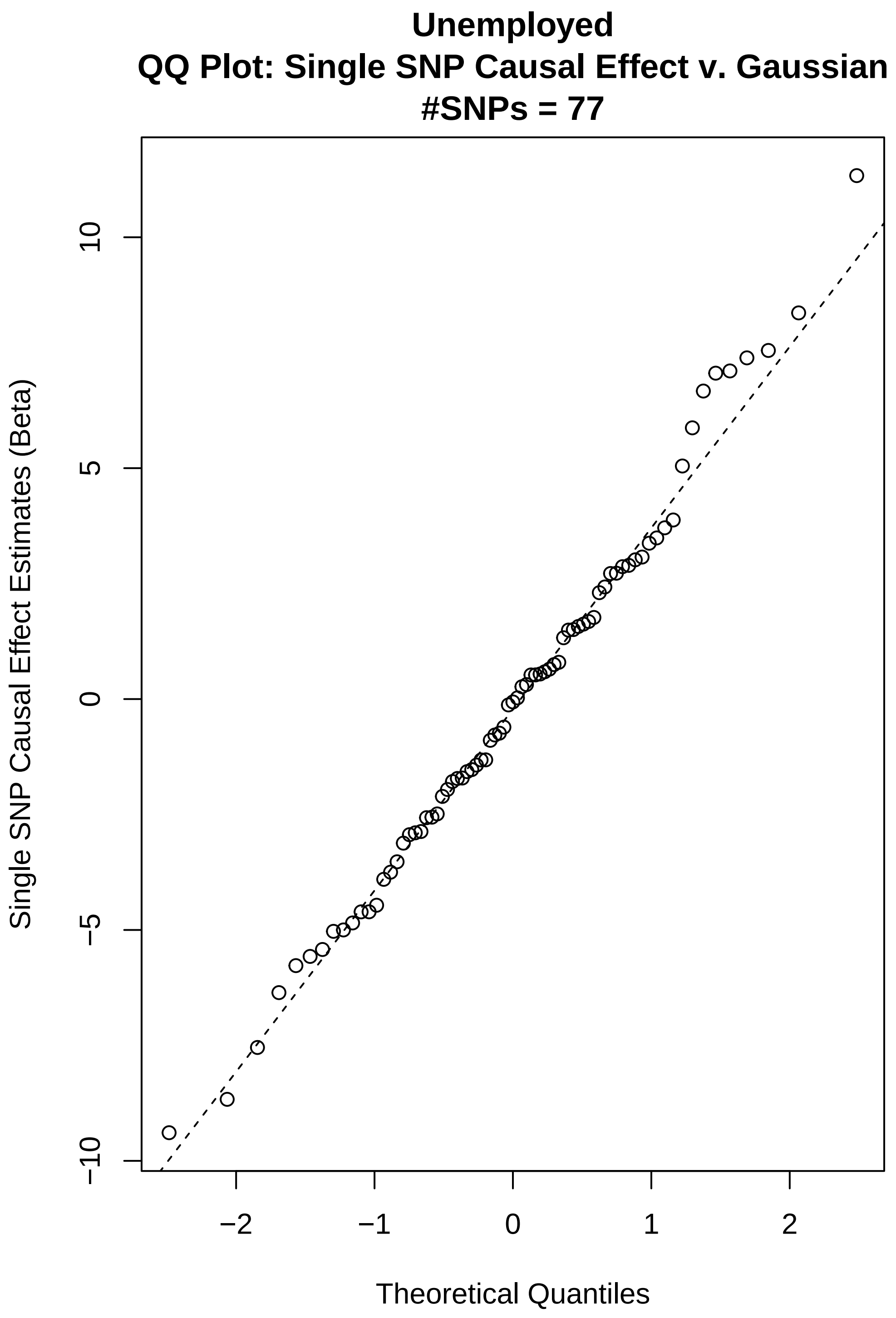
Unemployed
QQ Plot: Single SNP Causal Effect v. Gaussian
#SNPs = 77
Single SNP Causal Effect Estimates (Beta)
Theoretical Quantiles
−10
−5
0
5
10
−2
−1
0
1
2

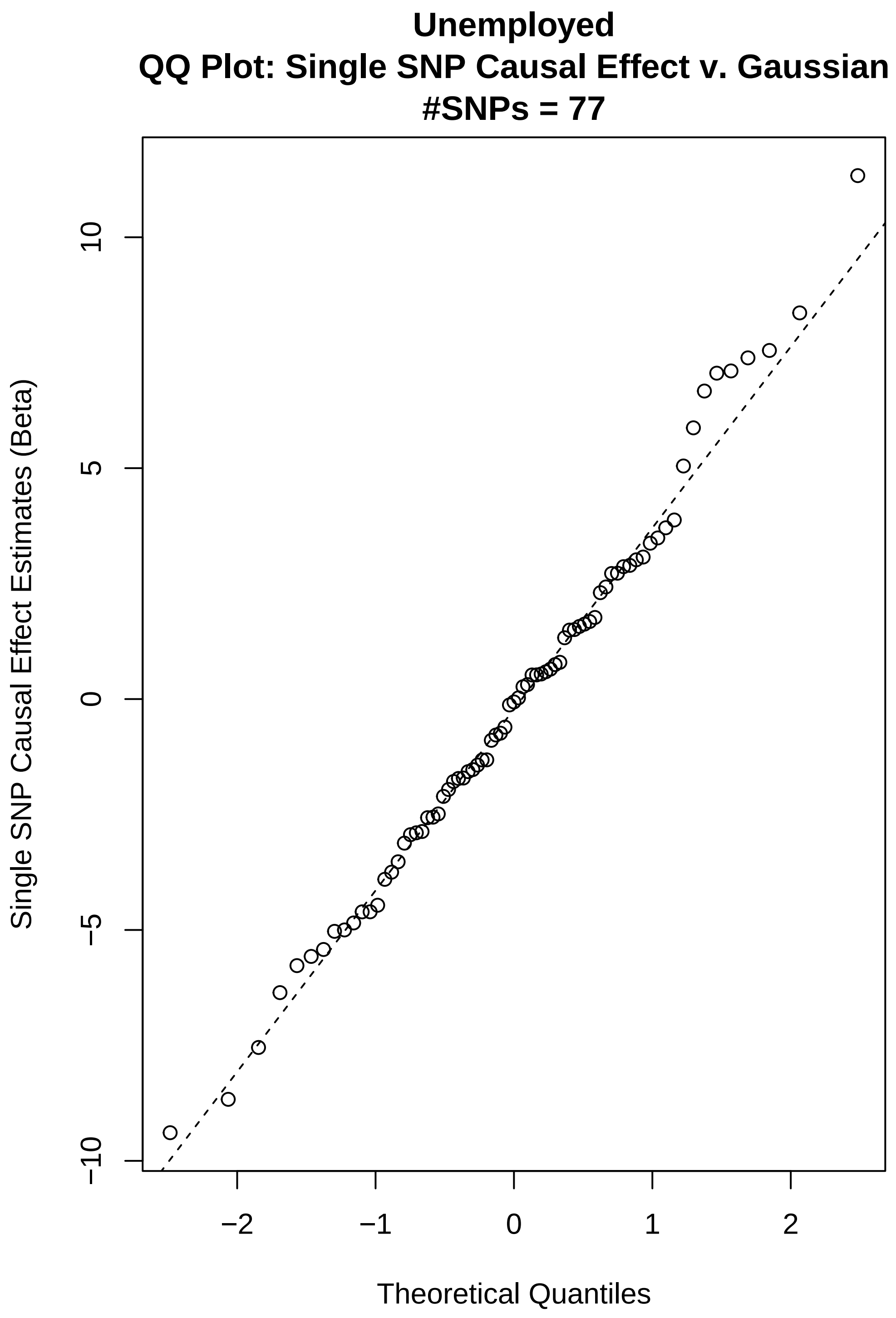
Unemployed
QQ Plot: Single SNP Causal Effect v. Gaussian
#SNPs = 77
Single SNP Causal Effect Estimates (Beta)
Theoretical Quantiles
−10
−5
0
5
10
−2
−1
0
1
2

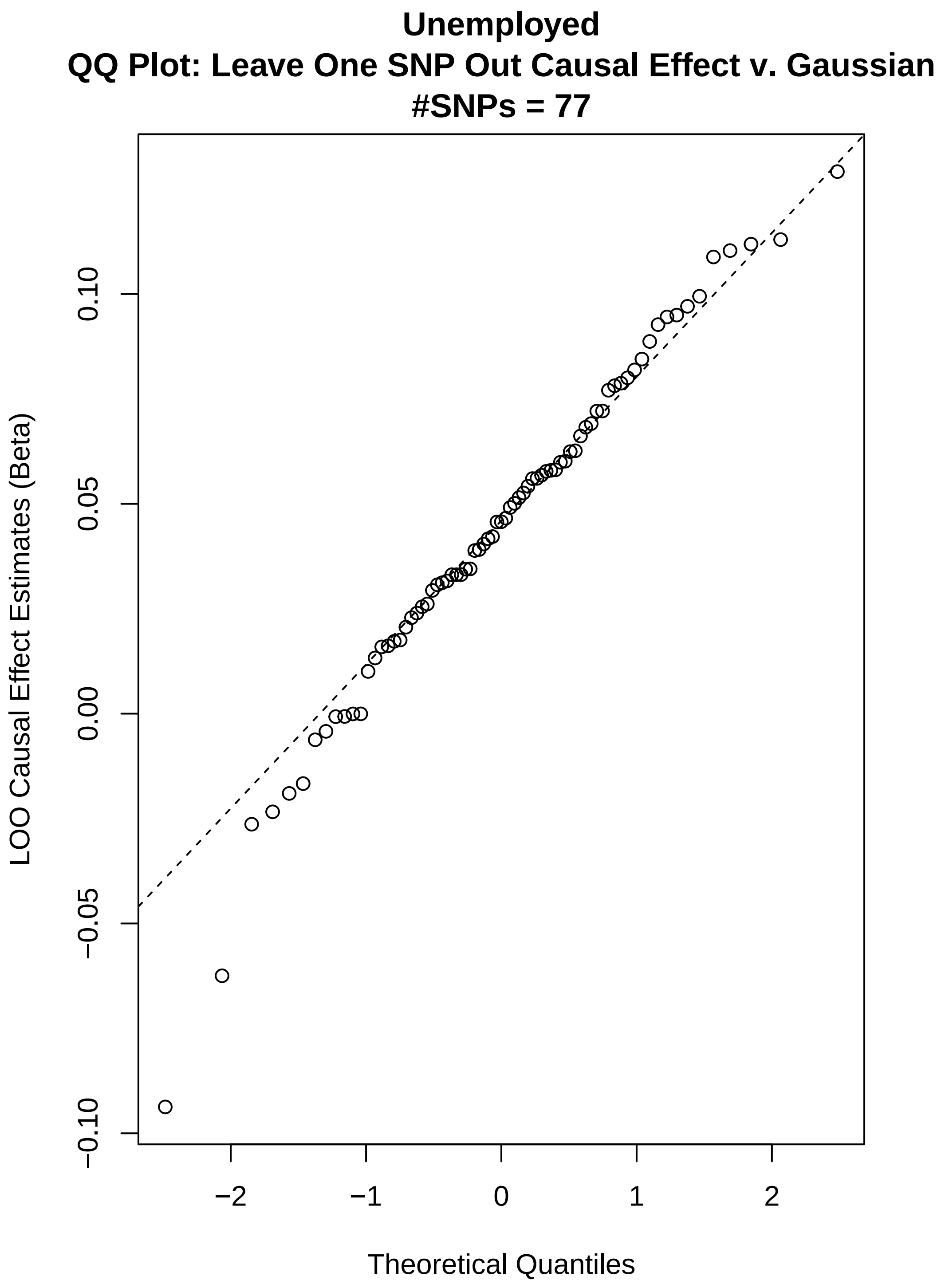
Unemployed
QQ Plot: Leave One SNP Out Causal Effect v. Gaussian
#SNPs = 77
LOO Causal Effect Estimates (Beta)
−0.10
−0.05
0.00
0.05
0.10
−2
−1
0
1
2
Theoretical Quantiles

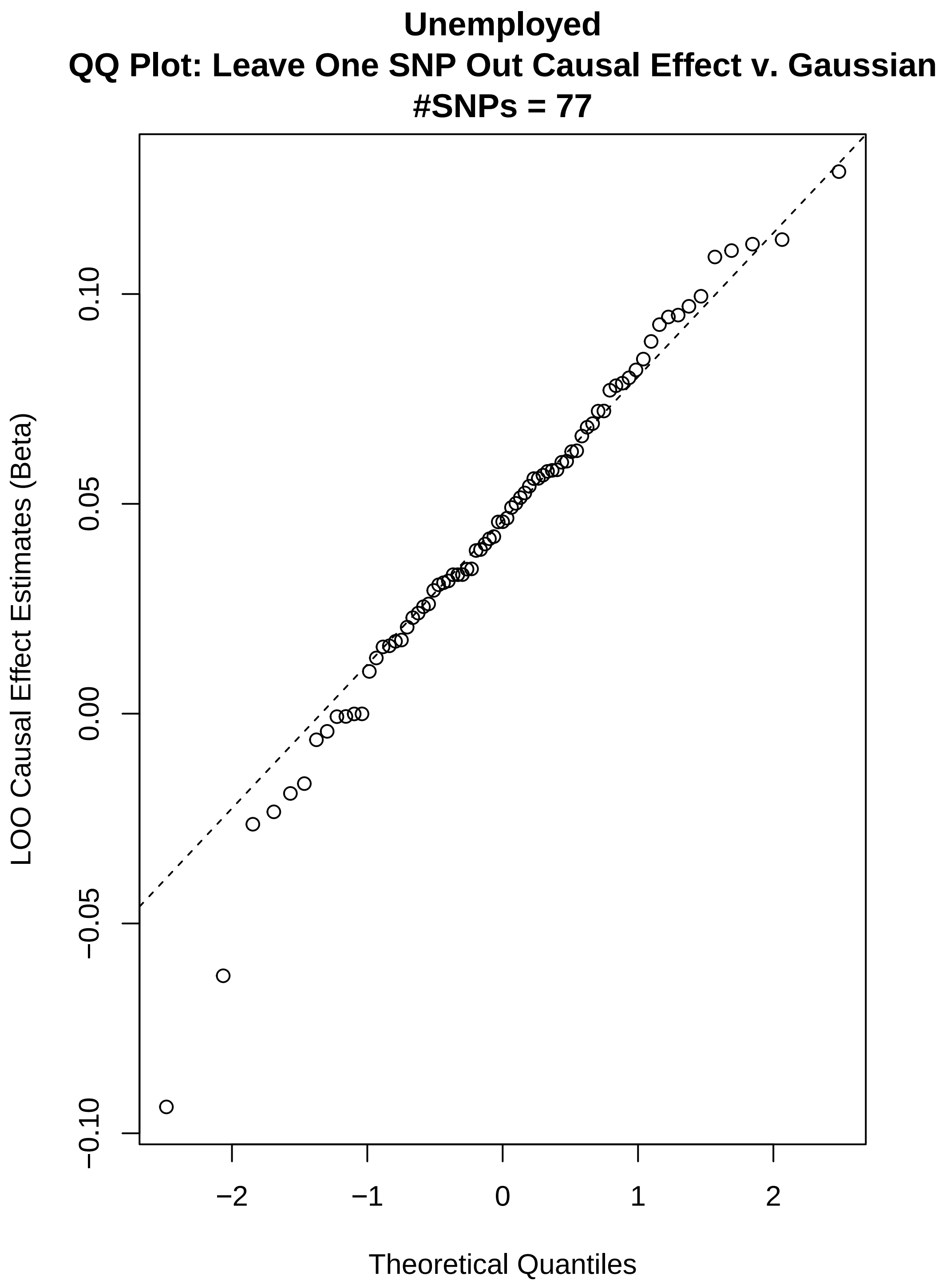
Unemployed
QQ Plot: Leave One SNP Out Causal Effect v. Gaussian
#SNPs = 77
LOO Causal Effect Estimates (Beta)
−0.10
−0.05
0.00
0.05
0.10
−2
−1
0
1
2
Theoretical Quantiles

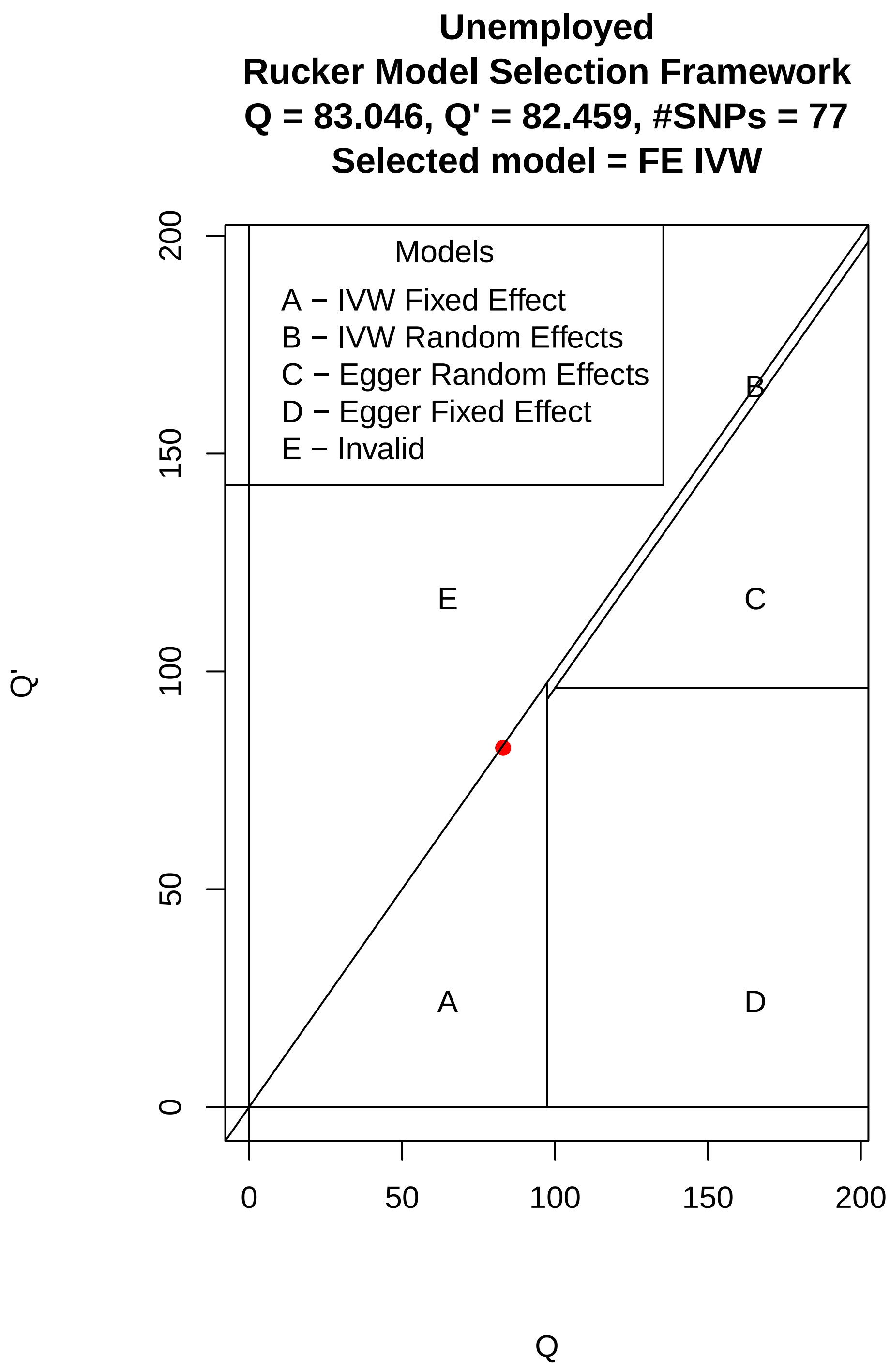
Unemployed
Rucker Model Selection Framework
Q = 83.046, Q' = 82.459, #SNPs = 77
Selected model = FE IVW
Models
A − IVW Fixed Effect
B − IVW Random Effects
C − Egger Random Effects
D − Egger Fixed Effect
E − Invalid
A
B
C
D
E
Q'
Q
0
50
100
150
200

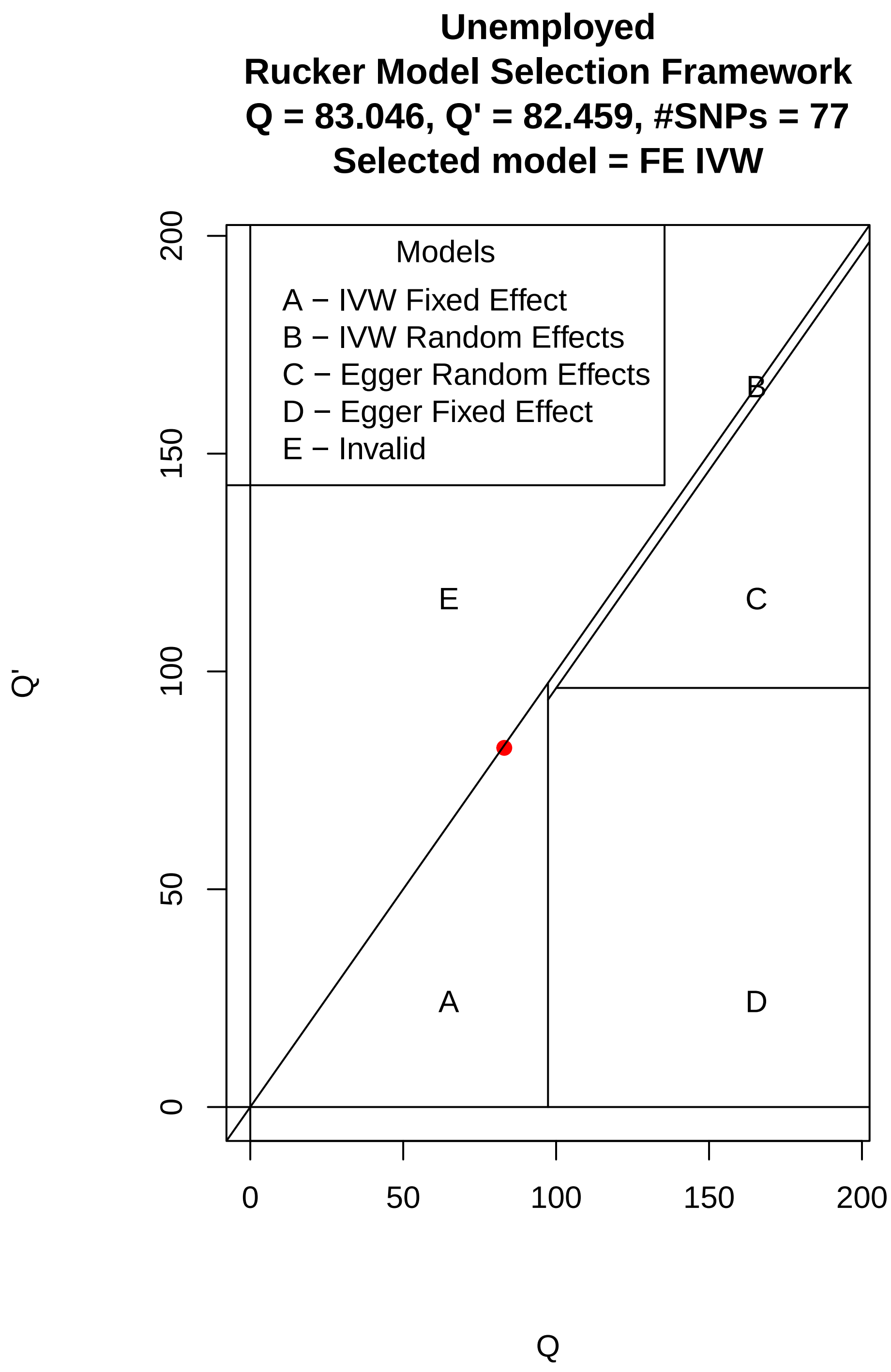
Unemployed
Rucker Model Selection Framework
Q = 83.046, Q' = 82.459, #SNPs = 77
Selected model = FE IVW
Models
A − IVW Fixed Effect
B − IVW Random Effects
C − Egger Random Effects
D − Egger Fixed Effect
E − Invalid
A
B
C
D
E
Q'
Q
0
50
100
150
200

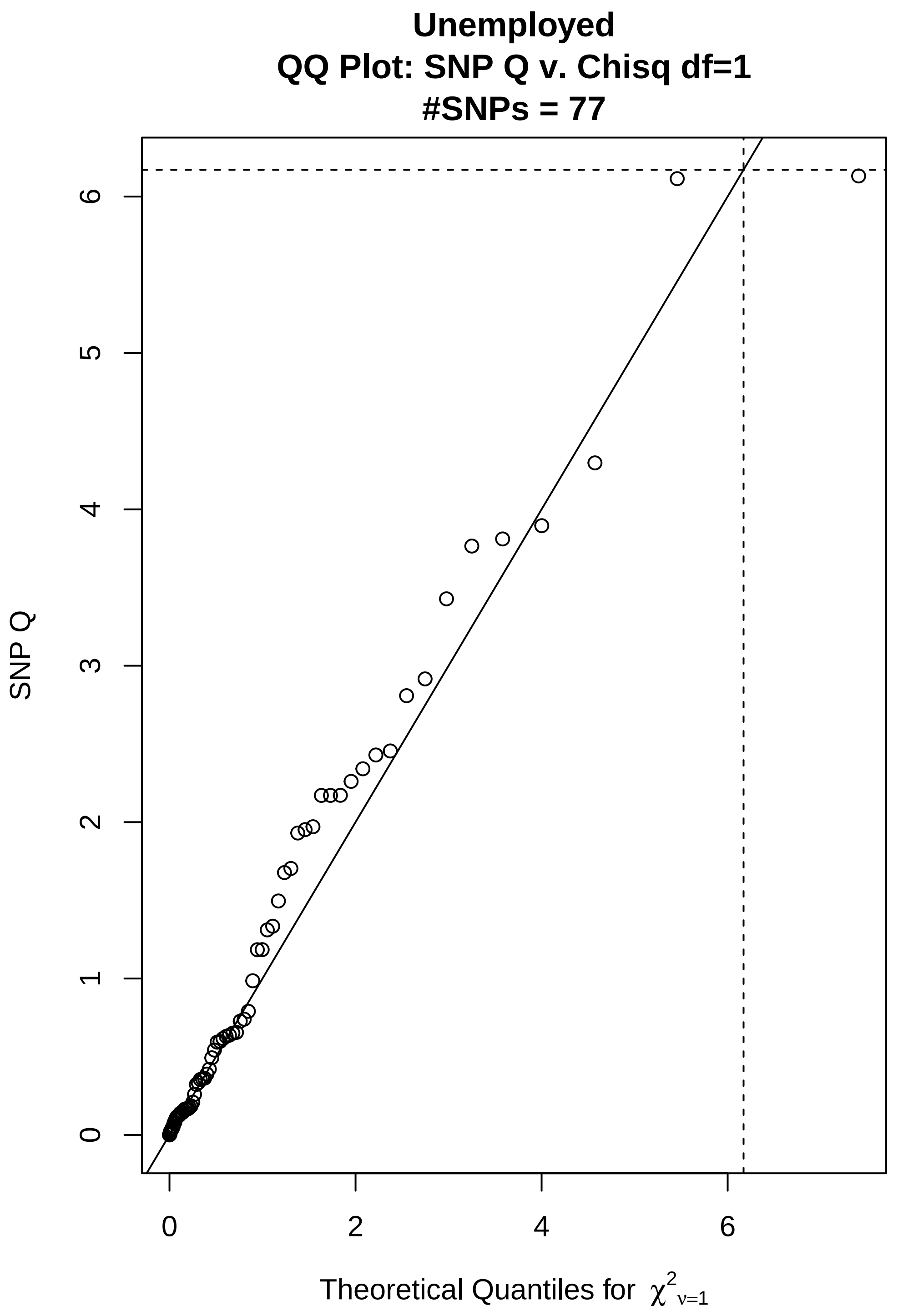
Unemployed
QQ Plot: SNP Q v. Chisq df=1
#SNPs = 77
SNP Q
0
1
2
3
4
5
6
0
2
4
6
Theoretical Quantiles for $\chi^2_{\nu=1}$

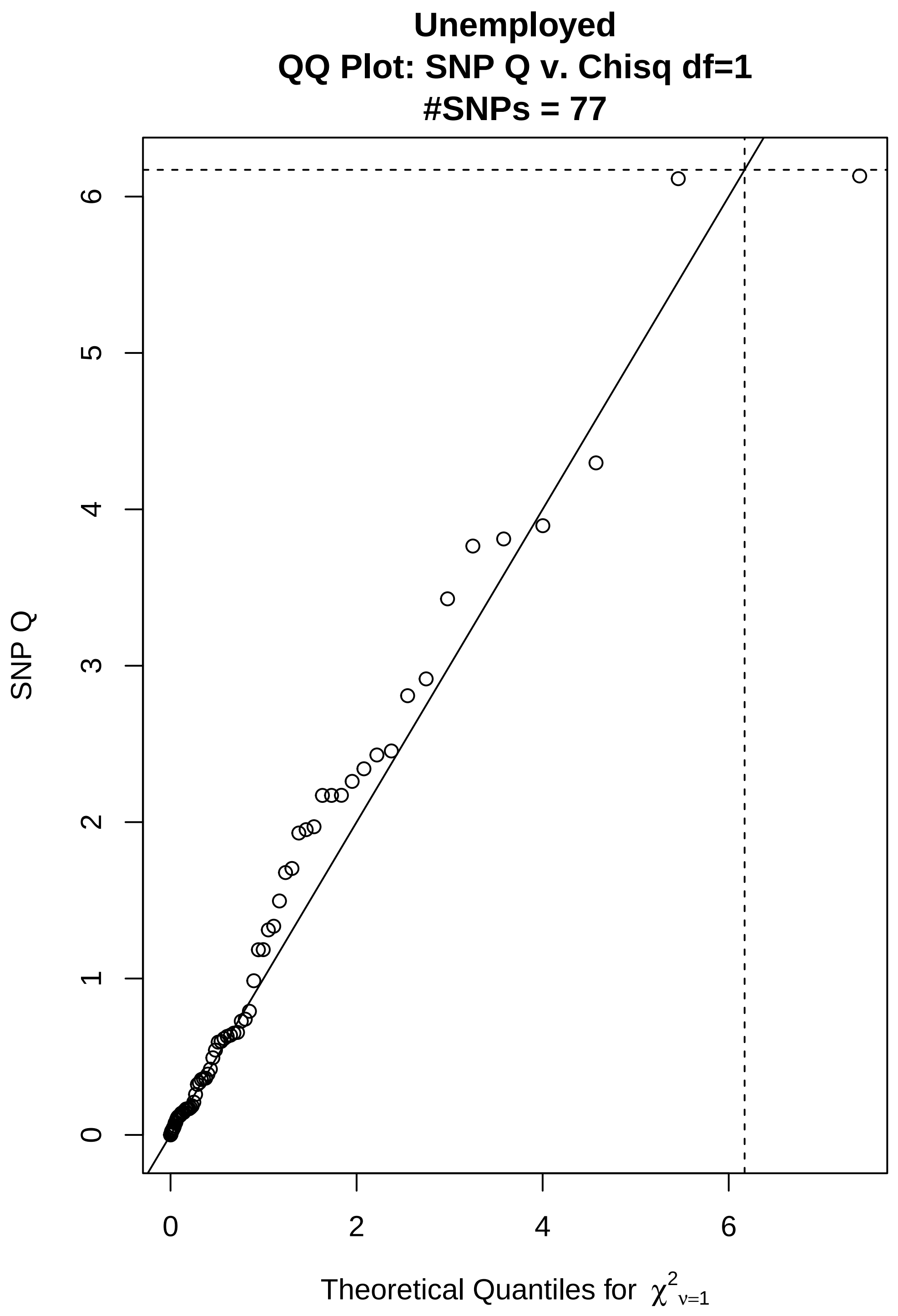
Unemployed
QQ Plot: SNP Q v. Chisq df=1
#SNPs = 77
SNP Q
0
1
2
3
4
5
6
0
2
4
6
Theoretical Quantiles for $\chi^2_{\nu=1}$
