# Supplementary material for: Effects of alcohol consumption on employment and social outcomes: a Mendelian randomisation study
Source: Alcohol Alcohol. 2025 Jul 18;60(5):agaf038. doi: 10.1093/alcalc/agaf038 (PMC12271571; doi:10.1093/alcalc/agaf038)
Supplement: Campbell_Green_Davies_et_al_2025_agaf038 [file campbell_green_davies_et_al_2025_agaf038.zip › Campbell_Green_Davies_et_al_2025/Female/drink/do2SampleMrAnalyses_alcoholUnitsWeekly_wins140_workHoursWeekly_wins84_ageCentreGpc.pdf]

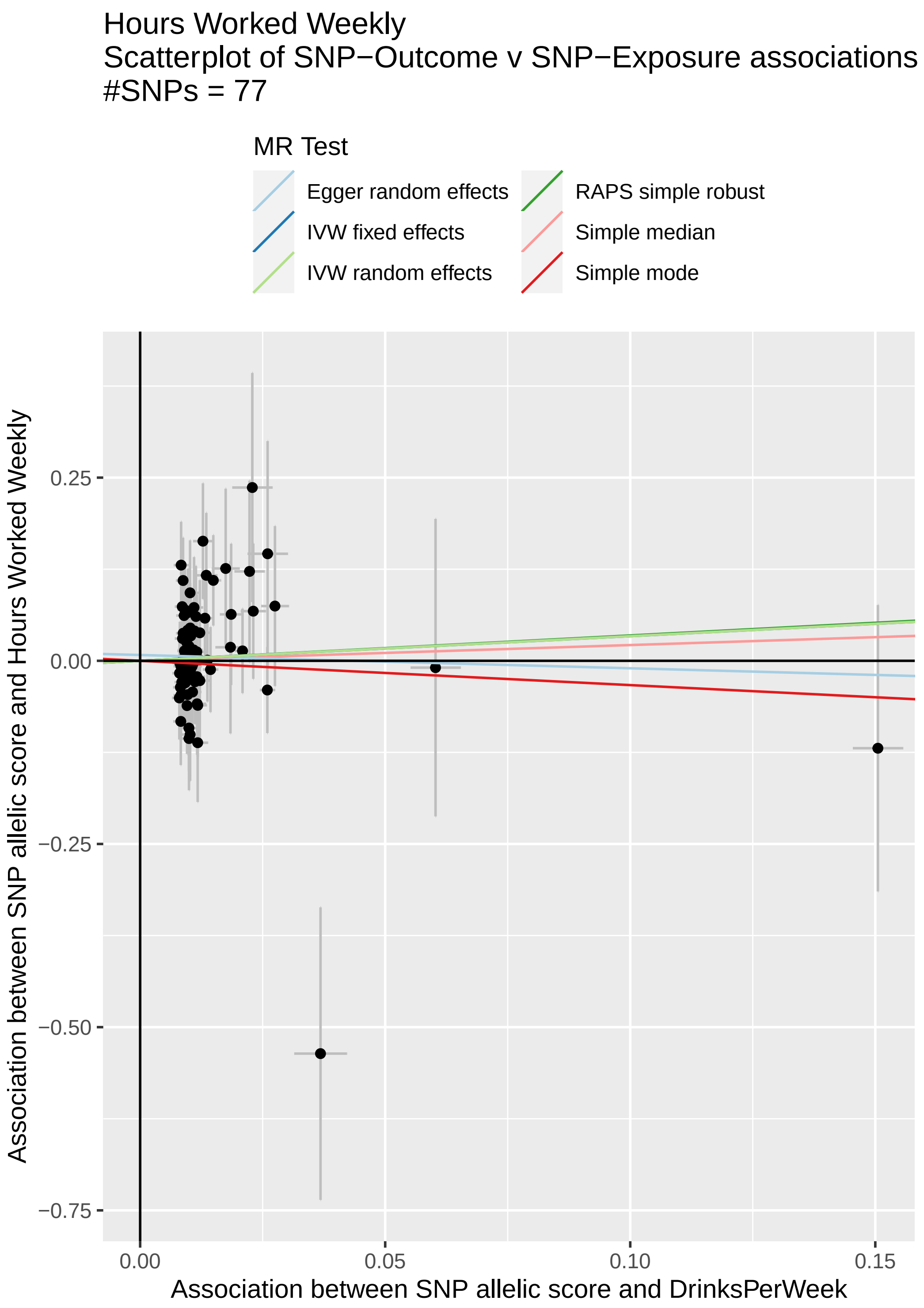
Hours Worked Weekly
Scatterplot of SNP−Outcome v SNP−Exposure associations
#SNPs = 77
MR Test
Egger random effects
IVW fixed effects
IVW random effects
RAPS simple robust
Simple median
Simple mode
0.25
0.00
−0.25
−0.50
−0.75
0.00
0.05
0.10
0.15
Association between SNP allelic score and Hours Worked Weekly
Association between SNP allelic score and DrinksPerWeek

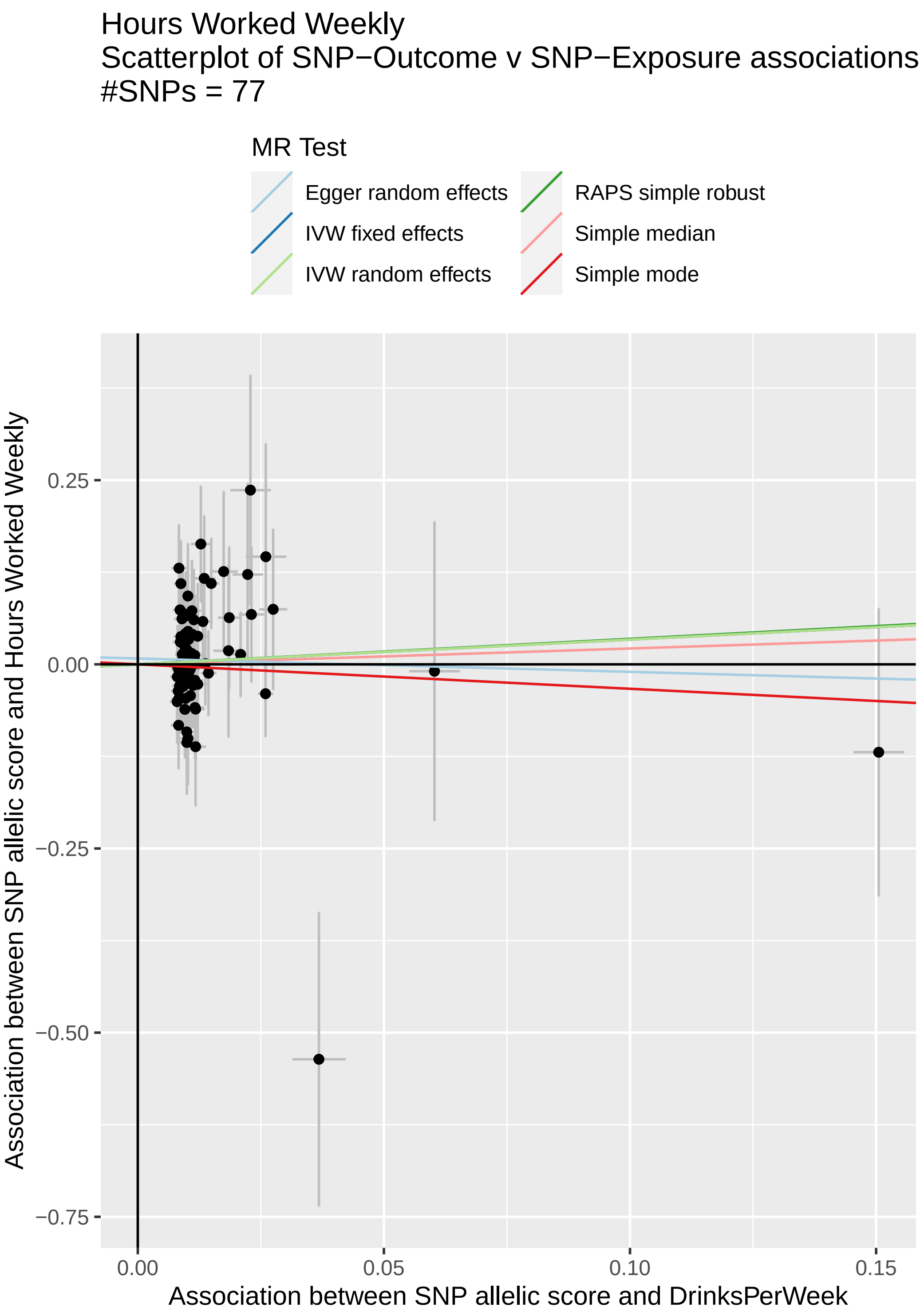
Hours Worked Weekly
Scatterplot of SNP−Outcome v SNP−Exposure associations
#SNPs = 77
MR Test
Egger random effects
IVW fixed effects
IVW random effects
RAPS simple robust
Simple median
Simple mode
0.25
0.00
−0.25
−0.50
−0.75
0.00
0.05
0.10
0.15
Association between SNP allelic score and Hours Worked Weekly
Association between SNP allelic score and DrinksPerWeek

Hours Worked Weekly
Causal Effect estimates for alcoholUnitsWeekly_wins140 on Hours Worked Weekly
#SNPs = 77, #Outlier SNPs removed = 0

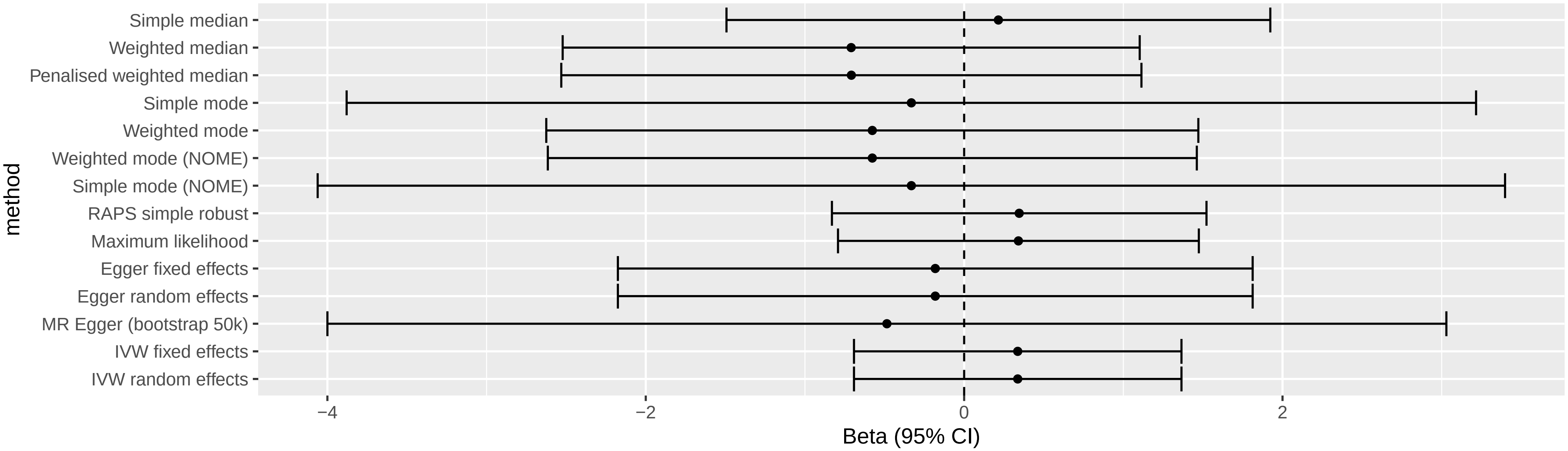

Hours Worked Weekly
Causal Effect estimates for alcoholUnitsWeekly_wins140 on Hours Worked Weekly
#SNPs = 77, #Outlier SNPs removed = 0

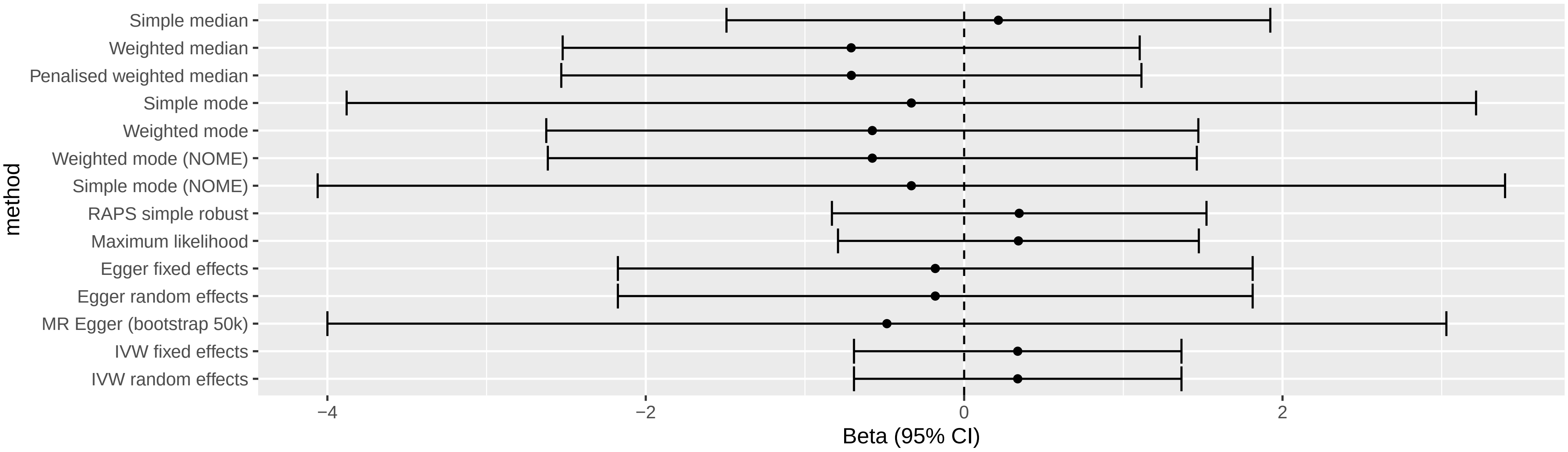

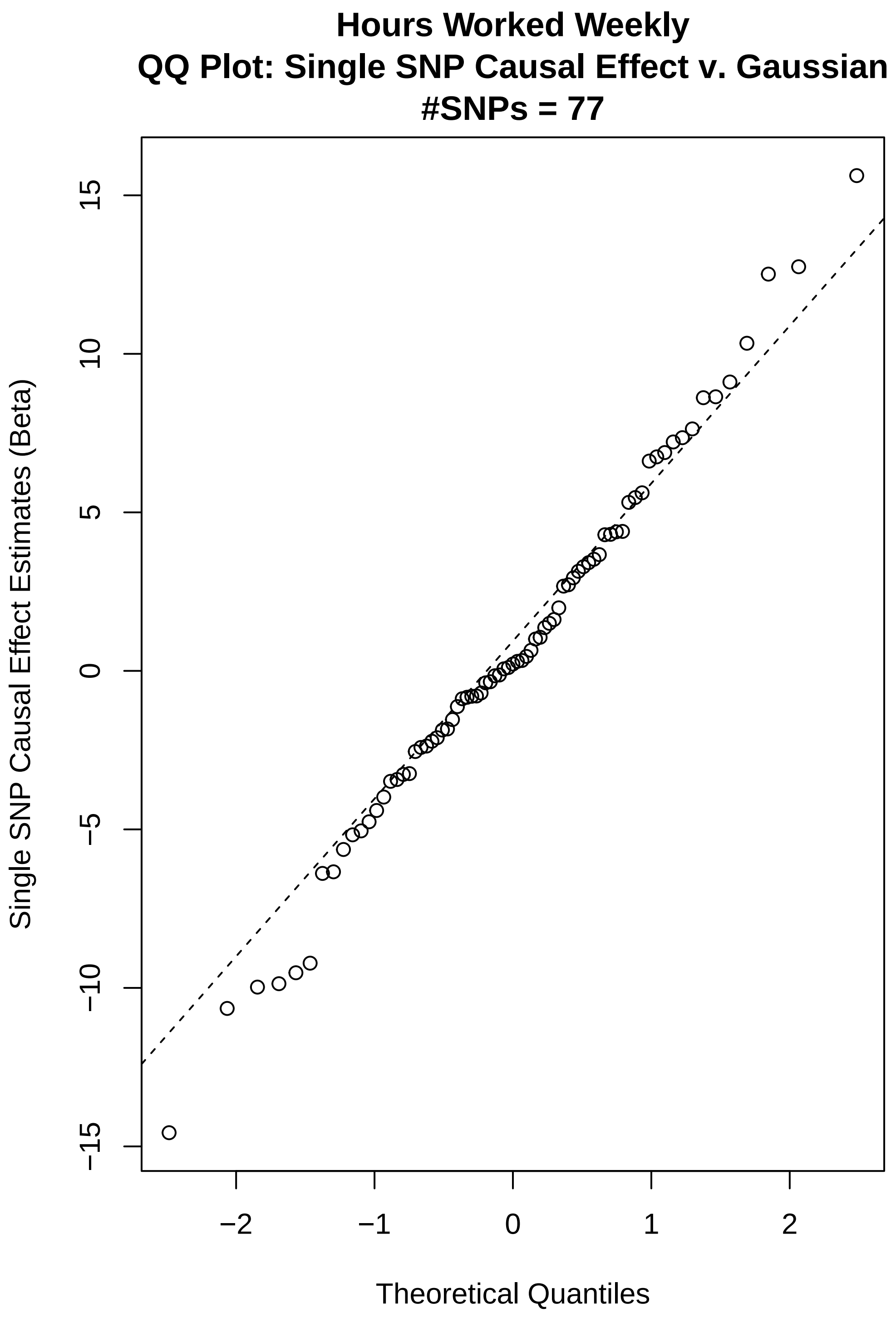
Hours Worked Weekly
QQ Plot: Single SNP Causal Effect v. Gaussian
#SNPs = 77
Single SNP Causal Effect Estimates (Beta)
Theoretical Quantiles
−15
−10
−5
0
5
10
15
−2
−1
0
1
2

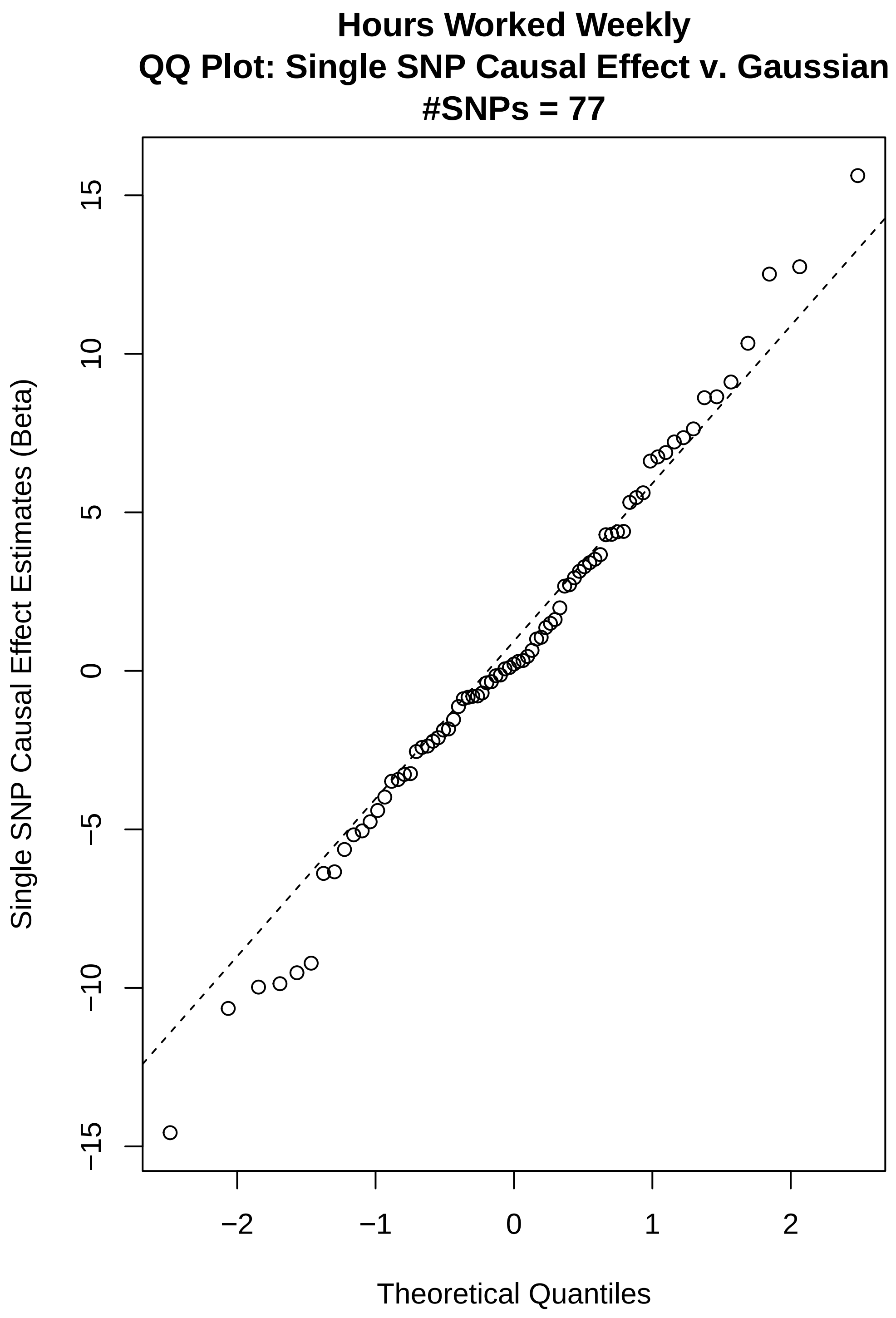
Hours Worked Weekly
QQ Plot: Single SNP Causal Effect v. Gaussian
#SNPs = 77
Single SNP Causal Effect Estimates (Beta)
Theoretical Quantiles
−15
−10
−5
0
5
10
15
−2
−1
0
1
2

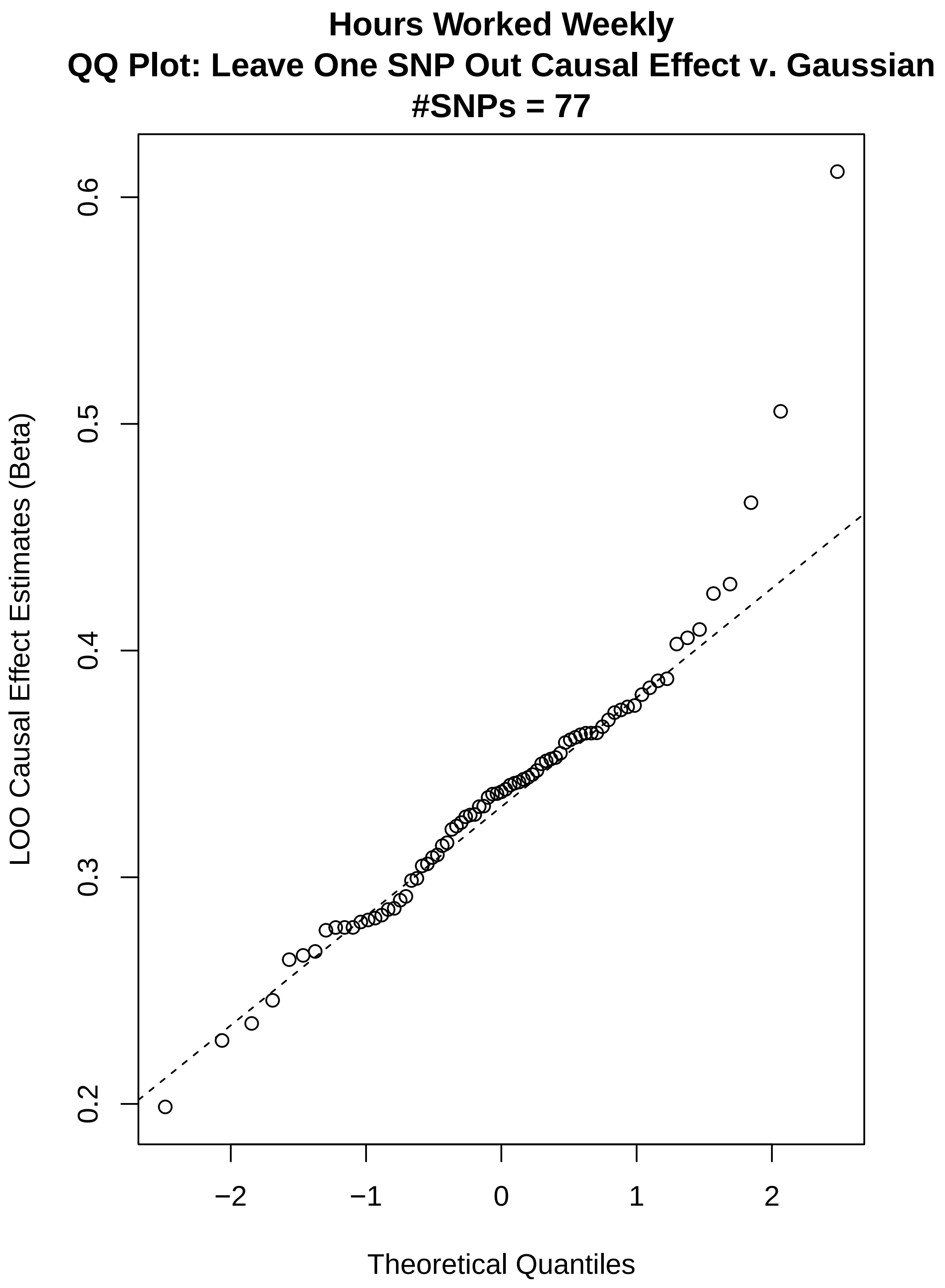
Hours Worked Weekly
QQ Plot: Leave One SNP Out Causal Effect v. Gaussian
#SNPs = 77
LOO Causal Effect Estimates (Beta)
0.2
0.3
0.4
0.5
0.6
−2
−1
0
1
2
Theoretical Quantiles

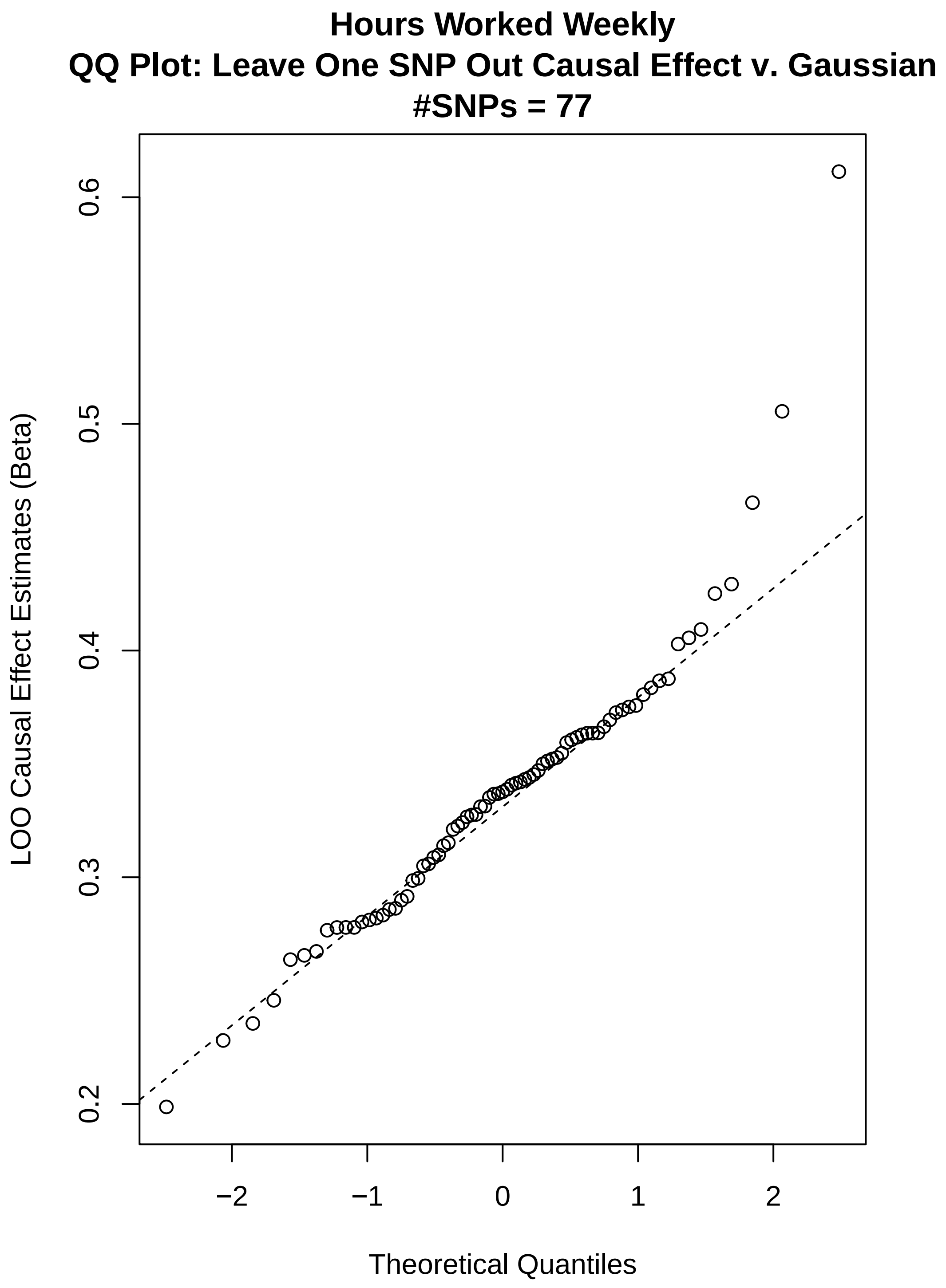
Hours Worked Weekly
QQ Plot: Leave One SNP Out Causal Effect v. Gaussian
#SNPs = 77
LOO Causal Effect Estimates (Beta)
0.2
0.3
0.4
0.5
0.6
−2
−1
0
1
2
Theoretical Quantiles

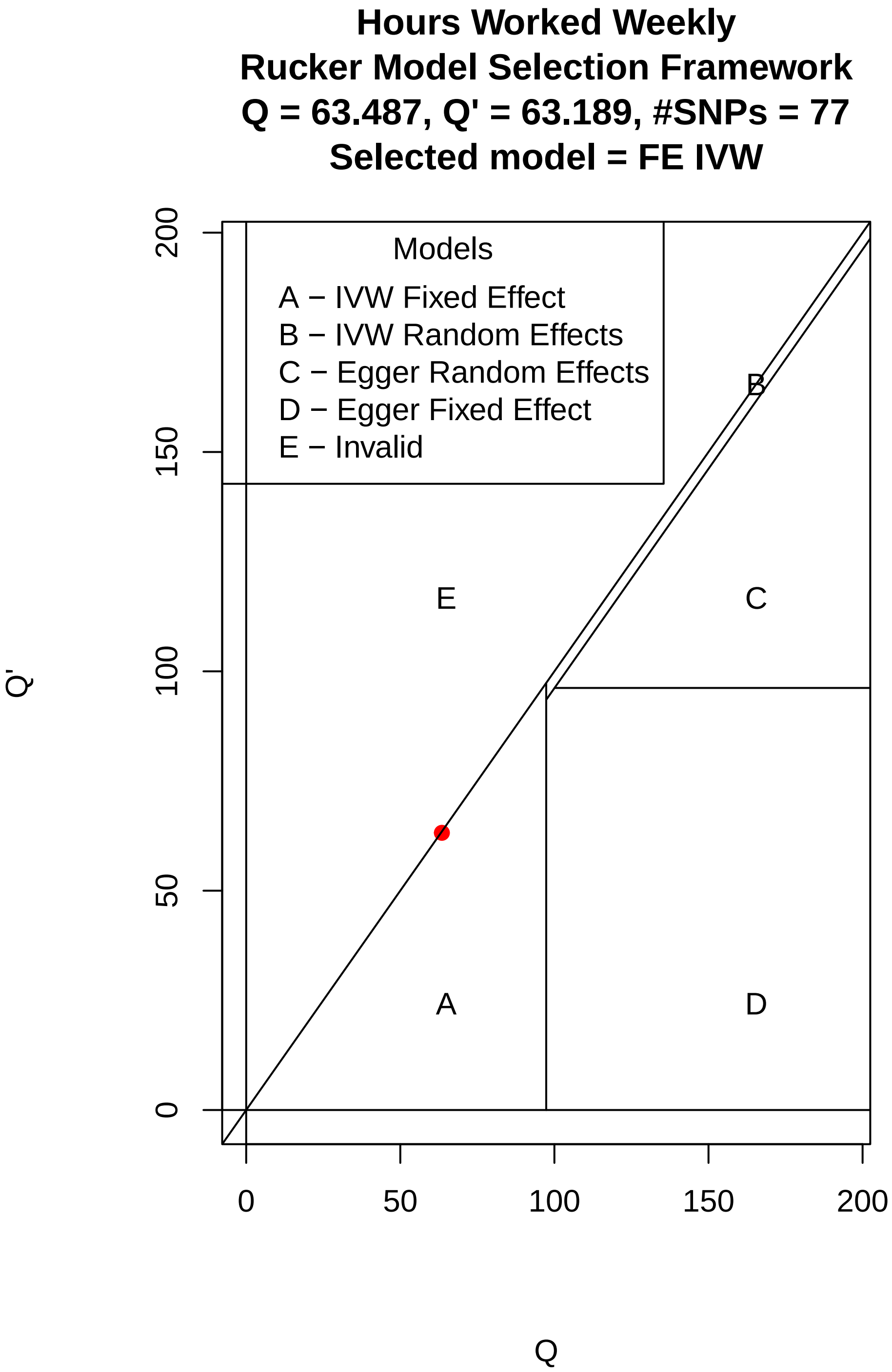
Hours Worked Weekly
Rucker Model Selection Framework
Q = 63.487, Q' = 63.189, #SNPs = 77
Selected model = FE IVW
Models
A − IVW Fixed Effect
B − IVW Random Effects
C − Egger Random Effects
D − Egger Fixed Effect
E − Invalid
B
C
E
A
D
Q'
Q
0
50
100
150
200

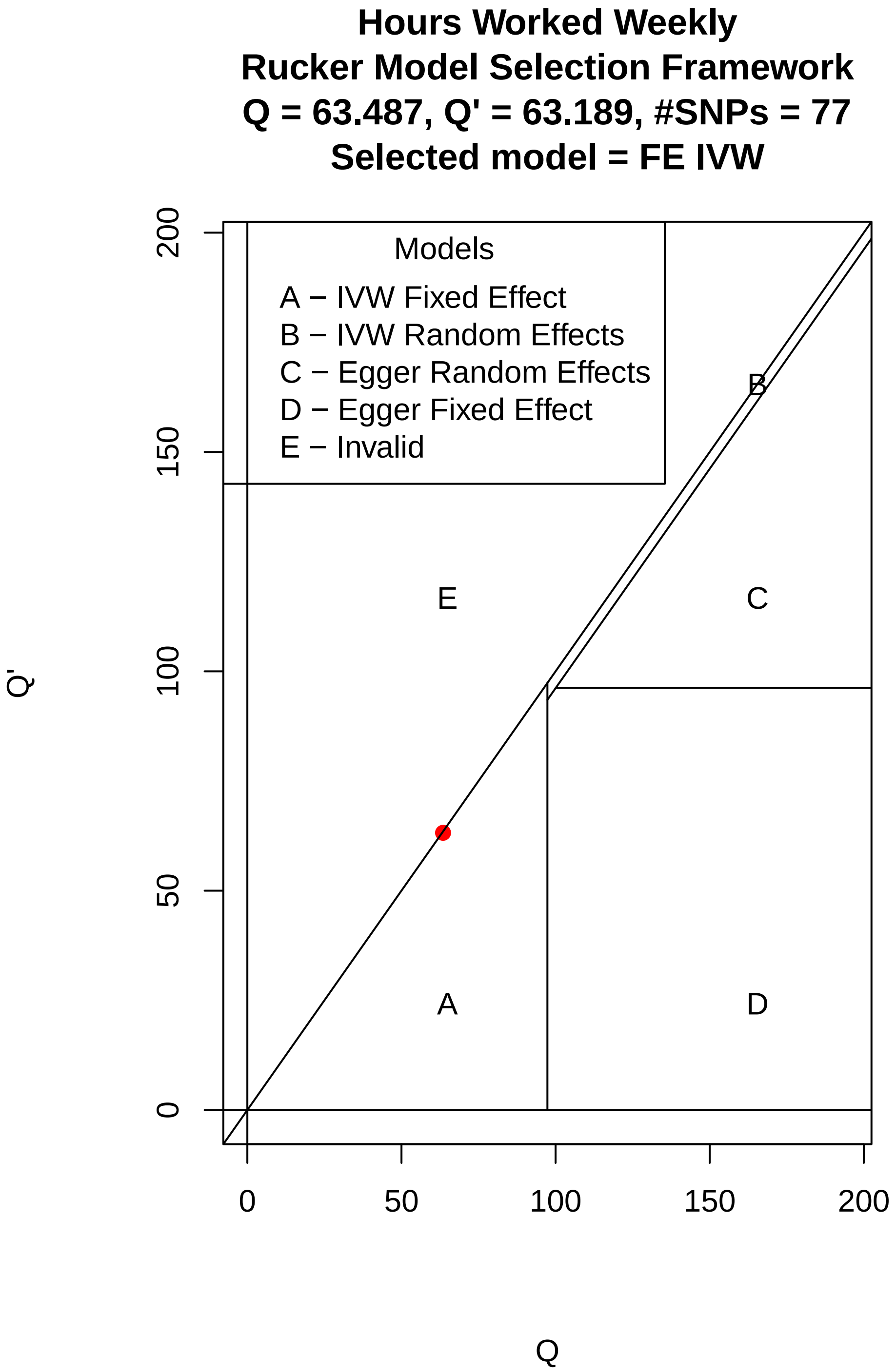
Hours Worked Weekly
Rucker Model Selection Framework
Q = 63.487, Q' = 63.189, #SNPs = 77
Selected model = FE IVW
Models
A − IVW Fixed Effect
B − IVW Random Effects
C − Egger Random Effects
D − Egger Fixed Effect
E − Invalid
B
C
E
A
D
Q'
Q
0
50
100
150
200

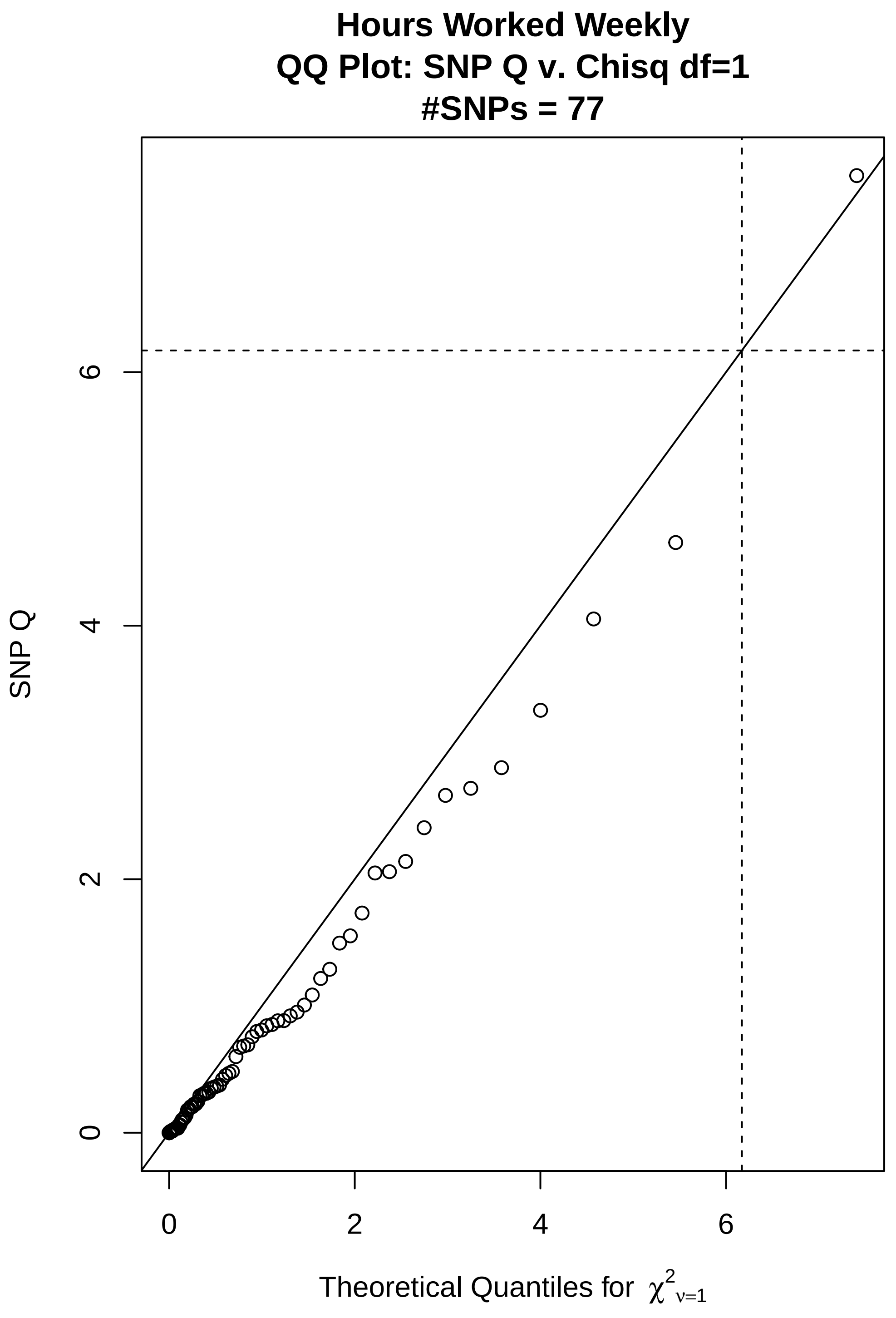
Hours Worked Weekly
QQ Plot: SNP Q v. Chisq df=1
#SNPs = 77
SNP Q
0
2
4
6
Theoretical Quantiles for $\chi^2_{\nu=1}$

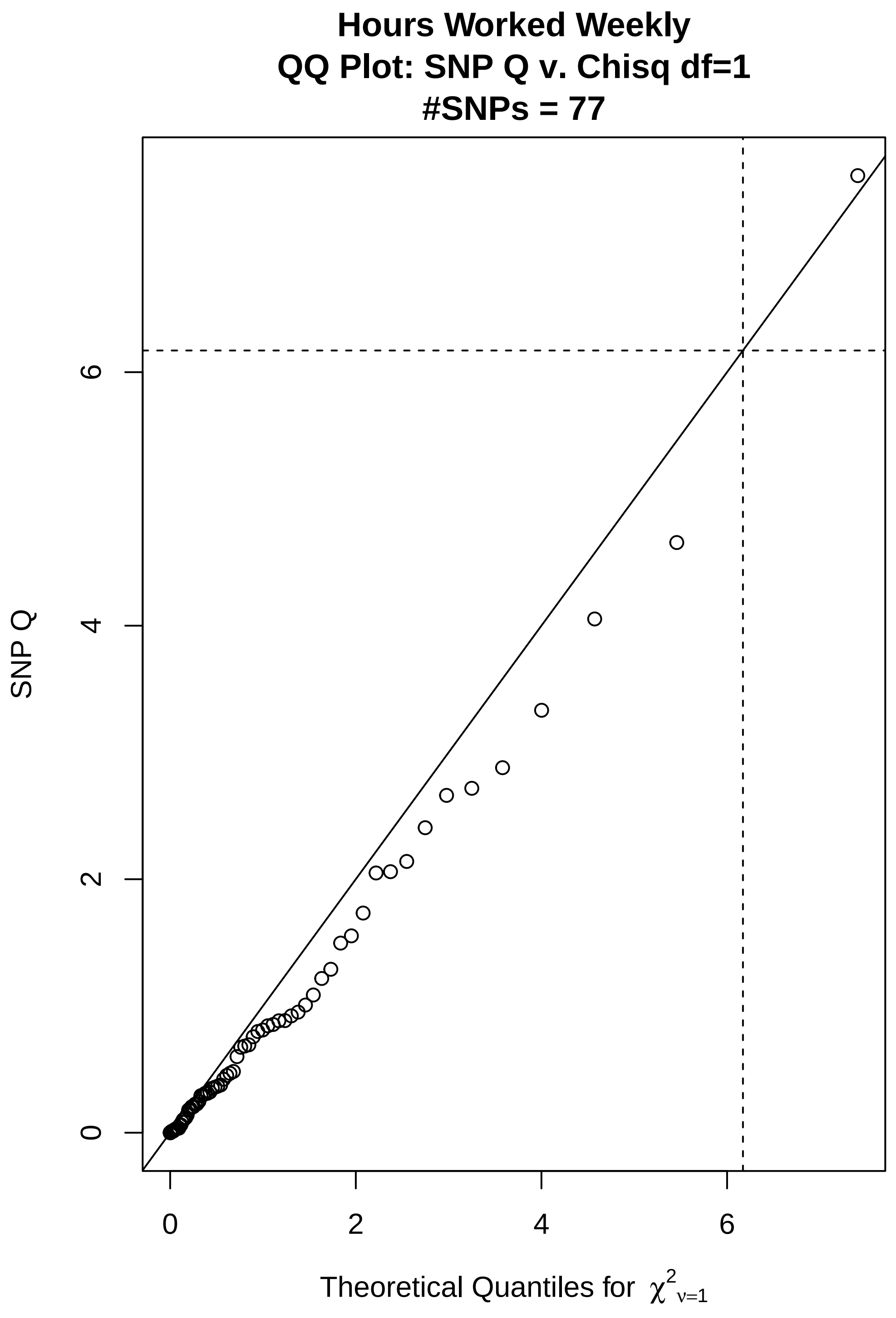
Hours Worked Weekly
QQ Plot: SNP Q v. Chisq df=1
#SNPs = 77
SNP Q
0
2
4
6
Theoretical Quantiles for $\chi^2_{\nu=1}$
