# Supplementary material for: Effects of alcohol consumption on employment and social outcomes: a Mendelian randomisation study
Source: Alcohol Alcohol. 2025 Jul 18;60(5):agaf038. doi: 10.1093/alcalc/agaf038 (PMC12271571; doi:10.1093/alcalc/agaf038)
Supplement: Campbell_Green_Davies_et_al_2025_agaf038 [file campbell_green_davies_et_al_2025_agaf038.zip › Campbell_Green_Davies_et_al_2025/Male/aud/do2SampleMrAnalyses_bAlcoholUseDisorder_tdiDecile_ageCentreGpc.pdf]

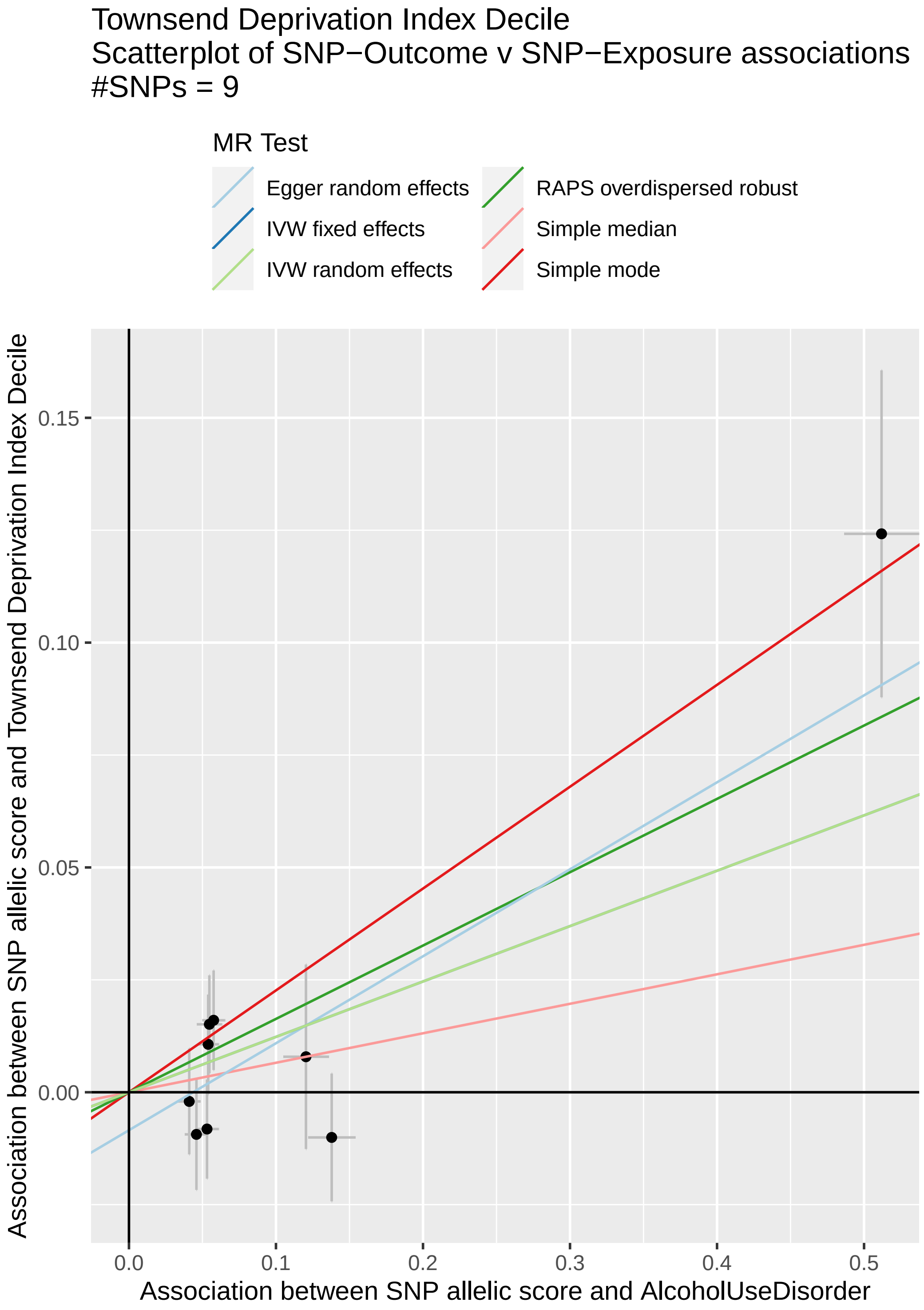

Townsend Deprivation Index Decile
Scatterplot of SNP−Outcome v SNP−Exposure associations
#SNPs = 9
MR Test
Egger random effects
IVW fixed effects
IVW random effects
RAPS overdispersed robust
Simple median
Simple mode
Association between SNP allelic score and Townsend Deprivation Index Decile
0.15
0.10
0.05
0.00
0.0
0.1
0.2
0.3
0.4
0.5
Association between SNP allelic score and AlcoholUseDisorder

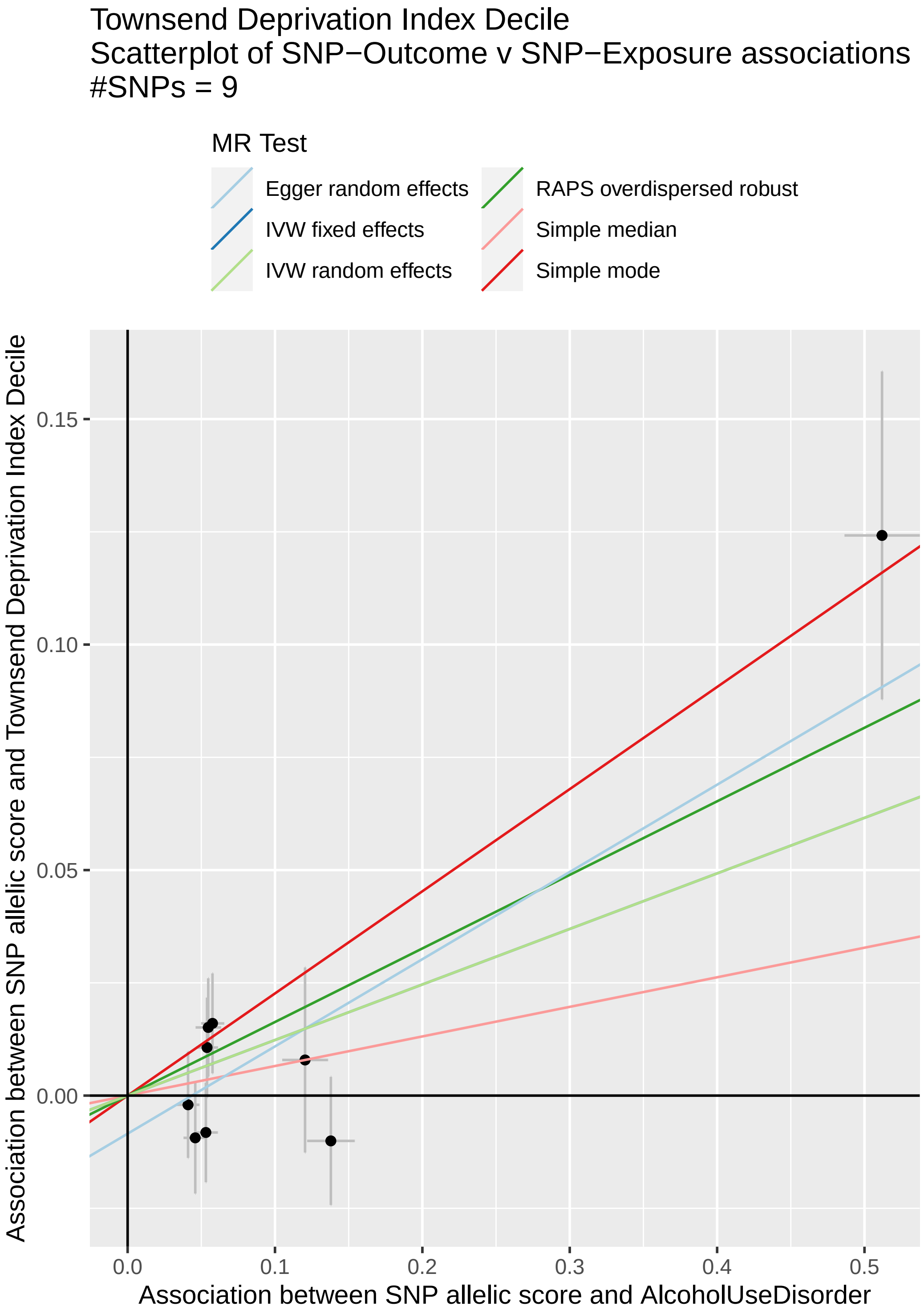

Townsend Deprivation Index Decile
Scatterplot of SNP−Outcome v SNP−Exposure associations
#SNPs = 9
MR Test
Egger random effects
IVW fixed effects
IVW random effects
RAPS overdispersed robust
Simple median
Simple mode
Association between SNP allelic score and Townsend Deprivation Index Decile
0.15
0.10
0.05
0.00
0.0
0.1
0.2
0.3
0.4
0.5
Association between SNP allelic score and AlcoholUseDisorder

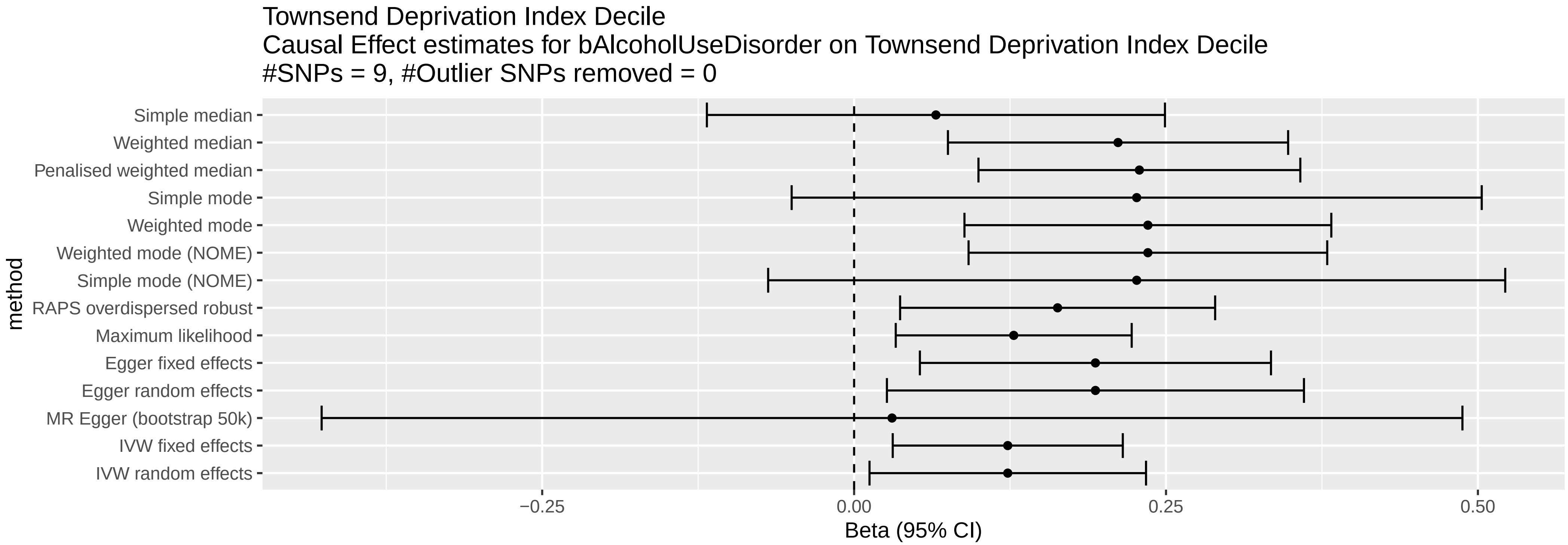

Townsend Deprivation Index Decile
Causal Effect estimates for bAlcoholUseDisorder on Townsend Deprivation Index Decile
#SNPs = 9, #Outlier SNPs removed = 0
method
Simple median
Weighted median
Penalised weighted median
Simple mode
Weighted mode
Weighted mode (NOME)
Simple mode (NOME)
RAPS overdispersed robust
Maximum likelihood
Egger fixed effects
Egger random effects
MR Egger (bootstrap 50k)
IVW fixed effects
IVW random effects
−0.25
0.00
0.25
0.50
Beta (95% CI)

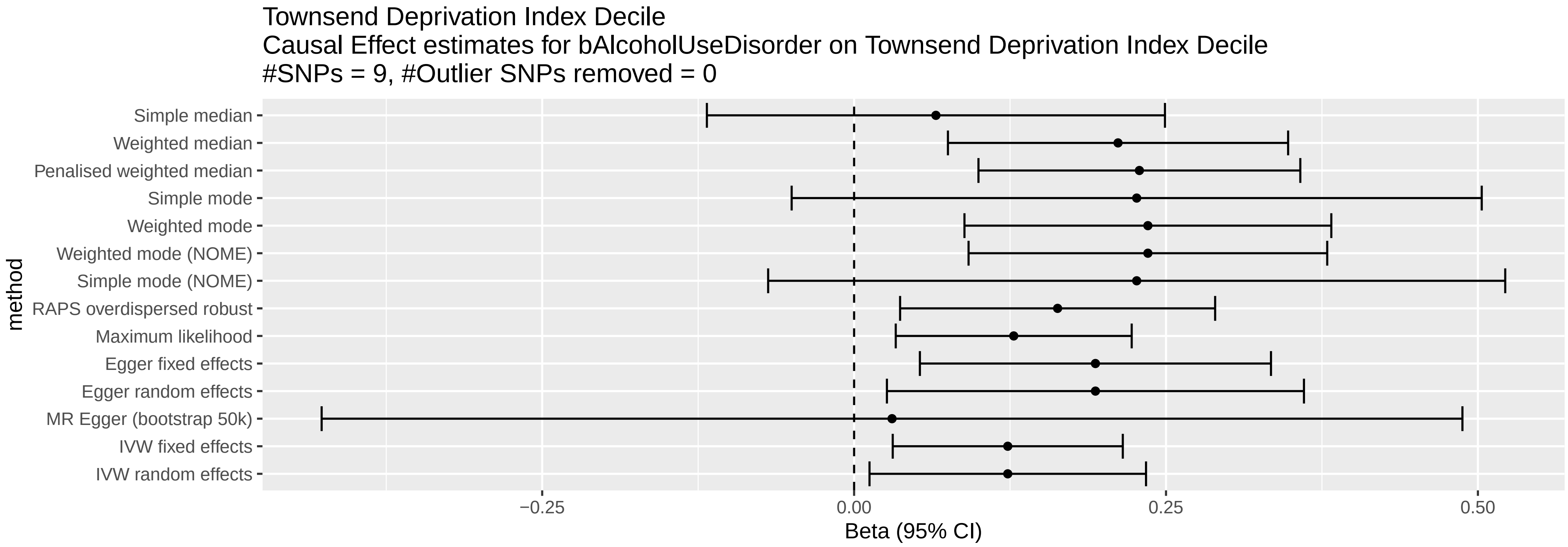

Townsend Deprivation Index Decile
Causal Effect estimates for bAlcoholUseDisorder on Townsend Deprivation Index Decile
#SNPs = 9, #Outlier SNPs removed = 0
method
Simple median
Weighted median
Penalised weighted median
Simple mode
Weighted mode
Weighted mode (NOME)
Simple mode (NOME)
RAPS overdispersed robust
Maximum likelihood
Egger fixed effects
Egger random effects
MR Egger (bootstrap 50k)
IVW fixed effects
IVW random effects
−0.25
0.00
0.25
0.50
Beta (95% CI)

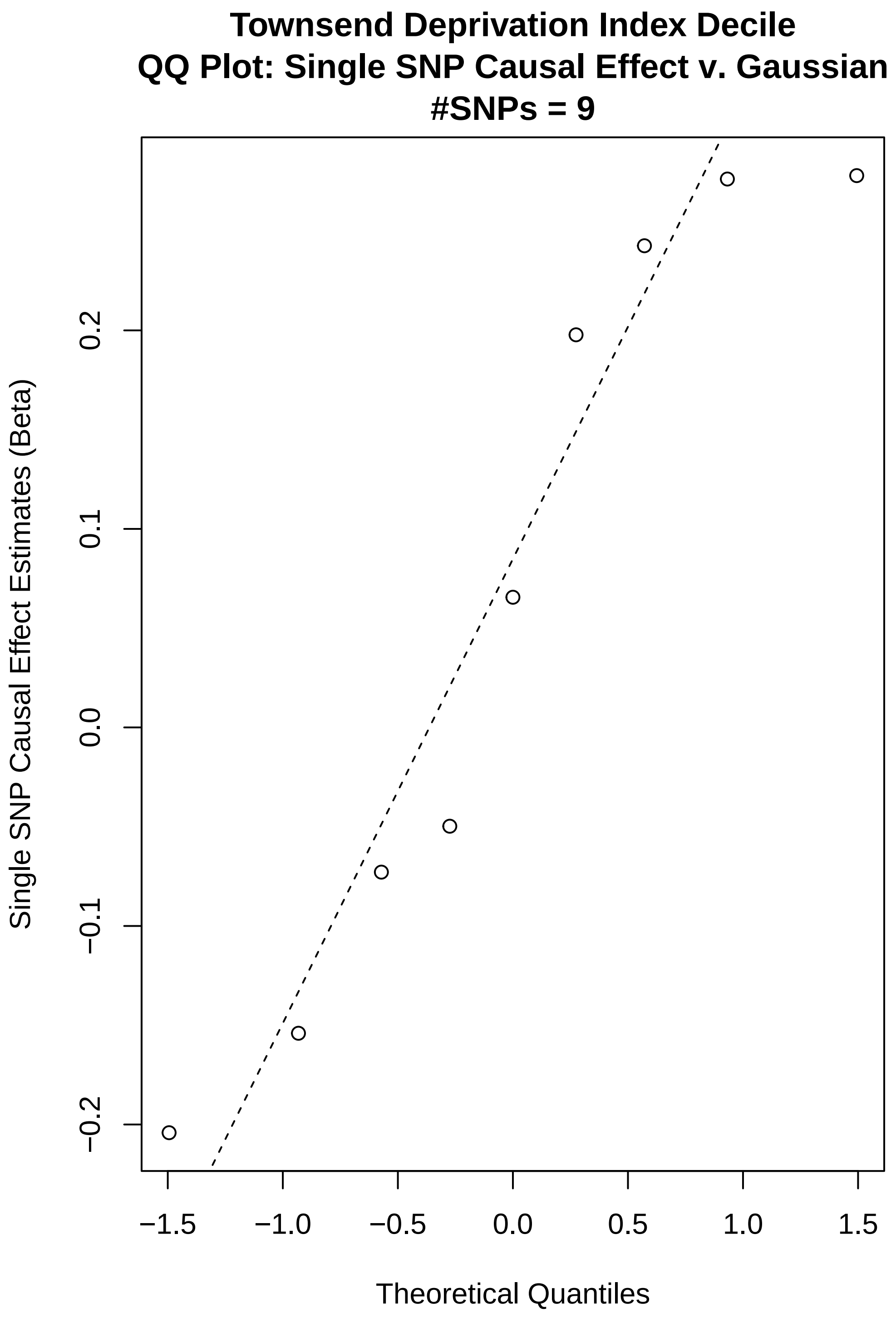
Townsend Deprivation Index Decile
QQ Plot: Single SNP Causal Effect v. Gaussian
#SNPs = 9
Single SNP Causal Effect Estimates (Beta)
Theoretical Quantiles
−0.2
−0.1
0.0
0.1
0.2
−1.5
−1.0
−0.5
0.0
0.5
1.0
1.5

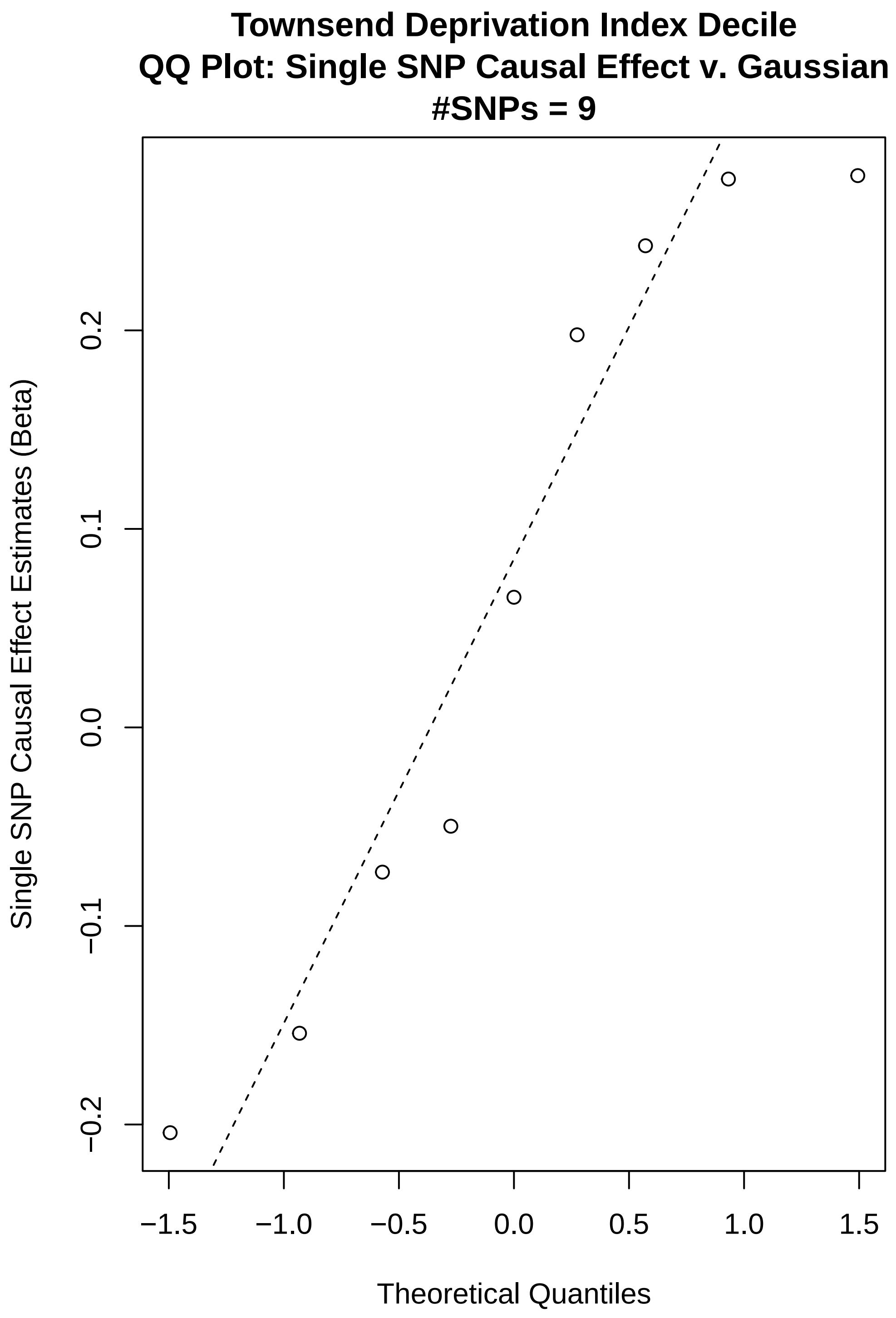
Townsend Deprivation Index Decile
QQ Plot: Single SNP Causal Effect v. Gaussian
#SNPs = 9
Single SNP Causal Effect Estimates (Beta)
Theoretical Quantiles
−0.2
−0.1
0.0
0.1
0.2
−1.5
−1.0
−0.5
0.0
0.5
1.0
1.5

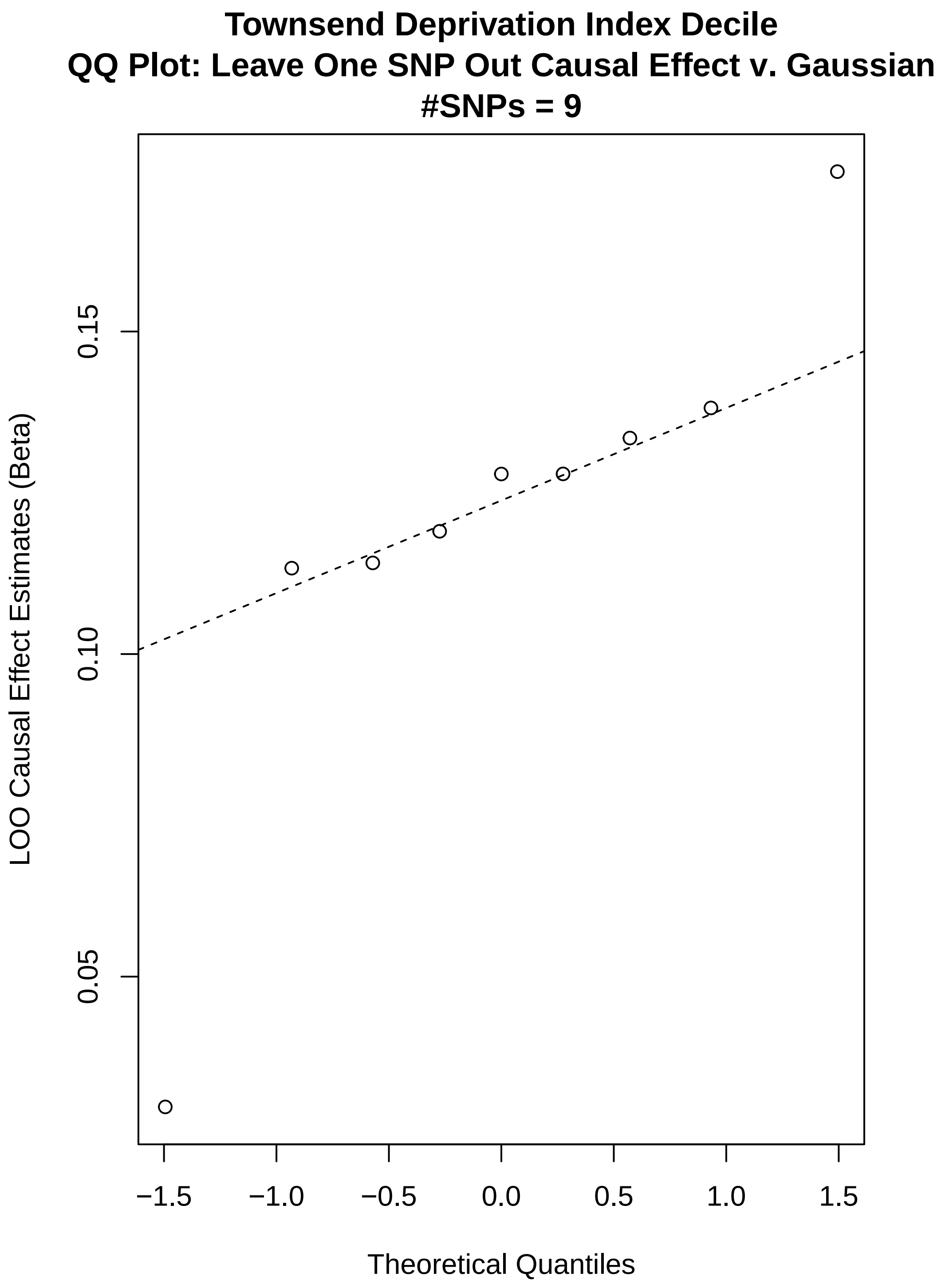
Townsend Deprivation Index Decile
QQ Plot: Leave One SNP Out Causal Effect v. Gaussian
#SNPs = 9
LOO Causal Effect Estimates (Beta)
0.05
0.10
0.15
−1.5
−1.0
−0.5
0.0
0.5
1.0
1.5
Theoretical Quantiles

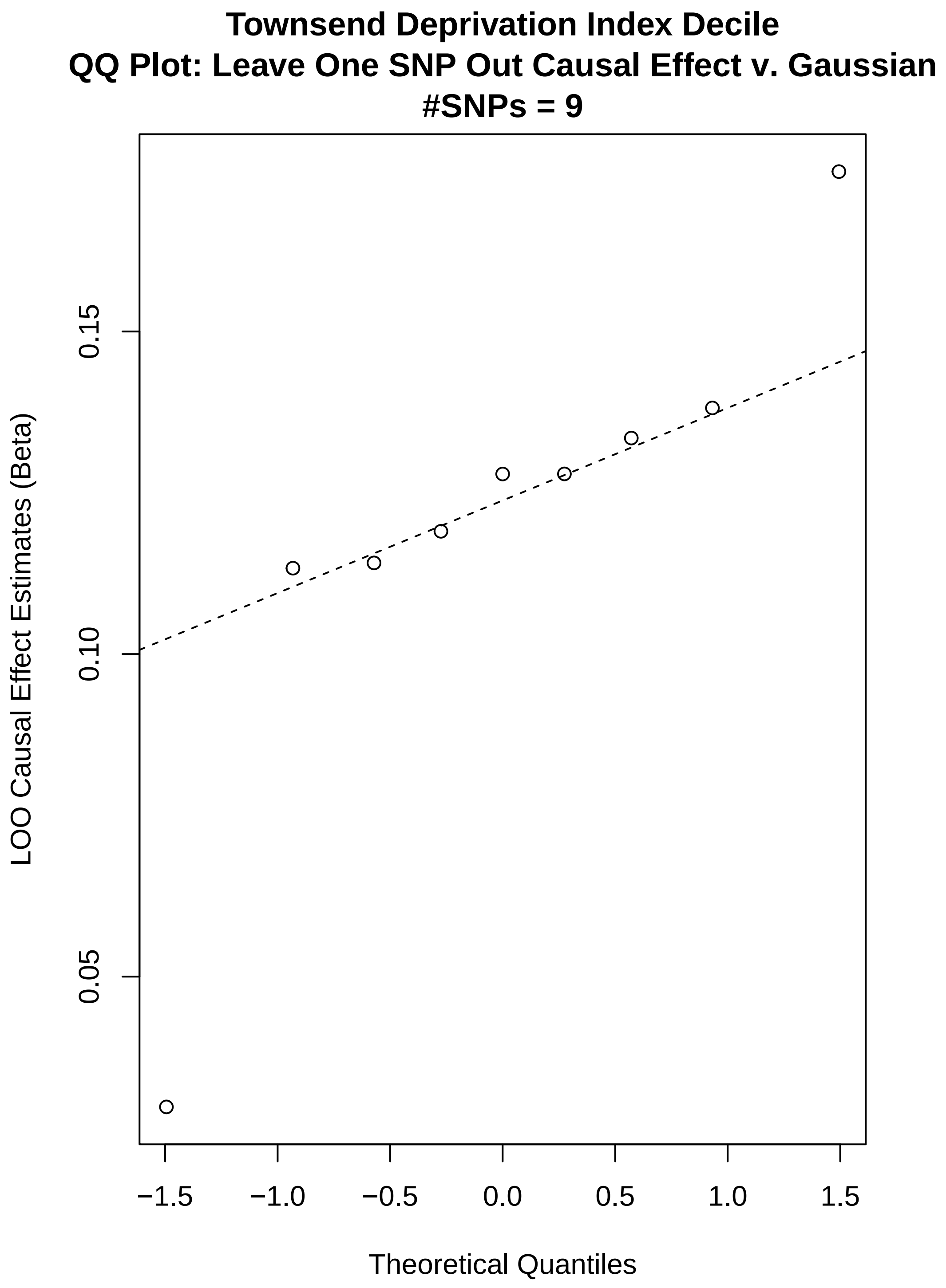
Townsend Deprivation Index Decile
QQ Plot: Leave One SNP Out Causal Effect v. Gaussian
#SNPs = 9
LOO Causal Effect Estimates (Beta)
0.05
0.10
0.15
−1.5
−1.0
−0.5
0.0
0.5
1.0
1.5
Theoretical Quantiles

Townsend Deprivation Index Decile
Rucker Model Selection Framework
Q = 11.555, Q' = 9.8756, #SNPs = 9
Selected model = FE IVW

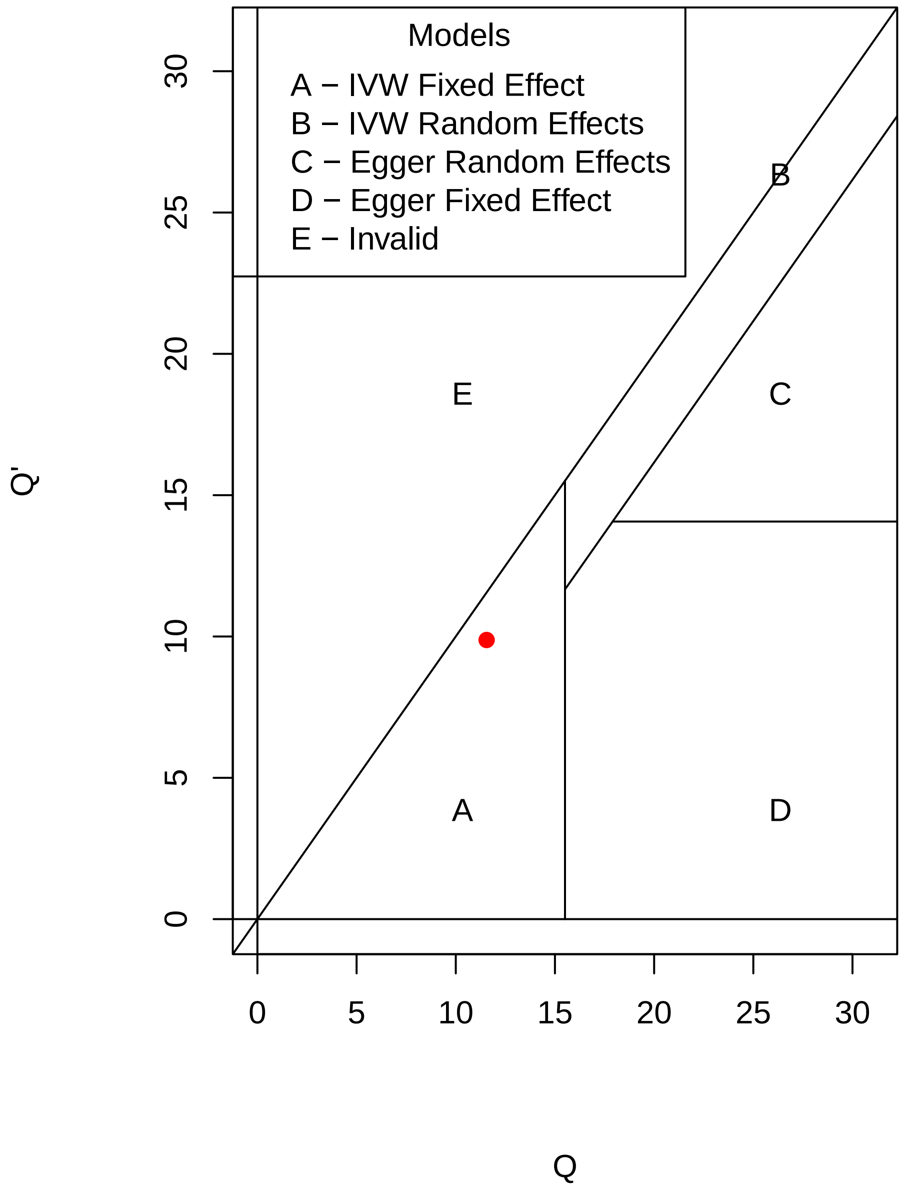

Townsend Deprivation Index Decile
Rucker Model Selection Framework
Q = 11.555, Q' = 9.8756, #SNPs = 9
Selected model = FE IVW

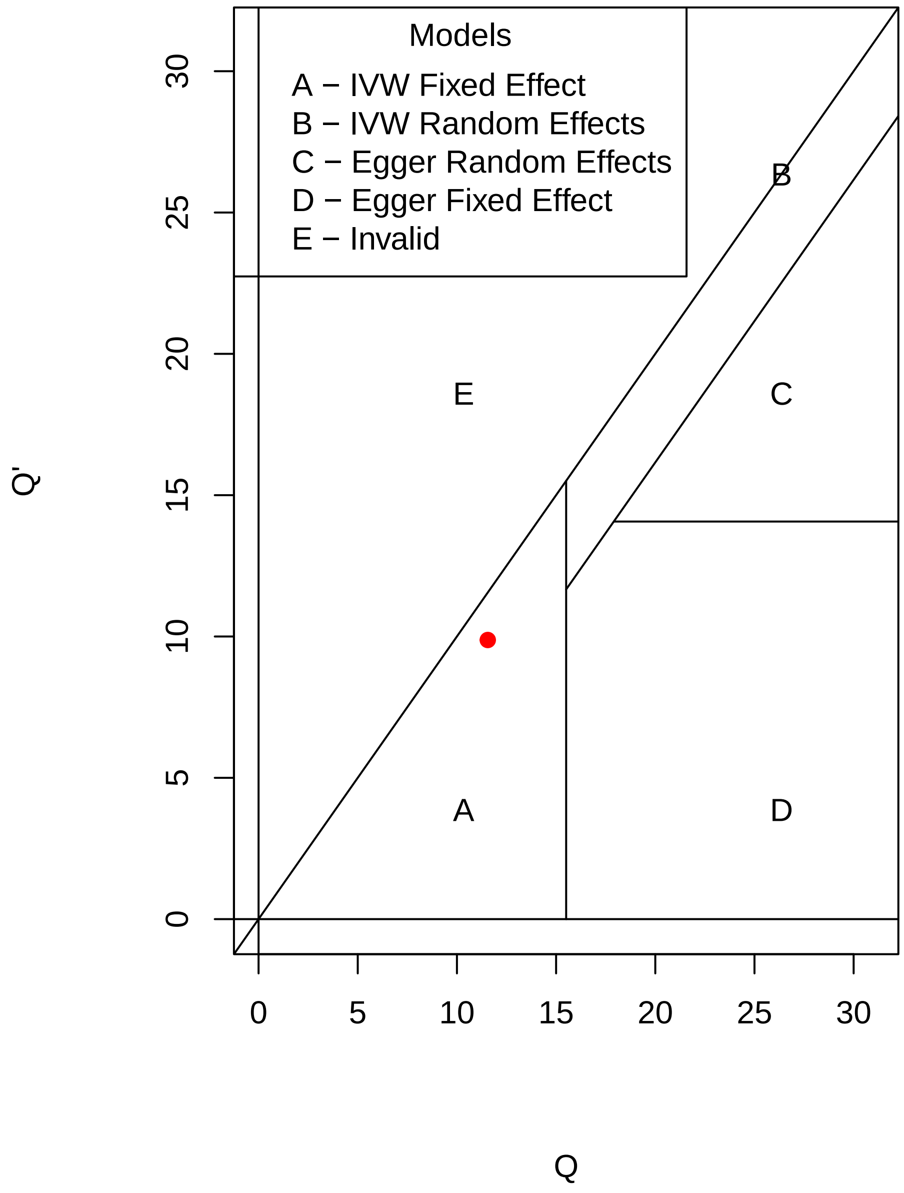

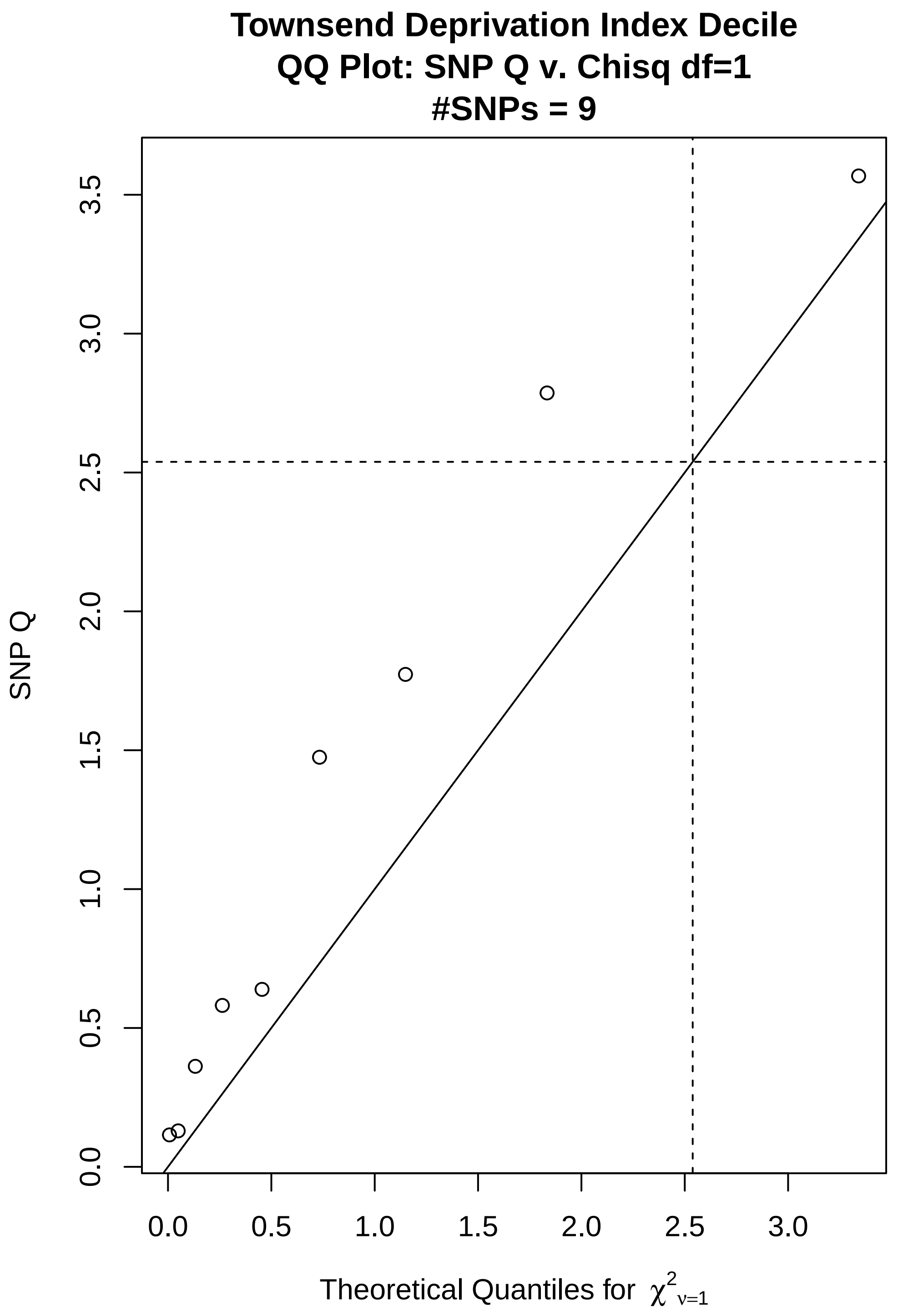
Townsend Deprivation Index Decile
QQ Plot: SNP Q v. Chisq df=1
#SNPs = 9
SNP Q
0.0
0.5
1.0
1.5
2.0
2.5
3.0
3.5
0.0
0.5
1.0
1.5
2.0
2.5
3.0
Theoretical Quantiles for $\chi^2_{\nu=1}$

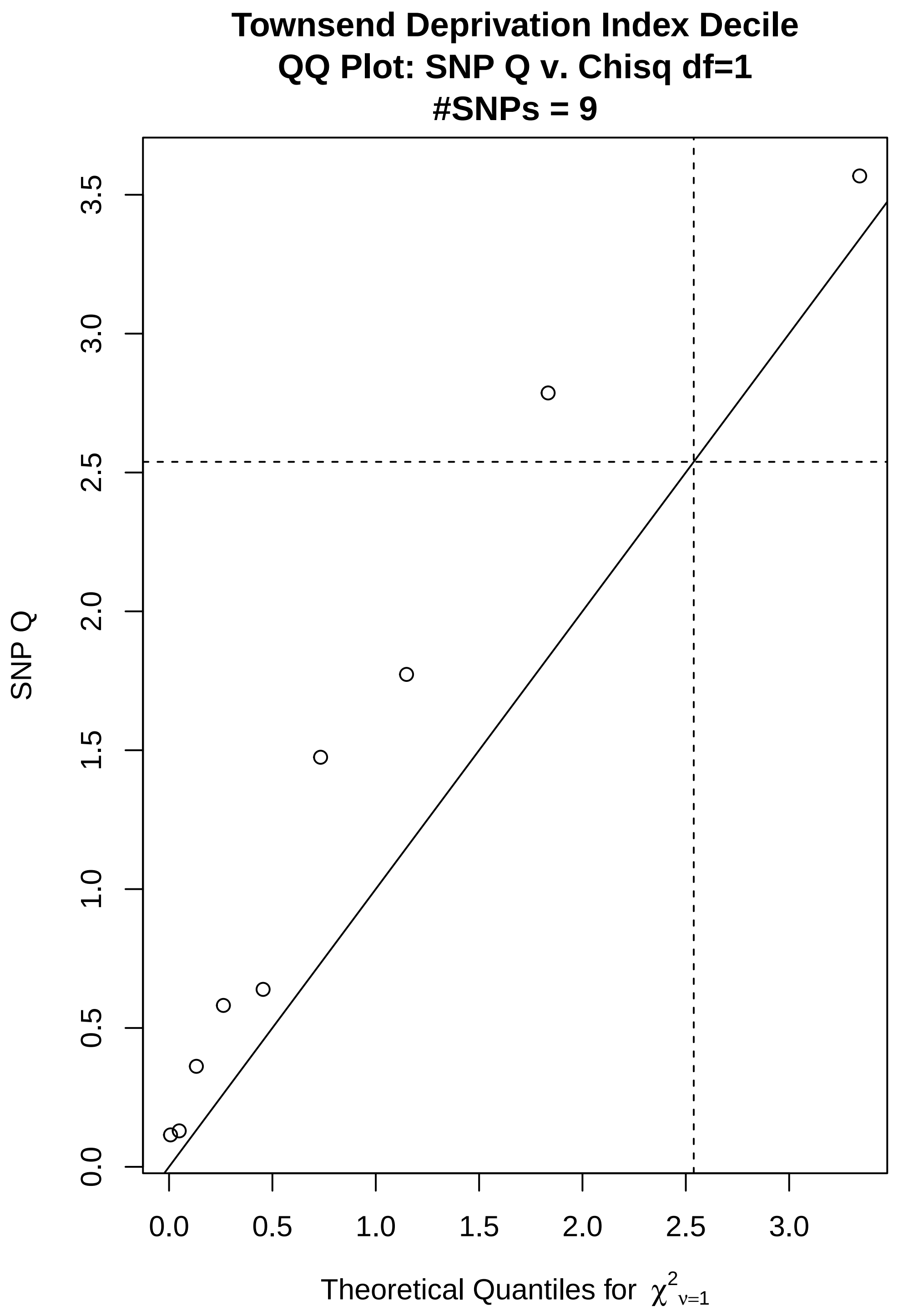
Townsend Deprivation Index Decile
QQ Plot: SNP Q v. Chisq df=1
#SNPs = 9
SNP Q
0.0
0.5
1.0
1.5
2.0
2.5
3.0
3.5
0.0
0.5
1.0
1.5
2.0
2.5
3.0
Theoretical Quantiles for $\chi^2_{\nu=1}$
