# Supplementary material for: Effects of alcohol consumption on employment and social outcomes: a Mendelian randomisation study
Source: Alcohol Alcohol. 2025 Jul 18;60(5):agaf038. doi: 10.1093/alcalc/agaf038 (PMC12271571; doi:10.1093/alcalc/agaf038)
Supplement: Campbell_Green_Davies_et_al_2025_agaf038 [file campbell_green_davies_et_al_2025_agaf038.zip › Campbell_Green_Davies_et_al_2025/Male/drink/do2SampleMrAnalyses_alcoholUnitsWeekly_wins140_tdi_ageCentreGpc.pdf]

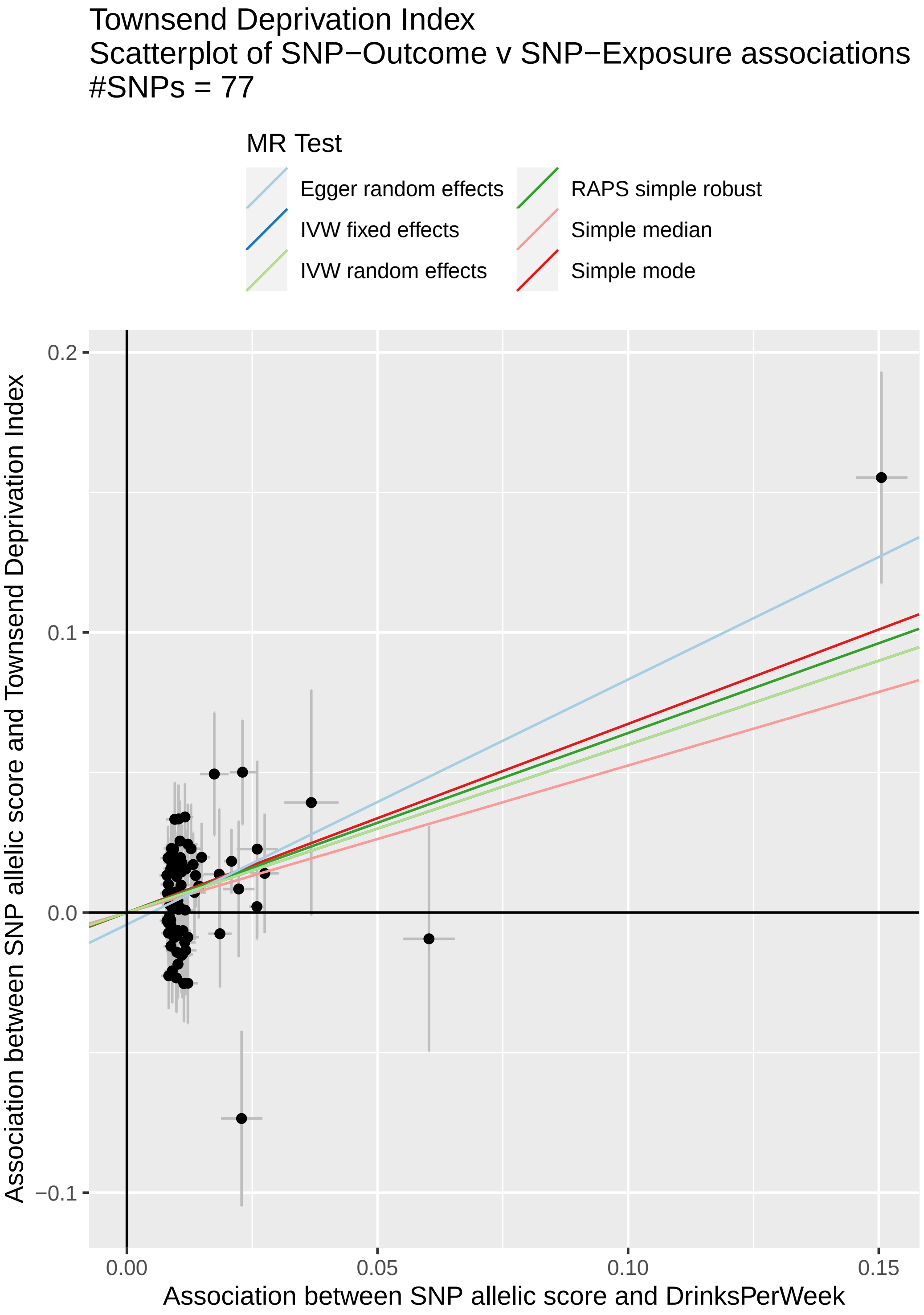
Townsend Deprivation Index
Scatterplot of SNP−Outcome v SNP−Exposure associations
#SNPs = 77
MR Test
Egger random effects
IVW fixed effects
IVW random effects
RAPS simple robust
Simple median
Simple mode
Association between SNP allelic score and Townsend Deprivation Index
Association between SNP allelic score and DrinksPerWeek
0.2
0.1
0.0
−0.1
0.00
0.05
0.10
0.15

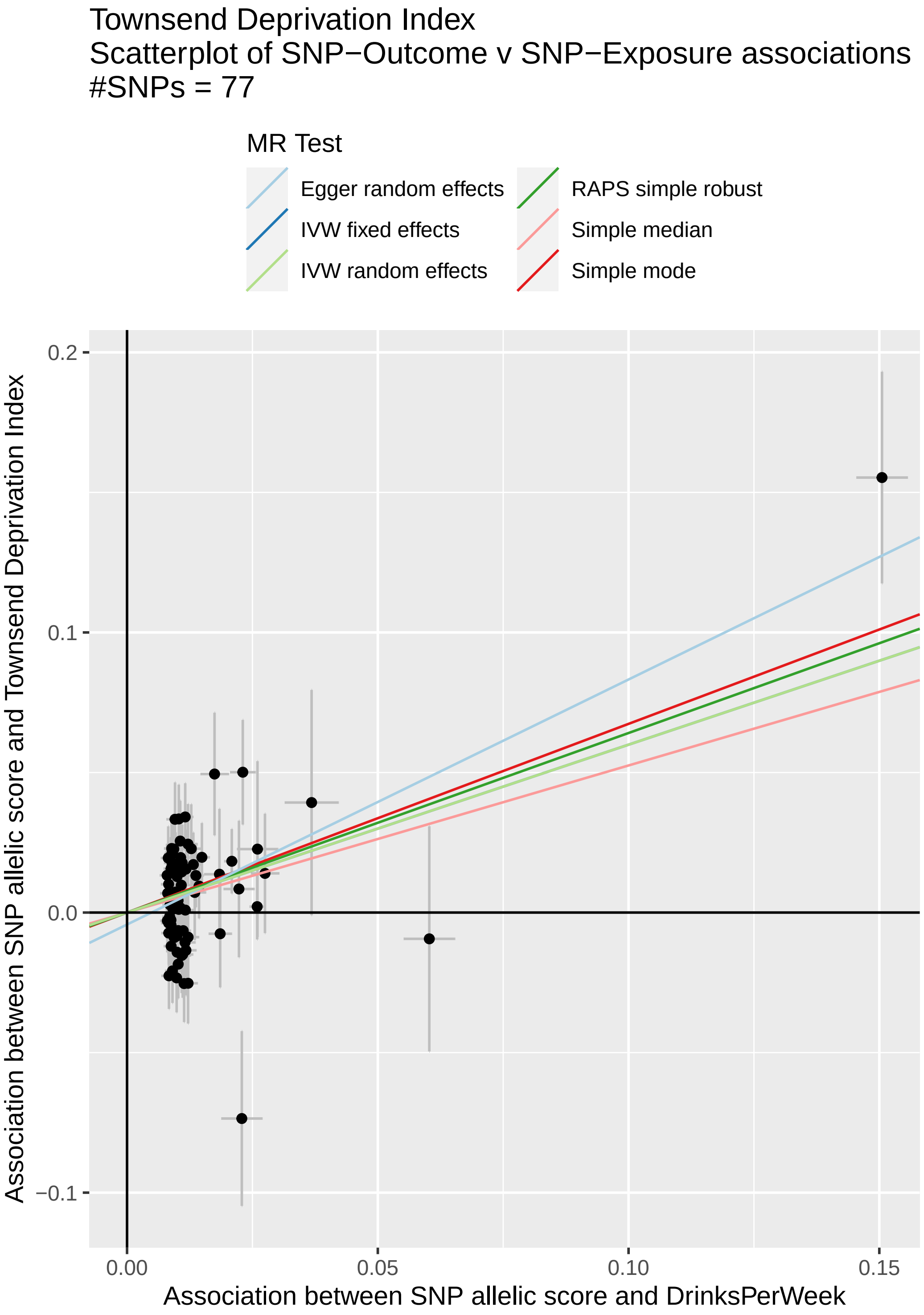
Townsend Deprivation Index
Scatterplot of SNP−Outcome v SNP−Exposure associations
#SNPs = 77
MR Test
Egger random effects
IVW fixed effects
IVW random effects
RAPS simple robust
Simple median
Simple mode
Association between SNP allelic score and Townsend Deprivation Index
Association between SNP allelic score and DrinksPerWeek
0.2
0.1
0.0
−0.1
0.00
0.05
0.10
0.15

Townsend Deprivation Index
Causal Effect estimates for alcoholUnitsWeekly_wins140 on Townsend Deprivation Index
#SNPs = 77, #Outlier SNPs removed = 0

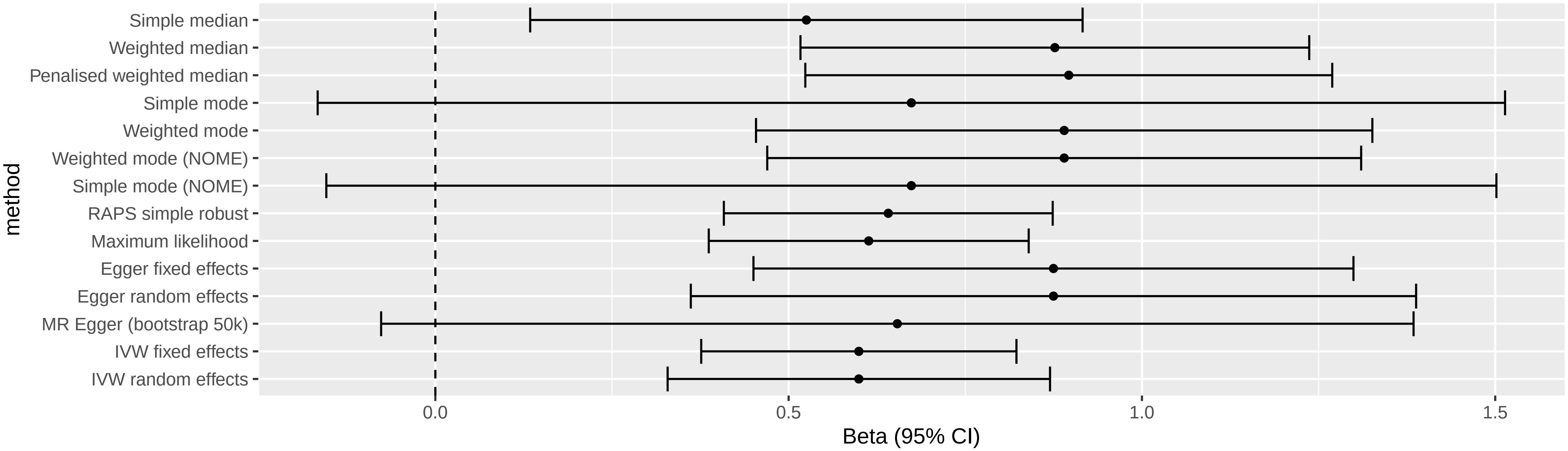

Townsend Deprivation Index
Causal Effect estimates for alcoholUnitsWeekly_wins140 on Townsend Deprivation Index
#SNPs = 77, #Outlier SNPs removed = 0

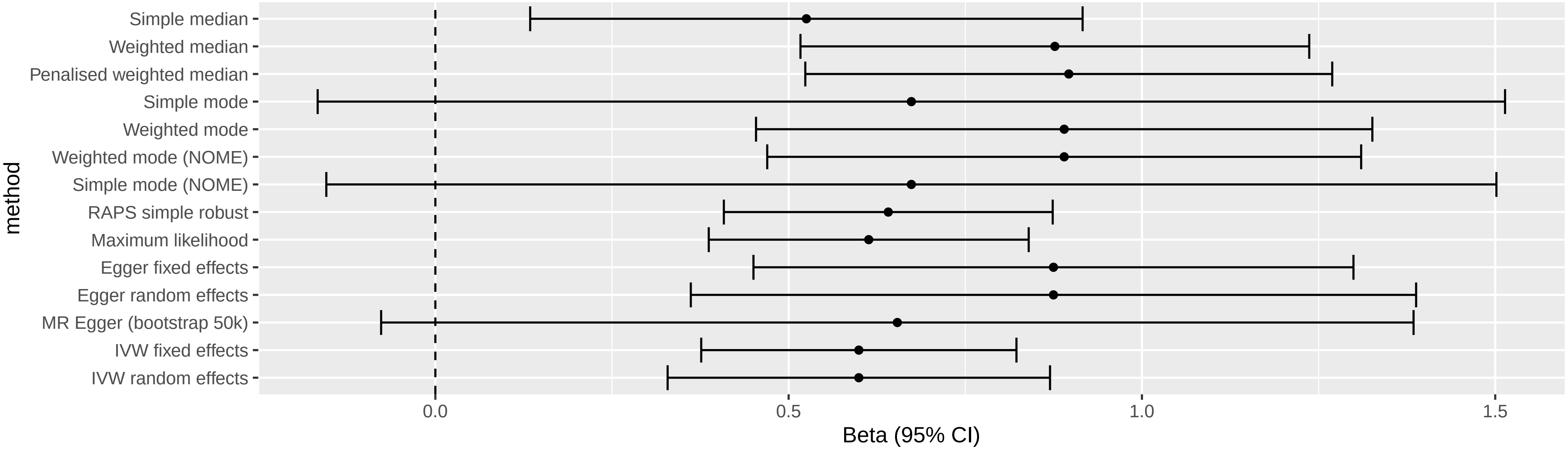

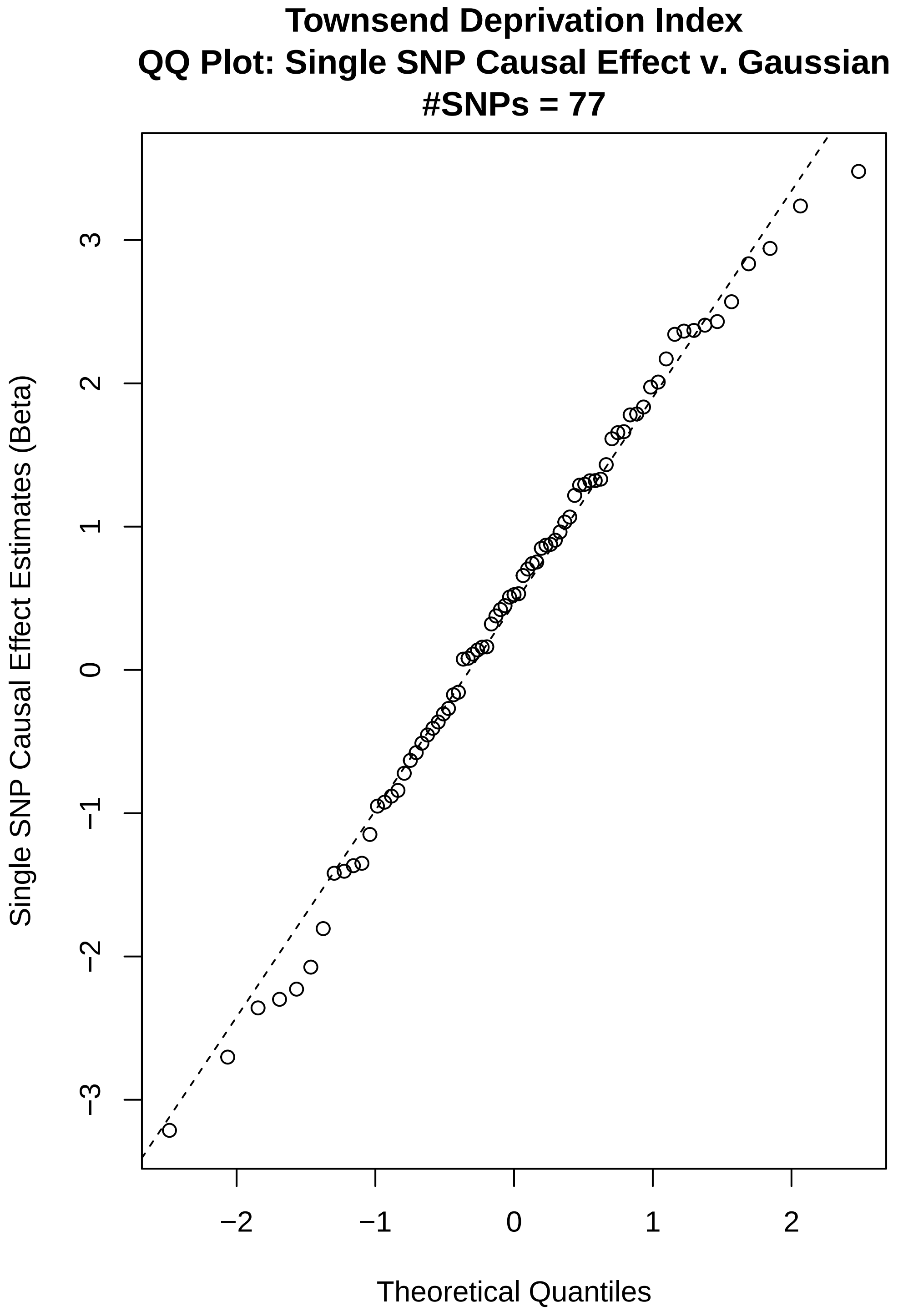
Townsend Deprivation Index
QQ Plot: Single SNP Causal Effect v. Gaussian
#SNPs = 77
Single SNP Causal Effect Estimates (Beta)
Theoretical Quantiles
−3
−2
−1
0
1
2
3
−2
−1
0
1
2

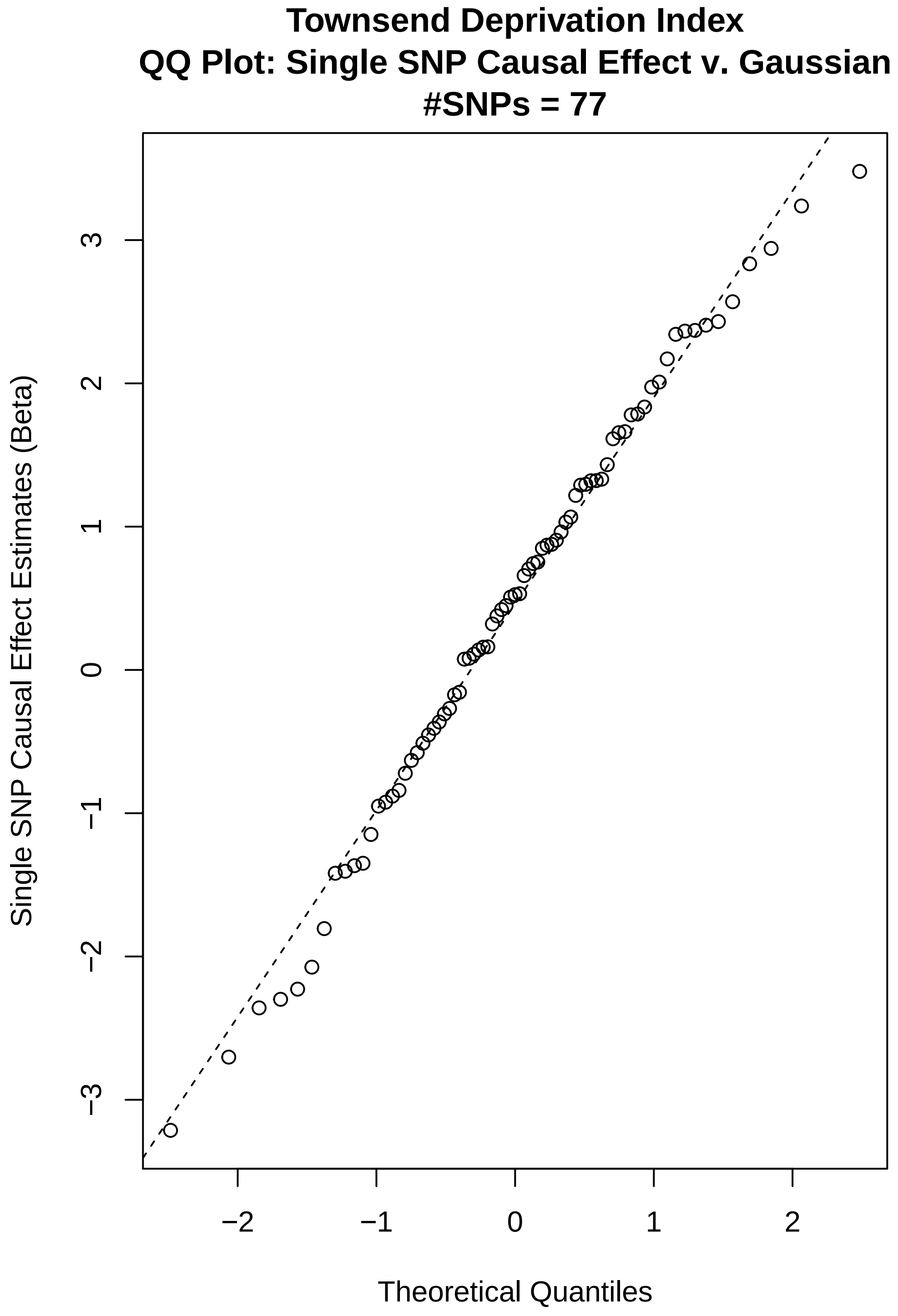
Townsend Deprivation Index
QQ Plot: Single SNP Causal Effect v. Gaussian
#SNPs = 77
Single SNP Causal Effect Estimates (Beta)
Theoretical Quantiles
−3
−2
−1
0
1
2
3
−2
−1
0
1
2

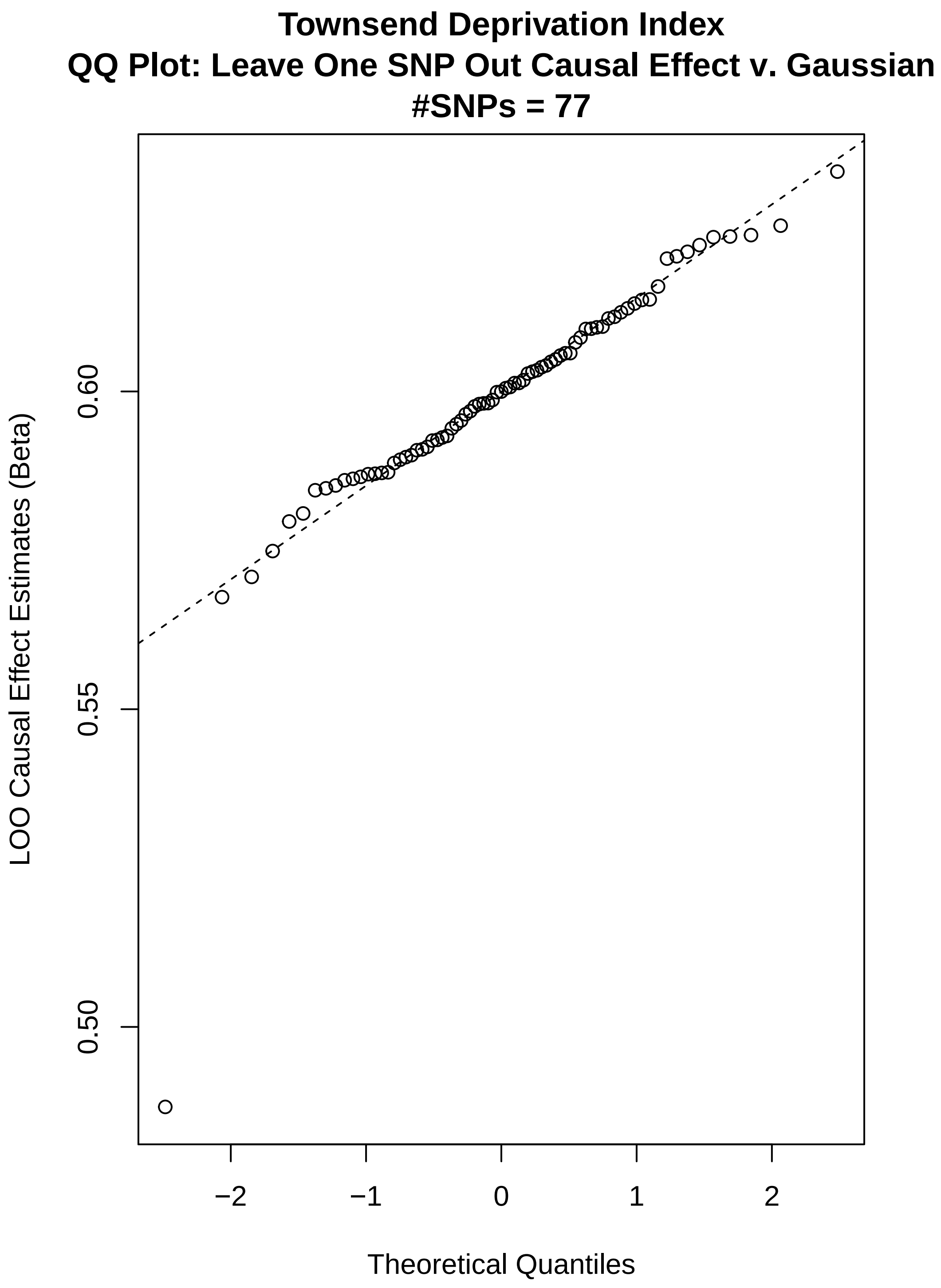
Townsend Deprivation Index
QQ Plot: Leave One SNP Out Causal Effect v. Gaussian
#SNPs = 77
LOO Causal Effect Estimates (Beta)
0.50
0.55
0.60
−2
−1
0
1
2
Theoretical Quantiles

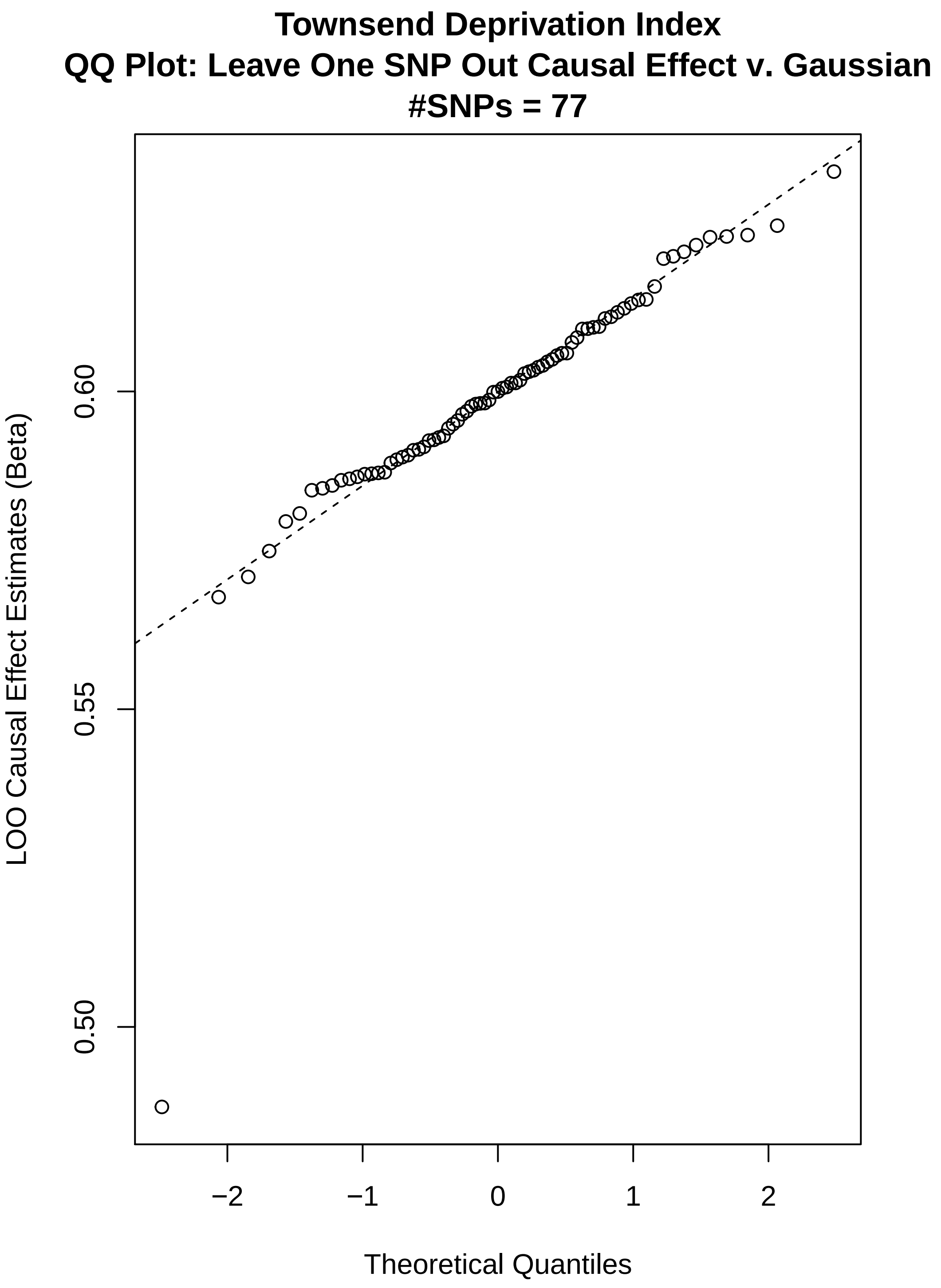
Townsend Deprivation Index
QQ Plot: Leave One SNP Out Causal Effect v. Gaussian
#SNPs = 77
LOO Causal Effect Estimates (Beta)
0.50
0.55
0.60
−2
−1
0
1
2
Theoretical Quantiles

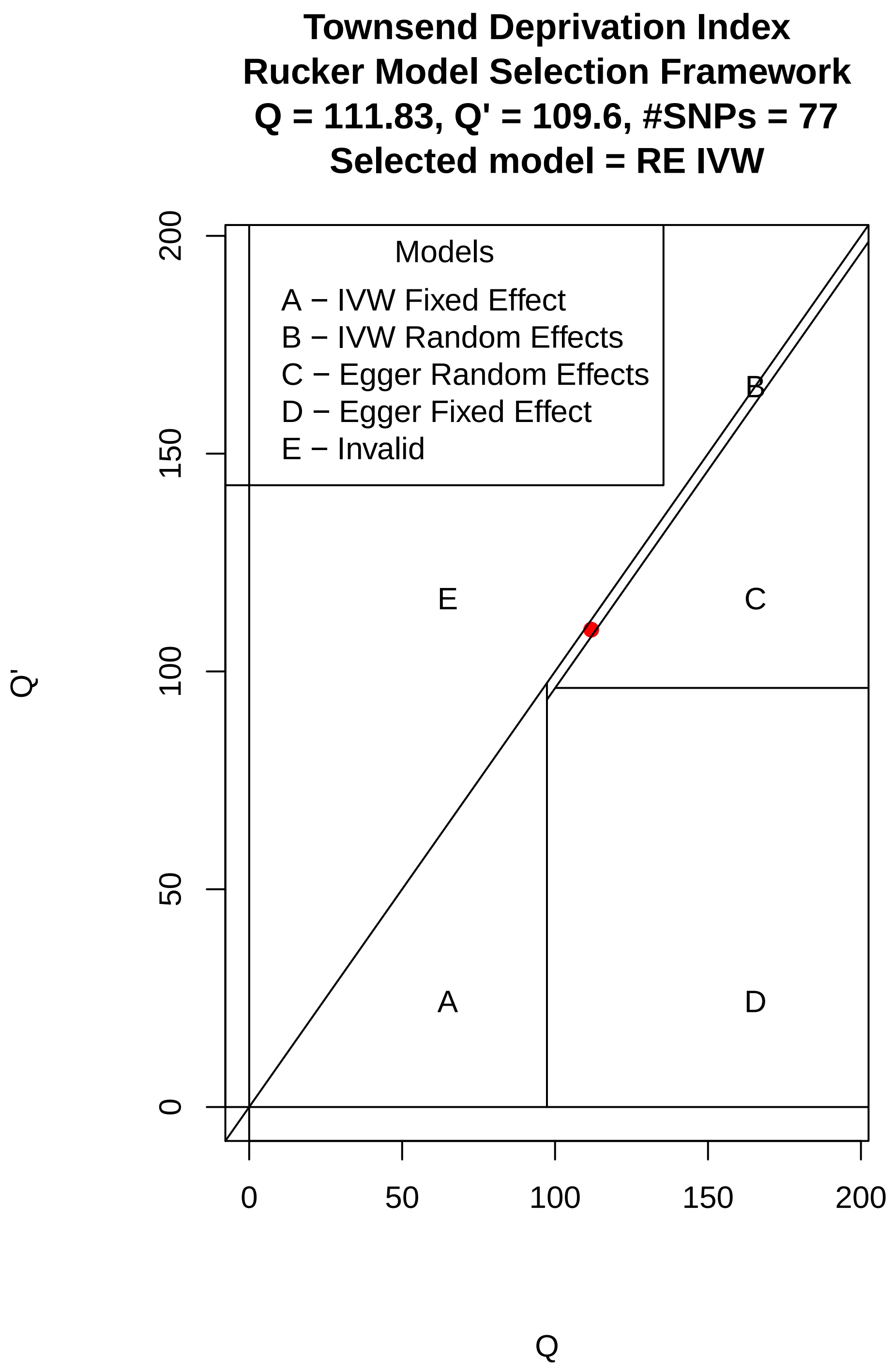
Townsend Deprivation Index
Rucker Model Selection Framework
Q = 111.83, Q' = 109.6, #SNPs = 77
Selected model = RE IVW
Models
A − IVW Fixed Effect
B − IVW Random Effects
C − Egger Random Effects
D − Egger Fixed Effect
E − Invalid
A
B
C
D
E
Q
Q'
0
50
100
150
200

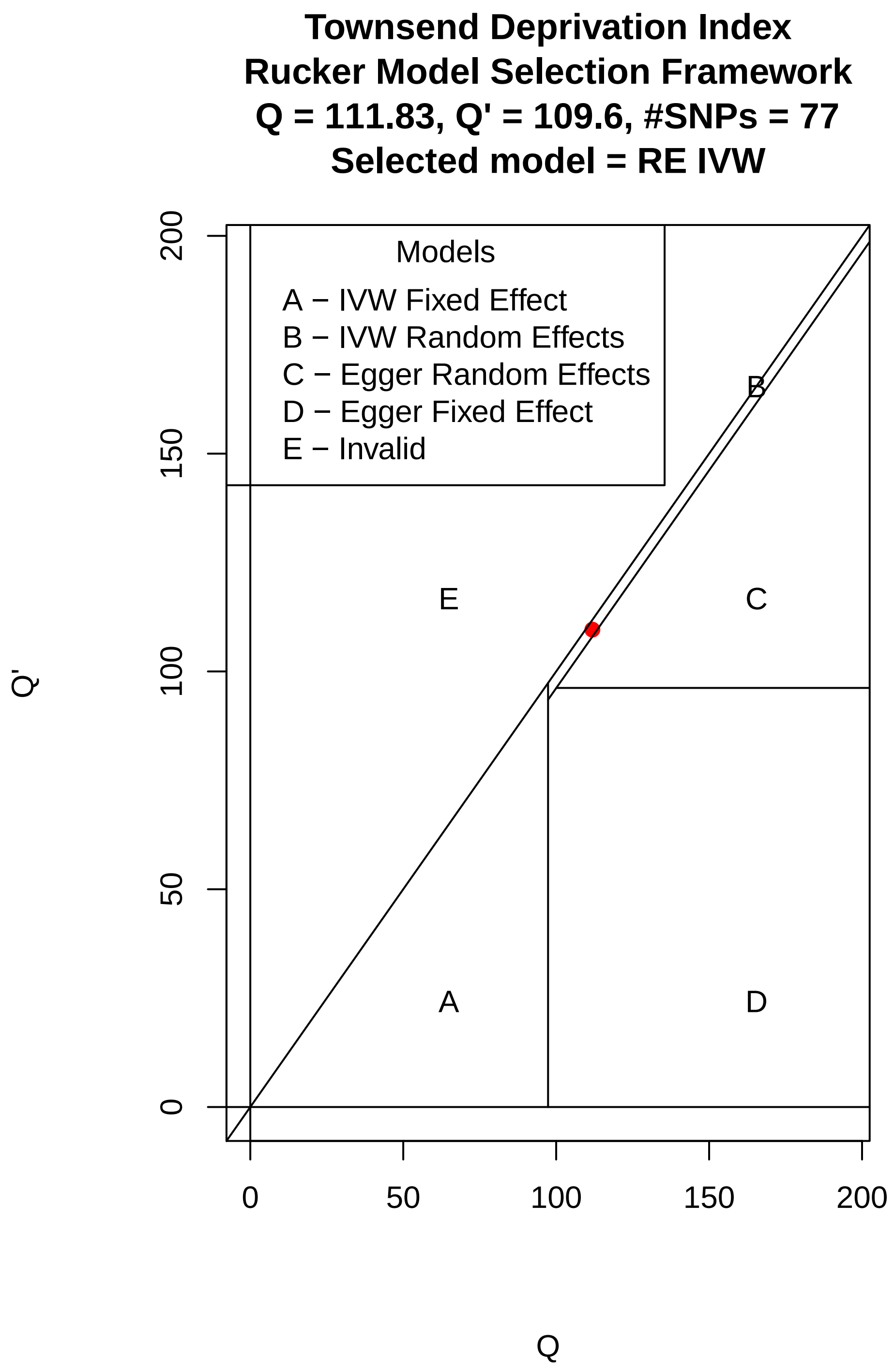
Townsend Deprivation Index
Rucker Model Selection Framework
Q = 111.83, Q' = 109.6, #SNPs = 77
Selected model = RE IVW
Models
A − IVW Fixed Effect
B − IVW Random Effects
C − Egger Random Effects
D − Egger Fixed Effect
E − Invalid
A
B
C
D
E
Q
Q'
0
50
100
150
200

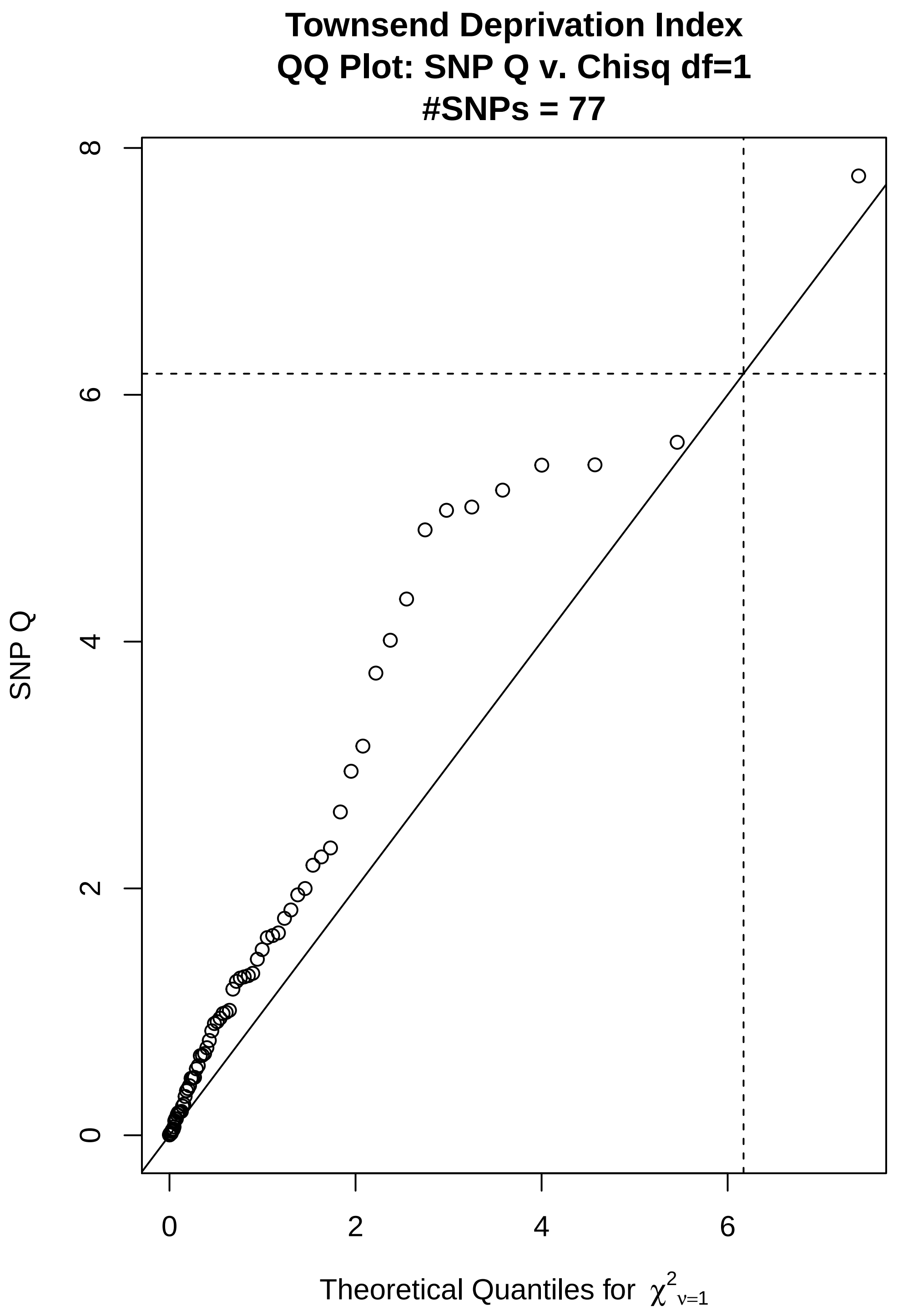
Townsend Deprivation Index
QQ Plot: SNP Q v. Chisq df=1
#SNPs = 77
SNP Q
Theoretical Quantiles for χ²ν=1
0
2
4
6
8

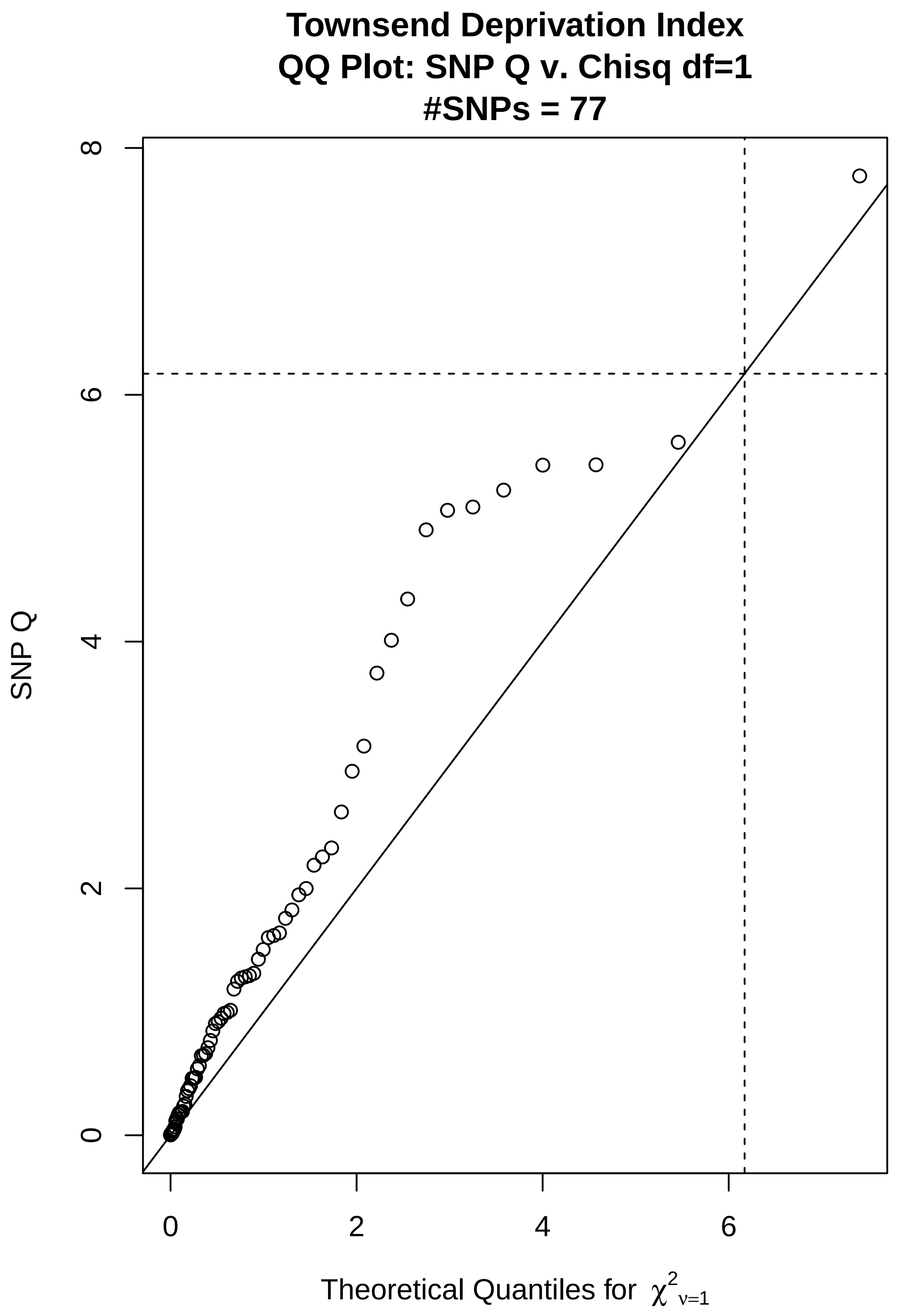
Townsend Deprivation Index
QQ Plot: SNP Q v. Chisq df=1
#SNPs = 77
SNP Q
Theoretical Quantiles for χ²ν=1
0
2
4
6
8
