# Supplementary material for: Effects of alcohol consumption on employment and social outcomes: a Mendelian randomisation study
Source: Alcohol Alcohol. 2025 Jul 18;60(5):agaf038. doi: 10.1093/alcalc/agaf038 (PMC12271571; doi:10.1093/alcalc/agaf038)
Supplement: Campbell_Green_Davies_et_al_2025_agaf038 [file campbell_green_davies_et_al_2025_agaf038.zip › Campbell_Green_Davies_et_al_2025/Male/drink/do2SampleMrAnalyses_alcoholUnitsWeekly_wins140_workHoursWeekly_wins84_ageCentreGpc.pdf]

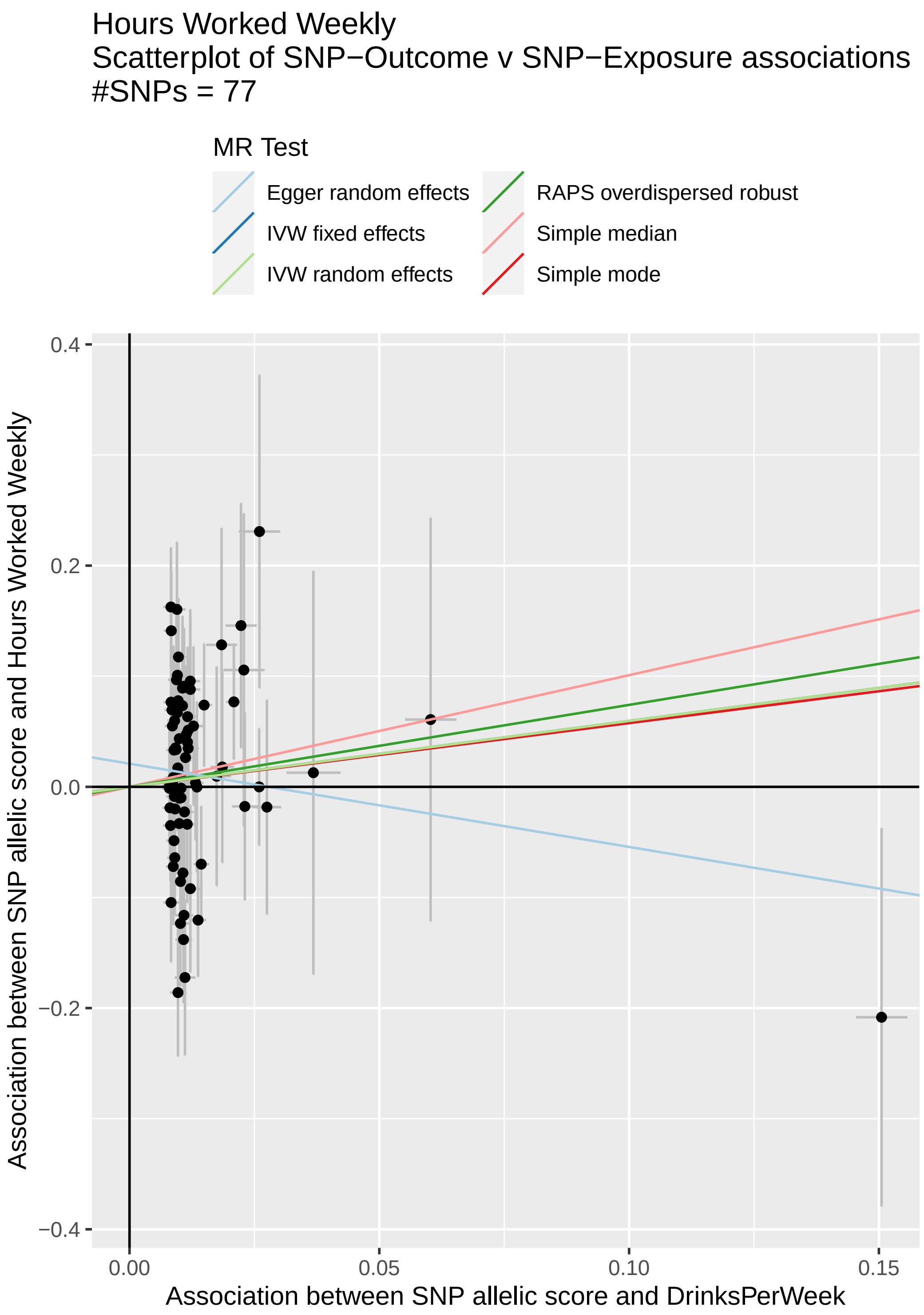
Hours Worked Weekly
Scatterplot of SNP−Outcome v SNP−Exposure associations
#SNPs = 77
MR Test
Egger random effects
IVW fixed effects
IVW random effects
RAPS overdispersed robust
Simple median
Simple mode
Association between SNP allelic score and Hours Worked Weekly
Association between SNP allelic score and DrinksPerWeek
0.4
0.2
0.0
−0.2
−0.4
0.00
0.05
0.10
0.15

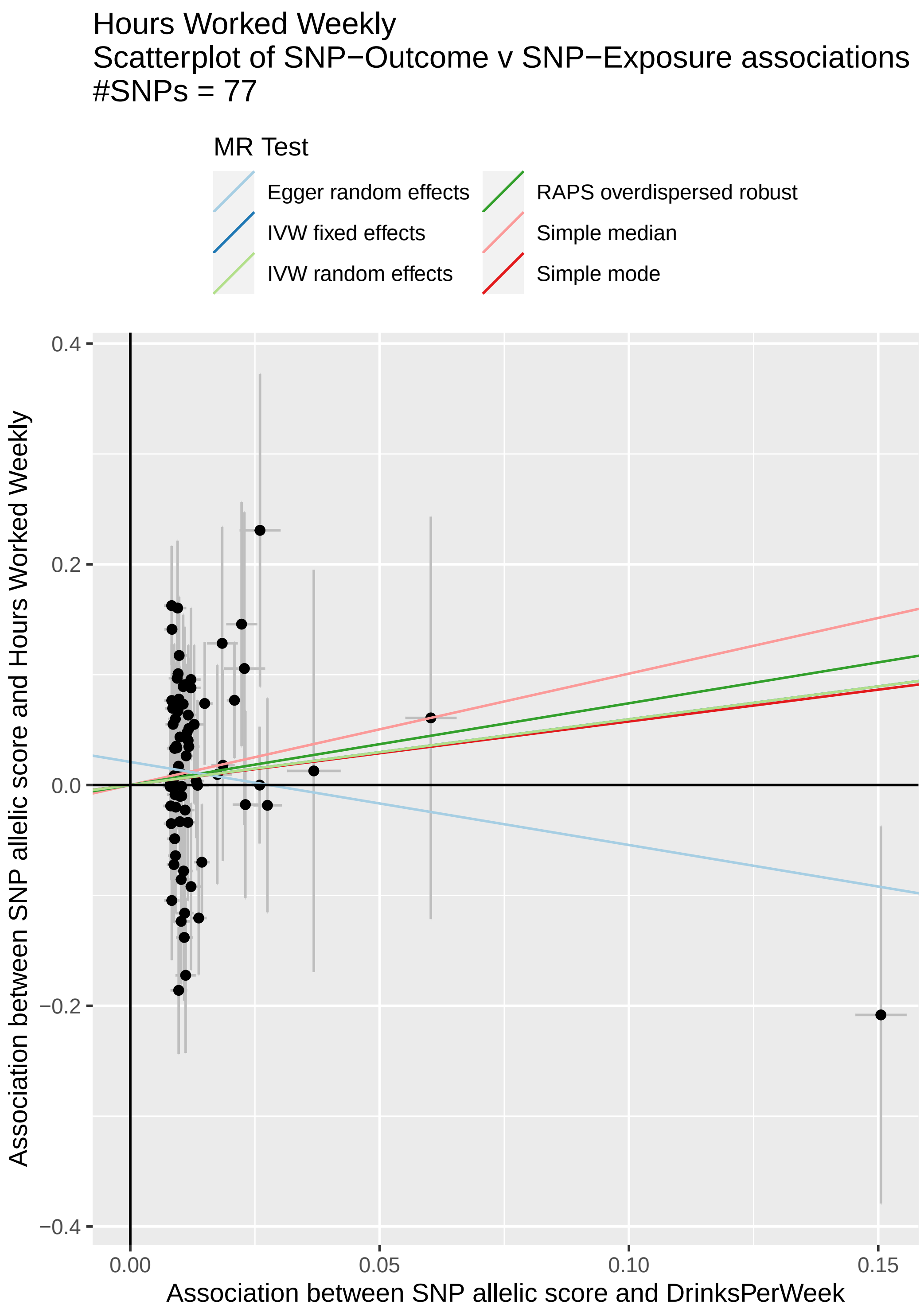
Hours Worked Weekly
Scatterplot of SNP−Outcome v SNP−Exposure associations
#SNPs = 77
MR Test
Egger random effects
IVW fixed effects
IVW random effects
RAPS overdispersed robust
Simple median
Simple mode
Association between SNP allelic score and Hours Worked Weekly
Association between SNP allelic score and DrinksPerWeek
0.4
0.2
0.0
−0.2
−0.4
0.00
0.05
0.10
0.15

Hours Worked Weekly
Causal Effect estimates for alcoholUnitsWeekly_wins140 on Hours Worked Weekly
#SNPs = 77, #Outlier SNPs removed = 0

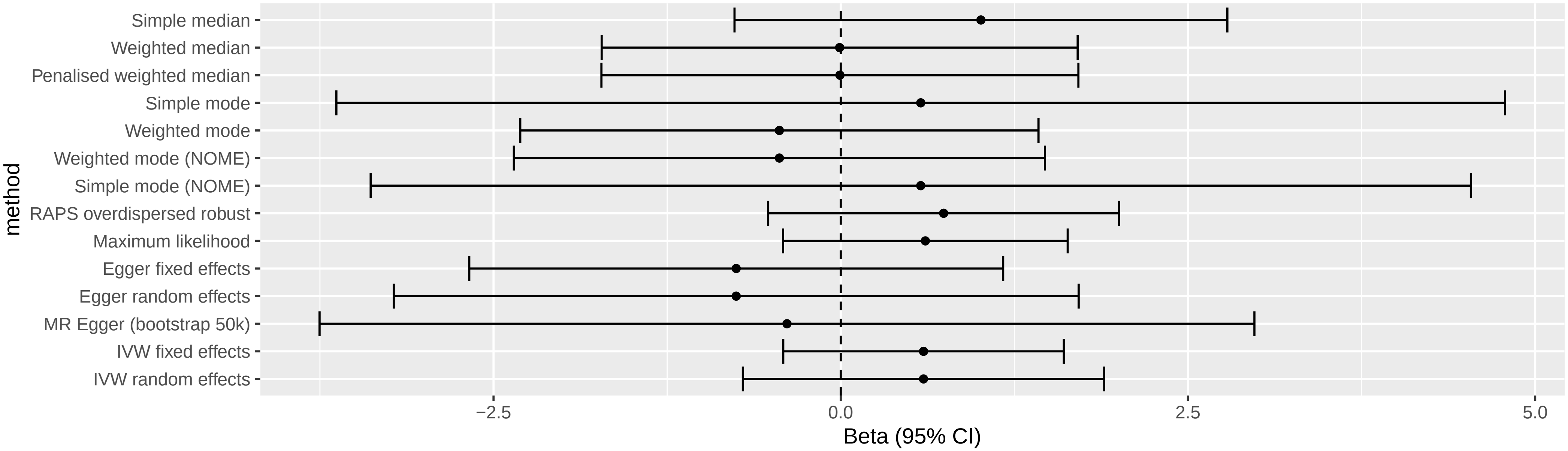

Hours Worked Weekly
Causal Effect estimates for alcoholUnitsWeekly_wins140 on Hours Worked Weekly
#SNPs = 77, #Outlier SNPs removed = 0

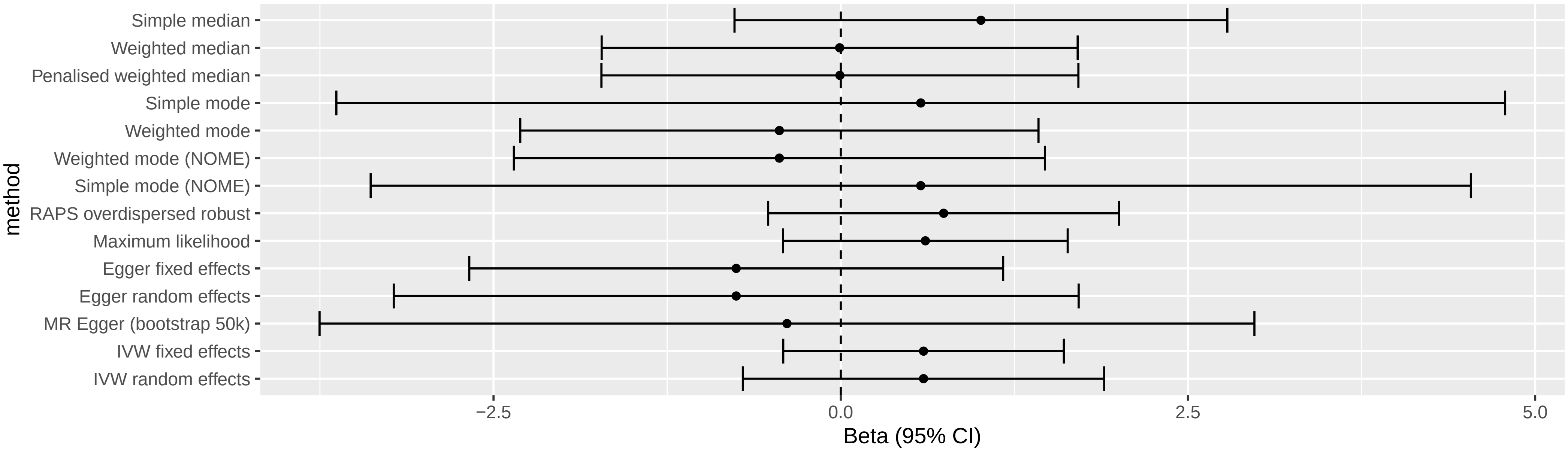

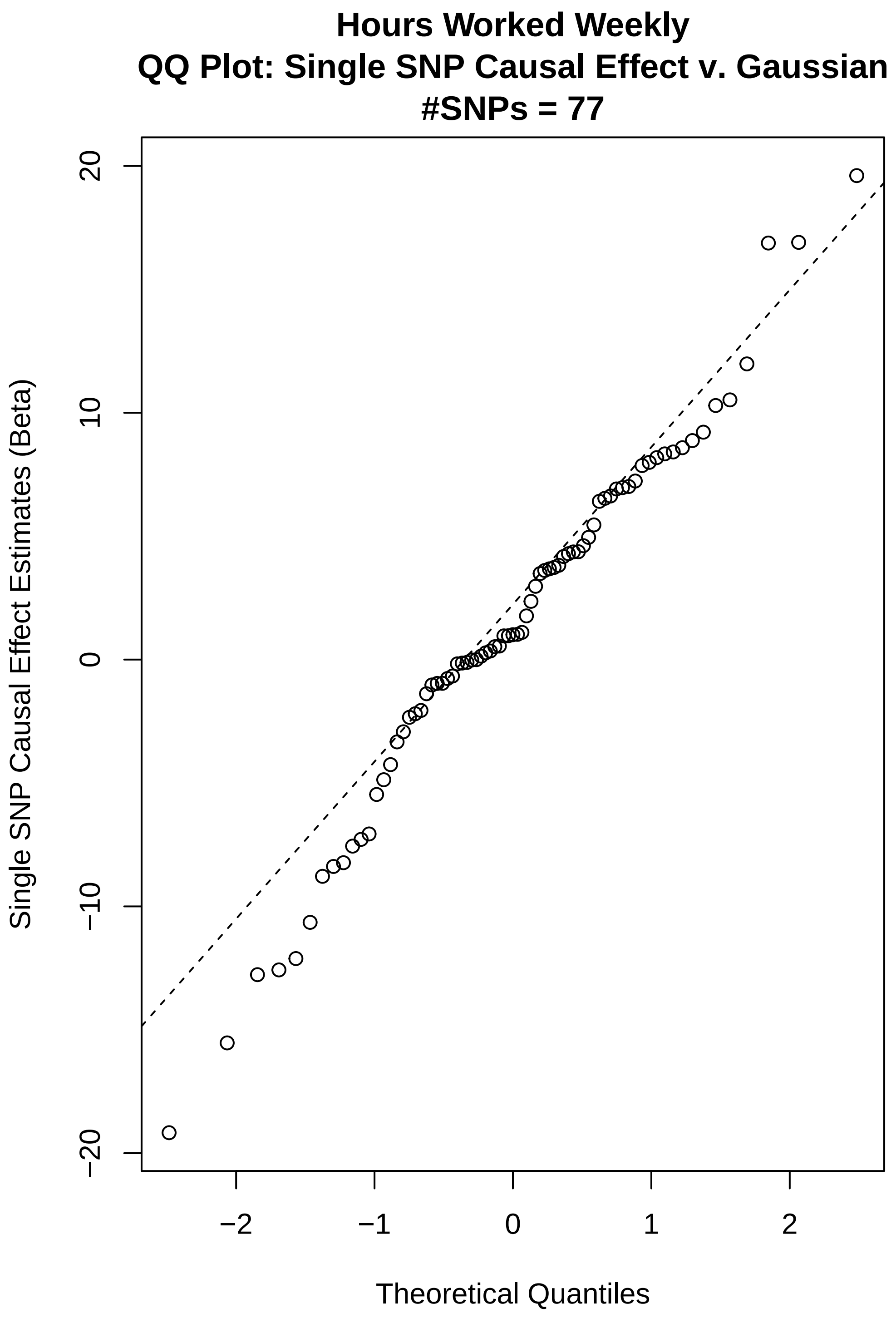

Hours Worked Weekly
QQ Plot: Single SNP Causal Effect v. Gaussian
#SNPs = 77
Single SNP Causal Effect Estimates (Beta)
Theoretical Quantiles
20
10
0
−10
−20
−2
−1
0
1
2

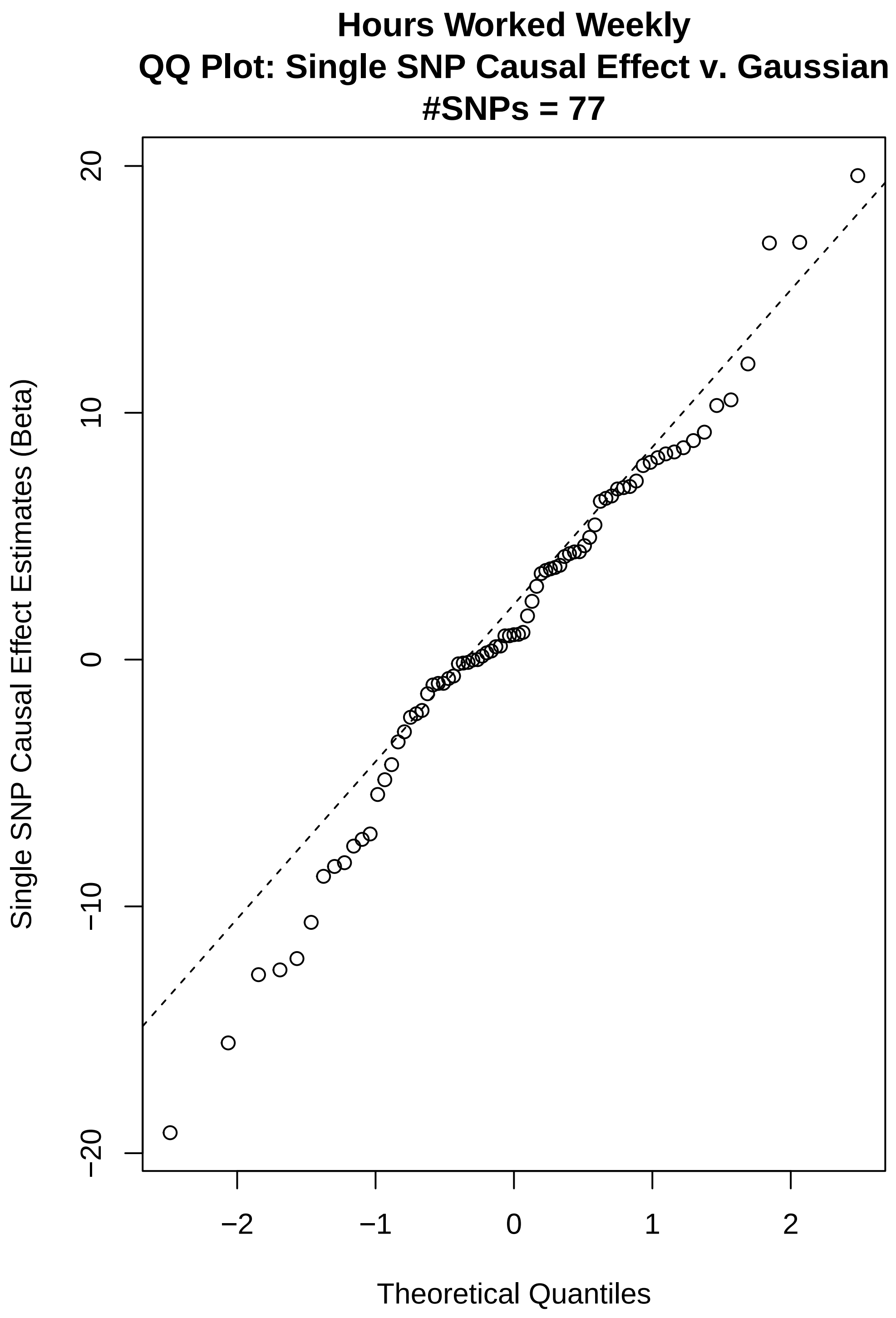

Hours Worked Weekly
QQ Plot: Single SNP Causal Effect v. Gaussian
#SNPs = 77
Single SNP Causal Effect Estimates (Beta)
Theoretical Quantiles
20
10
0
−10
−20
−2
−1
0
1
2

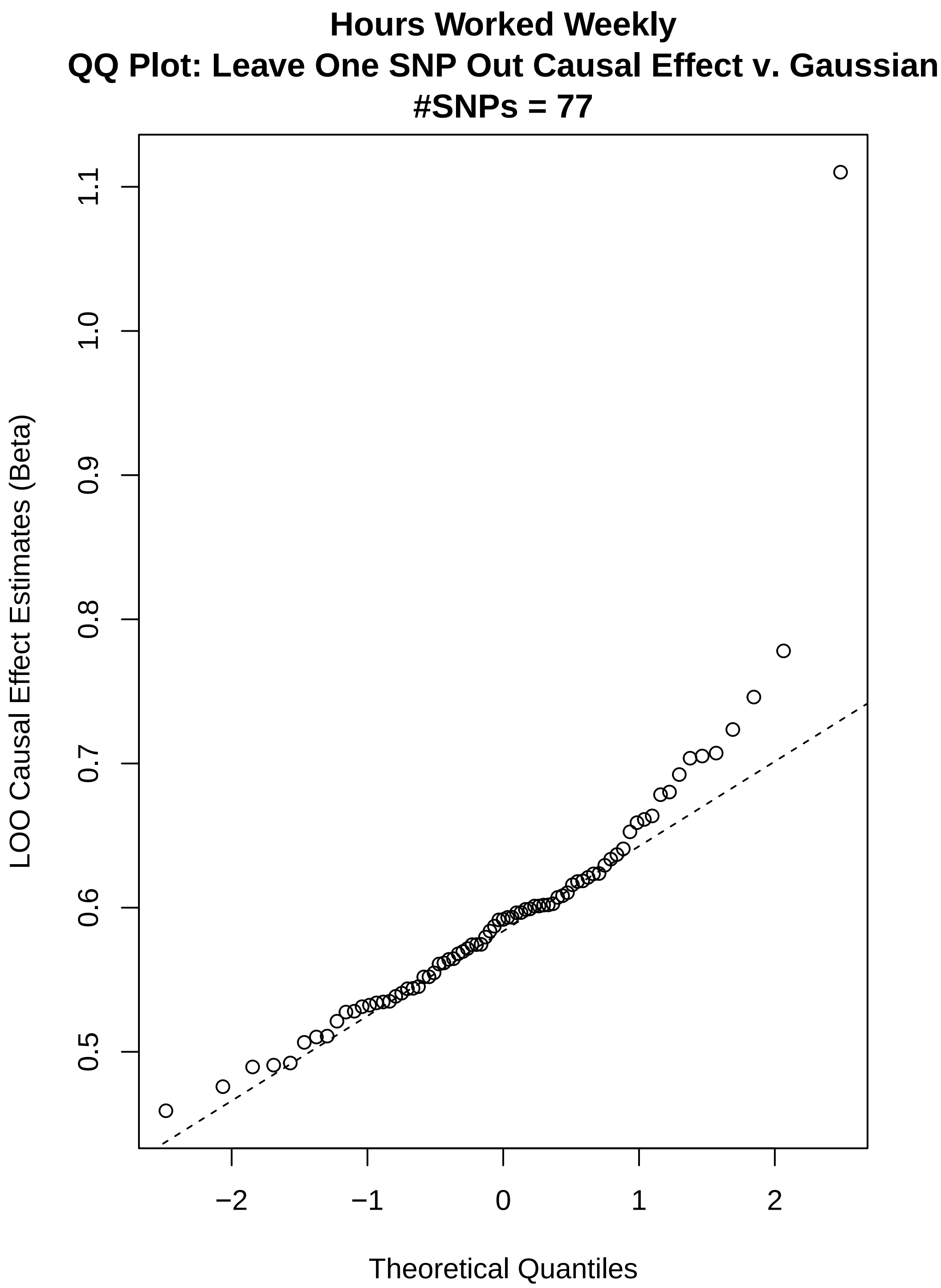
Hours Worked Weekly
QQ Plot: Leave One SNP Out Causal Effect v. Gaussian
#SNPs = 77
LOO Causal Effect Estimates (Beta)
Theoretical Quantiles
1.1
1.0
0.9
0.8
0.7
0.6
0.5
−2
−1
0
1
2

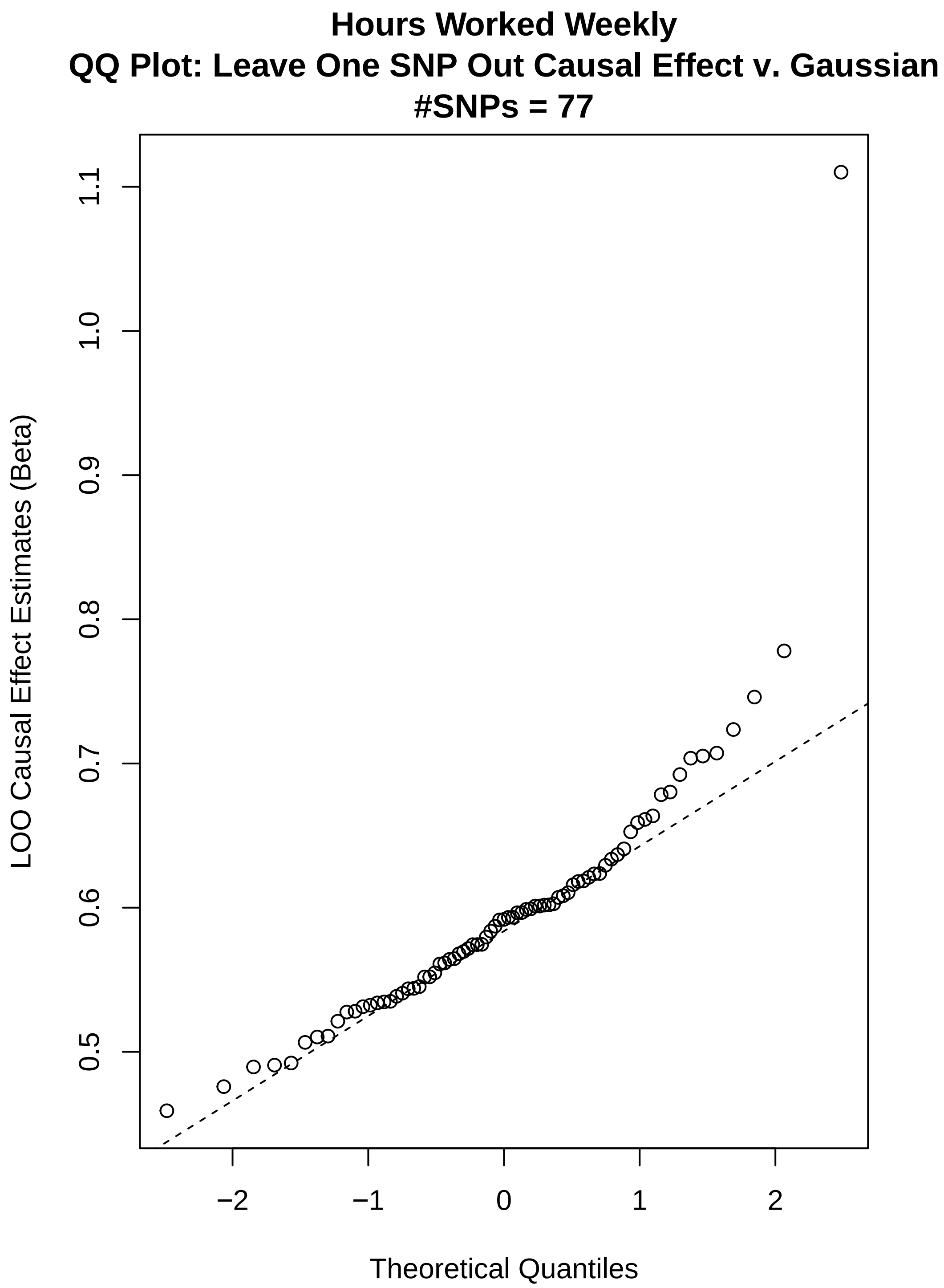
Hours Worked Weekly
QQ Plot: Leave One SNP Out Causal Effect v. Gaussian
#SNPs = 77
LOO Causal Effect Estimates (Beta)
Theoretical Quantiles
1.1
1.0
0.9
0.8
0.7
0.6
0.5
−2
−1
0
1
2

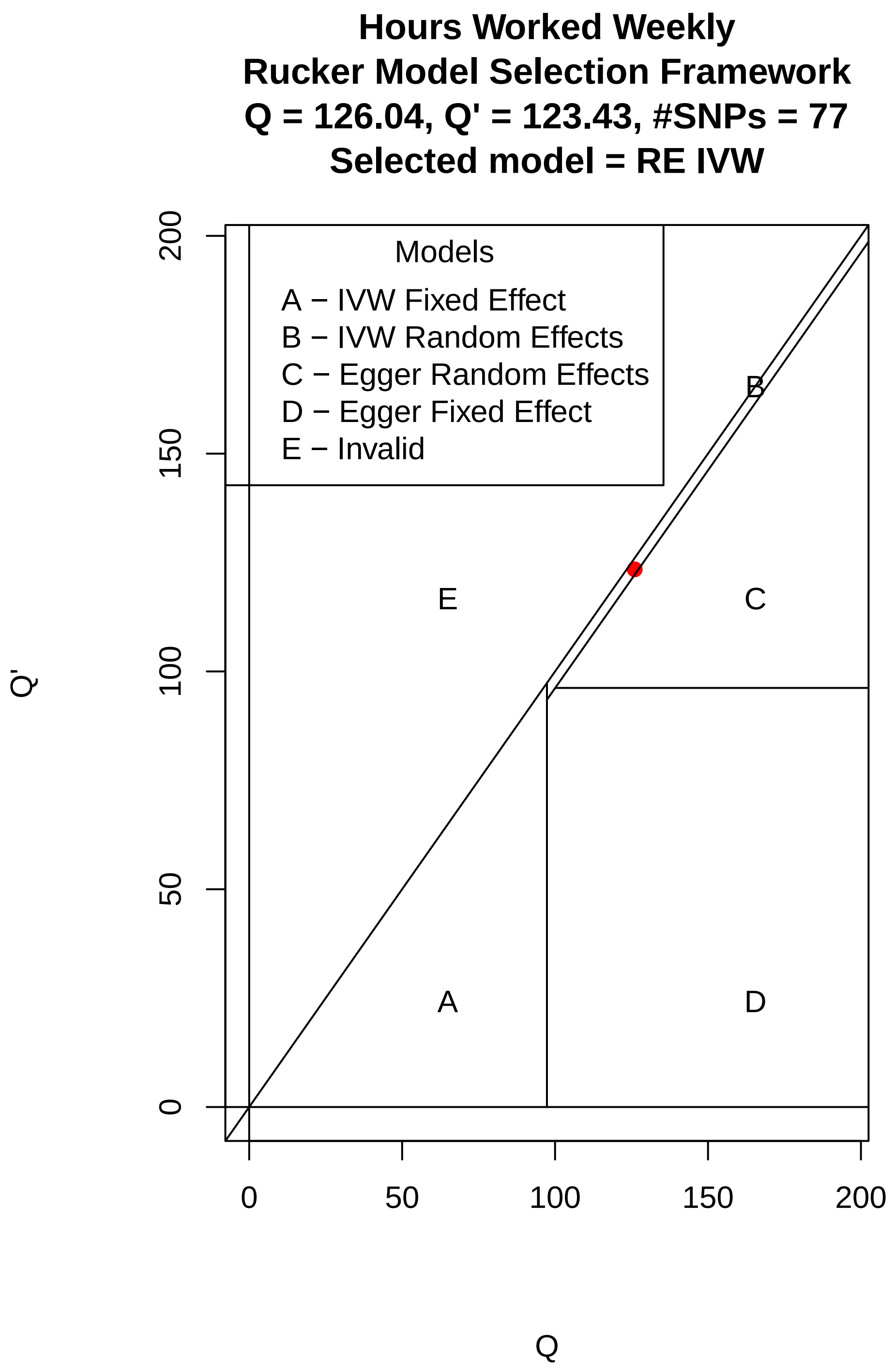
Hours Worked Weekly
Rucker Model Selection Framework
Q = 126.04, Q' = 123.43, #SNPs = 77
Selected model = RE IVW
Models
A − IVW Fixed Effect
B − IVW Random Effects
C − Egger Random Effects
D − Egger Fixed Effect
E − Invalid
B
E
C
A
D
Q'
Q
0
50
100
150
200

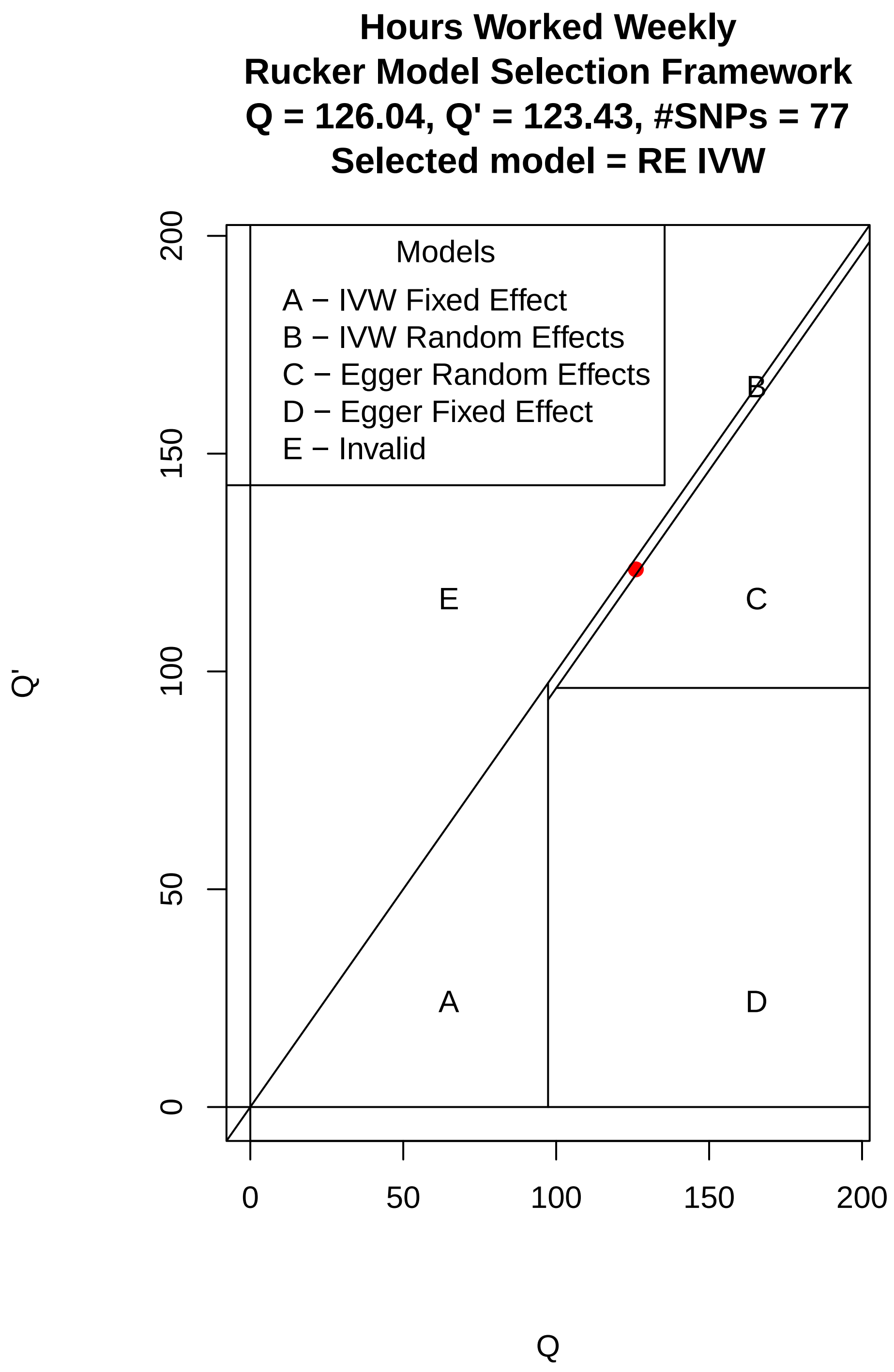
Hours Worked Weekly
Rucker Model Selection Framework
Q = 126.04, Q' = 123.43, #SNPs = 77
Selected model = RE IVW
Models
A − IVW Fixed Effect
B − IVW Random Effects
C − Egger Random Effects
D − Egger Fixed Effect
E − Invalid
B
E
C
A
D
Q'
Q
0
50
100
150
200

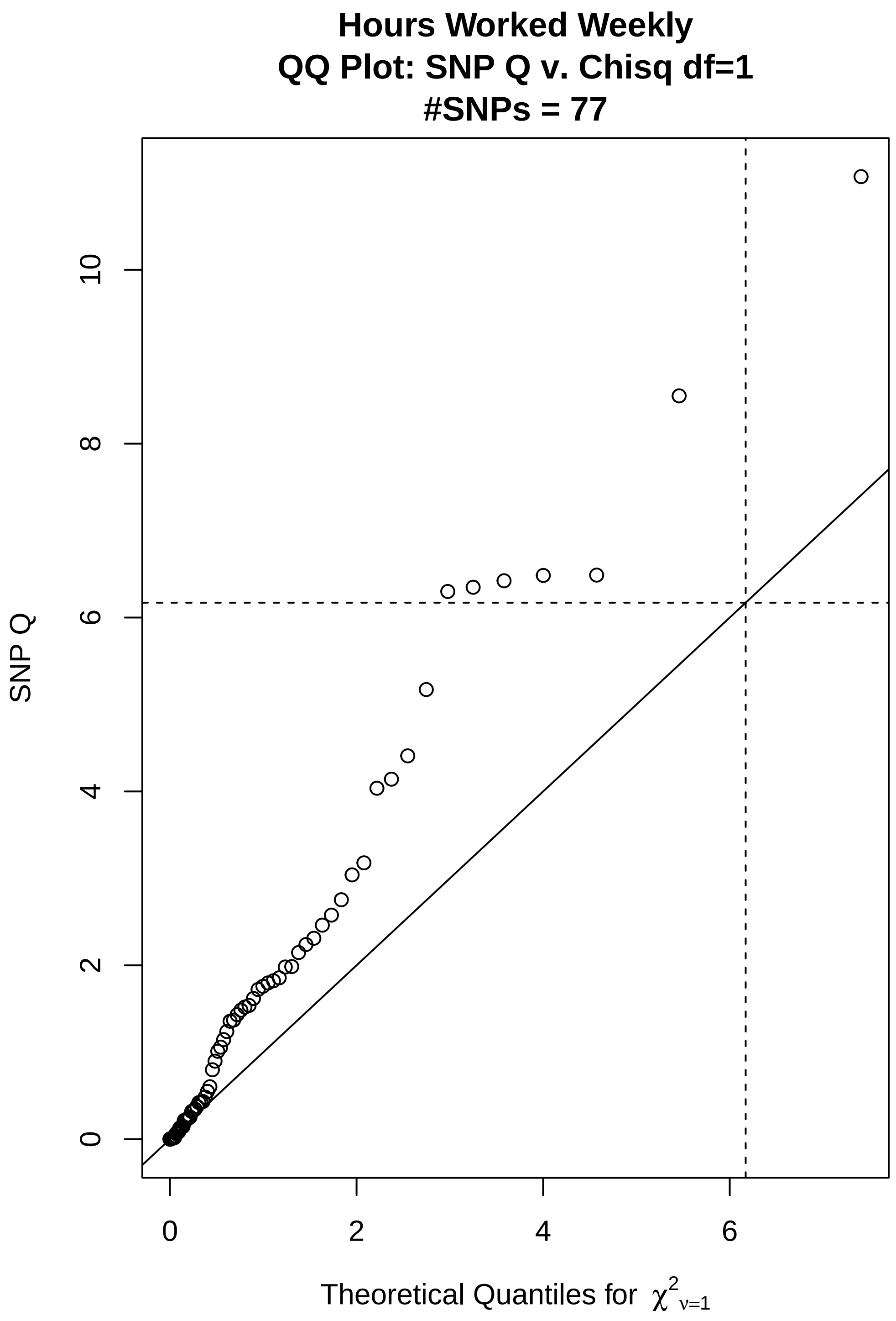

Hours Worked Weekly
QQ Plot: SNP Q v. Chisq df=1
#SNPs = 77
SNP Q
Theoretical Quantiles for $\chi^2_{\nu=1}$
0
2
4
6
8
10

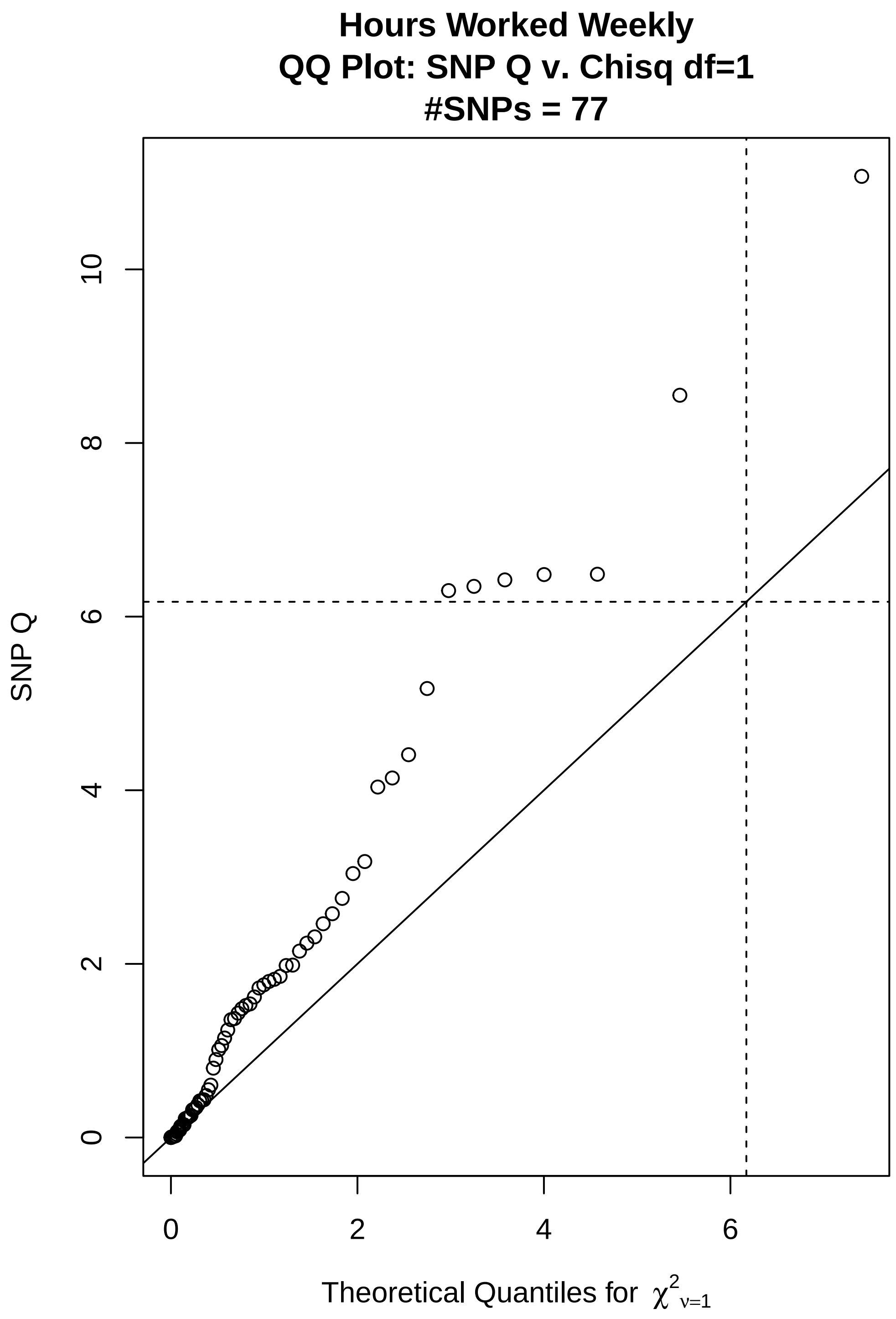

Hours Worked Weekly
QQ Plot: SNP Q v. Chisq df=1
#SNPs = 77
SNP Q
Theoretical Quantiles for $\chi^2_{\nu=1}$
0
2
4
6
8
10
